# Photocatalytic Hydroaminoalkylation of Styrenes with Unprotected Primary Alkylamines

**DOI:** 10.1021/jacs.1c07401

**Published:** 2021-09-20

**Authors:** Hannah E. Askey, James D. Grayson, Joshua D. Tibbetts, Jacob C. Turner-Dore, Jake M. Holmes, Gabriele Kociok-Kohn, Gail L. Wrigley, Alexander J. Cresswell

**Affiliations:** † Department of Chemistry, University of Bath, Claverton Down, Bath BA2 7AY, U.K.; ‡ Materials and Chemical Characterisation Facility (MC^2^), University of Bath, Claverton Down, Bath BA2 7AY, U.K.; § Oncology R&D, Research & Early Development, AstraZeneca, Darwin Building, 310, Cambridge Science Park, Milton Road, Cambridge CB4 0WG, U.K.

## Abstract

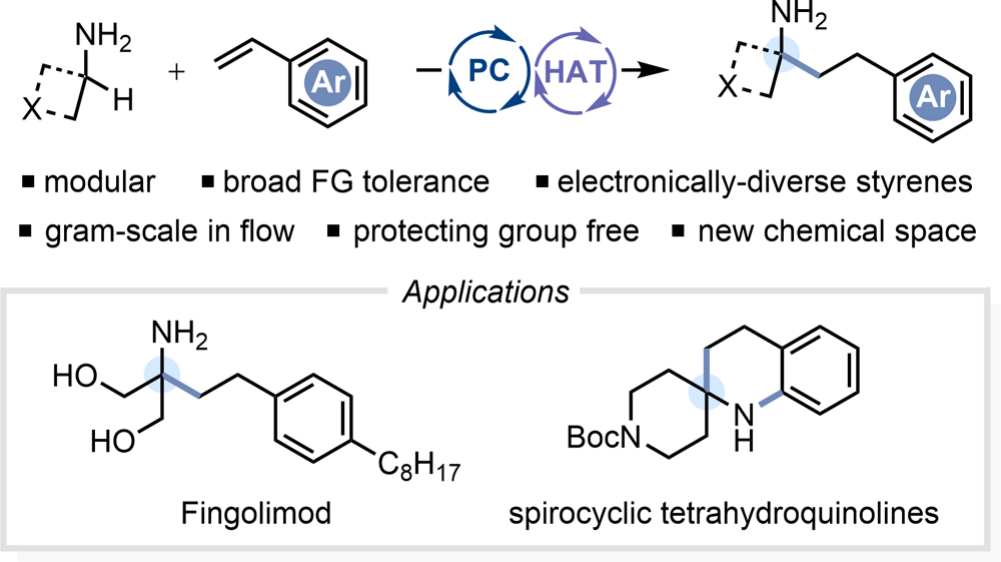

Catalytic, intermolecular hydroaminoalkylation (HAA) of styrenes provides a powerful disconnection for pharmacologically relevant γ-arylamines, but current methods cannot utilize unprotected primary alkylamines as feedstocks. Metal-catalyzed HAA protocols are also highly sensitive to α-substitution on the amine partner, and no catalytic solutions exist for α-tertiary γ-arylamine synthesis via this approach. We report a solution to these problems using organophotoredox catalysis, enabling a direct, modular, and sustainable preparation of α-(di)substituted γ-arylamines, including challenging electron-neutral and moderately electron-rich aryl groups. A broad range of functionalities are tolerated, and the reactions can be run on multigram scale in continuous flow. The method is applied to a concise, protecting-group-free synthesis of the blockbuster drug Fingolimod, as well as a phosphonate mimic of its *in vivo* active form (by iterative α-C–H functionalization of ethanolamine). The reaction can also be sequenced with an intramolecular *N*-arylation to provide a general and modular access to valuable (spirocyclic) 1,2,3,4-tetrahydroquinolines and 1,2,3,4-tetrahydronaphthyridines. Mechanistic and kinetic studies support an irreversible hydrogen atom transfer activation of the alkylamine by the azidyl radical and some contribution from a radical chain. The reaction is photon-limited and exhibits a zero-order dependence on amine, azide, and photocatalyst, with a first-order dependence on styrene.

## Introduction

**Figure 1:**
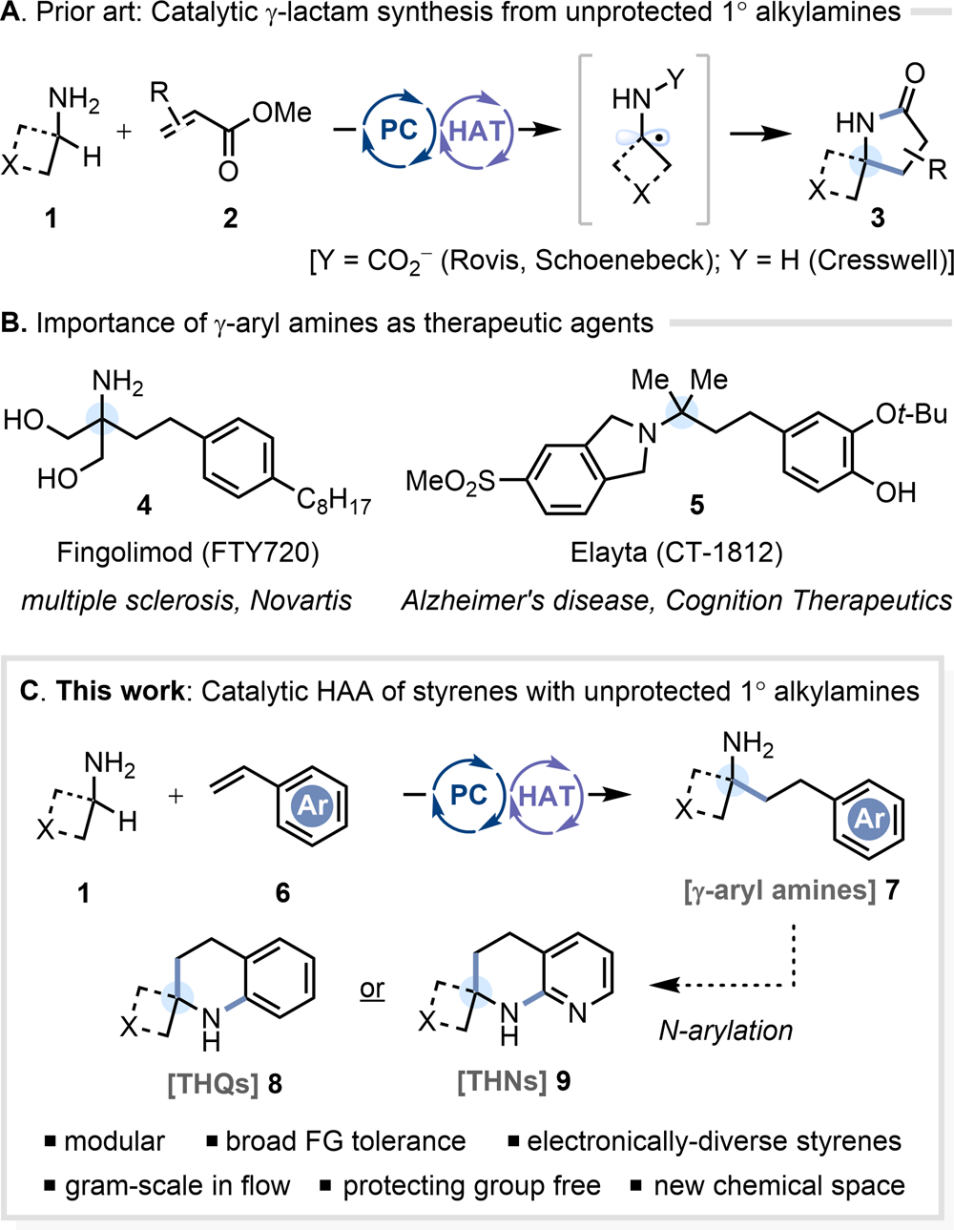
(A) Prior art for catalytic γ-lactam synthesis from primary alkylamines. (B) Importance of γ-arylamines. (C) This work.

Aliphatic amines and (semi)saturated azacycles are privileged motifs in pharmaceuticals, agrochemicals, biological probes, and other functional molecules,^1^ and the development of more efficient methods for their synthesis is a research priority.^2^ Perhaps the most attractive and atom-economical approach for the construction of α-alkylated amines is the net insertion of an alkene into an amine α-C–H bond, often termed a hydroaminoalkylation (HAA) reaction.^3^ For secondary^4^ and tertiary^5^ amines, the catalytic HAA of non-electrophilic^6^ alkenes has been dominated by early transition-metal-based catalysts. These reactions are typically sensitive to the substitution α to nitrogen, with the majority of reports focusing on *N*-methyl group functionalization, and linear selectivity being a particular challenge.^4e^ Linear-selective alkene HAAs with non-electrophilic alkenes are more common for late transition metal catalysis,^7^ but there is a need for specially tailored directing groups on the amine nitrogen. A different strategy altogether for alkene HAA deploys nucleophilic α-amino radicals generated via photoredox catalysis,^8^ but this approach is typically limited to suitably electrophilic alkenes such as acrylates or vinylpyridines.^3b,8,9^ For example, we recently reported a photoredox-catalyzed formation of γ-lactams **3** from primary alkylamines **1** and acrylates **2**,^9d^ and Rovis, Schoenebeck, and co-workers developed a similar process^9i^ based on *in situN*-protection of the amine with CO_2_ (Figure 1A). Despite the above successes, the HAA of electronically unbiased styrenes with primary alkylamines lacks a general and practical solution,^10^ although styrene HAA reactions have recently been developed with tertiary^11a,11b^ and (protected) secondary^11c^ amines. With primary amines, the only reported intermolecular examples have utilized 2-pyridyl directing groups on the amine nitrogen^12^ (with Ru or Ir catalysts) or *N*-silyl protecting groups at high temperature (>140 °C with Ti or Zr catalysts).^4d,13^ The use of unprotected primary alkylamines in catalytic HAA with non-electrophilic alkenes is currently limited to simple, unfunctionalized examples in the *intramolecular* mode (110–145 °C, 5–20 mol% Ti catalyst).^14^ Given the importance of γ-arylamines and their occurrence in several clinically approved drugs [e.g., Fingolimod **4**, Elayta **5** (Figure 1B), Cinacalcet, Fendiline, Pheniramine], a generally applicable catalytic HAA of simple styrenes with unprotected primary alkylamines would constitute a significant advance. We report a solution to this problem using visible-light photoredox catalysis in combination with hydrogen atom transfer (HAT) catalysis.^15^ This enables a direct and modular synthesis of pharmacologically relevant γ-arylamines **7**, including Fingolimod **4** and analogues thereof. Further application to the expedient synthesis of (spirocyclic) 1,2,3,4-tetrahydroquinolines **8** and 1,2,3,4-tetrahydronaphthyridines **9** is also described (Figure 1C).

## Results and Discussion

### Reaction Optimization

**Figure 2:**
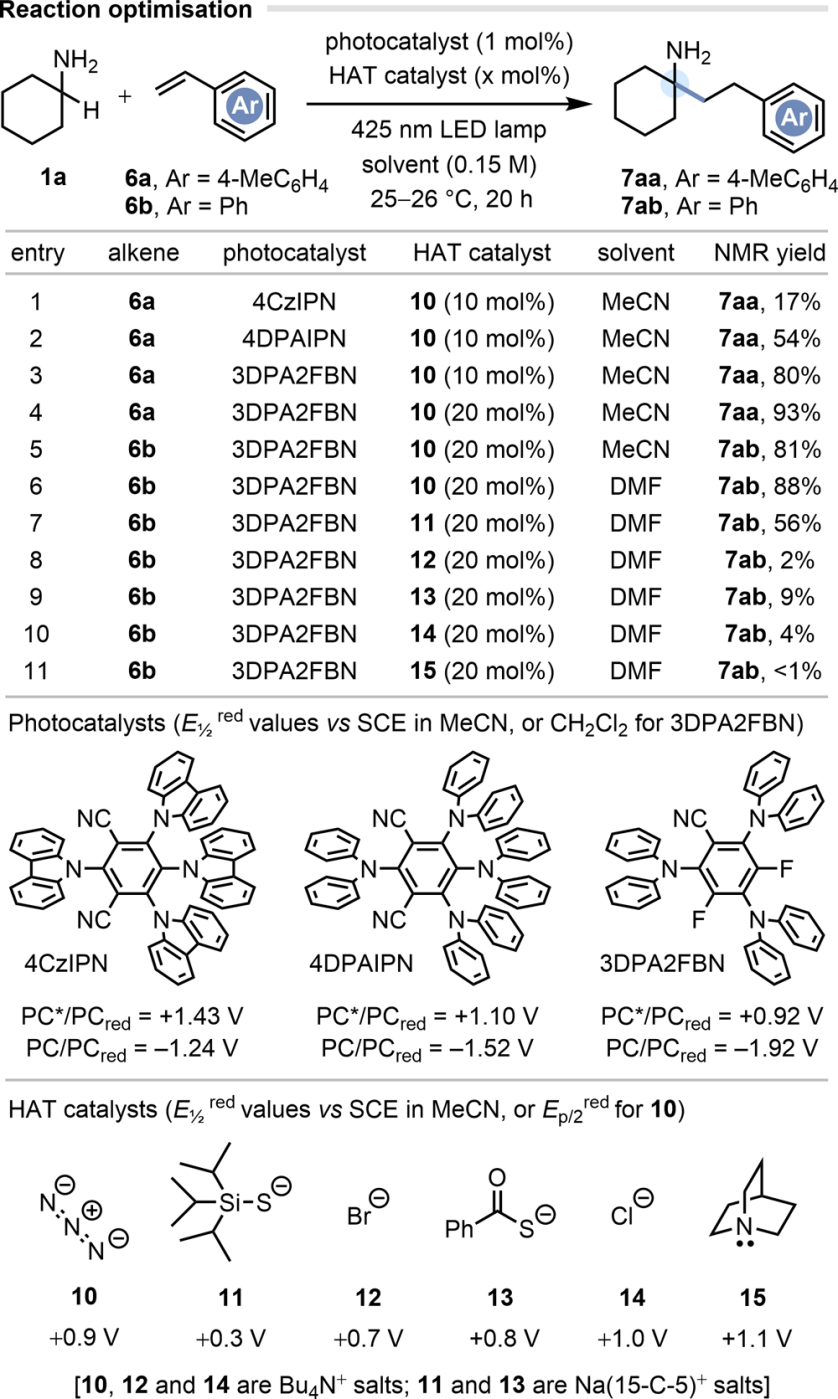
Yields measured by ^1^H NMR against 1,3,5-trimethoxybenzene as an internal standard. Reference for redox potentials of photocatalysts.^23^ References for oxidation potentials of HAT catalysts: **10**, ref (9d); **11**, ref (27); **12**, ref (16); **13**, ref (25); **14**, ref (16); and **15**, ref (9i).

The generation of α-amino radicals directly from primary alkylamines **1** by single-electron oxidation followed by deprotonation is complicated by the high oxidation potential of the nitrogen lone pair (*E*_p/2_^red^ = +1.53 V vs SCE in MeCN for cyclohexylamine^9d^),^16^ and the possibility for aminium radicals to form *N*-centered aminyl radicals by N–H cleavage.^17^ We recently found that azide ion (N_3_^–^) can serve as an effective catalytic mediator in the photoredox-catalyzed formation of α-amino radicals from primary alkylamines.^9d^ Chemoselective oxidation of azide ion (*E*_p/2_^red^ = +0.87 V vs SCE in MeCN^9d^) by the excited photocatalyst 2,4,5,6-tetra(9*H*-carbazol-9-yl)isophthalonitrile (4CzIPN)^18^ serves to generate the highly electrophilic azidyl radical (N_3_^•^), that can participate in a polarity-matched^19^ HAT process with the weak α-C–H bond of a primary alkylamine (BDE = 89–91 ± 2 kcal mol^–1^).^20^ The resultant α-amino radicals are highly nucleophilic and they engage successfully with electrophilic alkenes such as acrylates^9d^ and vinyl phosphonates.^9b^ To determine if non-electrophilic alkenes could be accommodated as reaction partners, we irradiated *p*-methylstyrene **6a** with cyclohexylamine **1a** in MeCN at 425 nm, using 4CzIPN as the photocatalyst and tetrabutylammonium azide (Bu_4_N^+^N_3_^–^) **10** as the HAT catalyst (Figure 2). Only a very low level of reactivity was found, with the HAA product **7aa** formed in 17% NMR yield (entry 1). We reasoned that photocatalyst turnover may be the issue, given that the reduction of a putative benzylic radical by the reduced photocatalyst (PC^–•^) should be far less facile than with an electrophilic alkene acceptor [i.e., *E*_1/2_^red^ = −1.43 V vs SCE for ^•^CH_2_Ph/^–^CH_2_Ph in MeCN,^21^ compared to *E*_1/2_^red^ = −0.63 V vs SCE for ^•^CH_2_CO_2_Et/^–^CH_2_CO_2_Et in MeCN^22^]. On that basis, we assayed photocatalysts known to be more strongly reducing in their reduced form. 4DPAIPN gave enhanced reactivity (entry 2), but the most promising result was obtained with 3DPA2FBN [*E*_1/2_ (PC/PC^–•^) = −1.92 V vs SCE in CH_2_Cl_2_^23^] (entry 3). Further experimentation showed that doubling the loading of azide ion to 20 mol% enhanced the yield (entry 4), which may be a consequence of the reduced excited state lifetime of 3DPA2FBN (*k*_p_^–1^ = 4.2 ns) relative to 4CzIPN (*k*_p_^–1^ = 12.7 ns) (i.e., competition of bimolecular quenching by N_3_^–^ with unimolecular fluorescence from ^1^PC*).^23^ After switching the alkene partner to styrene **6b** for further optimization, giving a somewhat reduced yield (entry 5), we changed the reaction solvent to dimethylformamide (DMF) from acetonitrile (MeCN) (entry 6). Finally, we surveyed a series of other commonly used HAT catalysts (**11**–**15**), to gauge whether or not the use of azide ion **10** conferred unique reactivity. Although tri(isopropyl)silanethiolate **11** (entry 7) did give appreciable turnover (56% NMR yield), it significantly underperformed azide ion **10**. Bromide ion **12**,^24^ thiobenzoate **13**,^25^ chloride ion **14**,^26^ and quinuclidine **15**(9f,9i) all gave negligible reactivity (entries 8–11). Control experiments verified that 3DPA2FBN, visible light, and azide catalyst are all necessary components for successful HAA.

### Amine Scope

**Figure 3:**
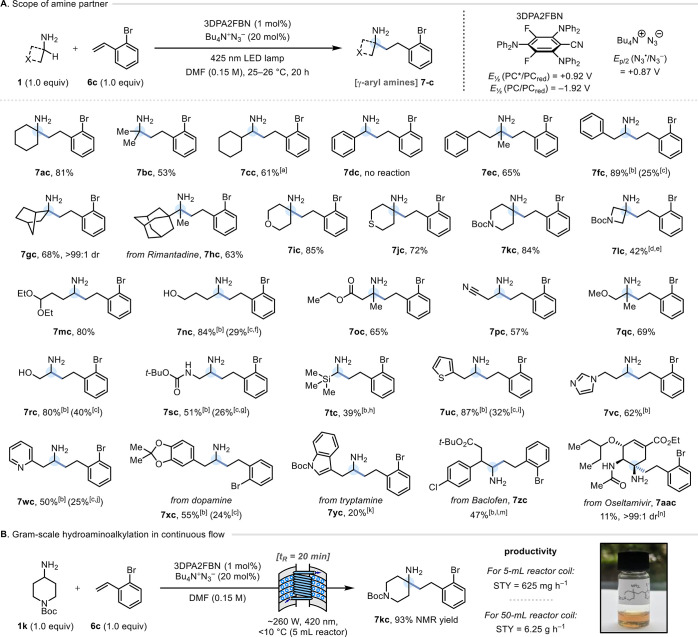
All reactions were carried out on a scale of 0.45 mmol. Isolated yields are reported. Notes: [a] 6% of inseparable, dialkylated product (wrt **1c**). [b] With 3.0 equiv of amine. [c] With 1.0 equiv of amine. [d] The mass balance comprised a mixture of unidentified byproducts but no detectable starting materials. [e] 44% of unreacted amine **1l**. [f] 46% of dialkylated product (wrt **1n**). [g] 41% of dialkylated product (wrt **1s**). [h] 54% of unreacted amine **1t** and 6% styrene **6c**. [i] 9% of dialkylated product (wrt **1u**). [j] 9% of dialkylated product (wrt **1u**). [k] Incomplete conversion to a complex mixture of products, which may include dialkylated material. [l] Isolated yield of Boc-protected **7zc** (61:39 dr) plus 11% of the lactam derived from thermal lactamization of **7zc** during workup. [m] 18% of dialkylated product (wrt **6c**). [n] Incomplete conversion to a complex mixture of products. Boc = *tert*-butoxycarbonyl.

With optimized conditions in hand, we next sought to determine the generality of the HAA reaction with respect to the alkylamine component **1** (Figure 3). 2-Bromostyrene **6c** was selected as the representative alkene partner, not because this confers the highest yields (i.e., electron-neutral styrenes **6a** or **6b** are superior), but because the bromine atom provides a useful synthetic handle for further elaboration (*vide infra*). The good performance of simple α,α-dialkylated amines such as cyclohexylamine **1a** and isopropylamine **1b** highlights a particular strength of this strategy relative to state-of-the-art metal-catalyzed HAAs: the insensitivity of the reaction to steric encumbrance at the α-position of the alkylamine. Indeed, this process is one of the few catalytic transformations on record that gives *direct* access to unprotected α-tertiary primary amines by C–C bond formation at the α-position.^9b,9d,28^ Pleasingly, the reaction also proved efficient with α-monosubstituted amine **1c**, with only 6% of α,α-dialkylation (with respect to **1c**). Some other α-monosubstituted amines gave more substantial α,α-dialkylation, but this issue was remedied by employing a 3-fold excess of the amine **1**. No reactivity with benzylamine **1d** was observed, and this suggests that the addition step to the C=C bond may be problematic, due to the higher thermodynamic stability of the α-amino radical.^29^ However, as evidenced by products **7ec** and **7fc**, the presence of benzylic C–H bonds on the alkylamine partner does not in itself pose a chemoselectivity issue, despite the fact that such C–H bonds are weaker than those α to the NH_2_ group (e.g., BDE = 85.4 ± 1.5 kcal mol^–1^ for PhC*H*_2_Me).^20^ Given that the N_3_^•^ radical is capable of hydrogen abstraction even from unactivated alkanes, the high selectivity here may arise from polarity-matching^19^ of the electrophilic azidyl radical with the more “hydridic” C–H bond α to the alkylamine. A diastereoselective reaction with *exo*-norbornylamine **1g** also proved possible, delivering product **7gc** as a single diastereomer, consistent with the proclivity of norbornyl radicals to be intercepted on the *exo* face. Steric encumbrance at the β-carbon of the alkylamine does not adversely affect the reaction, as evidenced by the successful HAA using Rimantadine **1h**—a marketed antiviral drug. The functional group compatibility of the reaction was next explored, including alkylamines bearing ether (**1i**,**q**), thioether (**1j**), carbamate (**1k**,**l**,**s**), acetal (**1m**), hydroxyl (**1n**,**r**), ester (**1o**), cyano (**1p**), and silyl (**1t**) groups. In all cases, the functionality was well accommodated and the selectivity for HAT α to the primary amine was very high,^30^ even in the presence of other weak and relatively “hydridic” C–H bonds, such as those α to free alcohols or acetals (i.e., **1m**,**n**,**r**). One of the most challenging amine substrates examined was 3-amino-*N*-Boc-azetidine **1l**, which gave the α-alkylated product **7lc** in 42% yield, returning 44% of unreacted amine **1l**. A strengthening of the α-C–H bond by virtue of the ring strain in **1l** is likely to be responsible for its lower reactivity.^9d^ A variety of heteroaromatic motifs were also tolerated, including thiophene (**1u**), imidazole (**1v**), and pyridine (**1w**) rings. Protected analogues of dopamine (**1x**), tryptamine (**1y**), and Baclofen (**1z**) were also successfully engaged in the HAA protocol. Even the complex antiviral drug Oseltamivir (**1aa**) could be α-C–H alkylated at the unprotected amino group, albeit in low yield.

### Scale Up in Continuous Flow

To demonstrate the scalability of the HAA process, we next performed a gram-scale reaction between 4-amino-*N*-Boc-piperidine **1k** and 2-bromostyrene **6c** in continuous flow.^31^ Using a Vapourtec R-series flow system equipped with a Uniqsis cold coil tubing module (5 mL) and a PhotoSyn HP LED photoreactor with a water-cooled 420 nm LED array (∼260 W radiant output power), a steady-state space-time yield (STY) of 625 mg h^–1^ for γ-arylamine **7kc** was obtained (Figure 3B). For a run time of 149 min, this delivered 1.55 g of isolated **7kc**, though a productivity of 6.25 g h^–1^ would be possible using the 50 mL reactor coil.

### Styrene Scope

**Figure 4:**
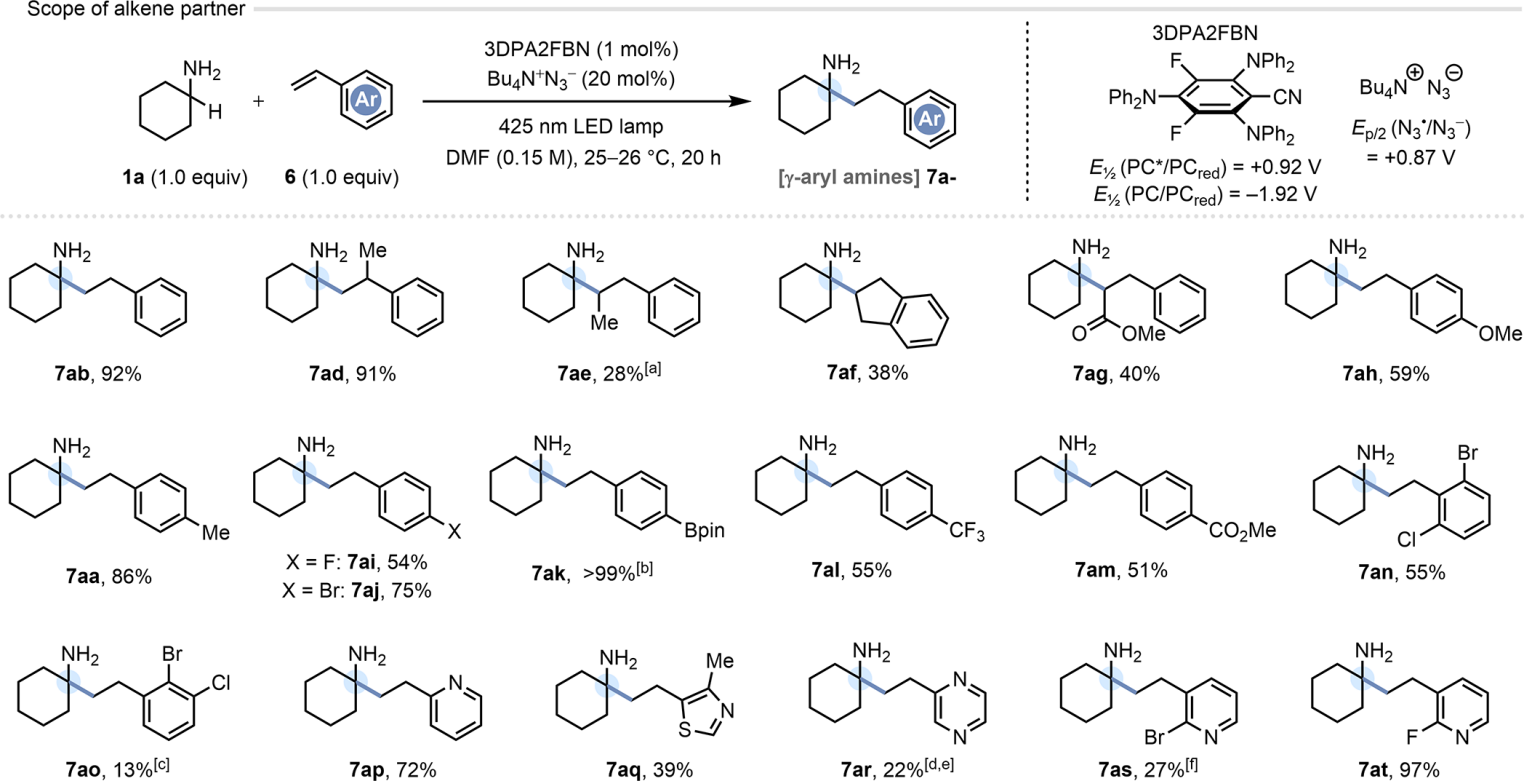
All reactions were carried out on a scale of 0.45 mmol. Isolated yields are reported. Notes: [a] Gave 40% NMR yield of **7ae** along with 24% unreacted **6e** and 6% of allylbenzene, plus other unidentified products. [b] Isolated as the phenol by oxidation the Bpin group with H_2_O_2_. [c] 22% of inseparable, debrominated product was also produced. [d] Yield given is for the *N*-Boc-protected derivative of **7ar**, which proved easier to isolate. [e] 9% of a 1:2 telomer and 43% (wrt **6r**) of reductive homocoupling product 1,4-di(pyrazin-2-yl)butane was also isolated. [f] The crude product mixture contained a 60:40 ratio of **7as** to its debrominated analogue.

The generality of the HAA protocol with respect to the styrene partner was next determined (Figure 4). Both styrene itself (**6b**) and α-methylstyrene (**6d**) returned γ-arylamines **7ab** and **7ad**, respectively, in yields exceeding 90%, although *trans*-β-methylstyrene (**6e**) gave incomplete conversion to **7ae** (i.e., 24% remaining **6e**), which was isolated in 28% yield. A similar issue was encountered with the *cis*-configured alkene indene (**6f**), which delivered **7af** in 38% yield. Notably, methyl cinnamate (**6g**) gave a HAA product derived from radical attack at the α-position of the cinnamate, contrary to the behavior of simple acrylates but congruent with other literature reports.^11b,11c,32^ Remarkably, the electron-rich acceptor *p*-methoxystyrene (**6h**) afforded the HAA product **7ah** in 59% yield,^33^ despite the pronounced polarity-mismatch of this reaction. Other electronically diverse *para*-substituents surveyed on the styrene partner included methyl (**6a**), fluoro (**6i**), bromo (**6j**), (pinacolato)boryl [pinB] (**6k**), trifluoromethyl (**6l**), and methyl ester (**6m**), with acceptable to excellent yields obtained in all cases. An electronic trend is difficult to identify, but it is clear that inclusion of strong +*M* (e.g., −OMe) or −*M* groups (e.g., −CF_3_) on the styrene partner does diminish the isolated yield. It should also be noted that a degree of styrene polymerization was suspected in some cases (i.e., insoluble precipitates formed when running earlier reactions in MeCN), and this may be operative to different extent with various styrenes. Although borylated product **7ak** was generated cleanly and quantitatively by ^1^H NMR, difficulties in purification led us to oxidize this compound with H_2_O_2_ and isolate the corresponding phenol (in >99% yield over two steps). Doubly halogenated styrenes **6n** and **6o** also participated, but the latter substrate also produced 22% of a debrominated HAA side-product, significantly compromising the yield of **7ao** (13%). This may arise from competitive attack of the electron-rich α-amino radical intermediate on the C–Br bond (activated by the adjacent chloro substituent) in an X atom transfer (XAT) step.^34^ Heteroaromatic styrene analogues were also assessed, bearing pyridyl (**6p**), thiazolyl (**6q**), and pyrazinyl (**6r**) motifs *in lieu* of a benzenoid ring. Although the pyridyl ring was well tolerated, and the thiazolyl ring to a lesser extent, the vinylpyrazine **6r** performed poorly, giving 22% of the HAA product **7ar**. Competitive telomerization (9% of a 1:2 adduct) and reductive homocoupling of **6r** (43% with respect to **6r**) were identified as side reactions in the latter case. Finally, the use of 2-bromovinylpyridine (**6s**) was attempted, to provide a functional handle for further elaboration (*vide infra*). However, competitive XAT at the C–Br bond was again problematic, and **7as** was obtained in 27% yield, alongside its debrominated analogue (∼1.5:1 ratio). Thankfully, this problem could be resolved by utilizing the 2-fluoro analogue **6t**, which delivered the γ-pyridylamine **7at** in 97% yield.

### Synthesis of Fingolimod

**Figure 5:**
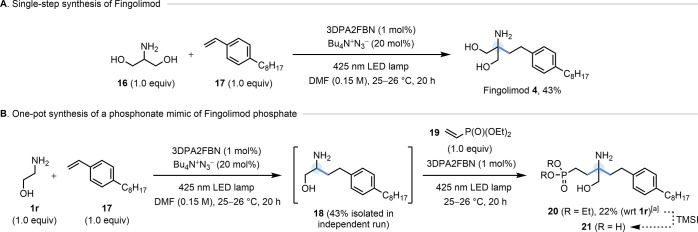
(A) Application to a protecting group-free synthesis of Fingolimod (**4**). (B) One-pot synthesis of a phosphonate mimic (**21**) of Fingolimod phosphate by tandem sequential α-C–H alkylation of ethanolamine (**1r**). Note: [a] 23% of the dialkylation product of **1r** with **17** was also isolated. TMS = trimethylsilyl.

To showcase the utility of our method, we next sought to apply our HAA protocol to the synthesis of a blockbuster drug. Fingolimod (**4**), developed by Novartis, is a S1P_1_ receptor agonist used to treat relapsing-remitting multiple sclerosis, with worldwide sales of $3 billion in 2020.^35^ It has also been recently identified as a promising lead for troponin-directed heart failure therapeutics.^36^ Several concise synthetic routes to Fingolimod **4** have been developed over the past two decades,^37^ but we reasoned that a HAA approach could raise the bar in terms of atom- and step-economy. Gratifyingly, the application of our optimized conditions to serinol **16** and 4-octylstyrene **17** (derived in 1 step from the commercial aldehyde) gave Fingolimod **4** in 43% isolated yield (Figure 5A). This is the shortest synthesis of Fingolimod on record, exhibiting 100% atom economy in the key step and with no recourse to any protecting groups. We anticipate that this operationally simple HAA procedure will find use in the synthesis of a diverse range of γ-arylamines as potential S1P_1_ receptor agonists.^38^

We were also drawn to the possibility of synthesizing α-tertiary amines by tandem sequential α-C–H dialkylation of an amine with two *different* radicophiles.^9c^ An obvious target to showcase this strategy was the phosphonic acid analogue **21** of Fingolimod phosphate (the active form of **4***in vivo*), which has been utilized as a nonhydrolyzable phosphate mimic in mechanism of action studies.^39^ Starting from ethanolamine **1r**, a photocatalytic α-C–H alkylation with 4-octylstyrene **17** followed by injection of vinyl phosphonate **19** into the reaction mixture and resubjection to irradiation gave α-tertiary amine **20** in 22% yield (over two steps, with respect to **1r**), in addition to 23% of the dialkylation product of **1r** with **17** (Figure 5B). A known phosphonate ester hydrolysis step would deliver target molecule **21** in only two synthetic operations. The previous synthetic route to **21** comprised nine steps from diethyl 2-aminomalonate,^39^ so the power of this new disconnection strategy for α-tertiary amines is clear.

### Synthesis of 1,2,3,4-Tetrahydroquinolines

**Figure 6:**
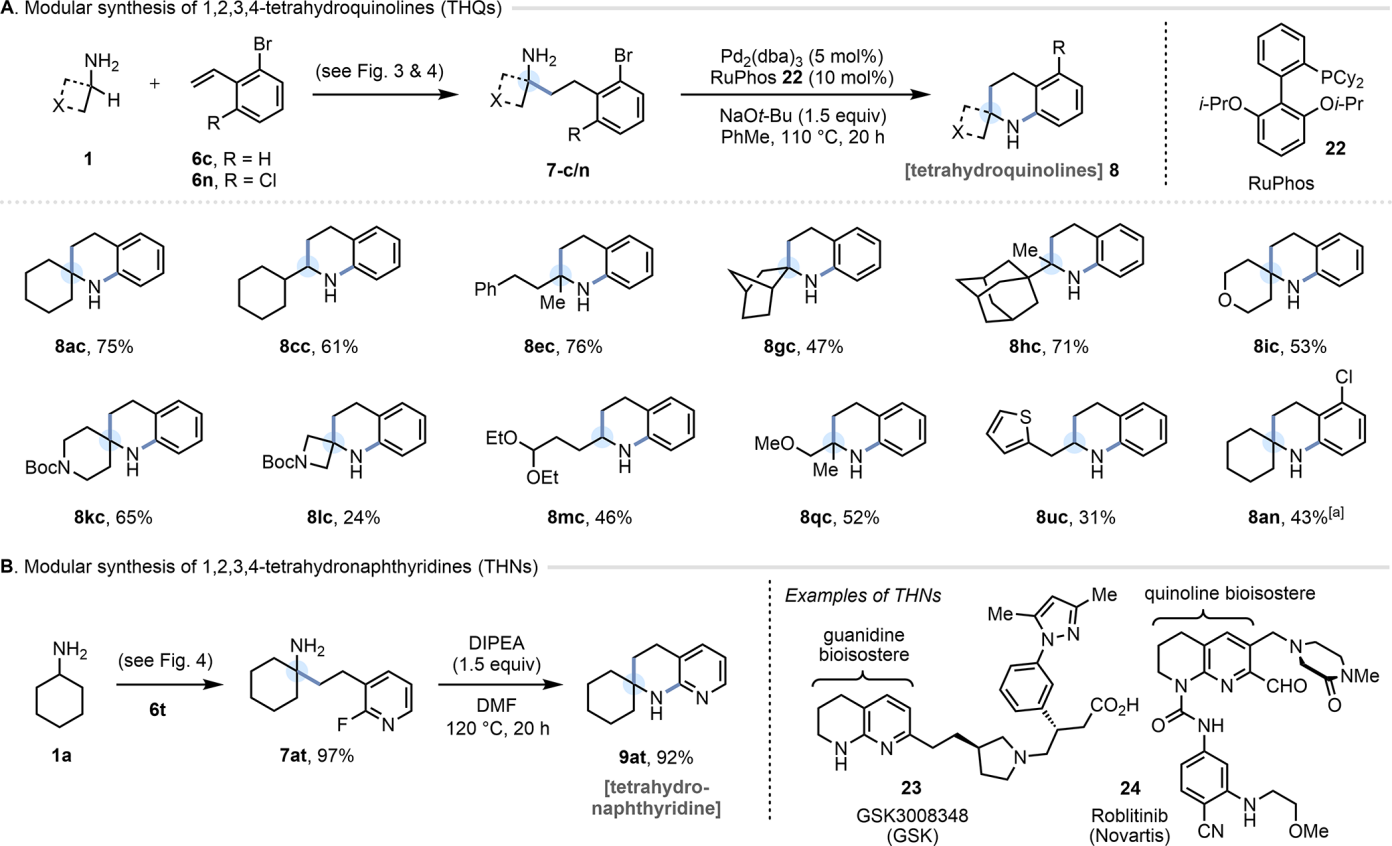
(A) Modular synthesis of 1,2,3,4-tetrahydroquinolines (THQs). In all cases except for **8an**, the remaining mass balance comprised unreacted starting material. Note: [a] Obtained as an inseparable mixture with **8ac** (14%), the proto-dechlorinated analogue of **8an**. (B) Modular synthesis of 1,2,3,4-tetrahydronaphthyridines (THNs).

Our HAA protocol can also serve as a key C–C bond-forming step for the synthesis of 1,2,3,4-tetrahydroquinolines (THQs) **8**.^40^ As partially saturated, benzo-fused *N*-heterocycles, THQs occupy a privileged position as core scaffolds in a host of natural and unnatural bioactives.^41^ Of the ∼43 000 known small-molecule THQs featuring alkylation α to nitrogen at C(2), only a third are α,α-dialkylated (almost exclusively α,α-dimethyl), and only ∼1% are spirocyclic at C(2).^42^ Given the explosion of interest in spirocycles in medicinal chemistry over the past two decades,^43^ the rarity of spirocyclic THQs is somewhat surprising. Thus, a modular strategy to access C(2)-(di)alkylated (including spirocyclic) THQs that is relatively insensitive to the electronics of the benzenoid component could greatly expand the accessible chemical space in this area. This is of particular relevance to fragment-based drug discovery,^44^ given that THQs exhibit multiple synthetically accessible growth vectors in three dimensions,^45^ and α-alkylated THQs have already been reported as fragment hits.^46^ By harnessing our HAA procedure to synthesize 2-bromo-substituted γ-arylamines **7-c/m** (see Figures 3 and 4), a palladium-catalyzed, intramolecular *N*-arylation allows for an expedient and modular assembly of (spirocyclic) THQs **8** (Figure 6A). Alternatively, in the case of 2-fluoropyridine substrate **7at**, a simple S_N_Ar reaction under basic conditions enabled access to a spirocyclic 1,2,3,4-tetrahydronaphthyridine (THN) scaffold **9at** (Figure 6B). THNs feature prominently as arginine mimics in αv integrin inhibitors (e.g., **23**),^47^ and the THN scaffold has also been deployed as a semi-saturated bioisostere of a quinoline, to enhance compound solubility (e.g., **24**).^48^

### Proposed Catalytic Cycle and Mechanistic Analysis

**Figure 7:**
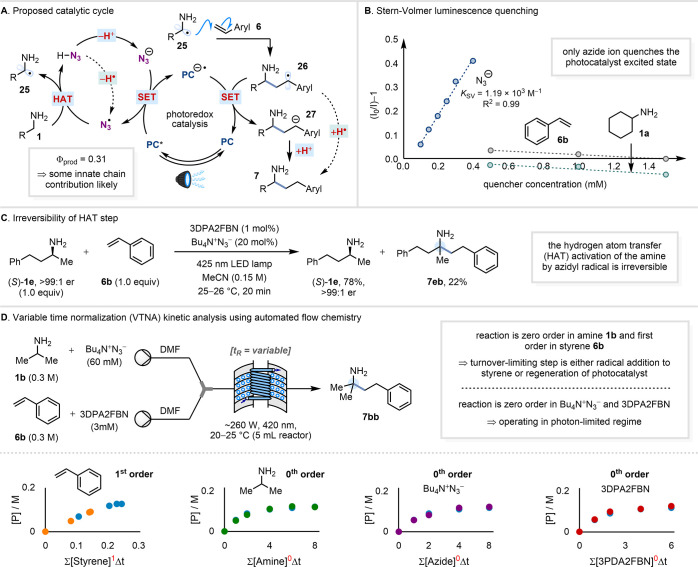
(A) Proposed catalytic cycle. (B) Stern–Volmer luminescence quenching. (C) Irreversibility of the HAT step. (D) Variable time normalization (VTNA) kinetic analysis using automated flow chemistry.

Our proposed catalytic cycle for the HAA process is outlined in Figure 7A. Initial oxidation of azide ion (*E*_p/2_^red^ = +0.87 V vs SCE in MeCN^9d^) by the photoexcited 3DPA2FBN [*E*_1/2_ (PC*/PC^–•^) = +0.92 V vs SCE^23^] generates the azidyl radical, N_3_^•^. This reductive quenching step is supported by Stern–Volmer luminescence quenching experiments (Figure 7B). Subsequent HAT from the relatively weak α-C–H bond of the primary alkylamine (BDE = 89–91 ± 2 kcal mol^–1^)^20^ occurs to give α-amino radical **25**,^49^ which undergoes addition to the styrene acceptor **6** to give a benzylic radical **26** [*E*_1/2_^red^ = −1.43 V vs SCE for ^•^CH_2_Ph/^–^CH_2_Ph in MeCN^21^]. Reduction of this radical to the corresponding carbanion **27** by the [3DPA2FBN]^−•^ radical anion [*E*_1/2_ (PC/PC^–•^) = −1.92 V vs SCE in MeCN] is presumably followed by proton transfer from HN_3_ (p*K*_a_ = 7.9 in DMSO)^50^ to give the γ-arylamine product **7** and regenerate the azide ion. Alternatively, a chain process involving HAT from HN_3_ (BDE = 93 kcal mol^–1^) to the benzylic radical **26** (BDE = 85.4 ± 1.5 kcal mol^–1^ for PhC*H*_2_Me)^20^ can be envisaged.^51^ To probe the latter possibility, the reaction quantum yield (Φ_prod_) was measured for the reaction of cyclohexylamine **1a** with styrene **6b** and found to be 0.31 (at 66% conversion to **7ab** by NMR).^52^ Given that quantum efficiencies for dual catalytic photoredox processes in which a cocatalyst is the quencher are typically very low (Φ_prod_ < 0.1),^9d,53^ a value of 0.31 is suggestive of at least some contribution from an innate chain (with a photonically inefficient initiation step). The operation of a photoredox process in parallel with an innate chain thus cannot be excluded.^52^ The reversibility of the HAT step between the alkylamine and N_3_^•^ was next investigated. Using enantiopure amine (*S*)-**1e**, the reaction with styrene **6b** was run to incomplete conversion (i.e., 78% of **1e** remaining) and the unreacted **1e** was recovered (Figure 7C). The enantiopurity of **1e** was found to have suffered no erosion during catalytic turnover (i.e., still >99:1 er), proving that formation of α-amino radical **25** is irreversible under the conditions. To gain further insight into the reaction mechanism, a variable time normalization analysis (VTNA) kinetic study was also conducted.^54^ The reaction of isopropylamine **1b** with styrene **6b** in DMF was run in continuous flow (see Supporting Information), using automated variation of residence times to construct the necessary concentration–time profiles (Figure 7D). The reaction displayed first order kinetics, with a first order dependence on styrene **6b** and a zero order dependence on amine **1b**, azide ion and photocatalyst (3DPA2FBN). This suggests that α-amino radical **25** addition to styrene **6** or, potentially, the photocatalyst regeneration step (PC^–•^ + **26** → PC + **27**) is turnover-limiting.^55,56^ A zero-order dependence on photocatalyst is consistent with the reaction operating in a “photon-limited” regime, where the rate is controlled by the light intensity and not by the photocatalyst concentration.^57^

## Conclusion

We have developed a metal-free, photoredox-catalyzed HAA of styrenes with unprotected primary alkylamines that provides direct access to γ-arylamines, including valuable α-tertiary derivatives. The protocol is executed under mild conditions, tolerates a wide variety of functional groups, and can be readily scaled in flow. We further illustrate the utility of this method in the shortest ever synthesis of the blockbuster drug Fingolimod, requiring no protecting groups. An iterative double α-C–H functionalization of the simple feedstock chemical ethanolamine is also showcased, to provide direct, one-pot access to a complex α-tertiary β-hydroxy amine (**20**) that previously required an eight-step synthesis. The application of this chemistry to the expedient synthesis of functionalized (and spirocyclic) 1,2,3,4-tetrahydroquinolines (THQs) and 1,2,3,4-tetrahydronaphthyridines (THNs) is also demonstrated, affording access to underexplored chemical space for drug discovery. Detailed mechanistic studies, including luminescence quenching and kinetic analyses, support a catalytic mechanism featuring reductive quenching of the organic photocatalyst by azide ion, to generate a highly reactive azidyl radical. This engages with the primary alkylamine in an irreversible HAT step to generate the key α-amino radical intermediate. The turnover-limiting step of the cycle is either radical addition to the styrene or regeneration of the photocatalyst, and a quantum yield measurement suggests some contribution from a radical chain process. In summary, we believe that the unique disconnection enabled by this new HAA protocol, together with its operational simplicity and sustainability, will help streamline the synthesis of complex alkylamines in both academia and industry.^58^

## Supporting Information Available

All experimental procedures and compound characterization (PDF)

## Accession Codes

CCDC 2093033 contains the supplementary crystallographic data for this paper. These data can be obtained free of charge via www.ccdc.cam.ac.uk/data_request/cif, or by emailing data_request@ccdc.cam.ac.uk, or by contacting The Cambridge Crystallographic Data Centre, 12 Union Road, Cambridge CB2 1EZ, UK; fax: +44 1223 336033.
