# Supplementary material for: Photocatalytic Hydroaminoalkylation of Styrenes with Unprotected Primary Alkylamines
Source: J Am Chem Soc. 2021 Sep 20;143(39):15936–45. doi: 10.1021/jacs.1c07401 (PMC8499025; doi:10.1021/jacs.1c07401)
Supplement: Supplementary file 1 — ja1c07401_si_001.pdf [file ja1c07401_si_001.pdf]

Photocatalytic Hydroaminoalkylation of Styrenes with Unprotected Primary Alkylamines

Hannah E. Askey,[a] James D. Grayson,[a] Joshua D. Tibbetts,[a] Jacob C. Turner-Dore,[a] Jake M. Holmes,[a] Gabriele Kociok-Kohn,[b] Gail L. Wrigley,[c] and Alexander J. Cresswell*[a]

[a] Department of Chemistry, University of Bath, Claverton Down, Bath, BA2 7AY, U.K., E-mail: a.j.cresswell@bath.ac.uk.

[b] Materials and Chemical Characterisation Facility (MC^2), University of Bath, Claverton Down, Bath, BA2 7AY, U.K.

[c] AstraZeneca, Oncology R&D, Research & Early Development, Darwin Building, 310, Cambridge Science Park, Milton Road, Cambridge, CB4 0WG, U.K.

TABLE OF CONTENTS

A. General Experimental *S2*

B. General Procedures *S4*

C. Preparation of Starting Materials *S6*

C.1. Literature Preparations *S6*

C.2. Preparation of Photocatalyst *S6*

C.3. Preparation of Amine Substrates *S7*

D. Hydroaminoalkylations of Styrenes with Primary Alkylamines *S8*

D.1. Reaction Generality: Amine Scope *S8*

D.2. Gram-Scale Hydroaminoalkylation in Continuous Flow *S44*

D.3. Reaction Generality: Styrene Scope *S45*

E. Application to Synthesis of Fingolimod and its Phosphonate Analogue *S81*

E.1. Synthesis of Fingolimod (**4**) *S81*

E.2. Synthesis of Phosphonate Mimic (**20**) of Fingolimod Phosphate *S83*

F. Application to Synthesis of THQs and THNs *S91*

F.1. Synthesis of THQs (**8**) *S91*

F.2. Synthesis of THN (**9at**) *S104*

G. Mechanistic Studies *S105*

G.1. Irreversibility of HAT Step *S105*

G.2. Variable Time Normalisation Analysis (VTNA) *S107*

G.3. Stern-Volmer Luminescence Quenching Analysis *S109*

G.4. Quantum Yield Measurement *S112*

H. ^{1}H and ^{13}C{^{1}H} NMR Spectra *S113*

I. References and Notes *S192*

A. General Experimental

General Setup: Procedures employing oxygen- and/or moisture-sensitive materials were performed with anhydrous solvents (*vide infra*) using standard inert atmosphere techniques (atmosphere of anhydrous nitrogen or argon). Room temperature (rt) typically ranged between 20–25 °C, depending on the time of day.

Photoreactors: All batch photoreactions were conducted in commercially-available EvoluChem PhotoRedOx Box reactors purchased from HepatoChem Inc. (100 Cummings Center, Suite 451C, Beverly, MA 01915 USA). An EvoluChem 18 W LED lamp (425 nm) was used for all reactions.

NMR Spectroscopy: ^{1}H, ^{13}C{^{1}H}, ^{19}F and ^{31}P spectra were recorded at 400/500 MHz, 101/126 MHz, 376 MHz, and 162 MHz respectively, using a Bruker Avance spectrometer (400 MHz) or an Agilent ProPulse spectrometer equipped with a OneNMR probe (500 MHz). Spectra were recorded at 298 K (25 °C) on the Bruker instrument and 298 K (25 °C) on the Agilent instrument, unless stated otherwise. ^{1}H and ^{13}C{^{1}H} NMR spectra were referenced to residual solvent peaks. Chemical shifts are reported in parts per million (ppm) relative to residual chloroform (δ = 7.26 ppm, ^{1}H; 77.16 ppm, ^{13}C), methanol (δ = 3.31 ppm, ^{1}H; 49.00 ppm, ^{13}C) or dimethylformamide (δ = 8.03 ppm, ^{1}H; 34.89 ppm, ^{13}C). All ^{13}C{^{1}H} resonances are assumed to be singlets, unless stated otherwise. Coupling constants, *J*, reported in Hertz (Hz), were calculated using *Mestrenova* 14 to the nearest 0.1 Hz. The following abbreviations (and their combinations) are used to label the multiplicities: br (broad), s (singlet), d (doublet), t (triplet), q (quartet), sept (septet), and m (multiplet). ^{1}H and ^{13}C{^{1}H} assignments for novel compounds are corroborated through 2D (COSY, HSQC, HMBC).

Infrared Spectroscopy: Infrared (IR) spectra of neat compounds were recorded over the range 4000–650 cm^{-1} using a PerkinElmer Spectrum 100 ATR-FTIR spectrometer. Peaks are reported in cm^{-1} with indicated relative intensities: s (strong, 0–33% T); m (medium, 34–66% T), w (weak, 67–100% T), and br (broad).

Mass Spectrometry: Electrospray ionisation (ESI^{+}) spectra were recorded on an Agilent Electrospray Quadrupole Time-of-Flight mass spectrometer (ESI-QTOF). Data are reported in the form of *m*/*z* (intensity relative to the base peak = 100).

Melting Points: Uncorrected melting points (mp) were determined on a Stanford Research Systems OptiMelt automated capillary melting point apparatus in open capillary tubes.

Single Crystal X-Ray Diffraction Analysis: Intensity data was collected at 150(2) K on a Rigaku Xcalibur, EosS2 single crystal diffractometer using graphite monochromated Mo-Kα radiation (λ = 0.71073 Å). Unit cell determination, data collection, data reduction and empirical absorption correction were performed using the CrysAlisPro software version 1.171.41.93a (Rigaku OD, 2020). The structures were solved with SHELXT and refined by a full-matrix least-squares procedure based on F2 (SHELXL-2018/3) (Sheldrick, G. M. *Acta Cryst.* **2015**, *C71*, 3–8). All non-hydrogen atoms were refined anisotropically. Hydrogen atoms were placed onto calculated positions and refined using a riding model.

Chromatography: Analytical thin-layer chromatography was performed on Merck silica gel 60 F_{254} aluminium-backed plates. Visualisation was accomplished with UV light (254 nm), iodine (I_2) on silica, aqueous basic potassium permanganate ($KMnO_4$), and/or phosphomolybdic Acid (PMA) solutions. Automated flash column chromatography (normal and reversed phase) was performed using a CombiFlash NextGen 300+ System equipped with UV and ELSD detectors. Manual flash column chromatography was performed using high-purity grade silica gel, pore size 60 Å, 200-400 mesh particle size (Sigma-Aldrich, Cat. No. 288594).

Solvents: Reaction solvents tetrahydrofuran (THF) and acetonitrile (MeCN) were dried by percolation through columns packed with neutral alumina under a positive pressure of nitrogen. Toluene (PhMe) was distilled over CaH_2 and stored under nitrogen over molecular sieves. Dimethylformamide (DMF) (extra dry, with molecular sieves), deuterated dimethylformamide (DMF-d_7), dichloromethane (CH_2Cl_2) and deuterated chloroform ($CDCl_3$) were used as received. Solvents for filtration, transfers, chromatography, and recrystallisation, including acetonitrile (MeCN), chloroform ($CHCl_3$), dichloromethane (CH_2Cl_2), diethyl ether (Et_2O), ethyl acetate (EtOAc), hexane, methanol (MeOH), and 40–60° petroleum ether (petrol) were used as received.

Chemicals: Chemicals purchased from commercial suppliers were used as received. Commercial styrenes contained stabilizers (e.g., 4-*tert*-butylcatechol), but these did not adversely affect the hydroaminoalkylation reaction.

B. General Procedures

General Procedure 1 for Hydroaminoalkylation with Primary Alkylamines

A 20-mL scintillation vial equipped with a stirrer bar was transferred to a nitrogen-filled purge box. In the case of solid or viscous oil substrates, the requisite amine **1** (1.0 equiv or 3.0 equiv) or styrene **6** (1.0 equiv) was weighed into the empty vial at this point, and the stirrer bar was replaced. The vial was then charged with stock solutions of 3DPA2FBN (2.80 mM in DMF, 1 mol%), tetrabutylammonium azide (70.3 mM in DMF, 20 mol%) and additional anhydrous DMF was added to give a total concentration of 0.15 M wrt the styrene **6**. For liquid amines, the requisite amine **1** (1.0 equiv or 3.0 equiv) was transferred into the vial by microlitre syringe. For liquid styrenes, the requisite styrene **6** (1.0 equiv) was added into the vial by microlitre syringe and the vial was sealed using a B24 rubber septa. It was then removed from the purge box and transferred to a photoreactor, and irradiated (with stirring) for 20 h at 425 nm. Fan cooling was used to maintain an external temperature of 25–26 °C. Following irradiation, the reaction mixture was concentrated *in vacuo* on a spiral evaporator.

Notes on tetrabutylammonium azide (Bu_4NN_3): Bu_4NN_3 (CAS# 993-22-6) is extremely hygroscopic and best handled under inert atmosphere. It can be conveniently prepared *in situ* by stirring Bu_4NCl with NaN_3 in MeCN, followed by filtration of NaCl. We found that the commercial material (Sigma-Aldrich) is contaminated with NaCl, such that dissolution in MeCN is visibly incomplete, leading to a slightly cloudy solution. Upon standing, the NaCl residues will settle and the clear supernatant solution can be used. Whilst Bu_4NN_3 should be treated with the same precautions taken for sodium azide (NaN_3) (e.g., not ingested/inhaled, and exposure to acids or low pH avoided), it has no impact sensitivity and a decomposition temperature of 196 °C (see: Shalibor, A.; Modarresi-Alam, A. R. A Green and Simple Process for Preparation of Tetraalkylammonium Azide with Excellent Environmental Factor: Comparison of Batch and Flow Column Reactor. *Org. Proc. Res. Dev.* **2018**, *22*, 1753–1760).

General Procedure 2 for the Synthesis of THQs

A microwave vial equipped with a stirrer bar was transferred to a nitrogen-filled purge box and charged with NaO*t*-Bu (1.5 equiv). The vial was then charged with solutions of $Pd_2(dba)_2$ (0.02 mM in PhMe, 5 mol%), RuPhos (0.02 mM in PhMe, 10 mol%), and the prerequisite γ-aryl amine (1.0 equiv). Additional anhydrous PhMe was added to a total concentration of 0.1 M (wrt substrate). The vial was then capped with an aluminium crimp seal and removed from the

purge box, then the mixture was heated to 110 °C for 20 h. After allowing the mixture to cool to rt, the mixture was eluted through Celite and concentrated *in vacuo* on a spiral evaporator.

C. Preparation of Starting Materials

C.1. Literature Preparations

2,4,5,6-Tetra(9*H*-carbazol-9-yl)isophthalonitrile (4CzIPN),[1] styrenes **6i**,[2] **6j**[3], **6m**,[2] **6n**,[2] **6r**,[2] **6s**,[2] and protected amines (**1x**,[4] **1y**[5,6,7]) were prepared according to literature procedures.

C.2. Preparation of Photocatalyst

Preparation of 2,4,6-tris(diphenylamino)-3,5-difluorobenzonitrile (3DPA2FBN)

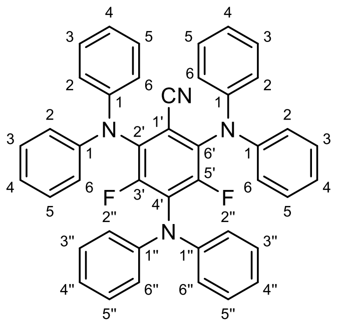

3DPA2FBN

To a Schlenk flask under an argon atmosphere, diphenylamine (5.29 g, 31.3 mmol, 6.25 equiv), anhydrous THF (100 mL, 0.05 M) and NaH (60% in oil) (1.87 g, 47 mmol, 9.4 equiv) were added and the mixture was heated to 50 °C for 30 min. Pentafluorobenzonitrile (965 mg, 5.0 mmol, 1.0 equiv) was added and the mixture was stirred at rt for 48 h. H_2O (50 mL) was added dropwise and the mixture was concentrated *in vacuo*. The mixture was extracted with CH_2Cl_2 (3 × 30 mL), and the combined organic layers were dried (Na_2SO_4), filtered, and concentrated *in vacuo*. Purification *via* recrystallisation from EtOAc gave 3DPA2FBN as a yellow solid (2.62 g, 82%). The NMR spectroscopic data was in accordance with the literature.[8]

<u>Data for 2,4,6-tris(diphenylamino)-3,5-difluorobenzonitrile (3DPA2FBN):</u>

<u>^{1}H NMR:</u> (400 MHz, $CDCl_3$)
7.27–7.23 (m, 16H), 7.08–6.92 (m, 24H)

<u>^{13}C NMR:</u> (126 MHz, $CDCl_3$)
155.1 (dd, *J* = 259, 5.5 Hz, *C*(3′), *C*(5′)), 145.9 (*C*(1)), 145.6 (*C*(1′)), 135.3 (dd, *J* = 12.4, 4.8 Hz, *C*(2′), *C*(6′)), 130.3 (t, *J* = 12.6 Hz, *C*(4′)), 129.5 (*C*(3), *C*(5), *C*(3″), *C*(5″)), 124.0 (*C*(4″)), 123.5 (*C*(4)), 121.8 (*C*(2), *C*(6)), 121.8 (*C*(2″), *C*(6″)), 113.3 (t, *J* = 4.0 Hz, *C*(1′)), 111.2 (t, *J* = 4.0 Hz, *C*N)

<u>^{19}F NMR:</u> (376 MHz, $CDCl_3$)
–120.2

C.3. Preparation of Amine Substrates

Preparation of *tert*-butyl (*RS*)-4-amino-3-(4-chlorophenyl)butanoate (1z)

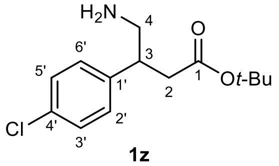

1z

To a stirred suspension of (±)-baclofen (1.60 g, 7.50 mmol, 1.0 equiv) in *tert*-butyl acetate (20 mL) was added 70% aqueous perchloric acid (1.05 mL, 12 mmol, 1.6 equiv) dropwise over 5 min. The resulting solution was stoppered and stirred at rt for 16 h. H_2O (20 mL) was then added and the layers were separated. The organic layer was extracted with 2 M HCl (2 × 10 mL) and the combined aqueous layers were then neutralised with solid K_2CO_3, before being extracted with Et_2O (3 × 30 mL). The combined organic layers were dried (Na_2SO_4) and concentrated *in vacuo* to give **1z** as a colourless solid (0.83 g, 41%).

Data for **1z**:

mp: 64–66 °C (Et_2O)

^{1}H NMR: (400 MHz, $CDCl_3$)

7.29 (d, *J* = 8.4 Hz, 2H, C(2′)*H*, C(6′)*H*), 7.15 (d, *J* = 8.4 Hz, 2H, C(3′)*H*, C(5′)*H*), 3..08 (dddd, 1H, *J* = 8.5, 8.3, 6.8, 5.6 Hz C(3)*H*), 2.92 (dd, *J* = 12.8, 5.6 Hz, 1H, C(4)H_A), 2.84 (dd, *J* = 12.8, 8.3 Hz, 1H, C(4)H_B), 2.61 (dd, *J* = 15.1, 6.8 Hz, 1H, C(2)H_A), 2.45 (dd, *J* = 15.1, 8.5 Hz, 1H, C(2)H_B), 1.32 (s, 9H, OC(CH_3)$_3$), 1.26 (br s, 2H, NH_2)

^{13}C NMR: (101 MHz, $CDCl_3$)

171.4 (*C*(1)), 140.8 (*C*(1′)), 132.6 (*C*(4′)), 129.4 (*C*(5′), *C*(3′)), 128.8 (*C*(2′), *C*(6′)), 80.8 (O*C*(CH$_3$)$_3$), 47.7 (*C*(4)), 45.6 (*C*(3)), 40.0 (*C*(2)), 28.1 (OC(*C*H$_3$)$_3$)

IR: (neat)

3840 (w), 2978 (w), 2931 (w), 2482 (w), 2169 (w), 2153 (w), 2007 (w), 1976 (w), 1720 (m), 1627 (w), 1559 (w), 1491 (w), 1459 (w), 1416 (w), 1366 (w), 1292 (w), 1242 (w), 1147 (m), 1106 (w), 1090 (w), 1014 (w), 949 (w), 825 (m), 763 (w), 719 (w), 678 (w)

MS: (ESI$^+$)

214 (36%), 216 (12%), 270 ([M+H]$^+$, 100%), 271 (16%), 272 (33%)

HRMS: (ESI$^+$)

Calcd for $C_{14}H_{21}ClNO_2$: 270.1255, found: 270.1262

D. Hydroaminoalkylations of Styrenes with Primary Alkylamines

D.1. Reaction Generality: Amine Scope

Preparation of 1-(2-bromophenethyl)cyclohexan-1-amine (7ac)

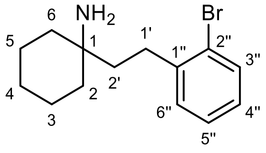

7ac

Following ***General Procedure 1***, cyclohexylamine (**1a**) (52 µL, 45 mg, 0.45 mmol, 1.0 equiv), 2-bromostyrene (**6c**) (57 µL, 83 mg, 0.45 mmol, 1.0 equiv), 3DPA2FBN (2.80 mM in DMF, 1.60 mL, 4.5 µmol, 1 mol%), tetrabutylammonium azide (70.3 mM in DMF, 1.28 mL, 90 µmol, 20 mol%) and DMF (120 µL) were reacted for 20 h with a 425 nm LED lamp. The mixture was concentrated *in vacuo* and purified *via* flash column chromatography on silica gel (12 g) in CH_2Cl_2 (5 CV) then 100:0:0→95:4.5:0.5 CH_2Cl_2–MeOH–aq. NH_4OH (over 20 CV) then 95:4.5:0.5 CH_2Cl_2–MeOH–aq. NH_4OH (5 CV) to give **7ac** as a yellow oil (103 mg, 81%).

Data for **7ac**:

^{1}H NMR: (500 MHz, $CDCl_3$)
7.55–7.47 (m, 1H, C(3″)*H*), 7.24–7.20 (m, 2H, C(4″)*H*, C(6″)*H*), 7.08–7.00 (m, 1H, C(5″)*H*), 2.80–2.76 (m, 2H, C(1′)H_2), 1.64–1.60 (m, 2H, C(2′)H_2), 1.58–1.48 (m, 7H, C(2)H_A, C(3)H_2, C(4)H_A, C(5)H_2, C(6)H_A), 1.44–1.35 (m, 3H, C(2)H_B, C(4)H_B, C(6)H_B), 1.25 (br s, 2H, NH_2)

^{13}C NMR: (101 MHz, $CDCl_3$)
142.5 (*C*(1″)), 132.9 (*C*(3″)), 130.5 (*C*(6″)), 127.7 (*C*(5″)), 127.5 (*C*(4″)), 124.5 (*C*(2″)), 50.8 (*C*(1)), 43.0 (*C*(2′)), 38.9 (*C*(2), *C*(6)), 30.3 (*C*(1′)), 26.2 (*C*(4)), 22.4 (*C*(3), *C*(5))

IR: (neat)
2923 (m), 2851 (w), 1593 (w), 1567 (w), 1471 (w), 1450 (w), 1439 (w), 1264 (w), 1188 (w), 1130 (w), 1044 (w), 1023 (m), 978 (w), 930 (w), 906 (w), 845 (w), 816 (w), 750 (m), 699 (w), 659 (w)

MS: (ESI^+)
285 (15%), 284 ($[M(^{81}Br)+H]^+$, 99%), 283 (15%), 282 ($[M(^{79}Br)+H]^+$, 100%), 204 (11%)

HRMS: (ESI$^+$)

Calcd for $C_{14}H_{21}(^{79}Br)N$: 282.0857, found: 282.0854

Preparation of 4-(2-bromophenyl)-2-methylbutan-2-amine (7bc)

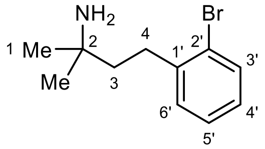

7bc

Following ***General Procedure 1***, isopropylamine (**1b**) (39 µL, 27 mg, 0.45 mmol, 1.0 equiv), 2-bromostyrene (**6c**) (57 µL, 83 mg, 0.45 mmol, 1.0 equiv), 3DPA2FBN (2.80 mM in DMF, 1.60 mL, 4.5 µmol, 1 mol%), tetrabutylammonium azide (70.3 mM in DMF, 1.28 mL, 90 µmol, 20 mol%) and DMF (120 µL) were reacted for 20 h with a 425 nm LED lamp. The mixture was concentrated *in vacuo* and purified *via* flash column chromatography on silica gel (12 g) in CH_2Cl_2 (5 CV) then 100:0:0→95:4.5:0.5 CH_2Cl_2–MeOH–aq. NH_4OH (over 20 CV) then 95:4.5:0.5 CH_2Cl_2–MeOH–aq. NH_4OH (5 CV) to give **7bc** as a yellow oil (57 mg, 53%).

Data for **7bc**:

^{1}H NMR: (400 MHz, $CDCl_3$)

7.54–7.50 (m, 1H, C(3′)*H*), 7.24–7.20 (m, 2H, C(4′)*H*, C(6′)*H*), 7.07–7.02 (m, 1H, C(5′)*H*), 2.82–2.73 (m, 2H, C(4)H_2), 1.66–1.61 (m, 2H, C(3)H_2), 1.55 (br s, 2H, NH_2), 1.20 (s, 6H, C(1)H_3, C(2)*Me*)

^{13}C NMR: (126 MHz, $CDCl_3$)

142.1 (*C*(1′)), 133.0 (*C*(3′)), 130.4 (*C*(6′)), 127.7 (*C*(5′)), 127.6 (*C*(4′)), 124.5 (*C*(2′)), 49.8 (*C*(2)), 45.5 (*C*(3)), 31.8 (*C*(4)), 30.4 (*C*(1), C(2)*Me*)

IR: (neat)

3057 (w), 2957 (w), 2925 (w), 2864 (w), 1567 (w), 1471 (w), 1439 (w), 1383 (w), 1365 (w), 1281 (w), 1223 (w), 1179 (w), 1109 (w), 1045 (w), 1024 (m), 942 (w), 853 (w), 826 (w), 746 (m), 659 (w)

MS: (ESI$^+$)

282 (14%), 245 (11%), 244 ([M(^{81}Br)+H]$^+$, 96%), 243 (12%), 242 ([M(^{79}Br)+H]$^+$, 100%)

HRMS: (ESI$^+$)

Calcd for $C_{11}H_{17}(^{79}Br)N$: 242.0544, found: 242.0542

Preparation of (*RS*)-3-(2-bromophenyl)-1-cyclohexylpropan-1-amine (7cc) and 1,5-bis(2-bromophenyl)-3-cyclohexylpentan-3-amine (28)

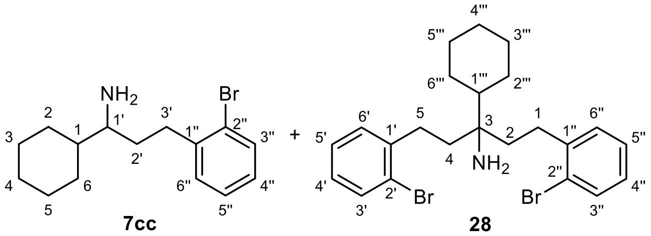

Following ***General Procedure 1***, cyclohexanemethylamine (**1c**) (59 µL, 51 mg, 0.45 mmol, 1.0 equiv), 2-bromostyrene (**6c**) (57 µL, 83 mg, 0.45 mmol, 1.0 equiv), 3DPA2FBN (2.80 mM in DMF, 1.60 mL, 4.5 µmol, 1 mol%), tetrabutylammonium azide (70.3 mM in DMF, 1.28 mL, 90 µmol, 20 mol%) and DMF (120 µL) were reacted for 20 h with a 425 nm LED lamp. The mixture was concentrated *in vacuo* and purified *via* flash column chromatography on silica gel (12 g) in CH_2Cl_2 (5 CV) then 100:0:0→95:4.5:0.5 CH_2Cl_2–MeOH–aq. NH_4OH (over 20 CV) then 95:4.5:0.5 CH_2Cl_2–MeOH–aq. NH_4OH (5 CV) to give an inseparable 91:9 mixture of **7cc** to **28** as a yellow oil [94.3 mg, comprising 81.2 mg of **7cc** (61%) and 13.1 mg of **28** (6% wrt **6c**)].

Data for **7cc**:

^{1}H NMR: (400 MHz, $CDCl_3$)
7.57–7.47 (m, 1H, C(3″)*H*), 7.26–7.19 (m, 2H, C(4″)*H*, C(6″)*H*), 7.08–7.02 (m, 1H, C(5″)*H*), 2.95–2.69 (m, 2H, C(3′)H_2), 2.64–2.51 (m, 1H, C(1′)*H*), 1.85–1.65 (m, 6H, C(2)H_A, C(3)H_A, C(4)H_A, C(5)H_A, C(6)H_A, C(2′)H_A), 1.56–1.44 (m, 1H, C(2′)H_B), 1.31–0.96 (m, 8H, C(1)*H*, C(2)H_B, C(3)H_B, C(4)H_B, C(5)H_B, C(6)H_B, NH_2)

^{13}C NMR: (101 MHz, $CDCl_3$)
142.1 (*C*(1″)), 132.9 (*C*(3″)), 130.5 (*C*(6″)), 127.6 (*C*(5″)), 127.6 (*C*(4″)), 124.5 (*C*(2″)), 56.1 (*C*(1′)), 44.1 (*C*(1)), 35.4 (*C*(2′)), 33.6 (*C*(3′)), 29.8 (*C*(2) or *C*(6)), 28.0 (*C*(2) or *C*(6)), 26.8 (*C*(4)), 26.7 (*C*(3) or *C*(5)), 26.6 (*C*(3) or *C*(5))

HRMS: (ESI^+)
Calcd for $C_{15}H_{23}(^{79}Br)N$: 296.1014, found: 296.1013

Data for **28**:

^{1}H NMR: (400 MHz, $CDCl_3$)

7.57–7.47 (m, 2H, C(3′)*H*, C(3″)*H*), 7.26–7.19 (m, 4H, C(4′)*H*, C(6′)*H*, C(4″)*H*, C(6″)*H*), 7.08–7.02 (m, 2H, C(5′)*H*, C(5″)*H*), 2.95–2.69 (m, 4H, C(1)H_2, C(5)H_2), 1.85–1.65 (m, 7H, C(2‴)H_A, C(3‴)H_A, C(4‴)H_A, C(5‴)H_A, C(6‴)H_A, C(2)H_A, C(4)H_A), 1.56–1.44 (m, 2H, C(2)H_B, C(4)H_B), 1.31–0.96 (m, 8H, C(1‴)*H*, C(2‴)H_B, C(3‴)H_B, C(4‴)H_B, C(5‴)H_B, C(6‴)H_B, NH_2)

^{13}C NMR: (101 MHz, $CDCl_3$)

142.5 (*C*(1′), *C*(1″)), 133.0 (*C*(3′), *C*(3″)), 130.6 (*C*(6′), *C*(6″)), 127.8 (*C*(5′), *C*(5″)), 127.7 (*C*(4′), *C*(4″)), 124.5 (*C*(2′), *C*(2″)), 55.6 (*C*(3)), 45.6 (*C*(1‴)), 38.0 (*C*(2), *C*(4)), 30.7 (*C*(1), *C*(5)), 27.1 (*C*(2‴) or *C*(6‴)), 26.9 (*C*(2‴) or *C*(6‴)), 26.8 (*C*(4‴)), 26.7 (*C*(3‴) or *C*(5‴)), 26.6 (*C*(3‴) or *C*(5‴))

HRMS: (ESI^+)

Calcd for $C_{23}H_{30}(^{79}Br)(^{81}Br)N$: 480.0725, found: 480.0724

Preparation of (*RS*)-1-(2-bromophenyl)-3-methyl-5-phenylpentan-3-amine (7ec)

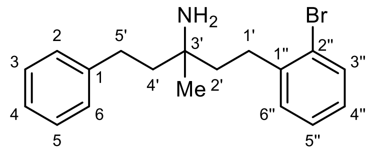

7ec

Following ***General Procedure 1***, 4-phenylbutan-2-amine (**1e**) (72 μL, 67 mg, 0.45 mmol, 1.0 equiv), 2-bromostyrene (**6c**) (57 μL, 83 mg, 0.45 mmol, 1.0 equiv), 3DPA2FBN (2.80 mM in DMF, 1.60 mL, 4.5 μmol, 1 mol%), tetrabutylammonium azide (70.3 mM in DMF, 1.28 mL, 90 μmol, 20 mol%) and DMF (120 μL) were reacted for 20 h with a 425 nm LED lamp. The mixture was concentrated *in vacuo* and purified *via* flash column chromatography on silica gel (12 g) in CH_2Cl_2 (5 CV) then 100:0:0→95:4.5:0.5 CH_2Cl_2–MeOH–aq. NH_4OH (over 20 CV) then 95:4.5:0.5 CH_2Cl_2–MeOH–aq. NH_4OH (5 CV) to give **7ec** as a colourless oil (96.9 mg, 65%).

Data for **7ec**:

^{1}H NMR: (400 MHz, $CDCl_3$)

7.58–7.48 (m, 1H, C(3″)*H*), 7.33–7.27 (m, 2H, C(2)*H*, C(6)*H*), 7.26–7.14 (m, 5H, C(3)*H*, C(4)*H*, C(5)*H*, C(4″)*H*, C(6″)*H*), 7.09–7.03 (m, 1H, C(5″)*H*), 2.84–

2.76 (m, 2H, C(1′)*H*2), 2.74–2.67 (m, 2H, C(5′)*H*2), 1.79–1.66 (m, 4H, C(2′)*H*2, C(4′)*H*2), 1.37 (br s, 2H, N*H*2), 1.23 (s, 3H, C(3′)*Me*)

^{13}C NMR: (101 MHz, $CDCl_3$)

142.9 (*C*(1)), 142.1 (*C*(1″)), 133.0 (*C*(3″)), 130.5 (*C*(6″)), 128.6 (*C*(3), *C*(5)), 128.5 (*C*(2), *C*(6)), 127.8 (*C*(5″)), 127.7 (*C*(4″)), 125.9 (*C*(4)), 124.5 (*C*(2″)), 51.9 (*C*(3′)), 45.0 (*C*(2′)), 43.3 (*C*(2′)), 31.4 (*C*(1′)), 30.8 (*C*(5′)), 28.2 (*C*(Me))

IR: (neat)

3060 (w), 3025 (w), 2930 (w), 2863 (w), 1602 (w), 1567 (w), 1495 (w), 1471 (w), 1454 (w), 1438 (w), 1376 (w), 1220 (w), 1045 (w), 1023 (m), 820 (w), 747 (m), 715 (w), 698 (m), 659 (w)

MS: (ESI^+)

516 (11%), 482 (11%), 480 (23%), 478 (11%), 335 (19%), 334 ($[M(^{81}Br)+H]^+$, 94%), 333 (20%), 332 ($[M(^{79}Br)+H]^+$, 100%), 282 (16%)

HRMS: (ESI^+)

Calcd for $C_{18}H_{23}(^{79}Br)N$: 332.1014, found: 332.1011

Preparation of (*RS*)-4-(2-bromophenyl)-1-phenylbutan-2-amine (7fc)

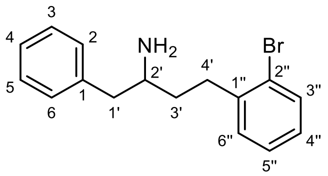

7fc

Following ***General Procedure 1***, 2-phenethylamine (**1f**) (170 μL, 164 mg, 1.35 mmol, 3.0 equiv), 2-bromostyrene (**6c**) (57 μL, 83 mg, 0.45 mmol, 1.0 equiv), 3DPA2FBN (2.80 mM in DMF, 1.60 mL, 4.5 μmol, 1 mol%), tetrabutylammonium azide (70.3 mM in DMF, 1.28 mL, 90 μmol, 20 mol%) and DMF (120 μL) were reacted for 20 h with a 425 nm LED lamp. The mixture was concentrated *in vacuo* and purified *via* flash column chromatography on silica gel (12 g) in CH_2Cl_2 (5 CV) then 100:0:0→95:4.5:0.5 CH_2Cl_2–MeOH–aq. NH_4OH (over 20 CV) then 95:4.5:0.5 CH_2Cl_2–MeOH–aq. NH_4OH (5 CV), followed by reversed-phase flash column chromatography on C_{18} silica gel (15.5 g) in 98:2 H_2O–MeOH (5 CV) then 98:2→0:100 H_2O–MeOH (over 15 CV) then MeOH (5 CV) to give **7fc** as a yellow oil (121 mg, 89%).

Data for **7fc**:

^{1}H NMR: (400 MHz, $CDCl_3$)

7.59–7.49 (m, 1H, C(3″)H_2), 7.34–7.28 (m, 2H, C(2)*H*, C(6)*H*), 7.26–7.17 (m, 5H, C(3)*H*, C(4)*H*, C(5)*H*, C(4″)*H*, C(6″)*H*), 7.09–7.02 (m, 1H, C(5″)*H*), 3.11–3.04 (m, 1H, C(2′)*H*), 2.97–2.78 (m, 3H, C(1′)H_A, C(4′)H_2), 2.57–2.49 (m, 1H, C(1′)H_B), 1.87–1.79 (m, 1H, C(3′)H_A), 1.71–1.61 (m, 1H, C(3′)H_B), 1.17 (br s, 2H, NH_2)

^{13}C NMR: (101 MHz, $CDCl_3$)

141.6 (*C*(1″)), 139.5 (*C*(1)), 132.9(*C*(3″)), 130.4 (*C*(6″)), 129.4(*C*(3), *C*(5)), 128.6 (*C*(2), *C*(6)), 127.7 (*C*(5″)), 127.6 (*C*(4″)), 126.4 (*C*(4)), 124.5 (*C*(2″)), 52.6 (*C*(2′)), 44.8 (*C*(1′)), 38.1 (*C*(3′)), 33.1 (*C*(4′))

IR: (neat)

3060 (w), 3026 (w), 2920 (w), 2851 (w), 1672 (w), 1601 (w), 1567 (w), 1494 (w), 1470 (w), 1453 (w), 1438 (w), 1381 (w), 1113 (w), 1077 (w), 1044 (w), 1022 (w), 909 (w), 833 (w), 746 (m), 700 (m), 659 (w)

MS: (ESI^+)

307 (17%), 306 ($[M(^{81}Br)+H]^+$, 97%), 305 (18%), 304 ($[M(^{79}Br)+H]^+$, 100%)

HRMS: (ESI^+)

Calcd for $C_{16}H_{19}(^{79}Br)N$: 304.0701, found: 304.0702

Preparation of (1*RS*,2*SR*,4*SR*)-2-(2-bromophenethyl)bicyclo[2.2.1]heptan-2-amine (7gc)

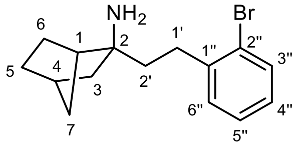

7gc

Following ***General Procedure 1***, exo-2-aminonorbornane (**1g**) (53 µL, 50 mg, 0.45 mmol, 1.0 equiv), 2-bromostyrene (**6c**) (57 µL, 83 mg, 0.45 mmol, 1.0 equiv), 3DPA2FBN (2.80 mM in DMF, 1.60 mL, 4.5 µmol, 1 mol%), tetrabutylammonium azide (70.3 mM in DMF, 1.28 mL, 90 µmol, 20 mol%) and DMF (120 µL) were reacted for 20 h with a 425 nm LED lamp. The mixture was concentrated *in vacuo* and purified *via* flash column chromatography on silica gel (12 g) in CH_2Cl_2 (5 CV) then 100:0:0→95:4.5:0.5 CH_2Cl_2–MeOH–aq. NH_4OH (over 20 CV) then 95:4.5:0.5 CH_2Cl_2–MeOH–aq. NH_4OH (5 CV) to give **7gc** as a brown oil (89.0 mg, 68%).

Data for **7gc**:

<u>^{1}H NMR:</u> (400 MHz, $CDCl_3$)
7.55–7.46 (m, 1H, C(3″)*H*), 7.24–7.19 (m, 2H, C(4″)*H*, C(6″)*H*), 7.06–7.00 (m, 1H, C(5″)*H*), 2.86–2.73 (m, 2H, C(1′)H_2), 2.23–2.18 (m, 1H, C(4)*H*), 2.08–2.03 (m, 1H, C(1)*H*), 1.91–1.83 (m, 1H, C(7)H_A), 1.75–1.66 (m, 3H, C(3)H_A, C(2′)H_2), 1.65–1.56 (m, 2H, C(5)H_A, C(6)H_A), 1.52–1.30 (m, 3H, C(7)H_B, NH_2), 1.29–1.21 (m, 2H, C(5)H_B, C(6)H_B), 1.00–0.93 (m, 1H, C(3)H_B)

<u>^{13}C NMR:</u> (101 MHz, $CDCl_3$)
142.3 (*C*(1″)), 132.9 (*C*(3″)), 130.5 (*C*(6″)), 127.6 (*C*(5″)), 127.5 (*C*(4″)), 124.5 (*C*(2″)), 59.0 (*C*(2)), 47.1 (*C*(1)), 46.9 (*C*(3)), 43.6 (*C*(2′)), 38.8 (*C*(6)), 37.7 (*C*(4)), 31.2 (*C*(1′)), 28.7 (*C*(5)), 23.3 (*C*(7))

<u>IR:</u> (neat)
2943 (m), 2866 (w), 1567 (w), 1471 (w), 1456 (w), 1438 (w), 1308 (w), 1229 (w), 1199 (w), 1109 (w), 1044 (w), 1021 (m), 937 (w), 820 (w), 798 (w), 746 (s), 657 (m)

<u>MS:</u> (ESI^+)
297 (16%), 296 ([M(^{81}Br)+H]$^+$, 99%), 295 (16%), 294 ([M(^{79}Br)+H]$^+$, 100%),

<u>HRMS:</u> (ESI^+)
Calcd for $C_{15}H_{21}$(^{79}Br)N: 294.0857, found: 294.0856

Preparation of 2-((2*RS*,3*rs*,5*rs*,7*rs*)-adamantan-1-yl)-4-(2-bromophenyl)butan-2-amine (7hc)

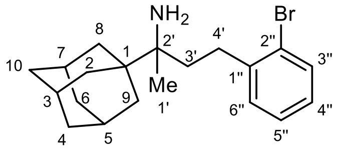

7hc

Following ***General Procedure 1***, Rimantadine (**1h**) (80 mg, 0.45 mmol, 1.0 equiv), 2-bromostyrene (**6c**) (57 µL, 83 mg, 0.45 mmol, 1.0 equiv), 3DPA2FBN (2.80 mM in DMF, 1.60 mL, 4.5 µmol, 1 mol%), tetrabutylammonium azide (70.3 mM in DMF, 1.28 mL, 90 µmol, 20 mol%) and DMF (120 µL) were reacted for 20 h with a 425 nm LED lamp. The mixture was concentrated *in vacuo* and purified *via* flash column chromatography on silica gel (12 g) in CH_2Cl_2 (5 CV) then 100:0:0→95:4.5:0.5 CH_2Cl_2–MeOH–aq. NH_4OH (over 20 CV) then 95:4.5:0.5 CH_2Cl_2–MeOH–aq. NH_4OH (5 CV) to give **7hc** as a white solid (103.0 mg, 63%).

Data for **7hc**:

mp: 86–88 °C (CH_2Cl_2)

^{1}H NMR: (400 MHz, $CDCl_3$)

7.59–7.47 (m, 1H, C(3″)*H*), 7.25–7.20 (m, 2H, C(4″)*H*, C(6″)*H*), 7.07–7.01 (m, 1H, C(5″)*H*), 2.90–2.81 (m, 1H, C(4′)H_A), 2.78–2.70 (m, 1H, C(4′)H_B), 2.01–1.95 (m, 3H, C(3)*H*, C(5)*H*, C(7)*H*), 1.69–1.50 (m, 14H, C(2)H_2, C(4)H_2, C(6)H_2, C(8)H_2, C(9)H_2, C(10)H_2, C(3′)H_2), 1.16–1.02 (m, 5H, C(1′)H_3, NH_2)

^{13}C NMR: (101 MHz, $CDCl_3$)

143.0 (*C*(1″)), 133.0 (*C*(3″)), 130.6 (*C*(6″)), 127.7 (*C*(5″)), 127.5 (*C*(4″)), 124.6 (*C*(2″)), 55.7 (*C*(2′)), 39.1 (*C*(1)), 37.3 (*C*(4), *C*(6), *C*(10)), 36.7 (*C*(3′)), 36.0 (*C*(2), *C*(8), *C*(9)), 31.5 (*C*(4′)), 28.9 (*C*(3), *C*(5), *C*(7)), 20.6 (*C*(1′))

IR: (neat)

2924 (w), 2901 (m), 2848 (w), 1612 (w), 1565 (w), 1471 (w), 1455 (w), 1438 (w), 1381 (w), 1362 (w), 1344 (w), 1281 (w), 1266 (w), 1208 (w), 1181 (w), 1149 (w), 1107 (w), 1072 (w), 1043 (w), 1023 (w), 986 (w), 975 (w), 940 (w), 832 (w), 816 (w), 775 (w), 747 (m), 720 (w), 692 (w), 658 (w)

MS: (ESI$^+$)

365 (21%), 364 ([M(^{81}Br)+H]$^+$, 100%), 363 (23%), 362 ([M(^{79}Br)+H]$^+$, 100%), 135 (17%), 126 (14%)

HRMS: (ESI$^+$)

Calcd for $C_{20}H_{29}$(^{79}Br)N: 362.1483, found: 362.1484

Preparation of 4-(2-bromophenethyl)tetrahydro-2*H*-pyran-4-amine (7ic)

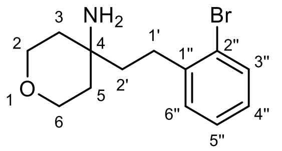

7ic

Following ***General Procedure 1***, 4-aminotetrahydropyran (**1i**) (47 µL, 46 mg, 0.45 mmol, 1.0 equiv), 2-bromostyrene (**6c**) (57 µL, 83 mg, 0.45 mmol, 1.0 equiv), 3DPA2FBN (2.80 mM in DMF, 1.60 mL, 4.5 µmol, 1 mol%), tetrabutylammonium azide (70.3 mM in DMF, 1.28 mL, 90 µmol, 20 mol%) and DMF (120 µL) were reacted for 20 h with a 425 nm LED lamp. The mixture was concentrated *in vacuo* and purified *via* flash column chromatography on silica gel (12 g) in CH_2Cl_2 (5 CV) then 100:0:0→95:4.5:0.5 CH_2Cl_2–MeOH–aq. NH_4OH (over 20 CV)

then 95:4.5:0.5 CH_2Cl_2–MeOH–aq. NH_4OH (5 CV) to give **7ic** as a yellow oil (108.0 mg, 85%).

Data for **7ic**:

^{1}H NMR: (400 MHz, $CDCl_3$)
7.55–7.48 (m, 1H, C(3″)*H*), 7.25–7.20 (m, 2H, C(4″)*H*, C(6″)*H*), 7.09–7.02 (m, 1H, C(5″)*H*), 3.82–3.71 (m, 4H, C(2)H_2, C(6)H_2), 2.84–2.74 (m, 2H, C(1′)H_2), 1.77–1.65 (m, 4H, C(3)H_A, C(5)H_A, C(2′)H_2), 1.47–1.41 (m, 2H, C(3)H_B, C(5)H_B), 1.31 (br s, 2H, NH_2)

^{13}C NMR: (101 MHz, $CDCl_3$)
141.8 (*C*(1″)), 133.0 (*C*(3″)), 130.4 (*C*(6″)), 127.8 (*C*(5″)), 127.8 (*C*(4″)), 124.5 (*C*(2″)), 64.2 (*C*(2) ,*C*(6)), 48.8 (*C*(4)), 44.2 (*C*(2′)), 38.8 (*C*(3), *C*(5)), 30.0 (*C*(1′))

IR: (neat)
2934 (w), 2860 (w), 1672 (w), 1592 (w), 1567 (w), 1471 (w), 1455 (w), 1439 (w), 1389 (w), 1355 (w), 1299 (w), 1237 (w), 1185 (w), 1107 (m), 1044 (w), 1022 (m), 986 (w), 842 (w), 752 (m), 713 (w), 660 (w)

MS: (ESI^+)
287 (14%), 286 ([M(^{81}Br)+H]$^+$, 98%), 285 (15%), 284 ([M(^{79}Br)+H]$^+$, 100%)

HRMS: (ESI^+)
Calcd for $C_{13}H_{19}$(^{79}Br)NO: 284.0650, found: 284.0650

Preparation of 4-(2-bromophenethyl)tetrahydro-2*H*-thiopyran-4-amine (7jc)

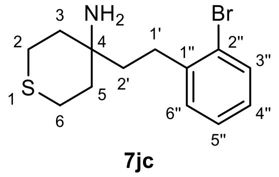

7jc

Following ***General Procedure 1***, 4-aminotetrahydrothiopyran (**1j**) (53 mg, 0.45 mmol, 1.0 equiv), 2-bromostyrene (**6c**) (57 µL, 83 mg, 0.45 mmol, 1.0 equiv), 3DPA2FBN (2.80 mM in DMF, 1.60 mL, 4.5 µmol, 1 mol%), tetrabutylammonium azide (70.3 mM in DMF, 1.28 mL, 90 µmol, 20 mol%) and DMF (120 µL) were reacted for 20 h with a 425 nm LED lamp. The mixture was concentrated *in vacuo* and purified *via* flash column chromatography on silica gel (12 g) in CH_2Cl_2 (5 CV) then 100:0:0→95:4.5:0.5 CH_2Cl_2–MeOH–aq. NH_4OH (over 20 CV)

then 95:4.5:0.5 CH_2Cl_2–MeOH–aq. NH_4OH (5 CV), followed by reversed-phase flash column chromatography on C_{18} silica gel (15.5 g) in 98:2 H_2O–MeOH (5 CV) then 98:2→0:100 H_2O–MeOH (over 15 CV) then MeOH (5 CV) to give **7jc** as a colourless oil (97 mg, 72%).

Data for **7jc**:

^{1}H NMR: (400 MHz, $CDCl_3$)
7.56–7.47 (m, 1H, C(3″)*H*), 7.25–7.18 (m, 2H, C(4″)*H*, C(6″)*H*), 7.09–7.03 (m, 1H, C(5″)*H*), 2.86 (ddd, *J* = 13.3, 9.6, 3.1 Hz, 2H, C(2)H_A, C(6)H_A), 2.80–2.73 (m, 2H, C(1′)H_2), 2.58 (ddd, *J* = 13.3, 7.3, 3.4 Hz, 2H, C(2)H_B, C(6)H_B), 1.87 (ddd, *J* = 13.3, 9.6, 3.4 Hz, 2H, C(3)H_A, C(5)H_A), 1.74 (ddd, *J* = 13.3, 7.3, 3.1 Hz, 2H, C(3)H_B, C(5)H_B), 1.65–1.59 (m, 2H, C(2′)H_2), 1.22 (br s, 2H, NH_2)

^{13}C NMR: (101 MHz, $CDCl_3$)
141.8 (*C*(1″)), 133.0 (*C*(3″)), 130.5 (*C*(6″)), 127.8 (*C*(5″)), 127.8 (*C*(4″)), 124.4 (*C*(2″)), 49.8 (*C*(4)), 43.7 (*C*(2′)), 39.4 (*C*(3), *C*(5)), 29.9 (*C*(1′)), 24.5 (*C*(2), *C*(6))

IR: (neat)
2920 (w), 1566 (w), 1471 (m), 1437 (w), 1273 (w), 1223 (w), 1083 (w), 1044 (w), 1023 (m), 931 (w), 821 (w), 751 (m), 658 (m)

MS: (ESI^+)
303 (14%), 302 ($[M(^{81}Br)+H]^+$, 100%), 301 (14%), 300 ($[M(^{79}Br)+H]^+$, 98%), 285 (12%), 283 (13%)

HRMS: (ESI^+)
Calcd for $C_{13}H_{19}(^{79}Br)NS$: 300.0422, found: 300.0422

Preparation of *tert*-butyl 4-amino-4-(2-bromophenethyl)piperidine-1-carboxylate (7kc)

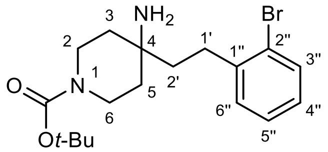

7kc

Following ***General Procedure 1***, *tert*-butyl 4-amino-1-piperidinecarboxylate (**1k**) (90 mg, 0.45 mmol, 1.0 equiv), 2-bromostyrene (**6c**) (57 μL, 83 mg, 0.45 mmol, 1.0 equiv), 3DPA2FBN (2.80 mM in DMF, 1.60 mL, 4.5 μmol, 1 mol%), tetrabutylammonium azide (70.3 mM in DMF, 1.28 mL, 90 μmol, 20 mol%) and DMF (120 μL) were reacted for 20 h with a 425 nm

LED lamp. The mixture was concentrated *in vacuo* and purified *via* flash column chromatography on silica gel (12 g) in CH_2Cl_2 (5 CV) then 100:0:0→95:4.5:0.5 CH_2Cl_2–MeOH–aq. NH_4OH (over 20 CV) then 95:4.5:0.5 CH_2Cl_2–MeOH–aq. NH_4OH (5 CV) to give **7kc** as a yellow oil (145.2 mg, 84%).

Data for **7kc**:

^{1}H NMR: (400 MHz, $CDCl_3$)
7.55–7.46 (m, 1H, C(3″)*H*), 7.24–7.16 (m, 2H, C(4″)*H*, C(6″)*H*), 7.08–7.02 (m, 1H, C(5″)*H*), 3.75–3.58 (m, 2H, C(2)H_A, C(6)H_A), 3.38–3.28 (m, 2H, C(2)H_B, C(6)H_B), 2.83–2.73 (m, 2H, C(1′)H_2), 1.67–1.55 (m, 4H, C(3)H_A, C(5)H_A, C(2′)H_2), 1.47–1.41 (m, 11H, C(3)H_B, C(5)H_B, OC(CH_3)$_3$), 1.24 (br s, 2H, NH_2)

^{13}C NMR: (101 MHz, $CDCl_3$)
154.9 (*C*=O), 141.7 (*C*(1″)), 132.9 (*C*(3″)), 130.3 (*C*(6″)), 127.7 (*C*(5″)), 127.7 (*C*(4″)), 124.3 (*C*(2″)), 79.4 (O*C*(CH_3)$_3$), 49.5 (*C*(4)), 43.7 (*C*(2′)), 40.0 (*C*(2), *C*(6)), 37.7 (*C*(3), *C*(5)), 30.0 (*C*(1′)), 28.5 (OC(*C*H_3)$_3$)

IR: (neat)
2974 (w), 2932 (w), 2867 (w), 1682 (m), 1471 (w), 1453 (w), 1421 (m), 1391 (w), 1364 (m), 1276 (w), 1262 (w), 1245 (m), 1164 (m), 1150 (m), 1122 (w), 1090 (w), 1023 (m), 971 (w), 864 (w), 824 (w), 753 (m)

MS: (ESI$^+$)
386 (20%), 385 ([M(^{81}Br)+H]$^+$, 99%), 384 (21%), 383 ([M(^{79}Br)+H]$^+$, 100%), 329 (26%), 327 (25%)

HRMS: (ESI$^+$)
Calcd for $C_{18}H_{28}(^{79}Br)N_2O_2$: 383.1334, found: 383.1334

Preparation of *tert*-butyl 3-amino-3-(2-bromophenethyl)azetidine-1-carboxylate (7lc)

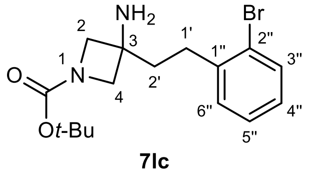

7lc

Following ***General Procedure 1***, *tert*-Butyl 3-aminoazetidine-1-carboxylate (**1l**) (78 mg, 0.45 mmol, 1.0 equiv), 2-bromostyrene (**6c**) (57 µL, 83 mg, 0.45 mmol, 1.0 equiv), 3DPA2FBN (2.80 mM in DMF, 1.60 mL, 4.5 µmol, 1 mol%), tetrabutylammonium azide (70.3 mM in DMF, 1.28 mL, 90 µmol, 20 mol%) and DMF (120 µL) were reacted for 20 h with a 425 nm LED lamp. The mixture was concentrated *in vacuo* and purified *via* flash column

chromatography on silica gel (12 g) in CH_2Cl_2 (5 CV) then 100:0:0→95:4.5:0.5 CH_2Cl_2–MeOH–aq. NH_4OH (over 20 CV) then 95:4.5:0.5 CH_2Cl_2–MeOH–aq. NH_4OH (5 CV) to give **7lc** as a yellow oil (67.4 mg, 42%).

Data for **7lc**:

^{1}H NMR: (400 MHz, $CDCl_3$)
7.54–7.51 (m, 1H, C(3″)*H*), 7.29–7.19 (m, 2H, C(4″)*H*, C(6″)*H*), 7.10–7.04 (m, 1H, C(5″)*H*), 3.87 (d, *J* = 8.8 Hz, 2H, C(2)H_A, C(4)H_A), 3.65 (d, *J* = 8.8 Hz, 2H, C(2)H_B, C(4)H_B), 2.82–2.77 (m, 2H, C(1′)H_2), 1.97–1.92 (m, 2H, C(2′)H_2), 1.67 (br s, 2H, NH_2), 1.44 (s, 9H, OC(CH_3)$_3$)

^{13}C NMR: (101 MHz, $CDCl_3$)
156.7 (*C*=O), 140.9 (*C*(1″)), 133.1 (*C*(3″)), 130.5 (*C*(6″)), 128.0 (*C*(5″)), 127.8 (*C*(4″)), 124.4 (*C*(2″)), 79.7 (O*C*(CH_3)$_3$), 62.7 (br s, *C*(2), *C*(4)), 51.8 (*C*(3)), 40.1 (*C*(2′)), 31.1 (*C*(1′)), 28.5 (OC(*C*H_3)$_3$)

IR: (neat)
3297 (w), 2975 (w), 2875 (w), 1686 (m), 1567 (w), 1473 (w), 1455 (w), 1403 (m), 1365 (m), 1251 (w), 1147 (m), 1120 (m), 1045 (w), 1022 (w), 907 (w), 859 (w), 771 (w), 751 (m), 731 (w), 700 (w), 658 (w)

MS: (ESI^+)
480 (10%), 304 (26%), 301 (59%), 299 (62%), 283 (19%), 282 ([M(^{79}Br)+H–O*t*-Bu]$^+$, 100%), 280 (13%)

HRMS: (ESI^+)
Calcd for $C_{16}H_{23}(^{79}Br)N_2NaO_2$: 377.0841, found: 377.0837

Preparation of (*RS*)-1-(2-bromophenyl)-6,6-diethoxyhexan-3-amine (7mc) and 3-(2-bromophenethyl)-1-(2-bromophenyl)-6,6-diethoxyhexan-3-amine (29)

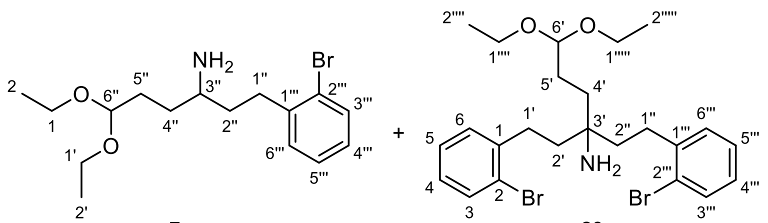

7mc **29**

Following ***General Procedure 1***, 4-aminobutyraldehyde diethyl acetal (**1m**) (78 μL, 73 mg, 0.45 mmol, 1.0 equiv), 2-bromostyrene (**6c**) (57 μL, 83 mg, 0.45 mmol, 1.0 equiv), 3DPA2FBN

(2.80 mM in DMF, 1.60 mL, 4.5 μmol, 1 mol%), tetrabutylammonium azide (70.3 mM in DMF, 1.28 mL, 90 μmol, 20 mol%) and DMF (120 μL) were reacted for 20 h with a 425 nm LED lamp. The mixture was concentrated *in vacuo* and purified *via* flash column chromatography on silica gel (12 g) in CH_2Cl_2 (5 CV) then 100:0:0→95:4.5:0.5 CH_2Cl_2–MeOH–aq. NH_4OH (over 20 CV) then 95:4.5:0.5 CH_2Cl_2–MeOH–aq. NH_4OH (5 CV) to give **7mc** as a colourless oil (123.0 mg, 79%) and **29** as a yellow oil (5 mg).

Data for **7mc**:

^{1}H NMR: (400 MHz, $CDCl_3$)
7.57–7.45 (m, 1H, C(3‴)*H*), 7.24–7.17 (m, 2H, C(4‴)*H*, C(6‴)*H*), 7.08–7.00 (m, 1H, C(5‴)*H*), 4.51–4.44 (m, 1H, C(6″)*H*), 3.68–3.59 (m, 2H, C(1)H_2 or C(1′)H_2), 3.53–3.44 (m, 2H, C(1)H_2 or C(1′)H_2), 2.90–2.70 (m, 3H, C(1″)H_2, C(3″)*H*), 1.78–1.50 (m, 5H, C(2″)H_2, C(4″)H_A, C(5″)H_2), 1.42–1.34 (m, 1H, C(4″)H_B), 1.30 (br s, 2H, NH_2), 1.22–1.17 (m, 6H, C(2)H_3, C(2′)H_3)

^{13}C NMR: (101 MHz, $CDCl_3$)
141.8 (*C*(1‴)), 132.9 (*C*(3‴)), 130.4 (*C*(6‴)), 127.6 (*C*(5‴)), 127.6 (*C*(4‴)), 124.5 (*C*(2‴)), 103.1 (*C*(6″)), 61.3 (*C*(1) or *C*(1′)), 61.1 (*C*(1) or *C*(1′)), 51.0 (*C*(3″)), 38.4 (*C*(2″), 33.2 (*C*(4″)), 33.0 (*C*(1″)), 30.4 (*C*(5″)), 15.5 (*C*(2), *C*(2′))

IR: (neat)
2973 (w), 2928 (w), 2352 (w), 1567 (w), 1471 (w), 1440 (w), 1373 (w), 1344 (w), 1122 (m), 1057 (m), 1022 (m), 816 (w), 748 (m), 668 (w), 658 (w)

MS: (ESI^+)
530 (33%), 529 (17%), 528 (67%), 526 (32%), 347 (17%), 346 ($[M(^{81}Br)+H]^+$, 98%), 345 (17%), 344 ($[M(^{79}Br)+H]^+$, 100%), 300 (18%), 298 (21%)

HRMS: (ESI^+)
Calcd for $C_{16}H_{27}(^{79}Br)NO_2$: 344.1225, found: 344.1223

Data for **29**:

^{1}H NMR: (400 MHz, $CDCl_3$)
7.59–7.47 (m, 2H, C(3)*H*, C(3‴)*H*), 7.28–7.22 (m, 4H, C(4)*H*, C(4‴)*H*, C(6)*H*, C(6‴)*H*), 7.10–7.03 (m, 2H, C(5)*H*, C(5‴)*H*), 4.54–4.50 (m, 1H, C(6′)*H*), 3.72–3.64 (m, 2H, C(1⁗)H_2), 3.57–3.49 (m, 2H, C(1⁗′)H_2), 2.90–2.74 (m, 4H,

C(1′)*H*2, C(1″)*H*2), 1.79–1.67 (m, 6H, C(2′)*H*2, C(5′)*H*2, C(2″)*H*2), 1.62–1.56 (m, 2H, C(4′)*H*2), 1.41 (s, 2H, N*H*2), 1.25–1.21 (m, 6H, C(2″″)*H*3, C(2″‴)*H*3)

^{13}C NMR: (101 MHz, $CDCl_3$)

142.0 (*C*(1), *C*(1‴)), 133.0 (*C*(3), *C*(3‴)), 130.5 (*C*(6), *C*(6‴)), 127.8 (*C*(5), *C*(5‴)), 127.7 (*C*(4), *C*(4‴)), 124.4 (*C*(2), *C*(2‴)), 103.5 (*C*(6′)), 61.4 (*C*(1″″), *C*(1″‴)), 53.6 (*C*(3′)), 40.5 (*C*(2′), *C*(2″)), 34.8 (*C*(4′)), 30.8 (*C*(1′), *C*(1″)), 28.2 (*C*(5′)), 15.5 (*C*(2″″), *C*(2″‴))

IR: (neat)

2972 (w), 2930 (w), 2872 (w), 1680 (w), 1567 (w), 1471 (w), 1455 (w), 1439 (w), 1373 (w), 1343 (w), 1124 (m), 1058 (m), 1021 (m), 822 (w), 747 (m), 659 (m)

MS: (ESI$^+$)

531 (12%), 530 ([M(^{79}Br)(^{81}Br)+H]$^+$, 50%), 529 (26%), 528 ([M(^{79}Br)(^{79}Br)+H]$^+$, 100%), 527 (13%)

HRMS: (ESI$^+$)

Calcd for $C_{24}H_{34}$(^{79}Br)(^{79}Br)NO_2: 528.0930, found: 528.0933

Preparation of (*RS*)-4-amino-6-(2-bromophenyl)hexan-1-ol (7nc) and 4-amino-4-(2-bromophenethyl)-6-(2-bromophenyl)hexan-1-ol (30)

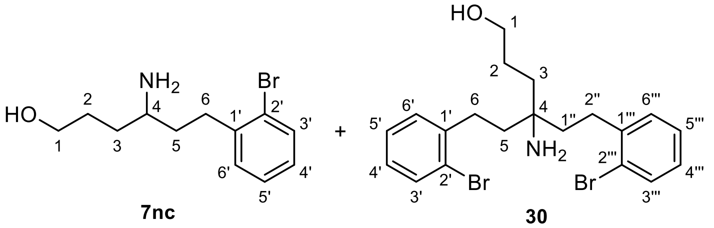

7nc **30**

Following ***General Procedure 1***, 4-amino-1-butanol (**1n**) (125 μL, 120 mg, 1.35 mmol, 3.0 equiv), 2-bromostyrene (**6c**) (57 μL, 83 mg, 0.45 mmol, 1.0 equiv), 3DPA2FBN (2.80 mM in DMF, 1.60 mL, 4.5 μmol, 1 mol%), tetrabutylammonium azide (70.3 mM in DMF, 1.28 mL, 90 μmol, 20 mol%) and DMF (120 μL) were reacted for 20 h with a 425 nm LED lamp. The mixture was concentrated *in vacuo* and purified *via* flash column chromatography on silica gel (12 g) in CH_2Cl_2 (5 CV) then 100:0:0→95:4.5:0.5 CH_2Cl_2–MeOH–aq. NH_4OH (over 20 CV) then 95:4.5:0.5 CH_2Cl_2–MeOH–aq. NH_4OH (5 CV), followed by reversed-phase flash column chromatography on C_{18} silica gel (15.5 g) in 98:2 H_2O–MeOH (5 CV) then 98:2→0:100 H_2O–MeOH (over 15 CV) then MeOH (5 CV) to give **7nc** as a colourless oil (102.5 mg, 84%) and **30** as a yellow oil (10.1 mg, 5% wrt **6c**).

The reaction was repeated with 4-amino-1-butanol (**1n**) (42 µL, 40 mg, 0.45 mmol, 1.0 equiv) to give **7nc** as a colourless oil (35.0 mg, 29%) and **30** as a yellow oil (93.0 mg, 46% wrt **1n**).

Data for **7nc**:

^{1}H NMR: (400 MHz, $CDCl_3$)
7.58–7.47 (m, 1H, C(3′)*H*), 7.26–7.16 (m, 2H, C(4′)*H*, C(6′)*H*), 7.12–6.97 (m, 1H, C(5′)*H*), 3.69–3.54 (m, 2H, C(1)H_2), 2.86–2.70 (m, 3H, C(4)*H*, C(6)H_2), 2.54 (br s, 2H, NH_2), 1.83–1.71 (m, 3H, C(2)H_A, C(3)H_A, C(5)H_A), 1.69–1.56 (m, 2H, C(2)H_B, C(5)H_B), 1.45–1.33 (m, 1H, C(3)H_B)

^{13}C NMR: (101 MHz, $CDCl_3$)
141.3 (*C*(1′)), 133.0 (*C*(3′)), 130.4 (*C*(6′)), 127.9 (*C*(5′)), 127.7 (*C*(4′)), 124.5 (*C*(2′)), 63.1 (*C*(1)), 51.4 (*C*(4)), 39.5 (*C*(5)), 36.1 (*C*(3)), 33.1 (*C*(5)), 30.7 (*C*(2))

IR: (neat)
3280 (w), 3061 (w), 2920 (w), 2855 (w), 1587 (w), 1567 (w), 1471 (m), 1452 (w), 1438 (w), 1363 (w), 1057 (w), 1021 (m), 941 (w), 748 (m), 718 (w), 658 (w)

MS: (ESI^+)
275 (12%), 274 ([M(^{81}Br)+H]$^+$, 93%), 273 (13%), 272 ([M(^{79}Br)+H]$^+$, 100%), 126 (22%)

HRMS: (ESI^+)
Calcd for $C_{12}H_{19}(^{79}Br)NO$: 272.0650, found: 272.0649

Data for **30**:

^{1}H NMR: (400 MHz, $CDCl_3$)
7.58–7.48 (m, 2H, C(3′)*H*, C(3‴)*H*), 7.28–7.20 (m, 4H, C(4′)*H*, C(6′)*H*, C(4‴)*H*, C(6‴)*H*), 7.10–7.03 (m, 2H, C(5′)*H*, C(5‴)*H*), 3.72–3.60 (m, 2H, C(1)H_2), 2.85–2.72 (m, 4H, C(6)H_2, C(2″)H_2), 2.38 (br s, 2H, NH_2), 1.87–1.65 (m, 8H, C(2)H_2, C(3)H_2, C(5)H_2, C(1″)H_2,).

^{13}C NMR: (101 MHz, $CDCl_3$)
141.6 (*C*(1′), *C*(1‴)), 133.0 (*C*(3′), *C*(3‴)), 130.5 (*C*(6′), *C*(6‴)), 127.9 (*C*(5′), *C*(5‴)), 127.9 (*C*(4′), *C*(4‴)), 124.4 (*C*(2′), *C*(2‴)), 63.5 (*C*(1)), 53.6 (*C*(4)), 40.2 (*C*(5), *C*(1″)), 38.4 (*C*(3)), 30.8 (*C*(6), *C*(2″)), 27.5 (*C*(2))

IR: (neat)

3278 (w), 2934 (w), 2863 (w), 1589 (w), 1567 (w), 1471 (m), 1455 (w), 1438 (w), 1057 (w), 1045 (w), 1022 (m), 908 (w), 747 (m), 731 (m), 659 (w)

MS: (ESI^+)

459 (10%), 458 ([M(^{81}Br)(^{81}Br)+H]$^+$, 50%), 457 (21%), 456 ([M(^{79}Br)(^{81}Br)+H]$^+$, 100%), 455 (11%), 454 ([M(^{79}Br)(^{79}Br)+H]$^+$,51%), 282 (16%)

HRMS: (ESI^+)

Calcd for $C_{20}H_{26}$(^{79}Br)(^{81}Br)NO: 456.0361, found: 456.0358

Preparation of ethyl (*RS*)-3-amino-5-(2-bromophenyl)-3-methylpentanoate (7oc)

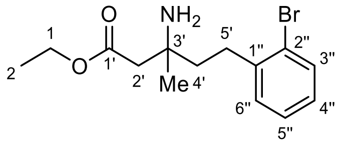

7oc

Following ***General Procedure 1***, ethyl 3-aminobutyrate (**1o**) (66 µL, 59 mg, 0.45 mmol, 1.0 equiv), 2-bromostyrene (**6c**) (57 µL, 83 mg, 0.45 mmol, 1.0 equiv), 3DPA2FBN (2.80 mM in DMF, 1.60 mL, 4.5 µmol, 1 mol%), tetrabutylammonium azide (70.3 mM in DMF, 1.28 mL, 90 µmol, 20 mol%) and DMF (120 µL) were reacted for 20 h with a 425 nm LED lamp. The mixture was concentrated *in vacuo* and purified *via* flash column chromatography on silica gel (12 g) in CH_2Cl_2 (5 CV) then 100:0:0→95:4.5:0.5 CH_2Cl_2–MeOH–aq. NH_4OH (over 20 CV) then 95:4.5:0.5 CH_2Cl_2–MeOH–aq. NH_4OH (5 CV) to give **7oc** as a yellow oil (92.1 mg, 65%).

Data for **7oc**:

^{1}H NMR: (400 MHz, $CDCl_3$)

7.54–7.47 (m, 1H, C(3″)*H*), 7.25–7.19 (m, 2H, C(4″)*H*, C(6″)*H*), 7.07–7.01 (m, 1H, C(5″)*H*), 4.19–4.12 (m, 2H, C(1)H_2), 2.85–2.74 (m, 2H, C(5′)H_2), 2.49 (d, J = 14.6 Hz, 1H, C(2′)H_A), 2.45 (d, J = 14.6 Hz, 1H, C(2′)H_B), 1.75–1.66 (m, 4H, C(4′)H_2, NH_2), 1.30–1.25 (m, 6H, C(2)H_3, C(3′)*Me*)

^{13}C NMR: (101 MHz, $CDCl_3$)

172.1 (*C*(1′)), 141.7 (*C*(1″)), 133.0 (*C*(3″)), 130.4 (*C*(6″)), 127.6 (*C*(5″)), 127.7 (*C*(4″)), 124.5 (*C*(2″)), 60.4 (*C*(1)), 51.4 (*C*(3′)), 46.9 (*C*(2′)), 43.6 (*C*(4′)), 31.3 (*C*(5′)), 28.0 (C(3′)*Me*), 14.4 (*C*(2))

IR: (neat)

3290 (w), 3054 (w), 2973 (w), 2932 (w), 2872 (w), 1726 (m), 1654 (w), 1566 (w), 1543 (w), 1471 (w), 1440 (w), 1371 (w), 1299 (w), 1255 (w), 1184 (w), 1096 (w), 1023 (w), 856 (w), 751 (w), 701 (w), 659 (w)

MS: (ESI^+)

402 (16%), 401 (82%), 400 (16%), 399 (82%), 317 (15%), 316 ($[M(^{81}Br)+H]^+$, 96%), 315 (16%), 314 ($[M(^{79}Br)+H]^+$, 100%)

HRMS: (ESI^+)

Calcd for $C_{14}H_{21}(^{79}Br)NO_2$: 314.0756, found: 314.0754

Preparation of (*RS*)-3-amino-5-(2-bromophenyl)pentanenitrile (7pc)

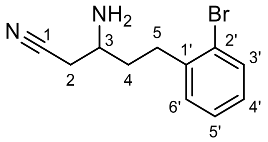

7pc

Following ***General Procedure 1***, 3-amino-propionitrile (**1p**) (32 mg, 0.45 mmol, 1.0 equiv), 2-bromostyrene (**6c**) (57 µL, 83 mg, 0.45 mmol, 1.0 equiv), 3DPA2FBN (2.80 mM in DMF, 1.60 mL, 4.5 µmol, 1 mol%), tetrabutylammonium azide (70.3 mM in DMF, 1.28 mL, 90 µmol, 20 mol%) and DMF (120 µL) were reacted for 20 h with a 425 nm LED lamp. The mixture was concentrated *in vacuo* and purified *via* flash column chromatography on silica gel (12 g) in CH_2Cl_2 (5 CV) then 100:0:0→95:4.5:0.5 CH_2Cl_2–MeOH–aq. NH_4OH (over 20 CV) then 95:4.5:0.5 CH_2Cl_2–MeOH–aq. NH_4OH (5 CV) to give **7pc** as a yellow oil (64.4 mg, 57%).

Data for **7pc**:

^{1}H NMR: (400 MHz, $CDCl_3$)

7.59–7.48 (m, 1H, C(3′)*H*), 7.28–7.22 (m, 2H, C(4′)*H*, C(6′)*H*), 7.13–7.03 (m, 1H, C(5′)*H*), 3.15 (dddd, *J* = 8.1, 6.7, 5.0, 4.9 Hz, 1H, C(3)*H*), 2.90 (ddd, *J* = 13.6, 10.0, 5.5 Hz, 1H, C(5)H_A), 2.79 (ddd, *J* = 13.6, 10.0, 6.3 Hz, 1H, C(5)H_B), 2.53 (dd, *J* = 16.6, 4.9 Hz, 1H, C(2)H_A), 2.42 (dd, *J* = 16.6, 6.7 Hz, 1H, C(2)H_B), 1.86 (dddd, *J* = 13.6, 10.0, 6.3, 5.0 Hz, 1H, C(4)H_A), 1.74 (dddd, *J* = 13.6, 10.0, 8.1, 5.5 Hz, 1H, C(4)H_B), 1.47 (s, 2H, NH_2)

^{13}C NMR: (101 MHz, $CDCl_3$)

140.4 (*C*(1′)), 133.1 (*C*(3′)), 130.5 (*C*(6′)), 128.1 (*C*(5′)), 127.8 (*C*(4′)), 124.4 (*C*(2′)), 118.1 (*C*(1)), 48.3 (*C*(3)), 37.4 (*C*(4)), 32.8 (*C*(5)), 27.3 (*C*(2))

IR: (neat)

3376 (w), 3059 (w), 2924 (w), 2861 (w), 2245 (w), 2194 (w), 1627 (w), 1567 (w), 1471 (w), 1454 (w), 1439 (w), 1421 (w), 1385 (w), 1361 (w), 1120 (w), 1045 (w), 1022 (w), 944 (w), 831 (w), 750 (m), 720 (w), 659 (w)

MS: (ESI^+)

507 (12%), 256 (13%), 255 ($[M(^{81}Br)+H]^+$, 100%), 254 (12%), 253 ($[M(^{79}Br)+H]^+$, 98%)

HRMS: (ESI^+)

Calcd for $C_{11}H_{14}(^{81}Br)N_2$: 255.0320, found: 255.0321

Preparation of (*RS*)-4-(2-bromophenyl)-1-methoxy-2-methylbutan-2-amine (7qc)

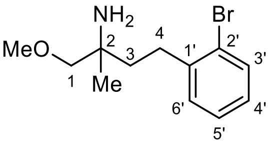

7qc

Following ***General Procedure 1***, 1-methoxy-2-propylamine (**1q**) (48 µL, 40 mg, 0.45 mmol, 1.0 equiv), 2-bromostyrene (**6c**) (57 µL, 83 mg, 0.45 mmol, 1.0 equiv), 3DPA2FBN (2.80 mM in DMF, 1.60 mL, 4.5 µmol, 1 mol%), tetrabutylammonium azide (70.3 mM in DMF, 1.28 mL, 90 µmol, 20 mol%) and DMF (120 µL) were reacted for 20 h with a 425 nm LED lamp. The mixture was concentrated *in vacuo* and purified *via* flash column chromatography on silica gel (12 g) in CH_2Cl_2 (5 CV) then 100:0:0→95:4.5:0.5 CH_2Cl_2–MeOH–aq. NH_4OH (over 20 CV) then 95:4.5:0.5 CH_2Cl_2–MeOH–aq. NH_4OH (5 CV) to give **7qc** as an orange oil (83.7 mg, 69%).

Data for **7qc**:

^{1}H NMR: (400 MHz, $CDCl_3$)

7.54–7.49 (m, 1H, C(3′)*H*), 7.25–7.19 (m, 2H, C(4′)*H*, C(6′)*H*), 7.07–7.00 (m, 1H, C(5′)*H*), 3.38 (s, 3H, O*Me*), 3.25–3.17 (m, 2H, C(1)H_2), 2.85–2.68 (m, 2H, C(4)H_2), 1.74–1.58 (m, 2H, C(3)H_2), 1.44 (s, 2H, NH_2), 1.15 (s, 3H, C(2)*Me*)

^{13}C NMR: (101 MHz, $CDCl_3$)

142.1 (*C*(1′)), 132.9 (*C*(3′)), 130.4 (*C*(6′)), 127.7 (*C*(5′)), 127.6 (*C*(4′)), 124.5 (*C*(2′)), 81.9 (*C*(1)), 59.4 (O*Me*), 52.4 (*C*(2)), 40.6 (*C*(3)), 31.1 (*C*(1)), 25.0 (C(2)*Me*)

IR: (neat)

2960 (w), 2923 (w), 2872 (w), 2826 (w), 1591 (w), 1567 (w), 1471 (w), 1458 (w), 1439 (w), 1377 (w), 1191 (w), 1156 (w), 1109 (m), 1045 (w), 1023 (w), 968 (w), 941 (w), 858 (w), 829 (w), 747 (m), 660 (w)

MS: (ESI^+)

275 (12%), 274 ($[M(^{81}Br)+H]^+$, 94%), 273 (13%), 272 ($[M(^{79}Br)+H]^+$, 100%)

HRMS: (ESI^+)

Calcd for $C_{12}H_{19}(^{79}Br)NO$: 272.0650, found: 272.0649

Preparation of (*RS*)-4-(2-bromophenyl)-1-methoxy-2-methylbutan-2-amine (7rc)

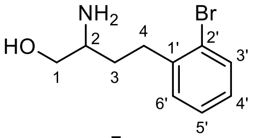

7rc

Following ***General Procedure 1***, ethanolamine (**1r**) (82 µL, 83 mg, 1.35 mmol, 3.0 equiv), 2-bromostyrene (**6c**) (57 µL, 83 mg, 0.45 mmol, 1.0 equiv), 3DPA2FBN (2.80 mM in DMF, 1.60 mL, 4.5 µmol, 1 mol%), tetrabutylammonium azide (70.3 mM in DMF, 1.28 mL, 90 µmol, 20 mol%) and DMF (120 µL) were reacted for 20 h with a 425 nm LED lamp. The mixture was concentrated *in vacuo* and purified *via* flash column chromatography on silica gel (12 g) in CH_2Cl_2 (5 CV) then 100:0:0→95:4.5:0.5 CH_2Cl_2–MeOH–aq. NH_4OH (over 20 CV) then 95:4.5:0.5 CH_2Cl_2–MeOH–aq. NH_4OH (5 CV) to give **7rc** as a clear oil (88.0 mg, 80%).

Data for **7rc**:

^{1}H NMR: (400 MHz, $CDCl_3$)

7.59–7.43 (m, 1H, C(3′)*H*), 7.26–7.17 (m, 2H, C(4′)*H*, C(6′)*H*), 7.11–7.02 (m, 1H, C(5′)*H*), 3.67–3.58 (m, 1H, C(1)H_A), 3.38–3.28 (m, 1H, C(1)H_B), 2.96–2.82 (m, 2H, C(2)*H*, C(4)H_A), 2.81–2.73 (m, 1H, C(4)H_B), 1.79–1.71 (m, 3H, C(3)H_A, NH_2), 1.61–1.51 (m, 1H, C(3)H_B)

^{13}C NMR: (101 MHz, $CDCl_3$)

141.3 (*C*(1′)), 133.0 (*C*(3′)), 130.4 (*C*(6′)), 127.9 (*C*(5′)), 127.7 (*C*(4′)), 124.5 (*C*(2′)), 67.0 (*C*(1)), 52.6 (*C*(2)), 35.1 (*C*(3)), 32.9 (*C*(4))

IR: (neat)

3377 (w), 3011 (w), 2933 (w), 2916 (w), 2857 (w), 1640 (w), 1567 (m), 1536 (w), 1468 (m), 1438 (m), 1380 (m), 1313 (m), 1305 (m), 1071 (w), 1056 (m), 1040 (w), 1020 (m), 817 (w), 764 (m), 749 (m)

MS: (ESI^+)

247 (12%), 246 ($[M(^{81}Br)+H]^+$, 98%), 245 (12%), 244 ($[M(^{79}Br)+H]^+$, 100%)

HRMS: (ESI^+)

Calcd for $C_{10}H_{15}(^{79}Br)NO$: 244.0337, found: 244.0334

Preparation of *tert*-butyl (*RS*)-(2-amino-4-(2-bromophenyl)butyl)carbamate (7sc) and *tert*-butyl (2-amino-2-(2-bromophenethyl)-4-(2-bromophenyl)butyl)carbamate (31)

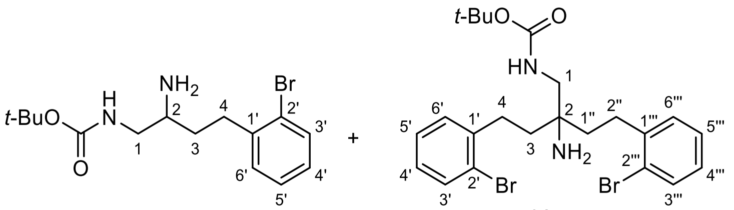

7sc **31**

Following ***General Procedure 1***, *N*-Boc-ethylenediamine (**1s**) (214 µL, 216 mg, 1.35 mmol, 3.0 equiv), 2-bromostyrene (**6c**) (57 µL, 83 mg, 0.45 mmol, 1.0 equiv), 3DPA2FBN (2.80 mM in DMF, 1.60 mL, 4.5 µmol, 1 mol%), tetrabutylammonium azide (70.3 mM in DMF, 1.28 mL, 90 µmol, 20 mol%) and DMF (120 µL) were reacted for 20 h with a 425 nm LED lamp. The mixture was concentrated *in vacuo* and purified *via* flash column chromatography on silica gel (12 g) in CH_2Cl_2 (5 CV) then 100:0:0→95:4.5:0.5 CH_2Cl_2–MeOH–aq. NH_4OH (over 20 CV) then 95:4.5:0.5 CH_2Cl_2–MeOH–aq. NH_4OH (5 CV) to give **7sc** as an orange oil (78.5 mg, 51%).

The reaction was repeated with *N*-Boc-ethylenediamine (**1s**) (71 µL, 72 mg, 0.45 mmol, 1.0 equiv) to give **7sc** as an orange oil (40.1 mg, 26%) and **31** as an orange solid (97.1 mg, 41% wrt **1s**).

Data for **7sc**:

^1H NMR: (400 MHz, $CDCl_3$)

7.57–7.47 (m, 1H, C(3′)*H*), 7.25–7.18 (m, 2H, C(4′)*H*, C(6′)*H*), 7.09–7.02 (m, 1H, C(5′)*H*), 4.95 (s, 1H C(1)N*H*), 3.33–3.21 (m, 1H, C(1)H_A), 3.03–2.81 (m,

3H, C(1)H_B, C(2)*H*, C(4)H_A), 2.81–2.73 (m, 1H, C(4)H_B), 1.79–1.70 (m, 1H, C(3)H_A), 1.60–1.51 (m, 1H, C(3)H_B), 1.46–1.35 (m, 11H, OC(CH_3)$_3$, NH_2)

^{13}C NMR: (101 MHz, $CDCl_3$)

156.4 (*C*=O), 141.3 (*C*(1′)), 133.0 (*C*(3′)), 130.5 (*C*(6′)), 127.9 (*C*(5′)), 127.7 (*C*(4′)), 124.5 (*C*(2′)), 79.4 (O*C*(CH_3)$_3$), 51.2 (*C*(2)), 47.0 (*C*(1)), 36.2 (*C*(3)), 32.9 (*C*(4)), 28.6 (OC(*C*H_3)$_3$)

IR: (neat)

3352 (w), 2974 (w), 2927 (w), 2865 (w), 1693 (m), 1512 (w), 1471 (w), 1453 (w), 1439 (w), 1390 (w), 1365 (m), 1270 (w), 1249 (m), 1167 (m), 1043 (w), 1022 (w), 863 (w), 750 (m), 659 (w)

MS: (ESI$^+$)

346 (16%), 345 ([M(^{81}Br)+H]$^+$, 94%), 344 (16%), 343 ([M(^{79}Br)+H]$^+$, 100%), 289 (14%), 287 (14%), 282 (31%), 126 (17%)

HRMS: (ESI$^+$)

Calcd for $C_{15}H_{24}$(^{79}Br)N_2O_2: 343.1021, found: 343.1020

Data for **31**:

mp: 92–94 °C (CH_2Cl_2)

^{1}H NMR: (400 MHz, $CDCl_3$)

7.59–7.44 (m, 2H, C(3′)*H*, C(3′′′)*H*), 7.30–7.21 (m, 4H, C(4′)*H*, C(6′)*H*, C(4′′′)*H*, C(6′′′)*H*), 7.11–7.02 (m, 2H, C(5′)*H*, C(5′′′)*H*), 5.05 (s, 1H, N*H*), 3.39–3.12 (m, 2H, C(1)H_2), 2.96–2.72 (m, 4H, C(4)H_2, C(2′′)H_2), 1.82–1.56 (m, 4H, C(3)H_2, C(1′′)H_2), 1.49–1.41 (m, 11H, C(CH_3)$_3$, NH_2)

^{13}C NMR: (101 MHz, $CDCl_3$)

156.6 (*C*=O), 141.7 (*C*(1′), *C*(1′′′)), 133.0 (*C*(3′), *C*(3′′′)), 130.7 (*C*(6′), *C*(6′′′)), 127.9 (*C*(5′), *C*(5′′′)), 127.9 (*C*(4′), *C*(4′′′)), 124.4 (*C*(2′), *C*(2′′′)), 79.4 (*C*(CH_3)$_3$), 54.8 (*C*(2)), 48.5 (*C*(1)), 38.2 (*C*(3), *C*(1′′)), 30.7 (*C*(4), *C*(2′′)), 28.6 (C(*C*H_3)$_3$)

IR: (neat)

3226 (w), 3056 (w), 2974 (w), 2929 (w), 2868 (w), 1686 (m), 1567 (w), 1535 (w), 1509 (w), 1472 (m), 1452 (w), 1439 (w), 1390 (w), 1365 (m), 1276 (m), 1250 (m), 1166 (m), 1043 (w), 1023 (m), 965 (w), 943 (w), 896 (w), 859 (w), 743 (m), 722 (w), 701 (w), 659 (w)

MS: (ESI$^+$)

530 (13%), 529 ([M(^{81}Br)(^{81}Br)+H]$^+$, 51%), 528 (26%), 527 ([M(^{79}Br)(^{81}Br)+H]$^+$, 100%), 526 (12%), 525 ([M(^{79}Br)(^{79}Br)+H]$^+$, 51%), 282 (36%)

HRMS: (ESI$^+$)

Calcd for $C_{23}H_{31}$(^{79}Br)(^{81}Br)N_2O_2: 527.0732, found: 527.0730

Preparation of (*RS*)-3-(2-bromophenyl)-1-(trimethylsilyl)propan-1-amine (7tc), 3-(2-bromophenyl)propan-1-amine (32) and 1,5-bis(2-bromophenyl)pentan-3-amine (33)

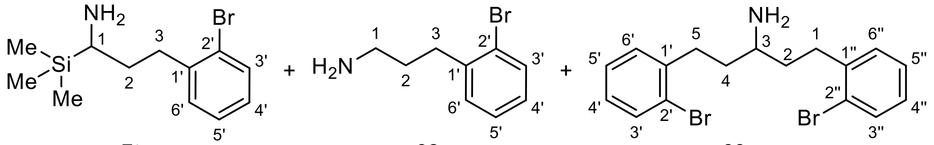

7tc **32** **33**

Following ***General Procedure 1***, (trimethylsilyl)methylamine (**1t**) (181 μL, 139 mg, 1.35 mmol, 3.0 equiv), 2-bromostyrene (**6c**) (57 μL, 83 mg, 0.45 mmol, 1.0 equiv), 3DPA2FBN (2.80 mM in DMF, 1.60 mL, 4.5 μmol, 1 mol%), tetrabutylammonium azide (70.3 mM in DMF, 1.28 mL, 90 μmol, 20 mol%) and DMF (120 μL) were reacted for 20 h with a 425 nm LED lamp. The mixture was concentrated *in vacuo* and purified *via* flash column chromatography on silica gel (12 g) in CH_2Cl_2 (5 CV) then 100:0:0→95:4.5:0.5 CH_2Cl_2–MeOH–aq. NH_4OH (over 20 CV) then 95:4.5:0.5 CH_2Cl_2–MeOH–aq. NH_4OH (5 CV) to give **7tc** as a yellow oil (49.8 mg, 39%) and an inseparable 44:56 mixture of **32** to Bu_4NN_3 as a yellow oil [13.2 mg, containing 4.6 mg of **32** (5%)].

A repeat of the above reaction in DMF-d_7 allowed us to measure the following product distribution, with yields calculated using the Bu_4N^+ ion as an internal standard: **7tc** (66%), **1t** (54%) and **6c** (6%).

The reaction was also repeated with (trimethylsilyl)methylamine (**1t**) (60 μL, 47 mg, 0.45 mmol, 1.0 equiv) to give **33** as a yellow oil (18.2 mg, 10% wrt **1t**).

Data for **7tc**:

^{1}H NMR: (400 MHz, $CDCl_3$)

7.54–7.51 (m, 1H, C(3′)*H*), 7.25–7.22 (m, 2H, C(4′)*H*, C(6′)*H*), 7.07–7.03 (m, 1H, C(5′)*H*), 2.99 (ddd, J = 13.4, 10.7, 4.7 Hz, 1H, C(3)H_A), 2.71 (ddd, J = 13.4, 10.5, 6.0 Hz, 1H, C(3)H_B), 2.20 (dd, J = 10.5, 3.2 Hz, 1H, C(1)*H*), 1.85 (dddd,

J = 13.8, 10.7, 6.0, 3.2 Hz, 1H, C(2)H_A), 1.56 (dddd, *J* = 13.8, 10.5, 10.5, 4.7 Hz, 1H, C(2)H_B), 1.33 (br s, 2H, NH_2), 0.05 (s, 9H, Si(Me)$_3$)

^{13}C NMR: (101 MHz, $CDCl_3$)

141.9 (*C*(1′)), 133.0 (*C*(3′)), 130.5 (*C*(6′)), 127.7 (*C*(5′)), 127.6 (*C*(4′)), 124.6 (*C*(2′)), 41.5 (*C*(1)), 34.7 (*C*(2)), 34.3 (*C*(3)), -3.6 (Si(Me)$_3$)

IR: (neat)

3058 (w), 2951 (w), 2855 (w), 1667 (w), 1567 (w), 1471 (w), 1439 (w), 1340 (w), 1308 (w), 1247 (m), 1159 (w), 1072 (w), 1042 (w), 1022 (w), 837 (m), 749 (m), 698 (w), 657 (w)

MS: (ESI^+)

289 (17%), 288 ([M(^{81}Br)+H]$^+$, 100%), 287 (18%), 286 ([M(^{79}Br)+H]$^+$, 100%), 279 (15%), 278 (81%), 94 (10%), 141 (115), 126 (22%)

HRMS: (ESI^+)

Calcd for $C_{12}H_{21}$(^{81}Br)NSi: 288.0606, found: 288.0608

Data for **32**:

^{1}H NMR: (400 MHz, $CDCl_3$)

7.53–7.49 (m, 1H, C(3′)*H*), 7.24–7.20 (m, 2H, C(4′)*H*, C(6′)*H*), 7.06–7.02 (m, 1H, C(5′)*H*), 2.84–2.71 (m, 4H, C(1)H_2, C(3)H_2), 1.84–1.76 (m, 2H, C(2)H_2)

^{13}C NMR: (101 MHz, $CDCl_3$)

141.4 (*C*(1′)), 132.9 (*C*(3′)), 130.4 (*C*(6′)), 127.7 (*C*(5′)), 127.6 (*C*(4′)), 124.6 (*C*(2′)), 41.7 (*C*(1)), 33.6 (*C*(2) or *C*(3)), 33.5 (*C*(2) or *C*(3))

HRMS: (ESI^+)

Calcd for C_9H_{13}(^{79}Br)N: 214.0231, found: 214.0229

Data for **33**:

^{1}H NMR: (400 MHz, $CDCl_3$)

7.58–7.48 (m, 2H, C(3′)*H*, C(3″)*H*), 7.26–7.15 (m, 4H, C(4′)*H*, C(6′)*H*, C(4″)*H*, C(6″)*H*), 7.09–7.01 (m, 2H, C(5′)*H*, C(5″)*H*), 2.94–2.72 (m, 5H, C(1)H_2, C(5)H_2, C(3)*H*), 1.87–1.75 (m, 2H, C(2)H_A, C(4)H_A), 1.69–1.59 (m, 2H, C(2)H_B, C(4)H_B), 1.52 (br s, 2H, NH_2)

^{13}C NMR: (101 MHz, $CDCl_3$)

141.7 (*C*(1′), *C*(1″)), 133.0 (*C*(3′), *C*(3″)), 130.5 (*C*(6′), *C*(6″)), 127.7 (*C*(5′), *C*(5″)), 127.7 (*C*(4′), *C*(4″)), 124.5 (*C*(2′), *C*(2″)), 51.0 (*C*(3)), 38.4 (*C*(2), *C*(6)), 33.0 (*C*(1), *C*(5))

IR: (neat)

3053 (w), 2923 (w), 2859 (w), 1672 (w), 1591 (w), 1566 (w), 1470 (m), 1452 (w), 1438 (w), 1384 (w), 1159 (w), 1120 (w), 1044 (w), 1021 (m), 941 (w), 907 (w), 822 (w), 746 (m), 658 (w)

MS: (ESI$^+$)

400 ([M(^{81}Br)(^{81}Br)+H]$^+$, 50%), 399 (19%), 398 ([M(^{79}Br)(^{81}Br)+H]$^+$, 100%), 396 ([M(^{79}Br)(^{79}Br)+H]$^+$, 52%), 282 (19%)

HRMS: (ESI$^+$)

Calcd for $C_{17}H_{20}$(^{79}Br)(^{81}Br)N: 397.9942, found: 397.9942

Preparation of (*RS*)-4-(2-bromophenyl)-1-(thiophen-2-yl)butan-2-amine (7uc) and 1,5-bis(2-bromophenyl)-3-(thiophen-2-ylmethyl)pentan-3-amine (34)

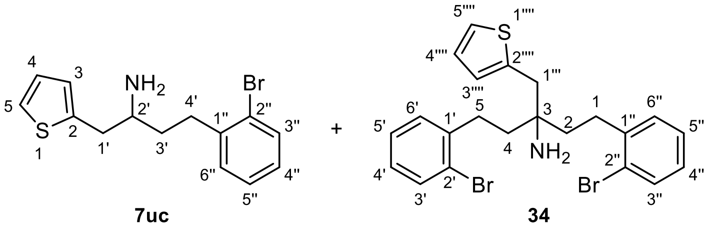

Following ***General Procedure 1***, 2-thiopheneethylamine (**1u**) (158 µL, 172 mg, 1.35 mmol, 3.0 equiv), 2-bromostyrene (**6c**) (57 µL, 83 mg, 0.45 mmol, 1.0 equiv), 3DPA2FBN (2.80 mM in DMF, 1.60 mL, 4.5 µmol, 1 mol%), tetrabutylammonium azide (70.3 mM in DMF, 1.28 mL, 90 µmol, 20 mol%) and DMF (120 µL) were reacted for 20 h with a 425 nm LED lamp. The mixture was concentrated *in vacuo* and purified *via* flash column chromatography on silica gel (12 g) in CH_2Cl_2 (5 CV) then 100:0:0→95:4.5:0.5 CH_2Cl_2–MeOH–aq. NH_4OH (over 20 CV) then 95:4.5:0.5 CH_2Cl_2–MeOH–aq. NH_4OH (5 CV), followed by reversed-phase flash column chromatography on C_{18} silica gel (15.5 g) in 98:2 H_2O–MeOH (5 CV) then 98:2→0:100 H_2O–MeOH (over 15 CV) then MeOH (5 CV) to give **7uc** as an orange oil (120.3 mg, 87%).

The reaction was repeated with 2-thiopheneethylamine (**1u**) (53 µL, 57 mg, 0.45 mmol, 1.0 equiv), to give **7uc** as an orange oil (44.5 mg, 32%) and **34** as an orange oil (19.6 mg, 9% wrt **1u**).

Data for **7uc**:

^{1}H NMR: (400 MHz, $CDCl_3$)

7.56–7.48 (m, 1H, C(3″)*H*), 7.26–7.19 (m, 2H, C(4″)*H*, C(6″)*H*), 7.16 (dd, *J* = 5.1, 1.2 Hz, 1H, C(5)*H*), 7.08–7.02 (m, 1H, C(5″)*H*), 6.95 (dd, *J* = 5.1, 3.4 Hz, 1H, C(4)*H*), 6.85 (dd, *J* = 3.4, 1.2 Hz, 1H, C(3)*H*), 3.14–3.00 (m, 2H, C(1′)H_A, C(2′)*H*), 2.97–2.88 (m, 1H, C(4′)H_B), 2.87–2.72 (m, 2H, C(1′)H_B, C(4′)H_B), 1.89–1.78 (m, 1H, C(3′)H_A), 1.70–1.60 (m, 1H, C(3′)H_B), 1.49 (br s, 2H, NH_2)

^{13}C NMR: (101 MHz, $CDCl_3$)

141.7 (*C*(2) or *C*(1″)), 141.5 (*C*(2) or *C*(1″)), 133.0 (*C*(3″)), 130.4 (*C*(6″)), 127.8 (*C*(5″)), 127.7 (*C*(4″)), 127.0 (*C*(4)), 126.0 (*C*(3)), 124.5 (*C*(2″)), 124.0 (*C*(5)), 52.8 (*C*(2′)), 38.8 (*C*(1′)), 37.7 (*C*(3′)), 33.1 (*C*(4′))

IR: (neat)

3066 (w), 2916 (w), 2854 (w), 1589 (w), 1566 (w), 1471 (w), 1438 (w), 1364 (w), 1244 (w), 1159 (w), 1128 (w), 1075 (w), 1044 (w), 1023 (w), 942 (w), 849 (w), 825 (w), 750 (m), 697 (m), 659 (w)

MS: (ESI^+)

618 (16%), 616 (29%), 613 (14%), 313 (16%), 312 ($[M(^{81}Br)+H]^+$, 100%), 311 (16%), 310 ($[M(^{79}Br)+H]^+$, 95%)

HRMS: (ESI^+)

Calcd for $C_{14}H_{17}(^{81}Br)NS$: 312.0245, found: 312.0246

Data for **34**:

^{1}H NMR: (400 MHz, $CDCl_3$)

7.58–7.46 (m, 2H, C(3′)*H*, C(3″)*H*), 7.29–7.21 (m, 4H, C(4′)*H*, C(6′)*H*, C(4″)*H*, C(6″)*H*), 7.21–7.18 (m, 1H, C(5″″)*H*), 7.09–7.03 (m, 2H, C(5′)*H*, C(5″)*H*), 7.01–6.97 (m, 1H, C(3″″)*H*), 6.95–6.91 (m, 1H, C(4″″)*H*), 3.07 (s, 2H, C(1‴)H_2), 2.98–2.85 (m, 4H, C(1)H_2, C(5)H_2), 1.89–1.65 (m, 4H, C(2)H_2, C(4)H_2), 1.55 (br s, 2H, NH_2)

^{13}C NMR: (101 MHz, $CDCl_3$)

141.8 (*C*(1′), *C*(1″)), 139.2 (*C*(2″″)), 133.0 (*C*(3′), *C*(3″)), 130.5 (*C*(6′), *C*(6″)), 127.8 (*C*(4′), *C*(5′), *C*(4″), *C*(5″)), 127.5 (*C*(4″″)), 126.9 (*C*(3″″)), 124.5 (*C*(2′), *C*(2″), *C*(5″″)), 54.3 (*C*(3)), 41.3 (*C*(1″″)), 39.8 (*C*(2), *C*(4)), 31.0 (*C*(1), *C*(5))

IR: (neat)

3065 (w), 2924 (w), 2863 (w), 1671 (w), 1590 (w), 1566 (w), 1495 (w), 1471 (m), 1452 (w), 1438 (w), 1260 (w), 1142 (w), 1078 (w), 1043 (w), 1022 (m), 908 (w), 850 (w), 823 (w), 747 (m), 731 (m), 696 (m), 659 (w)

MS: (ESI^+)

497 (12%), 496 ($[M(^{81}Br)(^{81}Br)+H]^+$, 53%), 495 (25%), 494 ($[M(^{79}Br)(^{81}Br)+H]^+$, 100%), 493 (12%), 492 ($[M(^{79}Br)(^{79}Br)+H]^+$, 49%), 283 (11%), 282 (56%), 126 (11%)

HRMS: (ESI^+)

Calcd for $C_{22}H_{24}(^{79}Br)(^{81}Br)NS$: 493.9976, found: 493.9974

Preparation of (*RS*)-1-(2-bromophenyl)-5-(1*H*-imidazol-1-yl)pentan-3-amine (7vc)

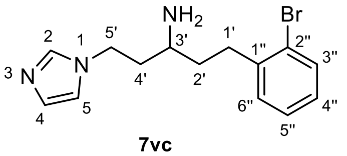

7vc

Following ***General Procedure 1***, 1-(3-aminopropyl)imidazole (**1v**) (161 µL, 169 mg, 1.35 mmol, 3.0 equiv), 2-bromostyrene (**6c**) (57 µL, 83 mg, 0.45 mmol, 1.0 equiv), 3DPA2FBN (2.80 mM in DMF, 1.60 mL, 4.5 µmol, 1 mol%), tetrabutylammonium azide (70.3 mM in DMF, 1.28 mL, 90 µmol, 20 mol%) and DMF (120 µL) were reacted for 20 h with a 425 nm LED lamp. The mixture was concentrated *in vacuo* and purified *via* flash column chromatography on silica gel (12 g) in CH_2Cl_2 (5 CV) then 100:0:0→95:4.5:0.5 CH_2Cl_2–MeOH–aq. NH_4OH (over 20 CV) then 95:4.5:0.5 CH_2Cl_2–MeOH–aq. NH_4OH (5 CV), followed by reversed-phase flash column chromatography on C_{18} silica gel (15.5 g) in 98:2 H_2O–MeOH (5 CV) then 98:2→0:100 H_2O–MeOH (over 15 CV) then MeOH (5 CV) to give **7vc** as a colourless oil (85.7 mg, 62%).

Data for **7vc**:

^1H NMR: (400 MHz, $CDCl_3$)

7.54–7.49 (m, 1H, C(3′)*H*), 7.47 (s, 1H, C(2)*H*), 7.24–7.14 (m, 2H, C(4″)*H*, C(6″)*H*), 7.07–7.01 (m, 2H, C(4)*H*, C(5″)*H*), 6.94–6.86 (m, 1H, C(5)*H*), 4.15–4.03 (m, 2H, C(5′)H_2), 2.85–2.77 (m, 1H, C(1′)H_A), 2.74–2.65 (m, 2H, C(1′)H_B, C(3′)*H*), 1.98–1.90 (m, 1H, C(4′)H_A), 1.77–1.65 (m, 2H, C(2′)H_A, C(4′)H_B), 1.62–1.53 (m, 1H, C(2′)H_B), 1.35 (br s, 2H, NH_2)

<u>^{13}C NMR:</u> (101 MHz, $CDCl_3$)

141.1 (*C*(1″)), 137.3 (*C*(2)), 133.0 (*C*(3″)), 130.4 (*C*(6″)), 129.6 (*C*(4)), 127.9 (*C*(5″)), 127.7 (*C*(4″)), 124.3 (*C*(2″)), 118.9 (*C*(5)), 48.5 (*C*(3′)), 44.3 (*C*(5′)), 39.3 (*C*(2′) or *C*(4′)), 39.0 (*C*(2′) or *C*(4′), 32.8 (*C*(1′))

<u>IR:</u> (neat)

3278 (w), 3108 (w), 2926 (w), 2862 (w), 1663 (w), 1588 (w), 1566 (w), 1508 (w), 1471 (w), 1453 (w), 1438 (w), 1366 (w), 1283 (w), 1229 (w), 1108 (w), 1079 (w), 1022 (w), 916 (w), 818 (w), 749 (m), 662 (w)

<u>MS:</u> (ESI$^+$)

311 (15%), 310 ([M(^{81}Br)+H]$^+$, 94%), 309 (16%), 308 ([M(^{79}Br)+H]$^+$, 100%), 171 (18%), 169 (19%), 156 (28%), 155 (29%), 123 (31%)

<u>HRMS:</u> (ESI$^+$)

Calcd for $C_{14}H_{19}(^{79}Br)N_3$: 308.0762, found: 308.0762

Preparation of (*RS*)-4-(2-bromophenyl)-1-(pyridin-2-yl)butan-2-amine (7wc) and 1,5-bis(2-bromophenyl)-3-(pyridin-2-ylmethyl)pentan-3-amine (35)

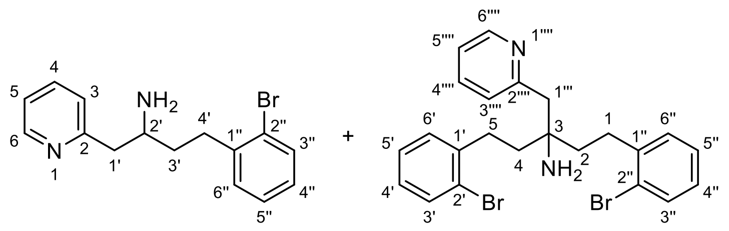

Following ***General Procedure 1***, 2-(2-pyridyl)ethylamine (**1w**) (162 µL, 165 mg, 1.35 mmol, 3.0 equiv), 2-bromostyrene (**6c**) (57 µL, 83 mg, 0.45 mmol, 1.0 equiv), 3DPA2FBN (2.80 mM in DMF, 1.60 mL, 4.5 µmol, 1 mol%), tetrabutylammonium azide (70.3 mM in DMF, 1.28 mL, 90 µmol, 20 mol%) and DMF (120 µL) were reacted for 20 h with a 425 nm LED lamp. The mixture was concentrated *in vacuo* and purified *via* flash column chromatography on silica gel (12 g) in CH_2Cl_2 (5 CV) then 100:0:0→95:4.5:0.5 CH_2Cl_2–MeOH–aq. NH_4OH (over 20 CV) then 95:4.5:0.5 CH_2Cl_2–MeOH–aq. NH_4OH (5 CV) to give **7wc** as an orange oil (68.4 mg, 50%).

The reaction was repeated with 2-(2-pyridyl)ethylamine (**1w**) (54 µL, 55 mg, 0.45 mmol, 1.0 equiv) to give **7wc** as an orange oil (34.0 mg, 25%) and **35** as an orange oil (35.0 mg, 16% wrt **1w**).

Data for **7wc**:

^{1}H NMR: (400 MHz, $CDCl_3$)

8.59–8.51 (m, 1H, C(2)*H*), 7.65–7.58 (m, 1H, C(4)*H*), 7.54–7.49 (m, 1H, C(3″)*H*), 7.25–7.19 (m, 2H, C(4″)*H*, C(6″)*H*), 7.19–7.12 (m, 2H, C(3)*H*, C(5)*H*), 7.07–7.02 (m, 1H, C(5″)*H*), 3.37–3.28 (m, 1H, C(2′)*H*), 3.05–2.99 (m, 1H, C(1′)H_A), 2.97–2.89 (m, 1H, C(4′)H_A), 2.86–2.77 (m, 1H, C(4′)H_B), 2.77–2.70 (m, 1H, C(1′)H_B), 1.88–1.79 (m, 1H, C(3′)H_A), 1.73–1.63 (m, 1H, C(3′)H_B), 1.52 (br s, 2H, NH_2)

^{13}C NMR: (101 MHz, $CDCl_3$)

160.0 (*C*(2)), 149.6 (*C*(6)), 141.7 (*C*(1″)), 136.5 (*C*(4)), 133.0 (*C*(3″)), 130.5 (*C*(6″)), 127.7 (*C*(5″)), 127.6 (*C*(4″)), 124.6 (*C*(2″)), 124.1 (*C*(3)), 121.5 (*C*(5)), 51.7 (*C*(2′)), 46.9 (*C*(1′)), 38.3 (*C*(3′)), 33.2 (*C*(4′))

IR: (neat)

3269 (w), 3056 (w), 3010 (w), 2921 (w), 2858 (w), 1676 (w), 1590 (w), 1568 (w), 1471 (m), 1435 (m), 1383 (w), 1309 (w), 1149 (w), 1098 (w), 1049 (w), 1022 (w), 995 (w), 942 (w), 838 (w), 748 (m), 659 (w)

MS: (ESI^+)

308 (15%), 307 ([M(^{81}Br)+H]$^+$, 97%), 306 (17%), 305 ([M(^{79}Br)+H]$^+$, 100%)

HRMS: (ESI^+)

Calcd for $C_{15}H_{18}(^{79}Br)N_2$: 305.0653, found: 305.0652

Data for **35**:

^{1}H NMR: (400 MHz, $CDCl_3$)

8.58–8.55 (m, 1H, C(6⁗)*H*), 7.62–7.57 (m, 1H, C(4⁗)*H*), 7.52–7.47 (m, 2H, C(3′)*H*, C(3″)*H*), 7.26–7.19 (m, 5H, C(4′)*H*, C(4″)*H*, C(6′)*H*, C(6″)*H* C(3⁗)*H*), 7.15–7.12 (m, 1H, C(4⁗)*H*), 7.05–7.00 (m, 2H, C(5′)*H*, C(5″)*H*), 3.02 (s, 2H, C(1‴)H_2), 2.97–2.87 (m, 4H, C(1)H_2, C(5)H_2), 1.83 (br s, 2H, NH_2), 1.78–1.69 (m, 4H, C(2)H_2, C(4)H_2)

^{13}C NMR: (101 MHz, $CDCl_3$)

159.0 (*C*(2⁗)), 149.3 (*C*(6⁗)), 142.1 (*C*(1′), *C*(1″)), 136.2 (*C*(4‴)), 132.9 (*C*(3′), *C*(3″)), 130.6 (*C*(6′), *C*(6″)), 127.7 (*C*(5′), *C*(5″)), 127.6 (*C*(4′), *C*(4″)), 125.2 (*C*(3⁗)), 124.5 (*C*(2′), *C*(2″)), 121.5 (*C*(5⁗)), 55.1 (*C*(3)), 48.4 (*C*(1‴)), 40.4 (*C*(2), *C*(4)), 31.0 (*C*(1), *C*(5))

<u>IR:</u> (neat)

3053 (w), 2932 (w), 2859 (w), 1590 (w), 1567 (w), 1471 (m), 1453 (w), 1436 (w), 1368 (w), 1292 (w), 1149 (w), 1125 (w), 1044 (w), 1022 (m), 995 (w), 908 (w), 860 (w), 795 (w), 749 (m), 660 (w)

<u>MS:</u> (ESI^+)

492 (12%), 491 ($[M(^{81}Br)(^{81}Br)+H]^+$, 50%), 490 (26%), 489 ($[M(^{79}Br)(^{81}Br)+H]^+$, 100%), 488 (13%), 487 ($[M(^{79}Br)(^{79}Br)+H]^+$, 49%), 282 (16%)

<u>HRMS:</u> (ESI^+)

Calcd for $C_{23}H_{25}(^{79}Br)(^{81}Br)N_2$: 489.0364, found: 489.0364

Preparation of (*RS*)-4-(2-bromophenyl)-1-(2,2-dimethylbenzo[*d*][1,3]dioxol-5-yl)butan-2-amine (7xc)

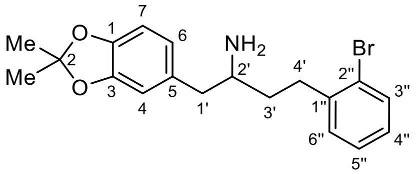

7xc

Following ***General Procedure 1***, 2,2-dimethyl-1,3-benzodioxole-5-ethanamine (**1x**) (87 mg, 0.45 mmol, 1.0 equiv), 2-bromostyrene (**6c**) (57 µL, 83 mg, 0.45 mmol, 1.0 equiv), 3DPA2FBN (2.80 mM in DMF, 1.60 mL, 4.5 µmol, 1 mol%), tetrabutylammonium azide (70.3 mM in DMF, 1.28 mL, 90 µmol, 20 mol%) and DMF (120 µL) were reacted for 20 h with a 425 nm LED lamp. The mixture was concentrated *in vacuo* and purified *via* flash column chromatography on silica gel (12 g) in CH_2Cl_2 (5 CV) then 100:0:0→95:4.5:0.5 CH_2Cl_2–MeOH–aq. NH_4OH (over 20 CV) then 95:4.5:0.5 CH_2Cl_2–MeOH–aq. NH_4OH (5 CV) to give **7xc** as an orange oil (92.0 mg, 55%).

<u>Data for **7xc**:</u>

<u>^{1}H NMR:</u> (400 MHz, $CDCl_3$)

7.54–7.50 (m, 1H, C(3″)*H*), 7.25–7.19 (m, 2H, C(4″)*H*, C(6″)*H*), 7.08–7.03 (m, 1H, C(5″)*H*), 6.69–6.62 (m, 1H, C(7)*H*), 6.62–6.54 (m, 2H, C(4)*H*, C(6)*H*), 3.02–2.96 (m, 1H, C(2′)*H*), 2.95–2.87 (m, 1H, C(4′)H_A), 2.84–2.74 (m, 2H, C(1′)H_A, C(4′)H_B), 2.45–2.37 (m, 1H, C(1′)H_B), 1.85–1.76 (m, 1H, C(3′)H_A), 1.69–1.59 (m, 7H, C(2)Me_2, C(3′)H_B), 1.36 (br s, 2H, NH_2)

^{13}C NMR: (101 MHz, $CDCl_3$)

147.7 (*C*(1)), 146.0 (*C*(3)), 141.7 (*C*(1″)), 133.0 (*C*(3″)), 132.6 (*C*(5)), 130.4 (*C*(6″)), 127.7 (*C*(5″)), 127.6 (*C*(4″)), 124.6 (*C*(2″)), 121.7 (*C*(6)), 117.8 (*C*(2)), 109.4 (*C*(4)), 108.1 (*C*(7)), 52.7 (*C*(2′)), 44.5 (*C*(1′)), 38.0 (*C*(3′)), 33.2 (*C*(4′)), 26.0 (C(2)*Me*$_2$)

IR: (neat)

3054 (w), 2990 (w), 2929 (w), 2856 (w), 1671 (w), 1590 (w), 1567 (w), 1494 (m), 1471 (w), 1440 (m), 1376 (w), 1253 (m), 1233 (m), 1158 (w), 1119 (w), 1045 (w), 1022 (w), 980 (m), 940 (w), 909 (w), 837 (m), 802 (w), 750 (m), 732 (m), 659 (w)

MS: (ESI$^+$)

379 (21%), 378 ([M(^{81}Br)+H]$^+$, 100%), 377 (22%), 376 ([M(^{79}Br)+H]$^+$, 97%), 318 (12%), 238 (31%), 126 (15%)

HRMS: (ESI$^+$)

Calcd for $C_{19}H_{23}$(^{81}Br)NO_2: 378.0892, found: 378.0893

Preparation of *tert*-butyl (*RS*)-3-(2-amino-4-(2-bromophenyl)butyl)-1*H*-indole-1-carboxylate (7yc)

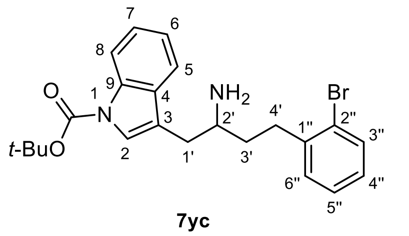

7yc

Following ***General Procedure 1***, 1-Boc-tryptamine (**1y**) (117 mg, 0.45 mmol, 1.0 equiv), 2-bromostyrene (**6c**) (57 µL, 83 mg, 0.45 mmol, 1.0 equiv), 3DPA2FBN (2.80 mM in DMF, 1.60 mL, 4.5 µmol, 1 mol%), tetrabutylammonium azide (70.3 mM in DMF, 1.28 mL, 90 µmol, 20 mol%) and DMF (120 µL) were reacted for 20 h with a 425 nm LED lamp. The mixture was concentrated *in vacuo* and purified *via* flash column chromatography on silica gel (12 g) in CH_2Cl_2 (5 CV) then 100:0:0→95:4.5:0.5 CH_2Cl_2–MeOH–aq. NH_4OH (over 20 CV) then 95:4.5:0.5 CH_2Cl_2–MeOH–aq. NH_4OH (5 CV) to give **7yc** as an orange oil (39.0 mg, 20%).

Data for **7yc**:

^{1}H NMR: (400 MHz, $CDCl_3$)

8.28–8.02 (m, 1H, C(8)*H*), 7.55–7.52 (m, 2H, C(5)*H*, C(3″)*H*), 7.46 (s, 1H, C(2)*H*), 7.34–7.30 (m, 1H, C(7)*H*), 7.25–7.21 (m, 3H, C(6)*H*, C(4″)*H*, C(6″)*H*), 7.08–7.04 (m, 1H, C(5″)*H*), 3.25–3.18 (m, 1H, C(2′)*H*), 2.99–2.92 (m, 2H, C(1′)H_A, C(4′)H_A), 2.89–2.81 (m, 1H, C(4′)H_B), 2.66–2.60 (m, 1H, C(1′)H_B), 1.93–1.83 (m, 1H, C(3′)H_A), 1.77–1.69 (m, 1H, C(3′)H_B), 1.67 (s, 9H, C(CH_3)$_3$), 1.41 (br s, 2H, NH_2)

^{13}C NMR: (101 MHz, $CDCl_3$)

149.9 (*C*=O), 141.6 (*C*(1″)), 135.8 (*C*(9)), 133.0 (*C*(3″)), 130.9 (*C*(4)), 130.4 (*C*(6″)), 127.8 (*C*(5″)), 127.7 (*C*(4″)), 124.6 (*C*(2″)), 124.5 (*C*(7)), 123.8 (*C*(2)), 122.6 (*C*(6)), 119.3 (*C*(5)), 118.1 (*C*(3)), 115.4 (*C*(8)), 83.6 (*C*(CH_3)$_3$), 50.9 (*C*(2′)), 38.3 (*C*(3′)), 34.1 (*C*(1′)), 33.3 (*C*(4′)), 28.4 (C(*C*H_3)$_3$)

IR: (neat)

2928 (w), 2352 (w), 2105 (w), 1727 (w), 1567 (w), 1472 (w), 1452 (m), 1367 (m), 1308 (w), 1254 (m), 1225 (w), 1154 (m), 1083 (m), 1020 (m), 907 (w), 855 (w), 745 (m), 728 (m), 668 (w), 658 (w)

MS: (ESI^+)

446 (27%), 445 ([M(^{81}Br)+H]$^+$, 100%), 444 (26%), 443 ([M(^{79}Br)+H]$^+$, 100%), 142 (18%)

HRMS: (ESI^+)

Calcd for $C_{23}H_{28}(^{79}Br)N_2O_2$: 443.1329, found: 443.1333

Preparation of *tert*-butyl 6-(2-bromophenyl)-4-((*tert*-butoxycarbonyl)amino)-3-(4-chlorophenyl)hexanoate (36), 5-(2-bromophenethyl)-4-(4-chlorophenyl)pyrrolidin-2-one (37) and *tert*-butyl (*RS*)-4-amino-4-(2-bromophenethyl)-6-(2-bromophenyl)-3-(4-chlorophenyl)hexanoate (38)

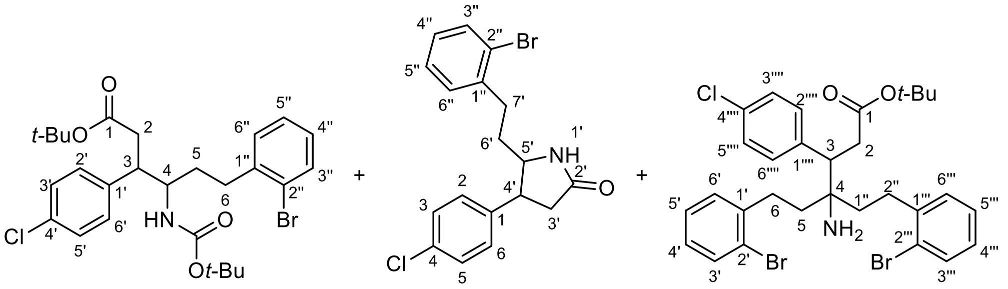

36 **37** **38**

Following ***General Procedure 1***, *tert*-butyl 4-amino-3-(4-chlorophenyl)butanoate (**1z**) (364 mg, 1.35 mmol, 3.0 equiv), 2-bromostyrene (**6c**) (57 µL, 83 mg, 0.45 mmol, 1.0 equiv), 3DPA2FBN (2.80 mM in DMF, 1.60 mL, 4.5 µmol, 1 mol%), tetrabutylammonium azide (70.3 mM in DMF, 1.28 mL, 90 µmol, 20 mol%) and DMF (120 µL) were reacted for 20 h with a 425 nm LED lamp. The mixture was concentrated *in vacuo* and purified *via* flash column chromatography on silica gel (12 g) in CH_2Cl_2 (5 CV) then 100:0:0→95:4.5:0.5 CH_2Cl_2–MeOH–aq. NH_4OH (over 20 CV) then 95:4.5:0.5 CH_2Cl_2–MeOH–aq. NH_4OH (5 CV), followed by reversed-phase flash column chromatography on C_{18} silica gel (15.5 g) in 98:2 H_2O–MeOH (5 CV) then 98:2→0:100 H_2O–MeOH (over 15 CV) then MeOH (5 CV) to give a mixture of **36** and **37**. Di-*tert*-butyl dicarbonate (72 mg, 0.33 mmol) was added to the mixture in CH_2Cl_2 (1 mL) and the resultant solution was stirred at rt for 3 h, followed by concentration *in vacuo*. The crude material was purified via flash column chromatography on silica gel (4 g) in CH_2Cl_2 (5 CV) then 100:0:0→95:4.5:0.5 CH_2Cl_2–MeOH–aq. NH_4OH (over 20 CV) then 95:4.5:0.5 CH_2Cl_2–MeOH–aq. NH_4OH (5 CV) to give **36** as a yellow oil (88.1 mg, 36%, 61:39 dr), **37** as a yellow oil (18.9 mg, 11%) and **38** as a yellow oil (49.9 mg, 18% wrt **6c**).

The reaction was repeated with *tert*-butyl 4-amino-3-(4-chlorophenyl)butanoate (**1z**) (121 mg, 0.45 mmol, 1.0 equiv) to give **38** as a yellow oil (96 mg, 34% wrt **1z**).

Data for **36**:

^{1}H NMR: (400 MHz, $CDCl_3$)

7.52–7.47 (m, 1H, C(3″)*H* for diastereomer 1), 7.47–7.44 (m, 1H, C(3″)*H* for diastereomer 2), 7.28–7.23 (m, 4H, C(3′)*H*, C(5′)*H* for both diastereomers), 7.20–7.00 (m, 10H, C(2′)*H*, C(6′)*H*, C(4″)*H*, C(5″)*H*, C(6″)*H*, for both

diastereomers), 4.55–4.46 (m, 1H, N*H* for diastereomer 2), 4.26–4.13 (m, 1H, N*H* for diastereomer 1), 4.00–3.88 (m, 1H, C(4)*H* for diastereomer 1), 3.88–3.79 (m, 1H, C(4)*H* for diastereomer 2), 3.32–3.20 (m, 1H, C(3)*H* for diastereomer 1), 3.12–3.05 (m, 1H, C(3)*H* for diastereomer 2), 2.82–2.68 (m, 5H, C(2)H_A, C(6)H_A for diastereomer 2 and C(2)H_A, C(6)H_A, C(6)H_B for diastereomer 1), 2.63–2.54 (m, 2H, C(6)H_B for diastereomer 2 and C(2)H_B for diastereomer 1), 2.49–2.42 (m, 1H, C(2)H_B for diastereomer 2), 1.83–1.74 (m, 1H, C(5)*H* for diastereomer 1), 1.72–1.65 (m, 1H, C(5)*H* for diastereomer 2), 1.49–1.43 (m, 18H, OC(CH_3)$_3$ for both diastereomers), 1.30–1.26 (m, 18H, C(1)OC(CH_3)$_3$ for both diastereomers)

^{13}C NMR: (101 MHz, $CDCl_3$)

172.0 (*C*(1) for diastereomer 2), 171.2 (*C*(1) for diastereomer 1), 156.1 (*C*=O for diastereomer 2) 155.9 (*C*=O for diastereomer 1), 140.9 (*C*(1″) for diastereomer 2), 140.2 (*C*(1″) for diastereomer 1), 138.2 (br s, *C*(1′) for both diastereomers), 133.0 (*C*(4′) for both diastereomers), 132.9 (*C*(3″) for diastereomer 2), 132.7 (*C*(3″) for diastereomer 1), 130.7 (*C*(6″) for diastereomer 1), 130.5 (*C*(6″) for diastereomer 2), 130.2 (*C*(6′) for diastereomer 1), 129.7 (*C*(6′) for diastereomer 2), 128.8 (*C*(5′) for diastereomer 2), 128.6 (*C*(5′) for diastereomer 1), 127.9 (*C*(5″) for diastereomer 1), 127.8 (*C*(5″) for diastereomer 2), 127.6 (*C*(4″) for diastereomer 1), 127.6 (*C*(4″) for diastereomer 2), 124.4 (*C*(2″) for both diastereomers), 80.8 (C(1)O*C*(CH_3)$_3$ for both diastereomers), 79.7 (O*C*(CH_3)$_3$ for both diastereomers), 55.0 (*C*(4) for diastereomer 2), 53.7 (*C*(4) for diastereomer 1), 47.0 (*C*(3) for diastereomer 2), 46.2 (*C*(3) for diastereomer 1), 39.8 (*C*(2) for diastereomer 2), 38.4 (*C*(2) for diastereomer 1), 34.2 (*C*(5) for diastereomer 1), 33.7 (*C*(5) for diastereomer 2), 33.2 (*C*(6) for diastereomer 1), 32.9 (*C*(6) for diastereomer 2), 28.6 (O*C*(CH_3)$_3$ for diastereomer 2), 28.5 (O*C*(CH_3)$_3$ for diastereomer 1), 28.1 (*C*(1)O*C*(CH_3)$_3$ for diastereomer 2), 28.0 (*C*(1)O*C*(CH_3)$_3$ for diastereomer 1)

IR: (neat)

3358 (w), 2978 (w), 2931 (w), 1701 (w), 1492 (w), 1472 (w), 1454 (w), 1413 (w), 1391 (w), 1366 (w), 1247 (w), 1165 (w), 1148 (w), 1107 (w), 1092 (w), 1045 (w), 1015 (w), 953 (w), 909 (w), 828 (w), 750 (w), 733 (w), 660 (w)

MS: (ESI^+)

578 (26%), 577 (29%), 576 ([M(^{81}Br)+Na]$^+$, 100%), 575 (22%), 574 ([M(^{79}Br)+Na]$^+$,77%), 444 (23%), 442 (91%), 440 (70%), 398 (55%), 396 (41%)

HRMS: (ESI$^+$)
Calcd for $C_{27}H_{35}$(^{81}Br)ClNNaO_4: 576.1315, found: 576.1312

Data for **37**:

^{1}H NMR: (400 MHz, $CDCl_3$)
7.56–7.49 (m, 1H, C(3″)*H*), 7.35–7.29 (m, 2H, C(2)*H,* C(6)*H*), 7.24–7.18 (m, 3H, C(3)*H,* C(5)*H,* C(4″)*H*), 7.14–7.11 (m, 1H, C(6″)*H*), 7.10–7.05 (m, 1H, C(5″)*H*), 6.05 (s, 1H, N*H*), 3.72–3.65 (m, 1H, C(5′)*H*), 3.28–3.20 (m, 1H, C(4′)*H*), 2.86–2.70 (m, 3H, C(3′)H_A, C(7′)H_2), 2.51–2.43 (m, 1H, C(3′)H_B), 1.97–1.80 (m, 2H, C(6′)H_2)

^{13}C NMR: (101 MHz, $CDCl_3$)
176.1 (*C*(2′)), 140.4 (*C*(1)), 140.1 (*C*(1″)), 133.2 (*C*(4)), 133.2 (*C*(3″)), 130.4 (*C*(6″)), 129.3 (*C*(2), *C*(6)), 128.8 (*C*(3), *C*(5)), 128.3 (*C*(5″)), 127.9 (*C*(4″)), 124.4 (*C*(2″)), 61.7 (*C*(5′)), 46.8 (*C*(4′)), 39.1 (*C*(3′)), 35.8 (*C*(6′)), 33.1 (*C*(7′))

IR: (neat)
3201 (w), 3098 (w), 2929 (w), 1693 (m), 1493 (w), 1472 (w), 1439 (w), 1411 (w), 1378 (w), 1339 (w), 1305 (w), 1287 (w), 1157 (w), 1091 (w), 1014 (w), 909 (w), 827 (w), 752 (w), 733 (w)

MS: (ESI$^+$)
759 (29%), 757 (31%), 591 (22%), 432 (47%), 402 ([M(^{81}Br)+Na]$^+$, 65%), 400 ([M(^{79}Br)+Na]$^+$, 49%), 380 ([M(^{81}Br)+H]$^+$, 82%), 378 ([M(^{79}Br)+H]$^+$, 65%), 282 (73%), 262 (100%)

HRMS: (ESI$^+$)
Calcd for $C_{18}H_{17}$(^{81}Br)ClNNaO: 402.0056, found: 402.0056

Data for **38**:

^{1}H NMR: (400 MHz, $CDCl_3$)
7.59–7.52 (m, 1H, C(3′)*H* or C(3‴)*H*), 7.52–7.45 (m, 1H, C(3′)*H* or C(3‴)*H*), 7.31–7.19 (m, 7H, C(4′)*H*, C(6′)*H* or C(6‴)*H*, C(4‴)*H*, C(2⁗)*H*, C(3⁗)*H*, C(5⁗)*H*, C(6⁗)*H*), 7.14–7.02 (m, 3H, C(5′)*H*, C(5‴)*H*, C(6′)*H* or C(6‴)*H*),

3.38–3.22 (m, 1H, C(3)*H*), 3.01–2.60 (m, 6H, C(2)H_2, C(6)H_2, C(2′′)H_2), 1.89–1.76 (m, 1H, C(5)*H* or C(1′′)*H*), 1.76–1.61 (m, 2H, C(5)*H*, C(1′′)*H*), 1.49–1.43 (m, 1H, C(5)*H* or C(1′′)*H*), 1.18 (s, 9H, C(1)C(CH_3)$_3$)

^{13}C NMR: (101 MHz, $CDCl_3$)

171.9 (*C*(1)), 141.7 (*C*(1′) or *C*(1′′′)), 141.6 (*C*(1′) or *C*(1′′′)), 138.8 (*C*(1′′′′)), 133.1 (*C*(3′) or *C*(3′′′)), 133.0 (*C*(3′) or *C*(3′′′)), 132.8 (*C*(4′′′′)), 131.4 (*C*(2′′′′) or *C*(6′′′′)), 130.5 (*C*(6′) or *C*(6′′′)), 130.5 (*C*(6′) or *C*(6′′′)), 128.2 (*C*(3′′′′) or *C*(5′′′′)), 127.9 (*C*(5′) or *C*(5′′′)), 127.9 (*C*(5′) or *C*(5′′′)), 127.8 (*C*(4′), *C*(4′′′)), 124.5 (*C*(2′) or *C*(2′′′)), 124.4 (*C*(2′) or *C*(2′′′)), 80.5 (C(1)*C*(CH_3)$_3$), 55.9 (*C*(4)), 49.3 (*C*(3)), 38.7 (*C*(5) or *C*(1′′)), 37.8 (*C*(5) or *C*(1′′)), 36.7 (*C*(2)), 30.7 (*C*(6) or *C*(2′′)), 30.6 (*C*(6) or *C*(2′′)), 27.9 (C(1)C(*C*H_3)$_3$)

IR: (neat)

2976 (w), 2931 (w), 1719 (m), 1491 (w), 1471 (w), 1455 (w), 1439 (w), 1413 (w), 1392 (w), 1367 (w), 1286 (w), 1256 (w), 1146 (m), 1108 (w), 1093 (w), 1044 (w), 1023 (w), 1015 (w), 959 (w), 908 (w), 829 (w), 751 (m), 732 (m), 660 (w)

MS: (ESI$^+$)

702 (22%), 700 (13%), 640 (16%), 639 (22%), 638 ([M(^{81}Br)(^{81}Br)+H]$^+$, 72%), 637 (33%), 636 ([M(^{79}Br)(^{81}Br)+H]$^+$, 100%), 635 (14%), 634 ([M(^{79}Br)(^{79}Br)+H]$^+$, 42%), 282 (19%)

HRMS: (ESI$^+$)

Calcd for $C_{30}H_{35}$(^{79}Br)(^{81}Br)$ClNO_2$: 636.0703, found: 636.0701

Preparation of ethyl (3*R*,4*R*,5*R*)-4-acetamido-5-amino-5-(2-bromophenethyl)-3-(pentan-3-yloxy)cyclohex-1-ene-1-carboxylate (7aac)

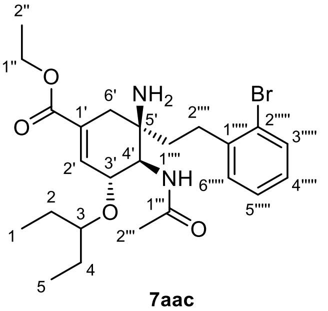

7aac

Following ***General Procedure 1***, Oseltamivir (**1aa**) (141 mg, 0.45 mmol, 1.0 equiv), 2-bromostyrene (**6c**) (57 μL, 83 mg, 0.45 mmol, 1.0 equiv), 3DPA2FBN (2.80 mM in DMF,

1.60 mL, 4.5 μmol, 1 mol%), tetrabutylammonium azide (70.3 mM in DMF, 1.28 mL, 90 μmol, 20 mol%) and DMF (120 μL) were reacted for 20 h with a 425 nm LED lamp. The mixture was concentrated *in vacuo* and purified *via* flash column chromatography on silica gel (12 g) in CH_2Cl_2 (5 CV) then 100:0:0→95:4.5:0.5 CH_2Cl_2–MeOH–aq. NH_4OH (over 20 CV) then 95:4.5:0.5 CH_2Cl_2–MeOH–aq. NH_4OH (5 CV) to give **7aac** as a yellow oil (25.1 mg, 11%). The remainder of the mass balance comprised a mixture of unidentified products.

Data for **7aac**:

^{1}H NMR: (400 MHz, $CDCl_3$)
7.51–7.47 (m, 1H, C(3′′′′′)*H*), 7.22–7.18 (m, 2H, C(4′′′′′)*H*, C(6′′′′′)*H*), 7.06–7.02 (m, 1H, C(5′′′′′)*H*), 6.92–6.87 (m, 1H, C(2′)*H*), 5.96–5.85 (m, 1H, N*H*), 4.23 (q, *J* = 7.1 Hz, 2H, C(1′′)H_2), 4.08–4.00 (m, 1H, C(4′)*H*), 3.99–3.94 (m, 1H, C(3′)*H*), 3.36–3.30 (m, 1H, C(3)*H*), 2.84–2.73 (m, 2H, C(2′′′)H_2), 2.71–2.65 (m, 1H, C(6′)H_A), 2.49–2.41 (m, 1H, C(6′)H_B), 2.02 (s, 3H, C(2′′′)H_3), 1.75–1.68 (m, 2H, C(1′′′)H_2), 1.59–1.46 (m, 6H, C(2)H_2, C(4)H_2, NH_2), 1.31 (t, *J* = 7.1 Hz, 3H, C(2′′)H_3), 0.93–0.85 (m, 6H, C(1)H_3, C(5)H_3)

^{13}C NMR: (101 MHz, $CDCl_3$)
170.2 (*C*(1′′′)), 166.8 (*C*(O)OEt), 141.2 (*C*(1′′′′′)), 137.7 (*C*(2′)), 132.9 (*C*(3′′′′′)), 130.7 (*C*(6′′′′′)), 128.5 (*C*(1′)), 127.9 (*C*(5′′′′′)), 127.9 (*C*(4′′′′′)), 124.3 (*C*(2′′′′′)), 82.4 (*C*(3)), 75.8 (*C*(3′)), 61.1 (*C*(1′′)), 55.5 (*C*(4′)), 54.8 (*C*(5′)), 39.8 (*C*(1′′′′)), 37.4 (*C*(6′)), 30.2 (*C*(2′′′′)), 26.2 (*C*(2) or *C*(4)), 25.8 (*C*(2) or *C*(4)), 23.8 (*C*(2′′′)), 14.4 (*C*(2′′)), 9.7 (*C*(1) or *C*(5)), 9.3 (*C*(1) or *C*(5))

IR: (neat)
3285 (w), 3071 (w), 2964 (w), 2933 (w), 2876 (w), 1710 (w), 1648 (m), 1551 (w), 1470 (w), 1439 (w), 1371 (w), 1295 (w), 1240 (m), 1172 (w), 1098 (w), 1058 (w), 1023 (w), 959 (w), 909 (w), 863 (w), 822 (w), 750 (w), 731 (m)

MS: (ESI^+)
498 (26%), 497 ($[M(^{81}Br)+H]^+$, 100%), 496 (27%), 495 ($[M(^{79}Br)+H]^+$, 100%), 299 (17%), 271 (17%), 125 (21%)

HRMS: (ESI^+)
Calcd for $C_{24}H_{36}(^{81}Br)N_2O_4$: 497.1838, found: 497.1841

D.2. Gram-Scale Hydroaminoalkylation in Continuous Flow

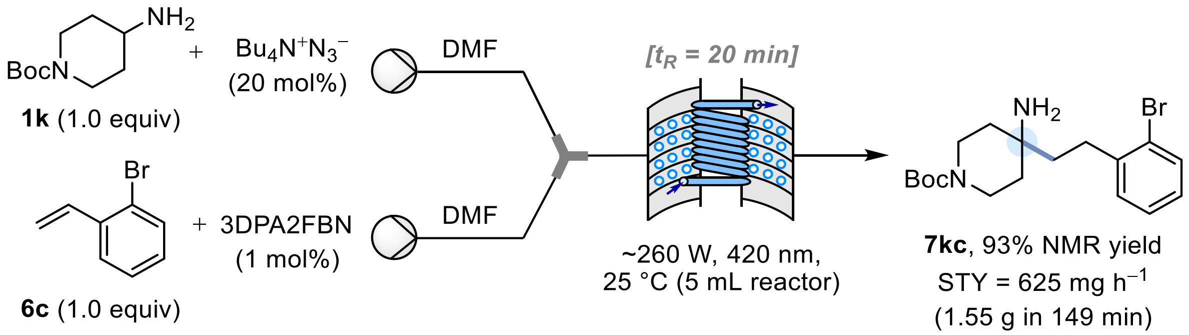

The following solutions were prepared in 25-mL volumetric flasks under N_2:

Reagent feed A: A 25-mL solution of 2-bromostyrene (**6c**) (940 mL, 1.37 g, 7.5 mmol, 1.0 equiv) and 3DPA2FBN (48 mg, 0.075 mmol, 1 mol%) in anhydrous DMF.

Reagent feed B: A 25-mL solution of *tert*-butyl 4-amino-1-piperidinecarboxylate (**1k**) (1.50 g, 7.5 mmol, 1.0 equiv) and tetrabutylammonium azide (428 mg, 1.5 mmol, 20 mol%) in anhydrous DMF.

A Vapourtec E-series flow reactor equipped with a Uniqsis cold coil tubing module and a PhotoSyn HP LED photoreactor with a water-cooled 420 nm LED array (set to ~260 W radiant output power) was used. After priming the reagent lines for feeds A and B, and flushing the system with anhydrous DMF, 15-mL portions of feeds A and B were injected simultaneously into the photoreactor at a flow rate of 0.125 mL min^{-1} (residence time: 20 min), mixed in a T-mixer and passed through a 5-mL coil (1.0 mm inner diameter, fluoropolymer tube), irradiated with a 420 nm LED array (~260 W radiant output power) at 25 °C. The pressure was kept below 8 bar using a back-pressure regulator (BPR). After the entire 30-mL mixture had entered the reactor, it was followed with anhydrous DMF at the same flow rate. The steady-state product mixture was collected in a 250-mL, round-bottomed flask as a yellow solution (28.7 mL), and was then concentrated *in vacuo*. Purification *via* flash column chromatography on silica gel (12 g) in CH_2Cl_2 (5 CV) then 100:0:0→95:4.5:0.5 CH_2Cl_2–MeOH–aq. NH_4OH (over 20 CV) then 95:4.5:0.5 CH_2Cl_2–MeOH–aq. NH_4OH (5 CV) gave **7kc** as a yellow oil (1.55 g, steady-state STY = 625 mg h^{-1}).

D.3. Reaction Generality: Styrene Scope

Preparation of 1-phenethylcyclohexan-1-amine (7ab)

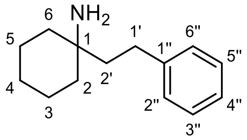

7ab

Following ***General Procedure 1***, cyclohexylamine (**1a**) (52 µL, 45 mg, 0.45 mmol, 1.0 equiv), styrene (**6b**) (52 µL, 47 mg, 0.45 mmol, 1.0 equiv), 3DPA2FBN (2.80 mM in DMF, 1.60 mL, 4.5 µmol, 1 mol%), tetrabutylammonium azide (70.3 mM in DMF, 1.28 mL, 90 µmol, 20 mol%) and DMF (120 µL) were reacted for 20 h with a 425 nm LED lamp. The mixture was concentrated *in vacuo* and purified *via* flash column chromatography on silica gel (12 g) in CH_2Cl_2 (5 CV) then 100:0:0→95:4.5:0.5 CH_2Cl_2–MeOH–aq. NH_4OH (over 20 CV) then 95:4.5:0.5 CH_2Cl_2–MeOH–aq. NH_4OH (5 CV) to give **7ab** as a yellow oil (84.0 mg, 92%). The ^{1}H NMR data was in agreement with the literature.[9]

Data for **7ab**:

^{1}H NMR: (400 MHz, $CDCl_3$)
7.31–7.25 (m, 2H, C(2″)*H*, C(6″)*H*), 7.24–7.13 (m, 3H, C(3″)*H*, C(4″)*H*, C(5″)*H*), 2.72–2.59 (m, 2H, C(1′)H_2), 1.70–1.64 (m, 2H, C(2′)H_2), 1.58–1.34 (m, 10H, C(2)H_2, C(3)H_2, C(4)H_2, C(5)H_2, C(6)H_2), 1.24 (br s, 2H, NH_2)

^{13}C NMR: (101 MHz, $CDCl_3$)
143.3 (*C*(1″)), 128.5 (*C*(3″), *C*(5″)), 128.5 (*C*(2″), *C*(6″)), 125.7 (*C*(4″)), 50.8 (*C*(1)), 44.8 (br s, *C*(2′)), 38.9 (*C*(2), *C*(6)), 29.7 (*C*(1′)), 26.2 (*C*(4)), 22.4 (*C*(3), *C*(5))

IR: (neat)
3025 (w), 2923 (m), 2851 (w), 1603 (w), 1497 (w), 1452 (w), 1264 (w), 1188 (w), 1069 (w), 1030 (w), 929 (w), 905 (w), 845 (w), 808 (w), 752 (w), 721 (w), 697 (m)

MS: (ESI^+)
421 (13%), 287 (13%), 286 (55%), 205 (175), 204 ($[M+H]^+$, 100%)

HRMS: (ESI^+)
Calcd for $C_{14}H_{22}N$: 204.1752, found: 204.1751

Preparation of (*RS*)-1-(2-phenylpropyl)cyclohexan-1-amine (7ad)

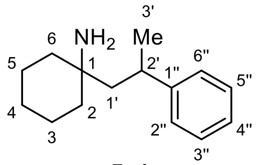

7ad

Following ***General Procedure 1***, cyclohexylamine (**1a**) (52 µL, 45 mg, 0.45 mmol, 1.0 equiv), α-methylstyrene (**6d**) (59 µL, 53 mg, 0.45 mmol, 1.0 equiv), 3DPA2FBN (2.80 mM in DMF, 1.60 mL, 4.5 µmol, 1 mol%), tetrabutylammonium azide (70.3 mM in DMF, 1.28 mL, 90 µmol, 20 mol%) and DMF (120 µL) were reacted for 20 h with a 425 nm LED lamp. The mixture was concentrated *in vacuo* and purified *via* flash column chromatography on silica gel (12 g) in CH_2Cl_2 (5 CV) then 100:0:0→95:4.5:0.5 CH_2Cl_2–MeOH–aq. NH_4OH (over 20 CV) then 95:4.5:0.5 CH_2Cl_2–MeOH–aq. NH_4OH (5 CV) to give **7ad** as a yellow oil (89 mg, 91%).

Data for **7ad**:

^{1}H NMR: (400 MHz, $CDCl_3$)
7.31–7.23 (m, 4H, C(2″)*H*, C(3″)*H*, C(5″)*H*, C(5″)*H*), 7.21–7.13 (m, 1H, C(4″)*H*), 3.02–2.93 (m, 1H, C(2′)*H*), 1.92–1.85 (m, 1H, C(1′)H_A), 1.70–1.65 (m, 1H, C(1′)H_B), 1.48–1.22 (m, 13H, C(2)H_2, C(3)H_2, C(4)H_2, C(5)H_2, C(6)H_2, C(3′)H_3), 1.04 (br s, 2H, NH_2)

^{13}C NMR: (101 MHz, $CDCl_3$)
149.2 (*C*(1‴)), 128.6 (*C*(3″), *C*(5″)), 127.2 (*C*(2″), *C*(6″)), 125.9 (*C*(4″)), 51.5 (*C*(1)), 50.7 (*C*(1′)), 39.5 (*C*(2), *C*(6)), 35.6 (*C*(2′)), 26.1 (*C*(4) or *C*(3′)), 26.0 (*C*(4) or *C*(3′)), 22.4 (*C*(3) or *C*(5)), 22.2 (*C*(3) or *C*(5))

IR: (neat)
3027 (w), 2922 (m), 2852 (w), 1602 (w), 1494 (w), 1450 (w), 1374 (w), 1079 (w), 1028 (w), 906 (w), 845 (w), 816 (w), 762 (m), 723 (w), 699 (s)

MS: (ESI^+)
219 (17%), 218 ($[M+H]^+$, 100%), 125 (12%)

HRMS: (ESI^+)
Calcd for $C_{15}H_{24}N$: 218.1909, found: 218.1908

Preparation of (*RS*)-1-(1-phenylpropan-2-yl)cyclohexan-1-amine (7ae)

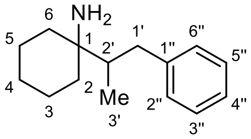

7ae

Following ***General Procedure 1***, cyclohexylamine (**1a**) (52 μL, 45 mg, 0.45 mmol, 1.0 equiv), *trans*-β-methylstyrene (**6e**) (58 μL, 53 mg, 0.45 mmol, 1.0 equiv), 3DPA2FBN (2.80 mM in DMF, 1.60 mL, 4.5 μmol, 1 mol%), tetrabutylammonium azide (70.3 mM in DMF, 1.28 mL, 90 μmol, 20 mol%) and DMF (120 μL) were reacted for 20 h with a 425 nm LED lamp. The mixture was concentrated *in vacuo* and purified *via* flash column chromatography on silica gel (12 g) in CH_2Cl_2 (5 CV) then 100:0:0→95:4.5:0.5 CH_2Cl_2–MeOH–aq. NH_4OH (over 20 CV) then 95:4.5:0.5 CH_2Cl_2–MeOH–aq. NH_4OH (5 CV) to give 7ae as a colourless oil (27 mg, 28%).

A repeat of the above reaction in DMF-d_7 allowed us to measure the following product distribution, with yields calculated using the Bu_4N^+ ion as an internal standard: **7ae** (40%), **6e** (24%) and tentatively assigned allylbenzene (6%).

Data for **7ae**:

^{1}H NMR: (400 MHz, $CDCl_3$)
7.30–7.25 (m, 2H, C(2″)*H*, C(6″)*H*), 7.21–7.14 (m, 3H, C(3″)*H*, C(4″)*H*, C(5″)*H*), 3.03 (dd, *J* = 13.1, 2.8 Hz, 1H, C(1′)H_A), 2.13 (dd, *J* = 13.1, 11.2 Hz, 1H, C(1′)H_B), 1.68 (ddd, *J* = 11.2, 6.8, 2.8 Hz, 1H, C(2′)*H*), 1.60–1.21 (m, 12H, C(2)H_2, C(3)H_2, C(4)H_2, C(5)H_2, C(6)H_2 NH_2), 0.76 (d, *J* = 6.8 Hz, 3H, C(3′)H_3)

^{13}C NMR: (101 MHz, $CDCl_3$)
142.6 (*C*(1″)), 129.4 (*C*(3″), *C*(5″)), 128.3 (*C*(2″), *C*(6″)), 125.7 (*C*(4″)), 52.9 (*C*(1)), 44.6 (*C*(2′)), 37.0 (*C*(1′)), 36.5 (*C*(2) or *C*(6)), 36.2 (*C*(2) or *C*(6)), 26.2 (*C*(4)), 22.2 (*C*(3) or *C*(5)), 22.1 (*C*(3) or *C*(5)), 12.6 (*C*(3′))

IR: (neat)
3025 (w), 2925 (m), 2851 (w), 1603 (w), 1494 (w), 1452 (w), 1378 (w), 1056 (w), 1030 (w), 905 (w), 799 (w), 746 (m), 697 (m)

MS: (ESI^+)
479 (17%), 260 (30%), 219 (16%), 218 ($[M+H]^+$, 100%)

HRMS: (ESI^+)
Calcd for $C_{15}H_{24}N$: 218.1909, found: 218.1906

Preparation of (*RS*)-1-(2,3-dihydro-1*H*-inden-2-yl)cyclohexan-1-amine (7af)

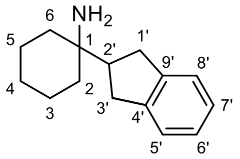

7af

Following ***General Procedure 1***, cyclohexylamine (**1a**) (52 µL, 45 mg, 0.45 mmol, 1.0 equiv), indene (**6f**) (52 µL, 52 mg, 0.45 mmol, 1.0 equiv), 3DPA2FBN (2.80 mM in DMF, 1.60 mL, 4.5 µmol, 1 mol%), tetrabutylammonium azide (70.3 mM in DMF, 1.28 mL, 90 µmol, 20 mol%) and DMF (120 µL) were reacted for 20 h with a 425 nm LED lamp. The mixture was concentrated *in vacuo* and purified *via* flash column chromatography on silica gel (12 g) in CH_2Cl_2 (5 CV) then 100:0:0→95:4.5:0.5 CH_2Cl_2–MeOH–aq. NH_4OH (over 20 CV) then 95:4.5:0.5 CH_2Cl_2–MeOH–aq. NH_4OH (5 CV), followed by reversed-phase flash column chromatography on C_{18} silica gel (15.5 g) in 98:2 H_2O–MeOH (5 CV) then 98:2→0:100 H_2O–MeOH (over 15 CV) then MeOH (5 CV) to give **7af** as a yellow solid (36.6 mg, 38%).

Data for **7af**:

mp: 82–84 °C (CH_2Cl_2)

^{1}H NMR: (400 MHz, $CDCl_3$)

7.23–7.16 (m, 2H, C(6′)*H*, C(7′)*H*), 7.15–7.09 (m, 2H, C(5′)*H*, C(8′)*H*), 2.86 (d, *J* = 9.3 Hz, 4H, C(1′)H_2, C(3′)H_2), 2.59 (p, *J* = 9.3 Hz, 1H, C(2′)*H*), 1.60–1.51 (m, 5H, C(3)H_2, C(4)H_A, C(5)H_2), 1.48–1.41 (m, 4H, C(2)H_2, C(6)H_2), 1.34–1.29 (m, 3H, C(4)H_B, NH_2)

^{13}C NMR: (126 MHz, $CDCl_3$)

143.3 (*C*(4′)), 126.3 (*C*(5′), *C*(8′)), 124.6 (*C*(6′), *C*(7′)), 52.2 (*C*(1)), 50.1 (*C*(2′)), 37.2 (*C*(2), *C*(6)), 33.0 (*C*(1′), *C*(3′)), 26.1 (*C*(4)), 22.0 (*C*(3), *C*(5))

IR: (neat)

2924 (m), 2850 (m), 1607 (w), 1485 (w), 1474 (w), 1458 (w), 1438 (w), 1282 (w), 1265 (w), 1243 (w), 1222 (w), 1207 (w), 1166 (w), 992 (w), 950 (w), 879 (w), 846 (w), 836 (w), 814 (w), 767 (w), 746 (s), 652 (w)

MS: (ESI$^+$)

217 (16%), 216 ($[M+H]^+$, 100%), 199 (12%)

HRMS: (ESI$^+$)

Calcd for $C_{15}H_{22}N$: 216.1752, found: 216.1730

Preparation of methyl (*RS*)-2-(1-aminocyclohexyl)-3-phenylpropanoate (7ag)

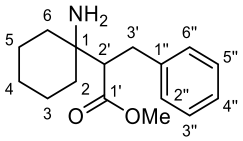

7ag

Following ***General Procedure 1***, cyclohexylamine (**1a**) (52 µL, 45 mg, 0.45 mmol, 1.0 equiv), methyl cinnamate (**6g**) (73 mg, 0.45 mmol, 1.0 equiv), 3DPA2FBN (2.80 mM in DMF, 1.60 mL, 4.5 µmol, 1 mol%), tetrabutylammonium azide (70.3 mM in DMF, 1.28 mL, 90 µmol, 20 mol%) and DMF (120 µL) were reacted for 20 h with a 425 nm LED lamp. The mixture was concentrated *in vacuo* and purified *via* flash column chromatography on silica gel (12 g) in CH_2Cl_2 (5 CV) then 100:0:0→95:4.5:0.5 CH_2Cl_2–MeOH–aq. NH_4OH (over 20 CV) then 95:4.5:0.5 CH_2Cl_2–MeOH–aq. NH_4OH (5 CV) to give **7ag** as a colourless oil (46.8 mg, 40%).

Data for **7ag**:

^{1}H NMR: (400 MHz, $CDCl_3$)
7.27–7.23 (m, 2H, C(2″)*H*, C(6″)*H*), 7.20–7.16 (m, 1H, C(4″)*H*), 7.15–7.11 (m, 2H, C(3″)*H*, C(5″)*H*), 3.47 (s, 3H, O*Me*), 2.99–2.88 (m, 2H, C(3′)H_2), 2.73–2.67 (m, 1H, C(2′)*H*), 1.68–1.44 (m, 10H, C(2)H_2, C(3)H_2, C(4)H_A, C(5)H_2, C(6)H_A, NH_2), 1.35–1.25 (m, 2H, C(4)H_B, C(6)H_B)

^{13}C NMR: (101 MHz, $CDCl_3$)
175.3 (*C*(1′)), 140.0 (*C*(1″)), 128.9 (*C*(3″), *C*(5″)), 128.5 (*C*(2″), *C*(6″)), 126.3 (*C*(4″)), 58.2 (*C*(2′)), 52.8 (*C*(1)), 51.2 (O*Me*), 38.1 (*C*(6)), 36.4 (*C*(2)), 33.1 (*C*(3′)), 25.9 (*C*(4)), 21.9 (*C*(3), *C*(5))

IR: (neat)
2927 (w), 2856 (w), 1722 (m), 1603 (w), 1495 (w), 1447 (w), 1435 (w), 1357 (w), 1335 (w), 1239 (w), 1199 (w), 1152 (m), 1070 (w), 1030 (w), 998 (w), 931 (w), 910 (w), 872 (w), 837 (w), 818 (w), 792 (w), 748 (w), 700 (m)

MS: (ESI^+)
263 (23%), 262 ($[M+H]^+$, 100%), 126 (13%)

HRMS: (ESI^+)
Calcd for $C_{16}H_{24}NO_2$: 284.1626, found: 284.1618

Preparation of methyl (*RS*)-3-phenyl-2-(1-(3-phenylthioureido)cyclohexyl)propanoate (39)

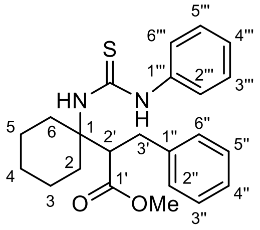

39

Phenyl isothiocyanate (14.2 mg, 0.105 mmol, 1.1 equiv) was added to a solution of **7ag** (25.0 mg, 0.096 mmol, 1.0 equiv) in hexane:Et3N:MeCN (1:0.1:0.1), and the mixture was stirred for 16 h at rt. The precipitate was washed with hexane (2 × 5 mL) to give **39** as a white solid (29.7 mg, 78%).

Data for **39**:

mp: 130–132 °C (hexane)

^{1}H NMR: (400 MHz, $CDCl_3$)
7.50–7.43 (m, 2H, C(2‴)*H*, C(6‴)*H*), 7.37–7.33 (m, 1H, C(4‴)*H*), 7.28–7.16 (m, 7H, C(2″)*H*, C(3″)*H*, C(4″)*H*, C(5″)*H*, C(6″)*H*, C(3‴)*H*, C(5‴)*H*), 5.87 (s, 1H, N*H*), 4.58 (br s, 1H, N*H*), 3.45 (s, 3H, O*Me*), 3.36–3.10 (m, 1H, C(2′)*H*), 2.85–2.72 (m, 2H, C(3′)H_2), 2.09–1.98 (m, 1H, C(2)H_A), 1.76–1.54 (m, 5H, C(2)H_B, C(3)H_A, C(4)H_A, C(6)H_2), 1.40–1.31 (m, 1H, C(4)H_B), 1.26–1.07 (m, 3H, C(3)H_B, C(5)H_2)

^{13}C NMR: (126 MHz, $CDCl_3$)
179.7 (*C*=S), 174.7 (*C*=O), 139.3 (*C*(1″)), 136.4 (br s, *C*(1‴)), 130.4 (*C*(2‴), *C*(6‴)), 129.1 (*C*(3″), *C*(5″)), 128.5 (*C*(2″), *C*(6″)), 127.9 (*C*(4‴)), 126.5 (*C*(4″)), 126.1 (*C*(3‴), *C*(5‴)), 60.3 (*C*(2′)), 52.9 (br s, *C*(1)), 51.5 (O*Me*), 33.6 (*C*(3′)), 31.8 (*C*(2), *C*(6)), 25.2 (*C*(4)), 21.7 (*C*(3) or *C*(5)), 21.4 (*C*(3) or *C*(5))

IR: (neat)
3362 (w), 3145 (w), 3061 (w), 3031 (w), 2938 (w), 2854 (w), 1732 (m), 1586 (w), 1539 (m), 1507 (m), 1495 (m), 1463 (w), 1450 (w), 1434 (w), 1385 (w), 1350 (w), 1328 (w), 1301 (w), 1283 (m), 1260 (m), 1248 (m), 1219 (m), 1168 (m), 1151 (m), 1136 (m), 1096 (w), 1068 (w), 1044 (w), 1030 (w), 1015 (w), 952 (w), 882 (w), 843 (w), 763 (w), 746 (w), 719 (w), 699 (m)

MS: (ESI^+)
435 (12%), 420 (12%), 419 (43%), 398 (26%), 397 ($[M+H]^+$, 100%), 306 (14%)

<u>HRMS:</u> (ESI^+)

Calcd for $C_{23}H_{29}N_2O_2S$: 397.1950, found: 397.1950

Crystals suitable for X-ray diffraction were obtained through recrystallisation of **39** from hexane.

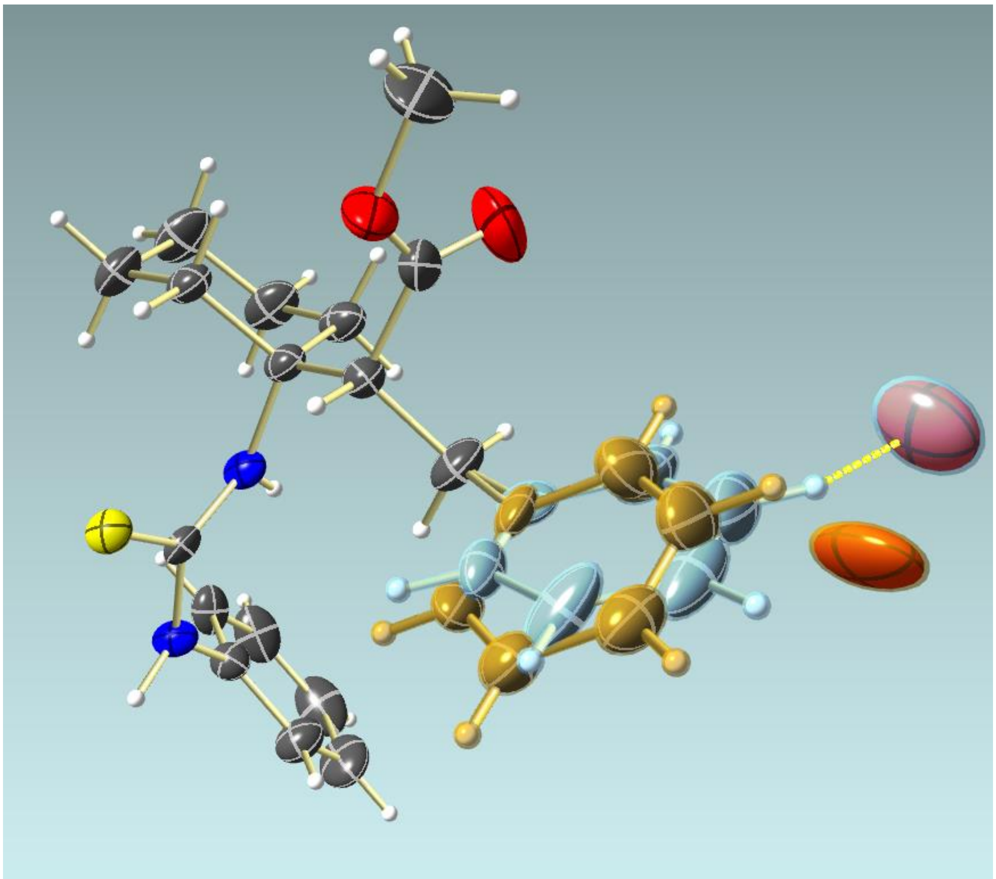

The structure was refined as a 2-component twin. One phenyl group is disordered over two sites in the ratio 40:60. The compound contains traces of water (O3 and O4) which have been refined with 25% occupation. O4 is located close to a centre of inversion. Hydrogen atoms for the water molecules could not be located in the difference Fourier map and have been omitted. The crystallographic data in CIF format has been deposited with CCDC. These data can be obtained free of charge at www.ccdc.cam.ac.uk/conts/retrieving.html [or from the Cambridge Crystallographic Data Centre, 12, Union Road, Cambridge CB2 1EZ, UK; fax: (internet.) +44-1223/336-033; E-mail: deposit@ccdc.cam.ac.uk]. CCDC No. 2093033

Table 1. Crystal data and structure refinement for 39.

Empirical formula	C92 H112 N8 O10 S4
Formula weight	1618.13

Temperature	150.00(10) K	
Wavelength	0.71073 Å	
Crystal system	Monoclinic	
Space group	$P2_1/c$	
Unit cell dimensions	a = 17.9253(10) Å	α= 90°.
	b = 9.7868(4) Å	β= 104.664(5)°.
	c = 12.9086(6) Å	γ = 90°.
Volume	2190.81(19) $Å^3$	
Z	1	
Density (calculated)	1.226 Mg/m^3	
Absorption coefficient	0.171 mm^{-1}	
F(000)	864	
Crystal size	0.550 x 0.450 x 0.220 mm^3	
Theta range for data collection	3.057 to 25.712°.	
Index ranges	-21<=h<=21, -11<=k<=11, -15<=l<=15	
Reflections collected	5309	
Independent reflections	5309 [R(int) = ?]	
Completeness to theta = 25.242°	99.90%	
Absorption correction	Semi-empirical from equivalents	
Max. and min. transmission	1.00000 and 0.91838	
Refinement method	Full-matrix least-squares on F^2	
Data / restraints / parameters	5309 / 0 / 312	
Goodness-of-fit on F^2	1.051	
Final R indices [I>2sigma(I)]	R1 = 0.0368, wR2 = 0.0883	
R indices (all data)	R1 = 0.0455, wR2 = 0.0906	

Extinction coefficient	n/a
Largest diff. peak and hole	0.199 and -0.197 e.Å^{-3}

Table 2. Atomic coordinates (x 10^4) and equivalent isotropic displacement parameters (Å^{2}x 10^3) for 39. U(eq) is defined as one third of the trace of the orthogonalized U^{ij} tensor.

	x	y	z	U(eq)
C(1)	9305(1)	7593(2)	3412(2)	26(1)
C(2)	9264(1)	8692(2)	4065(2)	39(1)
C(3)	9240(2)	10013(2)	3662(2)	55(1)
C(4)	9251(2)	10223(3)	2599(2)	56(1)
C(5)	9294(1)	9121(2)	1957(2)	48(1)
C(6)	9329(1)	7808(2)	2358(2)	35(1)
C(7)	8827(1)	5241(2)	3580(1)	23(1)
C(8)	7481(1)	4749(2)	2353(2)	29(1)
C(9)	7039(1)	5572(2)	1370(2)	39(1)
C(10)	7437(2)	5579(2)	459(2)	49(1)
C(11)	7607(2)	4137(2)	133(2)	55(1)
C(12)	8061(1)	3320(2)	1099(2)	42(1)
C(13)	7631(1)	3300(2)	1976(2)	33(1)
C(14)	7049(1)	4678(2)	3255(2)	32(1)
C(15)	6269(1)	3994(3)	2888(2)	47(1)
C(16)	5576(2)	1936(3)	2917(4)	113(2)
C(17)	6933(2)	6081(2)	3739(2)	47(1)
C(18)	6681(5)	6076(9)	4681(6)	39(3)
C(19)	7120(6)	5501(9)	5625(7)	37(3)
C(20)	6831(8)	5455(7)	6527(7)	64(4)

C(21)	6104(7)	5982(9)	6486(9)	63(4)
C(22)	5666(4)	6557(12)	5543(9)	81(4)
C(23)	5955(4)	6604(12)	4640(7)	65(3)
C(18A)	6762(2)	5870(5)	4880(3)	35(2)
C(19A)	7390(2)	5667(6)	5752(4)	39(2)
C(20A)	7277(3)	5503(5)	6770(3)	47(1)
C(21A)	6535(4)	5543(6)	6917(3)	49(2)
C(22A)	5907(2)	5746(7)	6045(4)	56(2)
C(23A)	6021(2)	5910(6)	5027(3)	52(2)
N(1)	9357(1)	6250(2)	3857(1)	26(1)
N(2)	8214(1)	5513(2)	2766(1)	26(1)
O(1)	5688(1)	4524(2)	2395(2)	85(1)
O(2)	6293(1)	2704(2)	3226(2)	65(1)
S	8973(1)	3764(1)	4287(1)	27(1)
O(3)	4337(6)	7711(11)	4887(8)	122(4)
O(4)	5119(7)	9393(11)	4808(11)	151(5)

Table 3. Bond lengths [Å] for 39.

C(1)-C(2)	1.380(3)	C(15)-O(1)	1.193(3)
C(1)-C(6)	1.388(3)	C(15)-O(2)	1.334(3)
C(1)-N(1)	1.428(2)	C(16)-O(2)	1.454(3)
C(2)-C(3)	1.390(3)	C(16)-H(16A)	0.98
C(2)-H(2)	0.95	C(16)-H(16B)	0.98
C(3)-C(4)	1.394(3)	C(16)-H(16C)	0.98
C(3)-H(3)	0.95	C(17)-C(18)	1.400(6)

C(4)-C(5)	1.374(3)	C(17)-C(18A)	1.591(4)
C(4)-H(4)	0.95	C(17)-H(17A)	0.99
C(5)-C(6)	1.381(3)	C(17)-H(17B)	0.99
C(5)-H(5)	0.95	C(18)-C(19)	1.39
C(6)-H(6)	0.95	C(18)-C(23)	1.39
C(7)-N(2)	1.341(2)	C(19)-C(20)	1.39
C(7)-N(1)	1.354(2)	C(19)-H(19)	0.95
C(7)-S	1.6945(18)	C(20)-C(21)	1.39
C(8)-N(2)	1.488(2)	C(20)-H(20)	0.95
C(8)-C(9)	1.542(3)	C(21)-C(22)	1.39
C(8)-C(13)	1.545(3)	C(21)-H(21)	0.95
C(8)-C(14)	1.554(3)	C(22)-C(23)	1.39
C(9)-C(10)	1.523(3)	C(22)-H(22)	0.95
C(9)-H(9A)	0.99	C(23)-H(23)	0.95
C(9)-H(9B)	0.99	C(18A)-C(19A)	1.39
C(10)-C(11)	1.526(3)	C(18A)-C(23A)	1.39
C(10)-H(10A)	0.99	C(19A)-C(20A)	1.39
C(10)-H(10B)	0.99	C(19A)-H(19A)	0.95
C(11)-C(12)	1.531(3)	C(20A)-C(21A)	1.39
C(11)-H(11A)	0.99	C(20A)-H(20A)	0.95
C(11)-H(11B)	0.99	C(21A)-C(22A)	1.39
C(12)-C(13)	1.523(3)	C(21A)-H(21A)	0.95
C(12)-H(12A)	0.99	C(22A)-C(23A)	1.39
C(12)-H(12B)	0.99	C(22A)-H(22A)	0.95
C(13)-H(13A)	0.99	C(23A)-H(23A)	0.95

C(13)-H(13B)	0.99	N(1)-H(1)	0.95(2)
C(14)-C(15)	1.514(3)	N(2)-H(2A)	0.858(18)
C(14)-C(17)	1.544(3)	O(4)-O(4)#1	1.40(2)
C(14)-H(14)	1		

Table 4. Bond angles [°] for 39.

C(2)-C(1)-C(6)	120.00(18)	C(17)-C(14)-H(14)	107.3
C(2)-C(1)-N(1)	118.71(17)	C(8)-C(14)-H(14)	107.3
C(6)-C(1)-N(1)	121.22(18)	O(1)-C(15)-O(2)	122.5(2)
C(1)-C(2)-C(3)	119.9(2)	O(1)-C(15)-C(14)	126.0(2)
C(1)-C(2)-H(2)	120.1	O(2)-C(15)-C(14)	111.4(2)
C(3)-C(2)-H(2)	120.1	O(2)-C(16)-H(16A)	109.5
C(2)-C(3)-C(4)	119.9(2)	O(2)-C(16)-H(16B)	109.5
C(2)-C(3)-H(3)	120.1	H(16A)-C(16)-H(16B)	109.5
C(4)-C(3)-H(3)	120.1	O(2)-C(16)-H(16C)	109.5
C(5)-C(4)-C(3)	119.7(2)	H(16A)-C(16)-H(16C)	109.5
C(5)-C(4)-H(4)	120.1	H(16B)-C(16)-H(16C)	109.5
C(3)-C(4)-H(4)	120.1	C(18)-C(17)-C(14)	117.0(4)
C(4)-C(5)-C(6)	120.5(2)	C(14)-C(17)-C(18A)	109.7(2)
C(4)-C(5)-H(5)	119.7	C(18)-C(17)-H(17A)	108
C(6)-C(5)-H(5)	119.7	C(14)-C(17)-H(17A)	108
C(5)-C(6)-C(1)	120.0(2)	C(18)-C(17)-H(17B)	108
C(5)-C(6)-H(6)	120	C(14)-C(17)-H(17B)	108
C(1)-C(6)-H(6)	120	H(17A)-C(17)-H(17B)	107.3

N(2)-C(7)-N(1)	116.30(16)	C(19)-C(18)-C(23)	120
N(2)-C(7)-S	125.19(15)	C(19)-C(18)-C(17)	121.9(5)
N(1)-C(7)-S	118.50(14)	C(23)-C(18)-C(17)	118.1(5)
N(2)-C(8)-C(9)	104.82(16)	C(20)-C(19)-C(18)	120
N(2)-C(8)-C(13)	111.42(16)	C(20)-C(19)-H(19)	120
C(9)-C(8)-C(13)	108.33(16)	C(18)-C(19)-H(19)	120
N(2)-C(8)-C(14)	108.46(15)	C(19)-C(20)-C(21)	120
C(9)-C(8)-C(14)	112.96(17)	C(19)-C(20)-H(20)	120
C(13)-C(8)-C(14)	110.73(16)	C(21)-C(20)-H(20)	120
C(10)-C(9)-C(8)	113.19(19)	C(20)-C(21)-C(22)	120
C(10)-C(9)-H(9A)	108.9	C(20)-C(21)-H(21)	120
C(8)-C(9)-H(9A)	108.9	C(22)-C(21)-H(21)	120
C(10)-C(9)-H(9B)	108.9	C(23)-C(22)-C(21)	120
C(8)-C(9)-H(9B)	108.9	C(23)-C(22)-H(22)	120
H(9A)-C(9)-H(9B)	107.8	C(21)-C(22)-H(22)	120
C(9)-C(10)-C(11)	112.0(2)	C(22)-C(23)-C(18)	120
C(9)-C(10)-H(10A)	109.2	C(22)-C(23)-H(23)	120
C(11)-C(10)-H(10A)	109.2	C(18)-C(23)-H(23)	120
C(9)-C(10)-H(10B)	109.2	C(19A)-C(18A)-C(23A)	120
C(11)-C(10)-H(10B)	109.2	C(19A)-C(18A)-C(17)	117.5(3)
H(10A)-C(10)-H(10B)	107.9	C(23A)-C(18A)-C(17)	122.5(3)
C(10)-C(11)-C(12)	111.15(18)	C(20A)-C(19A)-C(18A)	120
C(10)-C(11)-H(11A)	109.4	C(20A)-C(19A)-H(19A)	120
C(12)-C(11)-H(11A)	109.4	C(18A)-C(19A)-H(19A)	120
C(10)-C(11)-H(11B)	109.4	C(19A)-C(20A)-C(21A)	120

C(12)-C(11)-H(11B)	109.4	C(19A)-C(20A)-H(20A)	120
H(11A)-C(11)-H(11B)	108	C(21A)-C(20A)-H(20A)	120
C(13)-C(12)-C(11)	110.34(19)	C(22A)-C(21A)-C(20A)	120
C(13)-C(12)-H(12A)	109.6	C(22A)-C(21A)-H(21A)	120
C(11)-C(12)-H(12A)	109.6	C(20A)-C(21A)-H(21A)	120
C(13)-C(12)-H(12B)	109.6	C(23A)-C(22A)-C(21A)	120
C(11)-C(12)-H(12B)	109.6	C(23A)-C(22A)-H(22A)	120
H(12A)-C(12)-H(12B)	108.1	C(21A)-C(22A)-H(22A)	120
C(12)-C(13)-C(8)	112.58(17)	C(22A)-C(23A)-C(18A)	120
C(12)-C(13)-H(13A)	109.1	C(22A)-C(23A)-H(23A)	120
C(8)-C(13)-H(13A)	109.1	C(18A)-C(23A)-H(23A)	120
C(12)-C(13)-H(13B)	109.1	C(7)-N(1)-C(1)	126.51(17)
C(8)-C(13)-H(13B)	109.1	C(7)-N(1)-H(1)	118.0(12)
H(13A)-C(13)-H(13B)	107.8	C(1)-N(1)-H(1)	114.8(12)
C(15)-C(14)-C(17)	108.10(18)	C(7)-N(2)-C(8)	130.89(16)
C(15)-C(14)-C(8)	112.41(16)	C(7)-N(2)-H(2A)	115.9(12)
C(17)-C(14)-C(8)	114.02(18)	C(8)-N(2)-H(2A)	112.4(12)
C(15)-C(14)-H(14)	107.3	C(15)-O(2)-C(16)	116.5(2)

Symmetry transformations used to generate equivalent atoms:

#1 -x+1,-y+2,-z+1

Table 5. Anisotropic displacement parameters (Å^{2}x 10^3) for 39. The anisotropic displacement factor exponent takes the form: -2π^2[h^2 a*^{2}U^{11} + ... + 2 h k a* b* U^{12}]

	U_{11}	U_{22}	U_{33}	U_{23}	U_{13}	U_{12}
C(1)	29(1)	18(1)	30(1)	4(1)	6(1)	-3(1)
C(2)	53(2)	24(1)	40(1)	-2(1)	11(1)	-1(1)
C(3)	62(2)	19(1)	81(2)	-4(1)	13(2)	1(1)
C(4)	48(2)	33(1)	86(2)	31(2)	13(1)	-4(1)
C(5)	45(2)	48(2)	54(2)	28(1)	20(1)	3(1)
C(6)	37(1)	37(1)	35(1)	10(1)	18(1)	8(1)
C(7)	32(1)	17(1)	22(1)	-2(1)	12(1)	1(1)
C(8)	34(1)	20(1)	32(1)	-4(1)	3(1)	2(1)
C(9)	45(2)	26(1)	38(1)	-3(1)	-4(1)	6(1)
C(10)	72(2)	40(2)	29(1)	2(1)	3(1)	8(1)
C(11)	84(2)	47(2)	33(1)	-5(1)	15(1)	6(1)
C(12)	58(2)	31(1)	40(1)	-7(1)	18(1)	5(1)
C(13)	39(1)	22(1)	37(1)	-6(1)	5(1)	1(1)
C(14)	31(1)	26(1)	39(1)	-7(1)	6(1)	1(1)
C(15)	33(2)	51(2)	59(2)	-20(1)	13(1)	4(1)
C(16)	45(2)	92(3)	214(4)	-70(3)	52(2)	-40(2)
C(17)	64(2)	29(1)	49(2)	-11(1)	17(1)	5(1)
C(18)	46(6)	17(4)	54(5)	-10(4)	13(4)	-14(4)
C(19)	34(6)	28(4)	56(6)	-7(4)	26(5)	-5(4)
C(20)	117(12)	30(4)	58(6)	1(4)	47(8)	14(6)
C(21)	90(11)	37(6)	83(8)	-18(6)	62(9)	-20(7)

C(22)	55(6)	88(9)	115(9)	-49(8)	49(6)	-21(6)
C(23)	65(6)	59(7)	80(7)	-39(5)	38(5)	-20(5)
C(18A)	40(4)	24(3)	48(3)	-14(3)	23(3)	1(3)
C(19A)	28(3)	39(3)	54(3)	-14(2)	15(2)	-9(3)
C(20A)	51(3)	42(3)	47(3)	-11(2)	11(2)	3(3)
C(21A)	59(4)	43(3)	51(3)	-10(3)	27(3)	-10(3)
C(22A)	42(3)	67(5)	66(4)	-10(4)	28(3)	-10(3)
C(23A)	39(3)	64(4)	56(3)	-6(3)	15(2)	-8(3)
N(1)	36(1)	16(1)	24(1)	4(1)	4(1)	-4(1)
N(2)	36(1)	15(1)	26(1)	4(1)	5(1)	1(1)
O(1)	34(1)	110(2)	102(2)	-7(1)	1(1)	22(1)
O(2)	35(1)	45(1)	118(2)	-24(1)	26(1)	-16(1)
S	35(1)	16(1)	29(1)	6(1)	7(1)	-2(1)
O(3)	88(8)	131(9)	126(9)	15(7)	-13(7)	11(7)
O(4)	94(9)	150(12)	156(11)	-39(9)	-65(8)	11(9)

Table 6. Hydrogen coordinates (x 10^4) and isotropic displacement parameters (Å^{2}x 10 ^{3}) for 39.

	x	y	z	U(eq)
H(2)	9254	8547	4789	47
H(3)	9215	10772	4112	66
H(4)	9229	11124	2318	68
H(5)	9300	9263	1231	57
H(6)	9369	7052	1914	42

H(9A)	6516	5178	1106	47
H(9B)	6979	6526	1592	47
H(10A)	7927	6095	685	59
H(10B)	7105	6053	-167	59
H(11A)	7908	4188	-412	66
H(11B)	7116	3662	-191	66
H(12A)	8136	2372	876	50
H(12B)	8576	3737	1378	50
H(13A)	7936	2775	2596	40
H(13B)	7132	2826	1707	40
H(14)	7369	4108	3845	39
H(16A)	5430	1812	2139	170
H(16B)	5169	2437	3137	170
H(16C)	5647	1040	3268	170
H(17A)	7428	6582	3882	56
H(17B)	6554	6604	3191	56
H(19)	7616	5140	5653	44
H(20)	7131	5062	7172	77
H(21)	5907	5951	7103	75
H(22)	5169	6918	5515	97
H(23)	5655	6996	3996	78
H(19A)	7897	5640	5651	47
H(20A)	7706	5364	7366	56
H(21A)	6458	5431	7613	58
H(22A)	5400	5774	6146	67

H(23A)	5592	6049	4431	63
H(1)	9771(12)	6118(19)	4474(17)	39(6)
H(2A)	8199(11)	6315(19)	2493(14)	23(5)

Table 7. Torsion angles [°] for 39.

C(6)-C(1)-C(2)-C(3)	0.7(3)	C(14)-C(17)-C(18)-C(19)	62.5(6)
N(1)-C(1)-C(2)-C(3)	177.8(2)	C(14)-C(17)-C(18)-C(23)	-114.2(5)
C(1)-C(2)-C(3)-C(4)	0.4(4)	C(23)-C(18)-C(19)-C(20)	0
C(2)-C(3)-C(4)-C(5)	-0.6(4)	C(17)-C(18)-C(19)-C(20)	-176.7(7)
C(3)-C(4)-C(5)-C(6)	-0.2(4)	C(18)-C(19)-C(20)-C(21)	0
C(4)-C(5)-C(6)-C(1)	1.3(4)	C(19)-C(20)-C(21)-C(22)	0
C(2)-C(1)-C(6)-C(5)	-1.5(3)	C(20)-C(21)-C(22)-C(23)	0
N(1)-C(1)-C(6)-C(5)	-178.6(2)	C(21)-C(22)-C(23)-C(18)	0
N(2)-C(8)-C(9)-C(10)	65.3(2)	C(19)-C(18)-C(23)-C(22)	0
C(13)-C(8)-C(9)-C(10)	-53.8(2)	C(17)-C(18)-C(23)-C(22)	176.8(7)
C(14)-C(8)-C(9)-C(10)	-176.81(17)	C(14)-C(17)-C(18A)-C(19A)	81.7(3)
C(8)-C(9)-C(10)-C(11)	54.1(3)	C(14)-C(17)-C(18A)-C(23A)	-99.5(4)
C(9)-C(10)-C(11)-C(12)	-53.9(3)	C(23A)-C(18A)-C(19A)-C(20A)	0
C(10)-C(11)-C(12)-C(13)	55.7(3)	C(17)-C(18A)-C(19A)-C(20A)	178.8(4)
C(11)-C(12)-C(13)-C(8)	-58.2(2)	C(18A)-C(19A)-C(20A)-C(21A)	0
N(2)-C(8)-C(13)-C(12)	-58.6(2)	C(19A)-C(20A)-C(21A)-C(22A)	0
C(9)-C(8)-C(13)-C(12)	56.2(2)	C(20A)-C(21A)-C(22A)-C(23A)	0
C(14)-C(8)-C(13)-C(12)	-179.43(17)	C(21A)-C(22A)-C(23A)-C(18A)	0
N(2)-C(8)-C(14)-C(15)	176.37(17)	C(19A)-C(18A)-C(23A)-C(22A)	0
C(9)-C(8)-C(14)-C(15)	60.6(2)	C(17)-C(18A)-C(23A)-C(22A)	-178.7(4)
C(13)-C(8)-C(14)-C(15)	-61.1(2)	N(2)-C(7)-N(1)-C(1)	4.0(3)
N(2)-C(8)-C(14)-C(17)	52.9(2)	S-C(7)-N(1)-C(1)	-174.91(15)
C(9)-C(8)-C(14)-C(17)	-62.8(2)	C(2)-C(1)-N(1)-C(7)	115.4(2)
C(13)-C(8)-C(14)-C(17)	175.45(17)	C(6)-C(1)-N(1)-C(7)	-67.6(3)
C(17)-C(14)-C(15)-O(1)	45.2(3)	N(1)-C(7)-N(2)-C(8)	-170.20(18)
C(8)-C(14)-C(15)-O(1)	-81.6(3)	S-C(7)-N(2)-C(8)	8.7(3)

C(17)-C(14)-C(15)-O(2)	-132.04(19)	C(9)-C(8)-N(2)-C(7)	-177.29(18)
C(8)-C(14)-C(15)-O(2)	101.2(2)	C(13)-C(8)-N(2)-C(7)	-60.3(3)
C(15)-C(14)-C(17)-C(18)	65.9(5)	C(14)-C(8)-N(2)-C(7)	61.8(2)
C(8)-C(14)-C(17)-C(18)	-168.3(5)	O(1)-C(15)-O(2)-C(16)	3.0(4)
C(15)-C(14)-C(17)-C(18A)	73.7(3)	C(14)-C(15)-O(2)-C(16)	-179.7(2)
C(8)-C(14)-C(17)-C(18A)	-160.5(2)		

Symmetry transformations used to generate equivalent atoms:

#1 -x+1,-y+2,-z+1

Table 8. Hydrogen bonds for 39 [Å and °].

D-H...A	d(D-H)	d(H...A)	d(D...A)	<(DHA)
N(1)-H(1)...S#2	0.95(2)	2.41(2)	3.3271(18)	161.7(17)

Symmetry transformations used to generate equivalent atoms:

#1 -x+1,-y+2,-z+1 #2 -x+2,-y+1,-z+1

Preparation of 1-(4-methoxyphenethyl)cyclohexan-1-amine (7ah)

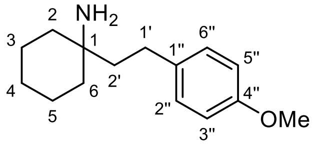

7ah

Following ***General Procedure 1***, cyclohexylamine (**1a**) (52 μL, 45 mg, 0.45 mmol, 1.0 equiv), 4-methoxystyrene (**6h**) (60 μL, 60 mg, 0.45 mmol, 1.0 equiv), 3DPA2FBN (2.80 mM in DMF, 1.60 mL, 4.5 μmol, 1 mol%), tetrabutylammonium azide (70.3 mM in DMF, 1.28 mL, 90 μmol, 20 mol%) and DMF (120 μL) were reacted for 20 h with a 425 nm LED lamp. The mixture was concentrated *in vacuo* and purified *via* flash column chromatography on silica gel (12 g) in CH_2Cl_2 (5 CV) then 100:0:0→95:4.5:0.5 CH_2Cl_2–MeOH–aq. NH_4OH (over 20 CV) then 95:4.5:0.5 CH_2Cl_2–MeOH–aq. NH_4OH (5 CV) to give **7ah** as an orange oil (62.2 mg, 59%).

Data for **7ah**:

^{1}H NMR: (400 MHz, $CDCl_3$)
7.12 (d, *J* = 8.6 Hz, 2H, C(2″)*H*, C(6″)*H*), 6.83 (d, *J* = 8.6 Hz, 2H, C(3″)*H*, C(5″)*H*), 3.78 (s, 3H, O*Me*), 2.66–2.54 (m, 2H, C(1′)H_2), 1.66–1.60 (m, 2H, C(2′)H_2), 1.57–1.22 (m, 12H, C(1)H_2, C(3)H_2, C(4)H_2, C(5)H_2, C(6)H_2, NH_2)

^{13}C NMR: (101 MHz, $CDCl_3$)
157.8 (*C*(4″)), 135.3 (*C*(1″)), 129.3 (*C*(2″), *C*(6″)), 114.0 (*C*(3″), *C*(5″)), 55.4 (O*Me*), 50.7 (*C*(1)), 45.1 (*C*(2′)), 39.0 (*C*(2′), *C*(6)), 28.7 (*C*(1′)), 26.2 (*C*(4)), 22.4 (*C*(3), *C*(5))

IR: (neat)
2923 (m), 2851 (w), 1611 (w), 1584 (w), 1510 (m), 1453 (w), 1300 (w), 1243 (m), 1177 (w), 1104 (w), 1036 (w), 930 (w), 897 (w), 820 (m), 719 (w)

MS: (ESI$^+$)
616 (11%), 282 (13%), 235 (16%), 234 ([M+H]$^+$, 100%)

HRMS: (ESI$^+$)
Calcd for $C_{15}H_{24}NO$: 234.1858, found: 234.1839

Preparation of 1-(4-methylphenethyl)cyclohexan-1-amine (7aa)

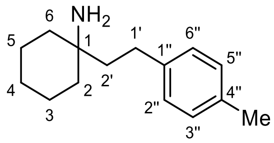

7aa

Following ***General Procedure 1***, cyclohexylamine (**1a**) (52 µL, 45 mg, 0.45 mmol, 1.0 equiv), 4-methylstyrene (**6a**) (59 µL, 53 mg, 0.45 mmol, 1.0 equiv), 3DPA2FBN (2.80 mM in DMF, 1.60 mL, 4.5 µmol, 1 mol%), tetrabutylammonium azide (70.3 mM in DMF, 1.28 mL, 90 µmol, 20 mol%) and DMF (120 µL) were reacted for 20 h with a 425 nm LED lamp. The mixture was concentrated *in vacuo* and purified *via* flash column chromatography on silica gel (12 g) in CH_2Cl_2 (5 CV) then 100:0:0→95:4.5:0.5 CH_2Cl_2–MeOH–aq. NH_4OH (over 20 CV) then 95:4.5:0.5 CH_2Cl_2–MeOH–aq. NH_4OH (5 CV) to give **7aa** as an orange oil (84.2 mg, 86%).

Data for **7aa**:

^{1}H NMR: (400 MHz, $CDCl_3$)
7.12–7.07 (m, 4H, C(2″)*H*, C(3″)*H*, C(5″)*H*, C(6″)*H*), 2.65–2.58 (m, 2H, C(1′)H_2), 2.32 (s, 3H, C(4″)*Me*), 1.67–1.62 (m, 2H, C(2′)H_2), 1.57–1.23 (m, 12H, C(2)H_2, C(3)H_2, C(4)H_2, C(5)H_2, C(6)H_2, NH_2)

^{13}C NMR: (101 MHz, $CDCl_3$)
140.2 (*C*(2″)), 135.2 (*C*(4″)), 129.2 (*C*(2″), *C*(6″) or *C*(3″), *C*(5″)), 128.4 (*C*(2″), *C*(6″) or *C*(3″), *C*(5″)), 50.7 (*C*(1)), 45.0 (*C*(2′)), 39.0 (*C*(2), *C*(6)), 29.2 (*C*(1′)), 26.2 (*C*(4)), 22.4 (*C*(3), *C*(5)), 21.1 (C(4″)*Me*)

IR: (neat)
3016 (w), 2922 (m), 2854 (w), 1579 (w), 1515 (w), 1449 (w), 1357 (w), 1264 (w), 1188 (w), 1105 (w), 1039 (w), 1022 (w), 979 (w), 930 (w), 897 (w), 844 (w), 806 (m), 718 (w)

MS: (ESI^+)
421 (14%), 219 (18%), 218 ($[M+H]^+$, 100%)

HRMS: (ESI^+)
Calcd for $C_{15}H_{24}N$: 218.1909, found: 218.1909

Preparation of 1-(4-fluorophenethyl)cyclohexan-1-amine (7ai)

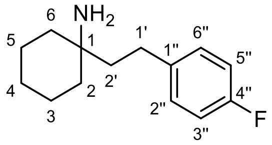

7ai

Following ***General Procedure 1***, cyclohexylamine (**1a**) (52 µL, 45 mg, 0.45 mmol, 1.0 equiv), 4-fluorostyrene (**6i**) (54 µL, 55 mg, 0.45 mmol, 1.0 equiv), 3DPA2FBN (2.80 mM in DMF, 1.60 mL, 4.5 µmol, 1 mol%), tetrabutylammonium azide (70.3 mM in DMF, 1.28 mL, 90 µmol, 20 mol%) and DMF (120 µL) were reacted for 20 h with a 425 nm LED lamp. The mixture was concentrated *in vacuo* and purified *via* flash column chromatography on silica gel (12 g) in CH_2Cl_2 (5 CV) then 100:0:0→95:4.5:0.5 CH_2Cl_2–MeOH–aq. NH_4OH (over 20 CV) then 95:4.5:0.5 CH_2Cl_2–MeOH–aq. NH_4OH (5 CV) to give **7ai** as a yellow oil (53.9 mg, 54%).

Data for **7ai**:

^{1}H NMR: (400 MHz, $CDCl_3$)
7.18–7.10 (m, 2H, C(2″)*H*, C(6″)*H*), 6.99–6.92 (m, 2H, C(3″)*H*, C(5″)*H*), 2.67–2.58 (m, 2H, C(1′)H_2), 1.66–1.60 (m, 2H, C(2′)H_2), 1.56–1.32 (m, 10H, C(2)H_2, C(3)H_2, C(4)H_2, C(5)H_2, C(6)H_2), 1.26 (br s, 2H, NH_2)

^{13}C NMR: (101 MHz, $CDCl_3$)
161.3 (d, *J* = 243.0 Hz, *C*(4″)), 138.8 (d, *J* = 3.2 Hz, *C*(1″)), 129.8 (d, *J* = 7.8 Hz, *C*(2″), *C*(6″)), 115.2 (d, *J* = 21.0 Hz, *C*(3″), *C*(5″)), 50.7 (*C*(1)), 45.0 (*C*(2′)), 39.0 (*C*(2), *C*(6)), 28.9 (*C*(1′)), 26.1 (*C*(4)), 22.4 (*C*(3), *C*(5))

^{19}F NMR: (376 MHz, $CDCl_3$)
-118.1

IR: (neat)
2923 (m), 2855 (m), 2744 (w), 2657 (w), 2587 (w), 2227 (w), 1639 (w), 1602 (w), 1557 (m), 1542 (m), 1508 (s), 1463 (m), 1455 (s), 1427 (m), 1368 (w), 1348 (m), 1337 (m), 1315 (w), 1304 (w), 1275 (m), 1265 (m), 1253 (w), 1218 (s), 1191 (w), 1159 (m), 1141 (w), 1118 (w), 1094 (w), 1064 (w), 850 (w), 822 (m), 814 (m), 790 (w), 774 (m), 766 (m), 725 (m)

MS: (ESI^+)
421 (16%), 399 (16%), 314 (11%), 233 (16%), 222 ($[M+H]^+$, 100%), 102 (10%)

HRMS: (ESI^+)
Calcd for $C_{14}H_{21}FN$: 222.1658, found: 222.1655

Preparation of 1-(4-bromophenethyl)cyclohexan-1-amine (7aj)

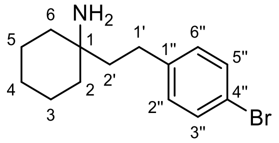

7aj

Following ***General Procedure 1***, cyclohexylamine (**1a**) (52 μL, 45 mg, 0.45 mmol, 1.0 equiv), 4-bromostyrene (**6j**) (59 μL, 82 mg, 0.45 mmol, 1.0 equiv), 3DPA2FBN (2.80 mM in DMF, 1.60 mL, 4.5 μmol, 1 mol%), tetrabutylammonium azide (70.3 mM in DMF, 1.28 mL, 90 μmol, 20 mol%) and DMF (120 μL) were reacted for 20 h with a 425 nm LED lamp. The mixture was concentrated *in vacuo* and purified *via* flash column chromatography on silica gel (12 g) in CH_2Cl_2 (5 CV) then 100:0:0→95:4.5:0.5 CH_2Cl_2–MeOH–aq. NH_4OH (over 20 CV) then 95:4.5:0.5 CH_2Cl_2–MeOH–aq. NH_4OH (5 CV), followed by reversed-phase flash column chromatography on C18 silica gel (15.5 g) in 98:2 H_2O–MeOH (5 CV) then 98:2→0:100 H_2O–MeOH (over 15 CV) then MeOH (5 CV) to give **7aj** as a yellow oil (94.3 mg, 75%).

Data for **7aj**:

^{1}H NMR: (400 MHz, $CDCl_3$)
7.39 (d, *J* = 8.3 Hz, 2H, C(2″)*H*, C(6″)*H*), 7.07 (d, *J* = 8.3 Hz, 2H, C(3″)*H*, C(5″)*H*), 2.64–2.56 (m, 2H, C(1′)H_2), 1.65–1.60 (m, 2H, C(2′)H_2), 1.57–1.31 (m, 13H, C(2)H_2, C(3)H_2, C(4)H_2, C(5)H_2, C(6)H_2, NH_2)

^{13}C NMR: (101 MHz, $CDCl_3$)
142.3 (*C*(1″)), 131.5 (*C*(2″), *C*(6″)), 130.3 (*C*(3″), *C*(5″)), 119.4 (*C*(4″)), 50.7 (*C*(1)), 44.7 (br s, *C*(2′)), 39.0 (*C*(2), *C*(6)), 29.2 (*C*(1′)), 26.1 (*C*(4)), 22.4 (*C*(3), *C*(5))

IR: (neat)
2922 (m), 2851 (w), 1674 (w), 1591 (w), 1488 (m), 1450 (w), 1403 (w), 1355 (w), 1265 (w), 1188 (w), 1071 (w), 1011 (w), 978 (w), 929 (w), 906 (w), 802 (m), 732 (w), 704 (w)

MS: (ESI^+)
285 (16%), 284 ($[M(^{81}Br)+H]^+$, 94%), 283 (17%), 282 ($[M(^{79}Br)+H]^+$, 100%)

HRMS: (ESI^+)
Calcd for $C_{14}H_{21}(^{79}Br)N$: 282.0857, found: 282.0857

Preparation of 4-(2-(1-aminocyclohexyl)ethyl)phenol (40)

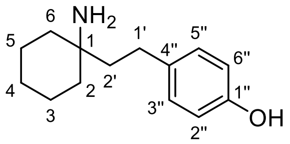

40

Following ***General Procedure 1***, cyclohexylamine (**1a**) (52 μL, 45 mg, 0.45 mmol, 1.0 equiv), 4-(4,4,5,5-tetramethyl-1,3,2-dioxaborolan-2-yl)styrene (**6k**) (104 mg, 0.45 mmol, 1.0 equiv), 3DPA2FBN (2.80 mM in DMF, 1.60 mL, 4.5 μmol, 1 mol%), tetrabutylammonium azide (70.3 mM in DMF, 1.28 mL, 90 μmol, 20 mol%) and DMF (120 μL) were reacted for 20 h with a 425 nm LED lamp. The crude material was redissolved in THF (3 mL), cooled to 0 °C and a solution of NaOH (100 mg, 2.5 mmol) and H_2O_2 (30% w/w, 0.25 mL, 2.5 mmol) in H_2O (1 mL) was added dropwise. The mixture was stirred at rt for 1 h, then sat. aq. $Na_2S_2O_3$ (10 mL) was added and the mixture was extracted with EtOAc (3 × 10 mL). The combined organic phases were dried ($MgSO_4$), filtered, and concentrated *in vacuo*. Purification *via* flash column chromatography on silica gel (12 g) in CH_2Cl_2 (5 CV) then 100:0:0→95:4.5:0.5 CH_2Cl_2–MeOH–aq. NH_4OH (over 20 CV) then 95:4.5:0.5 CH_2Cl_2–MeOH–aq. NH_4OH (5 CV) gave **40** as a white solid (105.3 mg, quant).

Data for **40**:

mp: 100 °C (decomp)

^{1}H NMR: (400 MHz, MeOD)
7.00 (d, *J* = 8.5 Hz, 2H, C(3″)*H*, C(5″)*H*), 6.68 (d, *J* = 8.5 Hz, 2H, C(2″)*H*, C(6″)*H*), 2.57–2.47 (m, 2H, C(1′)H_2), 1.66–1.61 (m, 2H, C(2′)H_2), 1.61–1.36 (m, 10H, C(2)H_2, C(3)H_2, C(4)H_2, C(5)H_2, C(6)H_2)

^{13}C NMR: (101 MHz, MeOD)
156.5 (*C*(1″)), 134.8 (*C*(4″)), 130.2 (*C*(3″), *C*(5″)), 116.2 (*C*(2″), *C*(6″)), 51.9 (*C*(1)), 44.8 (br s, *C*(2′)), 39.0 (*C*(2), *C*(6)), 29.5 (*C*(1′)), 27.0 (*C*(3)), 23.2 (*C*(3), *C*(5))

IR: (neat)
3676 (w), 2988 (w), 2931 (w), 1614 (w), 1591 (w), 1514 (w), 1453 (w), 1394 (w), 1372 (w), 1310 (w), 1248 (w), 1169 (w), 1121 (w), 1073 (w), 1027 (w), 1012 (w), 976 (w), 921 (w), 891 (w), 849 (w), 836 (w), 817 (w), 756 (w)

MS: (ESI$^+$)
338 (14%), 302 (12%), 282 (22%), 262 (11%), 221 (16%), 220 ([M+H]$^+$, 100%)

HRMS: (ESI$^+$)

Calcd for $C_{14}H_{22}NO$: 220.1701, found: 220.1701

Preparation of 1-(4-(trifluoromethyl)phenethyl)cyclohexan-1-amine (7al)

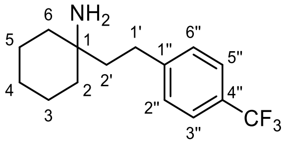

7ak

Following ***General Procedure 1***, cyclohexylamine (**1a**) (52 µL, 45 mg, 0.45 mmol, 1.0 equiv), 4-(trifluoromethyl)styrene (**6l**) (78 mg, 0.45 mmol, 1.0 equiv), 3DPA2FBN (2.80 mM in DMF, 1.60 mL, 4.5 µmol, 1 mol%), tetrabutylammonium azide (70.3 mM in DMF, 1.28 mL, 90 µmol, 20 mol%) and DMF (120 µL) were reacted for 20 h with a 425 nm LED lamp. The mixture was concentrated *in vacuo* and purified *via* flash column chromatography on silica gel (12 g) in CH_2Cl_2 (5 CV) then 100:0:0→95:4.5:0.5 CH_2Cl_2–MeOH–aq. NH_4OH (over 20 CV) then 95:4.5:0.5 CH_2Cl_2–MeOH–aq. NH_4OH (5 CV) to give **7al** as an orange oil (66.5 mg, 55%).

Data for **7al**:

^{1}H NMR: (400 MHz, $CDCl_3$)

7.52 (d, *J* = 8.0 Hz, 2H, C(3″)*H*, C(5″)*H*), 7.30 (d, *J* = 8.0 Hz, 2H, C(2″)*H*, C(6″)*H*), 2.78–2.64 (m, 2H, C(1′)H_2), 1.69–1.63 (m, 2H, C(2′)H_2), 1.59–1.30 (m, 12H, C(2)H_2, C(3)H_2, C(4)H_2, C(5)H_2, C(6)H_2, NH_2)

^{13}C NMR: (101 MHz, $CDCl_3$)

147.5 (*C*(1″)), 128.8 (*C*(2″), *C*(6″)), 128.2 (q, *J* = 32.3 Hz, *C*(4″)), 125.4 (q, *J* = 3.8 Hz, *C*(3″), *C*(5″)), 124.5 (q, *J* = 272 Hz, *C*F_3), 50.8 (*C*(1)), 44.6 (*C*(2′)), 38.9 (*C*(2), *C*(6)), 29.6 (*C*(1′)), 26.1 (*C*(4)), 22.4 (*C*(3), *C*(5))

^{19}F NMR: (376 MHz, $CDCl_3$)

-62.3

IR: (neat)

2927 (w), 2855 (w), 1673 (w), 1618 (w), 1452 (w), 1417 (w), 1322 (s), 1187 (w), 1160 (m), 1118 (m), 1067 (m), 1018 (m), 980 (w), 930 (w), 906 (w), 845 (w), 822 (m), 738 (w), 719 (w)

MS: (ESI$^+$)

421 (13%), 273 (17%), 272 ([M+H]$^+$, 100%)

HRMS: (ESI$^+$)

Calcd for $C_{15}H_{21}F_3N$: 272.1626, found: 272.1627

Preparation of methyl 4-(2-(1-aminocyclohexyl)ethyl)benzoate (7am)

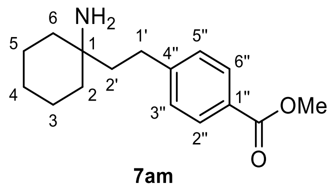

7am

Following ***General Procedure 1***, cyclohexylamine (**1a**) (52 µL, 45 mg, 0.45 mmol, 1.0 equiv), methyl 4-vinylbenzoate (**6m**) (73 mg, 0.45 mmol, 1.0 equiv), 3DPA2FBN (2.80 mM in DMF, 1.60 mL, 4.5 µmol, 1 mol%), tetrabutylammonium azide (70.3 mM in DMF, 1.28 mL, 90 µmol, 20 mol%) and DMF (120 µL) were reacted for 20 h with a 425 nm LED lamp. The mixture was concentrated *in vacuo* and purified *via* flash column chromatography on silica gel (12 g) in CH_2Cl_2 (5 CV) then 100:0:0→95:4.5:0.5 CH_2Cl_2–MeOH–aq. NH_4OH (over 20 CV) then 95:4.5:0.5 CH_2Cl_2–MeOH–aq. NH_4OH (5 CV) to give **7am** as a yellow oil (60.0 mg, 51%).

Data for **7am**:

^{1}H NMR: (400 MHz, $CDCl_3$)

7.93 (d, *J* = 8.3 Hz, 2H, C(2″)*H*, C(6″)*H*), 7.24 (d, *J* = 8.4 Hz, 2H, C(3″)*H*, C(5″)*H*), 3.88 (s, 3H, O*Me*), 2.76–2.63 (m, 2H, C(1′)H_2), 1.67–1.62 (m, 2H, C(2′)H_2), 1.58–1.29 (m, 12H, C(2)H_2, C(3)H_2, C(4)H_2, C(6)H_2, C(6)H_2, NH_2)

^{13}C NMR: (101 MHz, $CDCl_3$)

167.3 (*C*=O), 148.9 (*C*(4″)), 129.8 (*C*(2″), *C*(6″)), 128.5 (*C*(3″), *C*(5″)), 127.8 (*C*(1″)), 52.1 (O*Me*), 50.8 (*C*(1)), 44.4 (*C*(2′)), 38.9 (*C*(2), *C*(6)), 29.8 (*C*(1′)), 26.1 (*C*(4)), 22.4 (*C*(3), *C*(5))

IR: (neat)

2924 (w), 2852 (w), 2332 (w), 1717 (m), 1609 (w), 1574 (w), 1510 (w), 1435 (m), 1415 (w), 1310 (w), 1275 (s), 1177 (m), 1108 (m), 1020 (w), 967 (w), 906 (w), 838 (w), 802 (w), 767 (m), 728 (w), 701 (m), 668 (w), 651 (w)

MS: (ESI$^+$)

263 (18%), 262 ([M+H]$^+$, 100%), 245 (20%)

HRMS: (ESI$^+$)

Calcd for $C_{16}H_{24}NO_2$: 262.1807, found: 262.1805

Preparation of 1-(2-bromo-6-chlorophenethyl)cyclohexan-1-amine (7an)

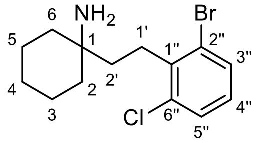

7an

Following ***General Procedure 1***, cyclohexylamine (**1a**) (52 μL, 45 mg, 0.45 mmol, 1.0 equiv), 1-bromo-3-chloro-2-vinylbenzene (**6n**) (98 mg, 0.45 mmol, 1.0 equiv), 3DPA2FBN (2.80 mM in DMF, 1.60 mL, 4.5 μmol, 1 mol%), tetrabutylammonium azide (70.3 mM in DMF, 1.28 mL, 90 μmol, 20 mol%) and DMF (120 μL) were reacted for 20 h with a 425 nm LED lamp. The mixture was concentrated *in vacuo* and purified *via* flash column chromatography on silica gel (12 g) in CH_2Cl_2 (5 CV) then 100:0:0→95:4.5:0.5 CH_2Cl_2–MeOH–aq. NH_4OH (over 20 CV) then 95:4.5:0.5 CH_2Cl_2–MeOH–aq. NH_4OH (5 CV) to give **7an** as a yellow oil (87 mg, 61%).

Data for **7an**:

^{1}H NMR: (400 MHz, $CDCl_3$)
7.44 (dd, *J* = 8.0, 1.2 Hz, 1H, C(3″)*H*), 7.29 (dd, *J* = 8.0, 1.2 Hz, 1H, C(5″)*H*), 6.96 (dd, *J* = 8.0, 8.0 Hz, 1H, C(4″)*H*), 3.04–2.96 (m, 2H, C(1′)H_2), 1.60–1.38 (m, 12H, C(2)H_2, C(3)H_2, C(4)H_2, C(5)H_2, C(6)H_2, C(2′)H_2), 1.26 (br s, 2H, NH_2)

^{13}C NMR: (101 MHz, $CDCl_3$)
140.2 (*C*(1″)), 135.0 (*C*(6″), 131.6 (*C*(3″)), 129.0 (*C*(5″)), 128.0 (*C*(4″)), 125.5 (*C*(2″)), 51.0 (*C*(1)), 40.0 (*C*(2′)), 38.8 (*C*(2), *C*(6)), 28.5 (*C*(1′)), 26.2 (*C*(4)), 22.5 (*C*(3), *C*(5))

IR: (neat)
3668 (w), 2924 (m), 2849 (w), 1677 (w), 1556 (w), 1452 (w), 1431 (m), 1257 (w), 1185 (w), 1065 (m), 973 (w), 931 (w), 896 (w), 812 (w), 769 (m), 729 (m), 705 (w)

MS: (ESI^+)
319 (16%), 318 ([M(^{81}Br)+H]$^+$, 100%), 317 (11%), 316 ([M(^{79}Br)+H]$^+$, 74%)

HRMS: (ESI^+)
Calcd for $C_{14}H_{20}$(^{81}Br)ClN: 316.0468, found: 316.0465

Preparation of 1-(2-bromo-3-chlorophenethyl)cyclohexan-1-amine (7ao) and 1-(3-chlorophenethyl)cyclohexan-1-amine (41)

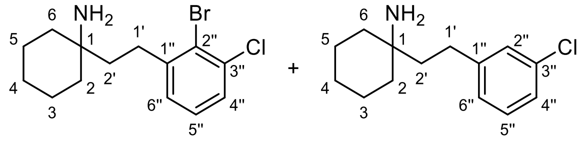

7ao **41**

Following ***General Procedure 1***, cyclohexylamine (**1a**) (34 µL, 30 mg, 0.3 mmol, 1.0 equiv), 2-bromo-1-chloro-3-vinylbenzene (**6o**) (65 mg, 0.3 mmol, 1.0 equiv), 3DPA2FBN (2.80 mM in DMF, 1.60 mL, 4.5 µmol, 1 mol%), tetrabutylammonium azide (70.3 mM in DMF, 1.28 mL, 90 µmol, 20 mol%) and DMF (120 µL) were reacted for 20 h with a 425 nm LED lamp. The mixture was concentrated *in vacuo* and purified *via* flash column chromatography on silica gel (12 g) in CH_2Cl_2 (5 CV) then 100:0:0→95:4.5:0.5 CH_2Cl_2–MeOH–aq. NH_4OH (over 20 CV) then 95:4.5:0.5 CH_2Cl_2–MeOH–aq. NH_4OH (5 CV) to give an inseparable 37:63 mixture of **7ao** to **41** as an orange oil [28.0 mg, containing 12.4 mg of **7ao** (13%) and 15.6 mg of **41** (22%)].

Data for **7ao**:

^{1}H NMR: (400 MHz, $CDCl_3$)
7.35–7.27 (m, 1H, C(4″)*H*), 7.25–7.09 (m, 2H, C(5″)*H*, C(6″)*H*), 2.89–2.81 (m, 1H, C(1′)*H*$_2$), 1.67–1.60 (m, 2H, C(2′)*H*$_2$), 1.57–1.32 (m, 12H, C(2)*H*$_2$, C(3)*H*$_2$, C(4)*H*$_2$, C(5)*H*$_2$, C(6)*H*$_2$, N*H*$_2$)

^{13}C NMR: (101 MHz, $CDCl_3$)
145.3 (*C*(1″)), 135.3 (*C*(3″)), 128.4 (*C*(6″)), 128.1 (*C*(5″)), 128.0 (*C*(4″)), 124.5 (*C*(2″)), 50.9 (*C*(1)), 42.7 (br s, *C*(2′)), 38.9 (*C*(2), *C*(6)), 31.7 (*C*(1′)), 26.1 (*C*(4)), 22.4 (*C*(3), *C*(5))

HRMS: (ESI$^+$)
Calcd for $C_{14}H_{20}(^{81}Br)ClN$: 318.0447, found: 318.0445

Data for **41**:

^{1}H NMR: (400 MHz, $CDCl_3$)
7.25–7.09 (m, 3H, C(2″)*H*, C(4″)*H*, C(5″)*H*), 7.09–7.05 (m, 1H, C(6″)*H*), 2.68–2.60 (m, 2H, C(1′)*H*$_2$), 1.67–1.60 (m, 2H, C(2′)*H*$_2$), 1.57–1.32 (m, 12H, C(2)*H*$_2$, C(3)*H*$_2$, C(4)*H*$_2$, C(5)*H*$_2$, C(6)*H*$_2$, N*H*$_2$)

<u>^{13}C NMR:</u> (101 MHz, $CDCl_3$)

145.4 (*C*(1″)), 134.2 (*C*(3″)), 129.7 (*C*(5″)), 128.6 (*C*(2″)), 126.7 (*C*(6″)), 125.9 (*C*(4″)), 50.8 (*C*(1)), 44.6 (br s, *C*(2′)), 38.9 (*C*(2), *C*(6)), 29.4 (*C*(1′)), 26.1 (*C*(4)), 22.4 (*C*(3), *C*(5))

<u>HRMS:</u> (ESI^+)

Calcd for $C_{14}H_{21}ClN$: 238.1363, found: 238.1362

Preparation of 1-(2-(pyridin-2-yl)ethyl)cyclohexan-1-amine (7ap)

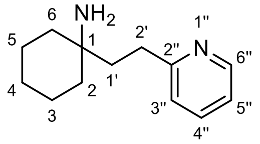

7ap

Following ***General Procedure 1***, cyclohexylamine (**1a**) (52 μL, 45 mg, 0.45 mmol, 1.0 equiv), 2-vinylpyridine (**6p**) (49 μL, 47 mg, 0.45 mmol, 1.0 equiv), 3DPA2FBN (2.80 mM in DMF, 1.60 mL, 4.5 μmol, 1 mol%), tetrabutylammonium azide (70.3 mM in DMF, 1.28 mL, 90 μmol, 20 mol%) and DMF (120 μL) were reacted for 20 h with a 425 nm LED lamp. The mixture was concentrated *in vacuo* and purified *via* flash column chromatography on silica gel (12 g) in CH_2Cl_2 (5 CV) then 100:0:0→95:4.5:0.5 CH_2Cl_2–MeOH–aq. NH_4OH (over 20 CV) then 95:4.5:0.5 CH_2Cl_2–MeOH–aq. NH_4OH (5 CV) to give **7ap** as a yellow oil (66.2 mg, 72%). The NMR spectroscopic data was in accordance with the literature.[10]

<u>Data for **7ap**:</u>

<u>^{1}H NMR:</u> (400 MHz, $CDCl_3$)

8.61–8.41 (m, 1H, C(6″)*H*), 7.64–7.51 (m, 1H, C(4″)*H*), 7.21–7.12 (m, 1H, C(3″)*H*), 7.12–7.01 (m, 1H, C(5″)*H*), 2.91–2.75 (m, 2H, C(2′)H_2), 1.83–1.72 (m, 2H, C(1′)H_2), 1.55–1.30 (m, 12H, C(2)H_2, C(3)H_2, C(4)H_2, C(5)H_2, C(6)H_2, NH_2)

<u>^{13}C NMR:</u> (101 MHz, $CDCl_3$)

163.0 (*C*(2″)), 149.4 (*C*(6″)), 136.5 (*C*(4″)), 122.9 (*C*(3″)), 121.0 (*C*(5″)), 50.8 (*C*(1)), 43.0 (*C*(2′)), 38.9 (*C*(2), *C*(6)), 32.3 (*C*(2′)), 26.2 (*C*(4)), 22.4 (*C*(3), *C*(5))

IR: (neat)

3353 (w), 3009 (w), 2923 (m), 2851 (w), 1590 (w), 1568 (w), 1474 (w), 1450 (w), 1434 (w), 1358 (w), 1267 (w), 1149 (w), 1051 (w), 993 (w), 907 (w), 845 (w), 825 (w), 775 (w), 749 (m)

MS: (ESI^+)

421 (12%), 304 (15%), 282 (42%), 206 (15%), 205 ($[M+H]^+$, 100%), 188 (17%)

HRMS: (ESI^+)

Calcd for $C_{13}H_{21}N_2$: 205.1705, found: 205.1702

Preparation of 1-(2-(4-methylthiazol-5-yl)ethyl)cyclohexan-1-amine (7aq)

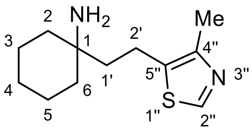

7aq

Following ***General Procedure 1***, cyclohexylamine (**1a**) (52 μL, 45 mg, 0.45 mmol, 1.0 equiv), 4-methyl-5-vinylthiazole (**6q**) (52 μL, 56 mg, 0.45 mmol, 1.0 equiv), 3DPA2FBN (2.80 mM in DMF, 1.60 mL, 4.5 μmol, 1 mol%), tetrabutylammonium azide (70.3 mM in DMF, 1.28 mL, 90 μmol, 20 mol%) and DMF (120 μL) were reacted for 20 h with a 425 nm LED lamp. The mixture was concentrated *in vacuo* and purified *via* flash column chromatography on silica gel (12 g) in CH_2Cl_2 (5 CV) then 100:0:0→95:4.5:0.5 CH_2Cl_2–MeOH–aq. NH_4OH (over 20 CV) then 95:4.5:0.5 CH_2Cl_2–MeOH–aq. NH_4OH (5 CV), followed by reversed-phase flash column chromatography on C18 silica gel (15.5 g) in 98:2 H_2O–MeOH (5 CV) then 98:2→0:100 H_2O–MeOH (over 15 CV) then MeOH (5 CV) to give **7aq** as an orange oil (39.5 mg, 39%).

Data for **7aq**:

^{1}H NMR: (400 MHz, $CDCl_3$)

8.51 (s, 1H, C(2″)*H*), 2.85–2.73 (m, 2H, C(2′)H_2), 2.37 (s, 3H, C(4″)*Me*), 1.66–1.60 (m, 2H, C(1′)H_2), 1.55–1.09 (m, 12H, C(2)H_2, C(3)H_2, C(4)H_2, C(5)H_2, C(6)H_2, NH_2)

^{13}C NMR: (101 MHz, $CDCl_3$)

148.7 (*C*(2″)), 148.2 (*C*(4″)), 132.6 (*C*(5″)), 50.7 (*C*(1)), 44.6 (*C*(1′)), 38.8 (*C*(2), *C*(6)), 26.0 (*C*(4)), 22.3 (*C*(3), *C*(5)), 20.2 (*C*(2′)), 14.9 (C(4″)*Me*)

<u>IR:</u> (neat)

3352 (w), 2922 (m), 2852 (w), 1591 (w), 1542 (w), 1450 (w), 1414 (w), 1377 (w), 1316 (w), 1265 (w), 1240 (w), 1198 (w), 1130 (w), 1033 (w), 905 (w), 840 (w), 784 (w), 732 (w), 668 (w)

<u>MS:</u> (ESI^+)

226 (14%), 225 ($[M+H]^+$, 100%), 208 (24%)

<u>HRMS:</u> (ESI^+)

Calcd for $C_{12}H_{21}N_2S$: 225.1425, found: 225.1425

Preparation of *tert*-butyl (1-(2-(pyrazin-2-yl)ethyl)cyclohexyl)carbamate (42), *tert*-butyl (*RS*)-(1-(2,4-di(pyrazin-2-yl)butyl)cyclohexyl)carbamate (43) and 1,4-di(pyrazin-2-yl)butane (44)

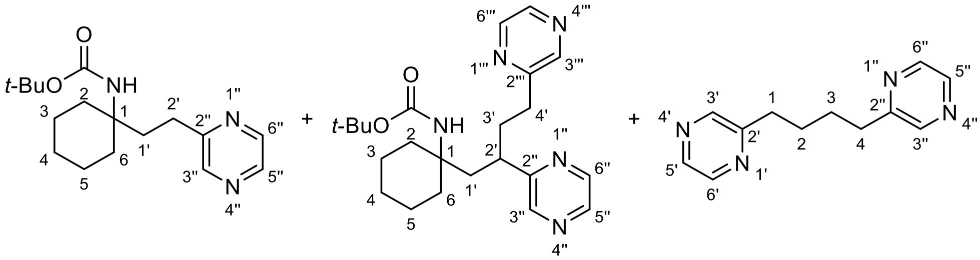

42 **43** **44**

Following ***General Procedure 1***, cyclohexylamine (**1a**) (52 μL, 45 mg, 0.45 mmol, 1.0 equiv), 2-vinylpyrazine (**6r**) (46 μL, 48 mg, 0.45 mmol, 1.0 equiv), 3DPA2FBN (2.80 mM in DMF, 1.60 mL, 4.5 μmol, 1 mol%), tetrabutylammonium azide (70.3 mM in DMF, 1.28 mL, 90 μmol, 20 mol%) and DMF (120 μL) were reacted for 20 h with a 425 nm LED lamp. The mixture was concentrated *in vacuo* and purified *via* flash column chromatography on silica gel (12 g) in CH_2Cl_2 (5 CV) then 100:0:0→95:4.5:0.5 CH_2Cl_2–MeOH–aq. NH_4OH (over 20 CV) then 95:4.5:0.5 CH_2Cl_2–MeOH–aq. NH_4OH (5 CV) to give **44** as an amorphous brown solid (20.8 mg, 43% wrt **6r**) and a mixture of **42** and **43**. Di-*tert*-butyl dicarbonate (98 mg, 0.45 mmol) was added to the mixture in CH_2Cl_2 (1 mL) and the resultant solution was stirred at rt for 3 h, followed by concentration *in vacuo*. The crude material was purified via flash column chromatography on silica gel (4 g) in CH_2Cl_2 (5 CV) then 100:0:0→90:9:1 CH_2Cl_2–MeOH–aq. NH_4OH (over 40 CV) then 90:9:1 CH_2Cl_2–MeOH–aq. NH_4OH (5 CV) to give **42** as a clear oil (20.3 mg, 22%) and **43** as a yellow oil (15.9 mg, 9% wrt **1a**).

Data for **42**:

^{1}H NMR: (400 MHz, $CDCl_3$)

8.47–8.43 (m, 2H, C(3″)*H*, C(5″)*H*), 8.38–8.35 (m, 1H, C(6″)*H*), 4.37 (s, 1H, N*H*), 2.81–2.76 (m, 2H, C(2′)H_2), 2.17–2.08 (m, 2H, C(1′)H_2), 2.04–1.93 (m, 2H, C(2)H_A, C(6)H_A), 1.57–1.25 (m, 17H, C(2)H_B, C(3)H_2, C(4)H_2, C(5)H_2, C(6)H_B, OC(CH_3)$_3$)

^{13}C NMR: (101 MHz, $CDCl_3$)

158.4 (*C*(2″)), 154.4 (*C*=O), 144.9 (*C*(3″)), 144.0 (*C*(6″)), 142.1 (*C*(5″)), 78.9 (O*C*(CH_3)$_3$), 54.5 (*C*(1)), 38.3 (*C*(1′)), 35.2 (*C*(2′), *C*(6′)), 29.9 (*C*(2″)), 28.6 (OC(*C*H_3)$_3$), 25.9 (*C*(4)), 21.8 (*C*(3), *C*(5))

IR: (neat)

2973 (w), 2930 (w), 2856 (w), 1713 (m), 1522 (w), 1496 (w), 1476 (w), 1451 (w), 1403 (w), 1390 (w), 1365 (w), 1306 (w), 1246 (w), 1163 (m), 1079 (w), 1059 (w), 1017 (w), 999 (w), 969 (w), 834 (w)

MS: (ESI^+)

328 ($[M+Na]^+$, 34%), 307 (23%), 306 ($[M+H]^+$, 100%), 189 (10%)

HRMS: (ESI^+)

Calcd for $C_{17}H_{27}N_3NaO_2$: 328.2001, found: 328.1999

Data for **43**:

^{1}H NMR: (400 MHz, $CDCl_3$)

8.50 (dd, J = 2.5, 1.5 Hz, 1H, C(5″)*H* or C(5‴)*H*), 8.46 (d, J = 1.5 Hz, 1H, C(3″)*H* or C(3‴)*H*), 8.44 (dd, J = 2.5, 1.5 Hz, 1H, C(5″)*H* or C(5‴)*H*), 8.38 (d, J = 2.5 Hz, 1H, C(6″)*H* or C(6‴)*H*), 8.35 (d, J = 2.5 Hz, 1H, C(6″)*H* or C(6‴)*H*), 8.33 (d, J = 1.5 Hz, 1H, C(3″)*H* or C(3‴)*H*), 4.12 (s, 1H, N*H*), 3.08–2.98 (m, 1H, C(2′)*H*), 2.77–2.65 (m, 1H, C(4′)H_A), 2.63–2.50 (m, 1H, C(4′)H_B), 2.47–2.33 (m, 1H, C(1′)H_A), 2.29–2.03 (m, 3H, C(1′)H_B, C(3′)H_2), 2.03–1.91 (m, 1H, C(2)H_A), 1.60–1.15 (m, 17H, C(2)H_B, C(3)H_2, C(4)H_2, C(5)H_2, C(6)H_B, OC(CH_3)$_3$), 1.06–0.93 (m, 1H, C(6)H_B)

^{13}C NMR: (101 MHz, $CDCl_3$)

161.3 (*C*(2″) or *C*(2‴)), 157.2 (*C*(2″) or *C*(2‴)), 154.1 (*C*=O), 145.0 (*C*(3″) or *C*(3‴)), 144.7 (*C*(3″) or *C*(3‴)), 144.4 (*C*(5″) or *C*(5‴)), 144.1 (*C*(5″) or *C*(5‴)), 142.4 (*C*(6″) or *C*(6‴)), 142.4 (*C*(6″) or *C*(6‴)), 78.6 (O*C*(CH_3)$_3$), 54.7 (*C*(1)),

43.3 (br s, *C*(1′)), 40.6 (*C*(2′)), 36.9 (*C*(3′)), 35.8 (*C*(2) or *C*(6)), 35.5 (*C*(2) or *C*(6)), 33.4 (*C*(4′)), 28.6 (OC(*C*H_3)$_3$), 25.7 (*C*(4)), 21.8 (*C*(3) or *C*(5)), 21.6 (*C*(3) or *C*(5))

IR: (neat)

3300 (w), 2929 (w), 2856 (w), 1709 (m), 1523 (w), 1495 (w), 1474 (w), 1450 (w), 1404 (w), 1390 (w), 1365 (w), 1327 (w), 1307 (w), 1280 (w), 1248 (w), 1162 (m), 1080 (w), 1060 (w), 1017 (w), 973 (w), 919 (w), 852 (w), 776 (w), 730 (m)

MS: (ESI^+)

506 22%, 413 (28%), 412 ([M+H]+, 100%), 312 (24%), 295 (12%)

HRMS: (ESI+)

Calcd for $C_{23}H_{34}N_5O_2$: 412.2713, found: 412.2716

Data for **44**:

^{1}H NMR: (400 MHz, $CDCl_3$)

8.48 (dd, *J* = 2.5, 1.5 Hz, 2H, C(5′)*H*, C(5″)*H*), 8.45 (d, *J* = 1.5 Hz, 2H, C(3′)*H*, C(3′)*H*), 8.40 (d, *J* = 2.5 Hz, 2H, C(6′)*H*, C(6′)*H*), 2.90–2.83 (m, 4H, C(1)H_2, C(4)H_2), 1.88–1.81 (m, 4H, C(2)H_2, C(3)H_2)

^{13}C NMR: (101 MHz, $CDCl_3$)

157.5 (*C*(2′), *C*(2″)), 144.7 (*C*(3′), *C*(3″)), 144.2 (*C*(5′), *C*(5″)), 142.4 (*C*(6′), *C*(6″)), 35.3 (*C*(1), *C*(4)), 29.0 (*C*(2), *C*(3))

IR: (neat)

3079 (w), 2940 (m), 2931 (w), 2892 (w), 2860 (w), 1672 (w), 1526 (m), 1481 (m), 1458 (m), 1420 (m), 1407 (m), 1366 (w), 1301 (w), 1244 (m), 1179 (w), 1161 (w), 1124 (m), 1056 (m), 1017 (s), 955 (w), 900 (m), 836 (m), 715 (m)

MS: (ESI^+)

304 (33%), 283 (15%), 282 (75%), 242 (29%), 216 (24%), 215 ($[M+H]^+$, 100%), 126 (12%)

HRMS: (ESI^+)

Calcd for $C_{12}H_{15}N_4$: 215.1297, found: 215.1298

Preparation of 1-(2-(2-bromopyridin-3-yl)ethyl)cyclohexan-1-amine (7as) and 1-(2-(pyridin-3-yl)ethyl)cyclohexan-1-amine (45)

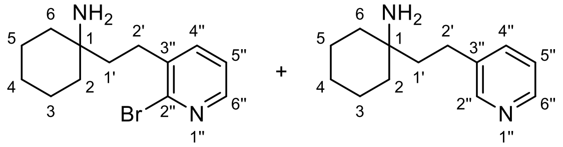

7as **45**

Following ***General Procedure 1***, cyclohexylamine (**1a**) (52 μL, 45 mg, 0.45 mmol, 1.0 equiv), 2-bromo-3-vinylpyridine (**6s**) (83 mg, 0.45 mmol, 1.0 equiv), 3DPA2FBN (2.80 mM in DMF, 1.60 mL, 4.5 μmol, 1 mol%), tetrabutylammonium azide (70.3 mM in DMF, 1.28 mL, 90 μmol, 20 mol%) and DMF (120 μL) were reacted for 20 h with a 425 nm LED lamp. The mixture was concentrated *in vacuo* to give a 60:40 mixture of **7as** to tentatively assigned de-brominated product **45**. Purification *via* flash column chromatography on silica gel (12 g) in CH_2Cl_2 (5 CV) then 100:0:0→95:4.5:0.5 CH_2Cl_2–MeOH–aq. NH_4OH (over 20 CV) then 95:4.5:0.5 CH_2Cl_2–MeOH–aq. NH_4OH (5 CV), followed by reversed-phase flash column chromatography on C18 silica gel (15.5 g) in 98:2 H_2O–MeOH (5 CV) then 98:2→0:100 H_2O–MeOH (over 15 CV) then MeOH (5 CV) gave **7as** as a brown oil (34.0 mg, 27%) and an inseparable mixture of **45** and Bu_4NN_3.

Data for **7as**:

^{1}H NMR: (400 MHz, $CDCl_3$)
8.21 (dd, *J* = 4.7, 2.0 Hz, 1H, C(6″)*H*), 7.53 (dd, *J* = 7.5, 2.0 Hz, 1H, C(4″)*H*), 7.19 (dd, *J* = 7.5, 4.7 Hz, 1H, C(5″)*H*), 2.80–2.74 (m, 2H, C(2′)H_2), 1.66–1.61 (m, 2H, C(1′)H_2), 1.56–1.39 (m, 12H, C(2)H_2, C(3)H_2, C(4)H_2, C(5)H_2, C(6)H_2, NH_2)

^{13}C NMR: (101 MHz, $CDCl_3$)
147.6 (*C*(6″)), 144.4 (*C*(3″)), 139.8 (*C*(2″)), 138.4 (*C*(4″)), 123.1 (*C*(5″)), 50.8 (*C*(1)), 42.5 (*C*(2′)), 38.8 (*C*(2), *C*(6)), 29.5 (*C*(1′)), 26.1 (*C*(4)), 22.4 (*C*(3), *C*(5))

IR: (neat)
2924 (w), 2851 (w), 1667 (w), 1577 (w), 1558 (w), 1448 (w), 1402 (m), 1263 (w), 1178 (w), 1111 (w), 1048 (w), 910 (w), 799 (w), 730 (w), 669 (w)

MS: (ESI^+)
286 (15%), 285 ($[M(^{81}Br)+H]^+$, 100%), 284 (15%), 283 ($[M(^{79}Br)+H]^+$, 100%), 268 (23%), 266 (24%), 242 (14%), 203 (10%), 126 (10%)

HRMS: (ESI$^+$)

Calcd for $C_{13}H_{20}(^{79}Br)N_2$: 283.0810, found: 283.0809

Data for **45** (contaminated with Bu_4NN_3)

^{1}H NMR: (400 MHz, $CDCl_3$)

8.47–8.44 (m, 1H, C(2″)*H*), 8.43–8.40 (m, 1H, C(6″)*H*), 7.52–7.48 (m, 1H, C(4″)*H*), 7.20–7.17 (m, 1H, C(5″)*H*), 2.67–2.63 (m, 2H, C(2′)H_2)

^{13}C NMR: (101 MHz, $CDCl_3$)

150.0 (*C*(2″)), 147.3 (*C*(6″)), 138.4 (*C*(3″)), 135.9 (*C*(4″)), 123.4 (*C*(5″)), 50.6 (*C*(1)), 44.5 (*C*(1′)), 38.8 (*C*(2), *C*(6)), 26.8 (*C*(2′)), 26.1 (*C*(4′)), 22.3 (*C*(3), *C*(5))

HRMS: (ESI$^+$)

Calcd for $C_{13}H_{21}N_2$: 205.1705, found: 205.1703

Preparation of 1-(2-(2-fluoropyridin-3-yl)ethyl)cyclohexan-1-amine (7at)

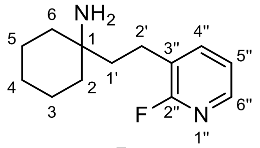

7at

Following ***General Procedure 1***, cyclohexylamine (**1a**) (52 µL, 45 mg, 0.45 mmol, 1.0 equiv), 2-fluoro-3-vinylpyridine (**6t**) (55 mg, 0.45 mmol, 1.0 equiv), 3DPA2FBN (2.80 mM in DMF, 1.60 mL, 4.5 µmol, 1 mol%), tetrabutylammonium azide (70.3 mM in DMF, 1.28 mL, 90 µmol, 20 mol%) and DMF (120 µL) were reacted for 20 h with a 425 nm LED lamp. The mixture was concentrated *in vacuo* and purified *via* flash column chromatography on silica gel (12 g) in CH_2Cl_2 (5 CV) then 100:0:0→95:4.5:0.5 CH_2Cl_2–MeOH–aq. NH_4OH (over 20 CV) then 95:4.5:0.5 CH_2Cl_2–MeOH–aq. NH_4OH (5 CV) to give **7at** as a brown oil (97.0 mg, 97%).

Data for **7at**:

^{1}H NMR: (400 MHz, $CDCl_3$)

8.06–7.89 (m, 1H, C(6″)*H*), 7.66–7.45 (m, 1H, C(4″)*H*), 7.11–6.95 (m, 1H, C(5″)*H*), 2.75–2.54 (m, 2H, C(2′)H_2), 1.63–1.29 (m, 14H, C(2)H_2, C(3)H_2, C(4)H_2, C(5)H_2, C(6)H_2, C(1′)H_2, NH_2)

^{13}C NMR: (101 MHz, $CDCl_3$)

162.1 (d, *J* = 238 Hz, *C*(2″)), 144.8 (d, *J* = 14.5 Hz, *C*(6″)), 140.7 (d, *J* = 6.1 Hz, *C*(4″)), 124.8 (d, *J* = 30.7 Hz, *C*(3″)), 121.5 (d, *J* = 4.2 Hz, *C*(5″)), 50.7 (*C*(1)), 42.3 (*C*(1′)), 38.6 (*C*(2), *C*(6)), 25.9 (*C*(4)), 22.8 (*C*(2′)), 22.2 (*C*(3), *C*(5))

^{19}F NMR: (376 MHz, $CDCl_3$)

–73.2

IR: (neat)

2925 (w), 2855 (w), 1635 (w), 1606 (w), 1576 (m), 1433 (m), 1369 (w), 1318 (w), 1275 (w), 1244 (m), 1196 (w), 1102 (w), 1069 (w), 869 (w), 804 (w), 789 (w), 764 (w)

MS: (ESI^+)

449 (12%), 326 (36%), 299 (19%), 282 (18%), 271 (25%), 249 (10%), 224 (14%), 223 ($[M+H]^+$, 100%), 206 (43%), 203 (32%)

HRMS: (ESI^+)

Calcd for $C_{13}H_{20}FN_2$: 223.1611, found: 223.1611

E. Application to Synthesis of Fingolimod and its Phosphonate Analogue

E.1. Synthesis of Fingolimod (4)

Preparation of Fingolimod (4)

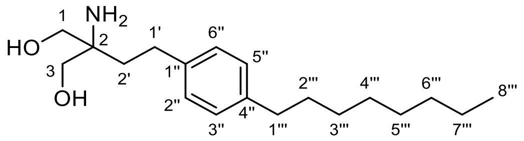

4

Following ***General Procedure 1***, serinol (**16**) (41 mg, 0.45 mmol, 1.0 equiv), 4-octylstyrene (**17**) (112 µL, 97 mg, 0.45 mmol, 1.0 equiv), 3DPA2FBN (2.80 mM in DMF, 1.60 mL, 4.5 µmol, 1 mol%), tetrabutylammonium azide (70.3 mM in DMF, 1.28 mL, 90 µmol, 20 mol%) and DMF (120 µL) were reacted for 20 h with a 425 nm LED lamp. The mixture was concentrated *in vacuo* and purified *via* flash column chromatography on silica gel (12 g) in CH_2Cl_2 (5 CV) then 100:0:0→95:4.5:0.5 CH_2Cl_2–MeOH–aq. NH_4OH (over 20 CV) then 95:4.5:0.5 CH_2Cl_2–MeOH–aq. NH_4OH (5 CV), followed by reversed-phase flash column chromatography on C18 silica gel (15.5 g) in 98:2 H_2O–MeOH (5 CV) then 98:2→0:100 H_2O–MeOH (over 15 CV) then MeOH (5 CV) to give **4** as a white solid (60.0 mg, 43%). The melting point and NMR spectroscopic data was in accordance with the literature.[11]

Data for **4**:

mp: 120–122 °C (MeOH) {121–124 °C}[11]

^{1}H NMR: (400 MHz, MeOD)
7.11 (d, *J* = 8.0 Hz, 2H, C(2″)*H*, C(6″)*H*), 7.05 (d, *J* = 8.0 Hz, 2H, C(3″)*H*, C(5″)*H*), 3.52 (d, *J* = 10.8 Hz, 2H, C(1)H_A, C(3)H_A), 3.45 (d, *J* = 10.8 Hz, 2H, C(1)H_B, C(3)H_B), 2.67–2.58 (m, 2H, C(1′)H_2), 2.57–2.52 (m, 2H, C(1‴)H_2), 1.69–1.63 (m, 2H, C(2′)H_2), 1.62–1.54 (m, 2H, C(2‴)H_2), 1.35–1.25 (m, 10H, C(3‴)H_2, C(4‴)H_2, C(5‴)H_2, C(6‴)H_2, C(7‴)H_2), 0.93–0.86 (m, 3H, C(8‴)H_3)

^{13}C NMR: (101 MHz, MeOD)
141.3 (*C*(1″) or *C*(4″)), 141.2 (*C*(1″) or *C*(4″)), 129.4 (*C*(2″), *C*(6″) or *C*(3″), *C*(5″)), 129.2 (*C*(2″), *C*(6″) or *C*(3″), *C*(5″)), 66.5 (*C*(1), *C*(3)), 56.8 (*C*(2)), 37.7 (*C*(2′)), 36.5 (*C*(1‴)), 33.0 (*C*(2‴) or *C*(6‴)), 32.8 (*C*(2‴) or *C*(6‴)), 30.6 (*C*(1′) or *C*(3‴) or *C*(4‴) or *C*(5‴)), 30.4 (*C*(1′) or *C*(3‴) or *C*(4‴) or *C*(5‴)), 30.3 (*C*(1′) or *C*(3‴) or *C*(4‴) or *C*(5‴)), 30.0 (*C*(1′) or *C*(3‴) or *C*(4‴) or *C*(5‴)), 23.7 (*C*(7‴)), 14.4 (*C*(8‴))

IR: (neat)

3313 (w), 2923 (m), 2853 (w), 2504 (w), 2464 (w), 2417 (w), 1987 (w), 1514 (w), 1476 (w), 1466 (w), 1454 (w), 1391 (w), 1377 (w), 1354 (w), 1313 (w), 1177 (w), 1133 (w), 1077 (w), 1017 (m), 966 (w), 908 (w), 804 (w), 783 (w), 758 (w), 722 (w)

MS: (ESI^+)

330 (16%), 309 (21%), 308 ($[M+H]^+$, 100%), 304 (11%), 282 (42%)

HRMS: (ESI^+)

Calcd for $C_{19}H_{34}NO_2$: 308.2590, found: 308.2592

E.2. Synthesis of Phosphonate Mimic (20) of Fingolimod Phosphate

E.2.1. Control Experiments

Preparation of (*RS*)-2-amino-4-(4-octylphenyl)butan-1-ol (18) and 2-amino-2-(4-octylphenethyl)-4-(4-octylphenyl)butan-1-ol (46)

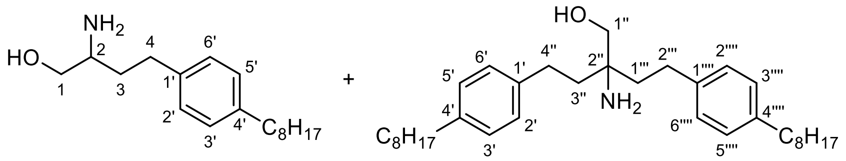

18 **46**

Following ***General Procedure 1***, ethanolamine (**1r**) (82 μL, 83 mg, 0.45 mmol, 1.0 equiv), 4-octylstyrene (17) (112 μL, 97 mg, 0.45 mmol, 1.0 equiv), 3DPA2FBN (2.80 mM in DMF, 1.60 mL, 4.5 μmol, 1 mol%), tetrabutylammonium azide (70.3 mM in DMF, 1.28 mL, 90 μmol, 20 mol%) and DMF (120 μL) were reacted for 20 h with a 425 nm LED lamp. The mixture was concentrated *in vacuo* and purified *via* flash column chromatography on silica gel (12 g) in CH_2Cl_2 (5 CV) then 100:0:0→95:4.5:0.5 CH_2Cl_2–MeOH–aq. NH_4OH (over 20 CV) then 95:4.5:0.5 CH_2Cl_2–MeOH–aq. NH_4OH (5 CV) to give **18** as a white solid (54.0 mg, 43%) and **46** as a white solid (49.2 mg, 22% wrt **1r**). The ^{13}C NMR data of **18** was in accordance with the literature.[12]

<u>Data for **18**:</u>

<u>mp:</u> 93–95 °C (CH_2Cl_2)

<u>^{1}H NMR:</u> (400 MHz, $CDCl_3$)
7.11–7.07 (m, 4H, C(2′)*H*, C(3′)*H*, C(5′)*H*, C(6′)*H*), 3.64–3.55 (m, 1H, C(1)H_A), 3.35–3.26 (m, 1H, C(1)H_B), 2.91–2.80 (m, 1H, C(2)*H*), 2.76–2.68 (m, 1H, C(4)H_A), 2.67–2.59 (m, 1H, C(4)H_B), 2.58–2.54 (m, 2H, C(1‴)H_2), 1.79–1.70 (m, 4H, C(3)H_A, O*H*, NH_2), 1.62–1.53 (m, 3H, C(3)H_B, C(2″)H_2), 1.34–1.24 (m, 10H, C(3″)H_2, C(4″)H_2, C(5″)H_2, C(6″)H_2, C(7″)H_2), 0.90–0.85 (m, 3H, C(8″)H_3)

<u>^{13}C NMR:</u> (101 MHz, $CDCl_3$)
140.7 (*C*(4′)), 139.0 (*C*(1′)), 128.6 (*C*(2′), *C*(6′) or (*C*(3′), *C*(5′)), 128.3 (*C*(2′), *C*(6′) or (*C*(3′), *C*(5′)), 67.0 (*C*(1)), 52.5 (*C*(2)), 36.5 (*C*(3)), 35.7 (*C*(1″)), 32.1 (*C*(2″) or *C*(6″)), 32.0 (*C*(2″) or *C*(6″)), 31.7 (*C*(4)), 29.6 (*C*(3″) or *C*(4″) or *C*(5″)), 29.5 (*C*(3″) or *C*(4″) or *C*(5″)), 29.4 (*C*(3″) or *C*(4″) or *C*(5″)), 22.8 (*C*(7″)), 14.3 (*C*(8″))

IR: (neat)

3344 (w), 2925 (w), 2855 (w), 1638 (w), 1567 (w), 1514 (w), 1466 (w), 1379 (w), 1309 (w), 1053 (w), 906 (w), 817 (w), 730 (m)

MS: (ESI^+)

494 (12%), 396 (20%), 279 (20%), 278 ($[M+H]^+$, 100%), 126 (11%)

HRMS: (ESI^+)

Calcd for $C_{18}H_{32}NO$: 278.2484, found: 278.2484

Data for **46**:

^{1}H NMR: (400 MHz, $CDCl_3$)

7.13–7.08 (m, 8H, C(2′)*H*, C(3′)*H*, C(5′)*H*, C(6′)*H*, C(2′′′′)*H*, C(3′′′′)*H*, C(5′′′′)*H*, C(6′′′′)*H*,), 3.43 (s, 2H, C(1′′)H_2), 2.65–2.54 (m, 8H, C(1)H_2, C(4′′)H_2, C(2′′′)H_2, C(1′′′′′)H_2), 1.85–1.77 (m, 2H, C(3′′)H_A, C(1′′′)H_A), 1.71–1.65 (m, 2H, C(3′′)H_B, C(1′′′)H_B), 1.64–1.54 (m, 5H, C(2)H_2, C(2′′′′′)H_2, O*H*), 1.42–1.18 (m, 22H, C(3)H_2, C(4)H_2, C(5)H_2, C(6)H_2, C(7)H_2, C(3′′′′′)H_2, C(4′′′′′)H_2, C(5′′′′′)H_2, C(6′′′′′)H_2, C(7′′′′′)H_2, NH_2), 0.91–0.86 (m, 6H, C(8)H_3, C(8′′′′′)H_3)

^{13}C NMR: (101 MHz, $CDCl_3$)

140.7 (*C*(1′), *C*(1′′′′) or *C*(4′), *C*(4′′′′)), 139.5 (*C*(1′), *C*(1′′′′) or *C*(4′), *C*(4′′′′)), 128.7 (*C*(2′), *C*(6′), *C*(2′′′′), *C*(6′′′′) or *C*(3′), *C*(5′), *C*(3′′′′), *C*(5′′′′)), 128.3 (*C*(2′), *C*(6′), *C*(2′′′′), *C*(6′′′′) or *C*(3′), *C*(5′), *C*(3′′′′), *C*(5′′′′)), 68.5 (*C*(1′′)), 55.3 (*C*(2′′)), 39.2 (*C*(3′′), *C*(1′′′)), 35.7 (*C*(1), *C*(1′′′′′)), 32.0 (*C*(2), *C*(2′′′′′) or *C*(6), *C*(6′′′′′)), 31.7 (*C*(2), *C*(2′′′′′) or *C*(6), *C*(6′′′′′)), 29.7 (*C*(3), *C*(3′′′′′) or *C*(4), *C*(4′′′′′) or *C*(5), *C*(5′′′′′) or *C*(4′′), *C*(2′′′)), 29.6 (*C*(3), *C*(3′′′′′) or *C*(4), *C*(4′′′′′) or *C*(5), *C*(5′′′′′) or *C*(4′′), *C*(2′′′)), 29.5 (*C*(3), *C*(3′′′′′) or *C*(4), *C*(4′′′′′) or *C*(5), *C*(5′′′′′) or *C*(4′′), *C*(2′′′)), 29.4 (*C*(3), *C*(3′′′′′) or *C*(4), *C*(4′′′′′) or *C*(5), *C*(5′′′′′) or *C*(4′′), *C*(2′′′)), 22.8 (*C*(7) or *C*(7′′′′′)), 14.3 (*C*(8) or *C*(8′′′′′))

IR: (neat)

3265 (w), 3017 (w), 2954 (w), 2923 (m), 2853 (w), 1584 (w), 1514 (w), 1456 (w), 1419 (w), 1377 (w), 1197 (w), 1118 (w), 1047 (w), 1021 (w), 910 (w), 816 (w), 722 (w)

MS: (ESI^+)

495 (27%), 494 ($[M+H]^+$, 53%), 345 (26%), 324 (18%), 323 (100%), 307 (20%), 295 (41%), 126 (37%)

HRMS: (ESI$^+$)

Calcd for $C_{34}H_{55}NNaO$: 516.4181, found: 516.4174

Preparation of (*RS*)-5-amino-2-ethoxy-5-(4-octylphenethyl)-1,2-oxaphosphinane 2-oxide (47)

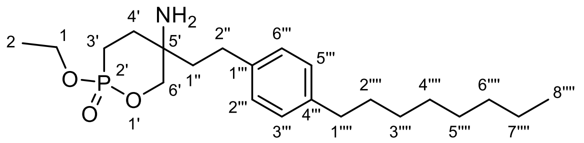

47

Following ***General Procedure 1***, (*RS*)-diethyl (3-amino-4-hydroxybutyl)phosphonate (**48**) (101 mg, 0.45 mmol, 1.0 equiv), 4-octylstyrene (**17**) (112 µL, 97 mg, 0.45 mmol, 1.0 equiv), 3DPA2FBN (2.80 mM in DMF, 1.60 mL, 4.5 µmol, 1 mol%), tetrabutylammonium azide (70.3 mM in DMF, 1.28 mL, 90 µmol, 20 mol%) and DMF (120 µL) were reacted for 20 h with a 425 nm LED lamp. The mixture was concentrated *in vacuo* and purified *via* flash column chromatography on silica gel (12 g) in CH_2Cl_2 (5 CV) then 100:0:0→95:4.5:0.5 CH_2Cl_2–MeOH–aq. NH_4OH (over 20 CV) then 95:4.5:0.5 CH_2Cl_2–MeOH–aq. NH_4OH (5 CV) to give **17** (60.4 mg, 62%) and **47** as an orange oil (22.1 mg, 12%).

Data for **47**:

^{1}H NMR: (400 MHz, $CDCl_3$)

7.18–7.00 (m, 4H, C(2‴)*H*, C(3‴)*H*, C(5‴)*H*, C(6‴)*H*), 4.34–4.06 (m, 2H, C(1)H_2), 4.07–3.96 (m, 1H, C(6′)H_A), 3.93–3.81 (m, 1H, C(6′)H_B), 2.74–2.52 (m, 4H, C(2″)H_2, C(1⁗)H_2), 2.19–1.67 (m, 6H, C(3′)H_2, C(4′)H_2, C(1″)H_2), 1.62–1.56 (m, 2H, C(2⁗)H_2), 1.39–1.24 (m, 15H, C(2)H_3, C(3⁗)H_2, C(4⁗)H_2, C(5⁗)H_2, C(6⁗)H_2, C(7⁗)H_2, NH_2), 0.89–0.85 (m, 3H, C(8⁗)H_3)

^{13}C NMR: (101 MHz, $CDCl_3$)

140.9 (*C*(4‴)), 138.7 (*C*(1‴)), 128.7 (*C*(3‴), *C*(5‴)), 128.3 (*C*(2‴), *C*(6‴)), 76.6 (d, *J* = 6.0 Hz, *C*(6′)), 61.6 (d, *J* = 6.5 Hz, *C*(1)), 50.7 (d, *J* = 5.5 Hz, *C*(5′)), 38.6 (*C*(1″)), 35.7 (*C*(1⁗)), 33.7 (d, *J* = 7.5 Hz, *C*(4′)), 32.0 (*C*(6⁗)), 31.7 (*C*(2⁗)), 29.6 (*C*(3⁗) or *C*(4⁗) or *C*(5⁗)), 29.5(*C*(3⁗) or *C*(4⁗) or *C*(5⁗)), 29.4 (*C*(3⁗) or *C*(4⁗) or *C*(5⁗)), 28.9 (*C*(2″)), 19.38 (d, *J* = 130 Hz, *C*(3′)), 16.62 (d, *J* = 5.9 Hz, *C*(2)), 14.3 (*C*(8⁗))

^{31}P NMR: (162 MHz, $CDCl_3$)
24.6

IR: (neat)
3368 (w), 2924 (w), 2854 (w), 1665 (w), 1514 (w), 1456 (w), 1415 (w), 1392 (w), 1378 (w), 1276 (w), 1240 (w), 1164 (w), 1097 (w), 1015 (m), 963 (w), 867 (w), 850 (w), 807 (w), 730 (w)

MS: (ESI^+)
450 (11%), 418 (19%), 397 (23%), 396 ($[M+H]^+$, 100%)

HRMS: (ESI^+)
Calcd for $C_{22}H_{39}NO_3P$: 396.2668, found: 396.2666

E.2.2. One-Pot Synthesis of Compound ***20****: Addition of Styrene* ***17*** *then Vinylphosphonate* ***19***

Preparation of diethyl (*RS*)-(3-amino-3-(hydroxymethyl)-5-(4-octylphenyl)pentyl)-phosphonate (20) and 2-amino-2-(4-octylphenethyl)-4-(4-octylphenyl)butan-1-ol (46)

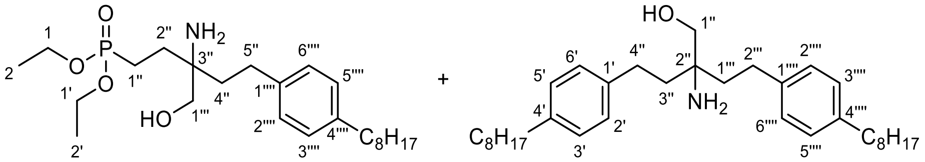

20 **46**

A 20-mL scintillation vial equipped with a stirrer bar was transferred to a nitrogen-filled purge box. The vial was charged with stock solutions of 3DPA2FBN (2.80 mM in DMF, 1.60 mL, 4.5 μmol, 1 mol%), tetrabutylammonium azide (70.3 mM in DMF, 1.28 mL, 90 μmol, 20 mol%) and additional anhydrous DMF (120 μL). Ethanolamine (**1r**) (82 μL, 83 mg, 0.45 mmol, 1.0 equiv) and 4-octylstyrene (**17**) (112 μL, 97 mg, 0.45 mmol, 1.0 equiv) were then transferred into the vial by microlitre syringe and the vial was sealed using a B24 rubber septa. It was then removed from the purge box and transferred to a photoreactor, and irradiated (with stirring) for 20 h at 425 nm. Fan cooling was used to maintain an external temperature of 25–26 °C. Following irradiation, the reaction mixture was returned to the nitrogen-filled purge box and 3DPA2FBN (2.80 mM in DMF, 1.60 mL, 4.5 μmol, 1 mol%) and vinylphosphonate (**19**) (70 μL, 74 mg, 0.45 mmol, 1.0 equiv) were added. It was then removed from the purge box and transferred to a photoreactor, and irradiated (with stirring) for 20 h at 425 nm. Fan cooling was used to maintain an external temperature of 25–26 °C. The reaction mixture was then concentrated *in vacuo* on a spiral evaporator and purified *via* flash column chromatography on silica gel (12 g) in CH_2Cl_2 (5 CV) then 100:0:0→95:4.5:0.5 CH_2Cl_2–MeOH–aq. NH_4OH (over

20 CV) then 95:4.5:0.5 CH_2Cl_2–MeOH–aq. NH_4OH (5 CV) to give **20** as an orange oil (44.0 mg, 22%) and **46** as a white amorphous solid (50.4 mg, 23% wrt **1r**).

Data for **20**:

^{1}H NMR: (400 MHz, $CDCl_3$)
7.10–7.06 (m, 4H, C(2′′′′)*H*, C(3′′′′)*H*, C(5′′′′)*H*, C(6′′′′)*H*), 4.16–4.04 (m, 4H, C(1)H_2, C(1′)H_2), 3.39 (s, 2H, C(1′′′)H_2), 2.61–2.50 (m, 4H, C(5′′)H_2, C(1′′′′′)H_2), 2.14 (br s, 3H, NH_2, O*H*), 1.84–1.55 (m, 8H, C(1′′)H_2, C(2′′)H_2, C(4′′)H_2, C(2′′′′′)H_2), 1.35–1.22 (m, 16H, C(2)H_3, C(2′)H_3, C(3′′′′′)H_2, C(4′′′′′)H_2, C(5′′′′′)H_2, C(6′′′′′)H_2, C(7′′′′′)H_2), 0.89–0.84 (m, 3H, C(8′′′′′)H_3)

^{13}C NMR: (101 MHz, $CDCl_3$)
140.7 (*C*(1′′′′) or *C*(4′′′′)), 139.2 (*C*(1′′′′) or *C*(4′′′′)), 128.6 (*C*(2′′′′), *C*(6′′′′) or *C*(3′′′′), *C*(5′′′′)), 128.2 (*C*(2′′′′), *C*(6′′′′) or *C*(3′′′′), *C*(5′′′′)), 68.0 (*C*(1′′′)), 61.9 (d, *J* = 6.5 Hz, *C*(1) or *C*(1′)), 61.9 (d, *J* = 6.5 Hz, *C*(1) or *C*(1′)), 55.1 (d, *J* = 14.7 Hz, *C*(3′′)), 38.8 (*C*(4′′)), 35.7 (*C*(1′′′′′)), 32.0 (*C*(6′′′′′)), 31.7 (*C*(2′′′′′)), 29.6 (*C*(3′′′′′) or *C*(4′′′′′) or *C*(5′′′′′)), 29.5 (*C*(3′′′′′) or *C*(4′′′′′) or *C*(5′′′′′)), 29.4 (*C*(3′′′′′) or *C*(4′′′′′) or *C*(5′′′′′)), 29.4 (*C*(5′′)), 29.1 (d, *J* = 4.6 Hz, *C*(2′′)), 22.8 (*C*(7′′′′′)), 20.0 (d, *J* = 142 Hz, *C*(1′′)), 16.6 (d, *J* = 6.0 Hz, *C*(2), *C*(2′)), 14.2 (*C*(8′′′′′))

^{31}P NMR: (162 MHz, $CDCl_3$)
33.2

IR: (neat)
3356 (w), 2925 (w), 2854 (w), 1597 (w), 1514 (w), 1456 (w), 1392 (w), 1215 (w), 1163 (w), 1097 (w), 1054 (m), 1024 (m), 961 (m), 830 (w), 790 (w), 721 (w)

MS: (ESI^+)
443 (28%), 442 ($[M+H]^+$, 100%)

HRMS: (ESI^+)
Calcd for $C_{24}H_{45}NO_4P$: 442.3086, found: 442.3090

E.2.3. One-Pot Synthesis of Compound ***20****: Addition of Vinylphosphonate* ***19*** *then Styrene* ***17***

Preparation of diethyl (*RS*)-(3-amino-3-(hydroxymethyl)-5-(4-octylphenyl)pentyl)-phosphonate (20) and (*RS*)-2-amino-4-(4-octylphenyl)butan-1-ol (18) and 2-amino-2-(4-octylphenethyl)-4-(4-octylphenyl)butan-1-ol (46)

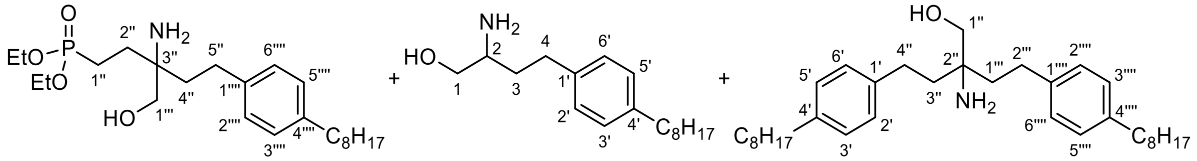

20 **18** **46**

A 20-mL scintillation vial equipped with a stirrer bar was transferred to a nitrogen-filled purge box. The vial was charged with stock solutions of 3DPA2FBN (2.80 mM in DMF, 1.60 mL, 4.5 μmol, 1 mol%), tetrabutylammonium azide (70.3 mM in DMF, 1.28 mL, 90 μmol, 20 mol%) and additional anhydrous DMF (120 μL). Ethanolamine (**1r**) (82 μL, 83 mg, 0.45 mmol, 1.0 equiv) and vinylphosphonate (**19**) (70 μL, 74 mg, 0.45 mmol, 1.0 equiv) were then transferred into the vial by microlitre syringe and the vial was sealed using a B24 rubber septa. It was then removed from the purge box and transferred to a photoreactor and irradiated (with stirring) for 20 h at 425 nm. Fan cooling was used to maintain an external temperature of 25–26 °C. Following irradiation, the reaction mixture was returned to the nitrogen-filled purge box and 3DPA2FBN (2.80 mM in DMF, 1.60 mL, 4.5 μmol, 1 mol%) and 4-octylstyrene (**17**) (112 μL, 97 mg, 0.45 mmol, 1.0 equiv) were added. It was then removed from the purge box and transferred to a photoreactor, and irradiated (with stirring) for 20 h at 425 nm. Fan cooling was used to maintain an external temperature of 25–26 °C. Following irradiation, the reaction mixture was concentrated *in vacuo* on a spiral evaporator and purified *via* flash column chromatography on silica gel (12 g) in CH_2Cl_2 (5 CV) then 100:0:0→95:4.5:0.5 CH_2Cl_2–MeOH–aq. NH_4OH (over 20 CV) then 95:4.5:0.5 CH_2Cl_2–MeOH–aq. NH_4OH (5 CV) to give an inseparable 66:34 mixture of **18** to **20** as an orange oil [86.9 mg, containing 48.4 mg of **18** (39%) and 38.5 mg of **20** (19%)] and **46** as a white solid (29.8 mg, 13%).

Data for **20**:

^{1}H NMR: (400 MHz, $CDCl_3$)

7.15–7.02 (m, 4H, C(2′′′′)*H*, C(3′′′′)*H*, C(5′′′′)*H*, C(6′′′′)*H*), 4.15–4.07 (m, 4H, C(1)H_2, C(1′)H_2), 3.39 (s, 2H, C(1′′′)H_2), 2.75–2.53 (m, 4H, C(5′′)H_2, C(1′′′′′)H_2), 1.89–1.56 (m, 11H, C(1′′)H_2, C(2′′)H_2, C(4′′)H_2, C(2′′′′′)H_2, O*H*, NH_2), 1.36–1.25 (m, 16H, C(2)H_3, C(2′)H_3, C(3′′′′′)H_2, C(4′′′′′)H_2, C(5′′′′′)H_2, C(6′′′′′)H_2, C(7′′′′′)H_2), 0.90–0.85 (m, 3H, C(8′′′′′)H_3)

<u>^{13}C NMR:</u> (101 MHz, $CDCl_3$)

140.8 (*C*(1′′′′) or *C*(4′′′′)), 139.2 (*C*(1′′′′) or *C*(4′′′′)), 128.7 (*C*(2′′′′), *C*(6′′′′) or *C*(3′′′′), *C*(5′′′′)), 128.2 (*C*(2′′′′), *C*(6′′′′) or *C*(3′′′′), *C*(5′′′′)), 68.0 (*C*(1′′′)), 61.9 (d, *J* = 6.5 Hz, *C*(1) or *C*(1′)), 61.9 (d, *J* = 6.5 Hz, *C*(1) or *C*(1′)), 55.1 (d, *J* = 14.4 Hz, *C*(3′′)), 38.9 (*C*(4′′)), 35.7 (*C*(1′′′′′)), 32.0 (*C*(6′′′′′)), 31.7 (*C*(2′′′′′)),, 29.6 (*C*(3′′′′) or *C*(4′′′′′), or *C*(5′′′′′)), 29.5 (*C*(3′′′′) or *C*(4′′′′′), or *C*(5′′′′′)), 29.4 (*C*(3′′′′) or *C*(4′′′′′) or *C*(5′′′′′)), 29.4 (*C*(5′′)), 29.1 (d, *J* = 4.9 Hz, *C*(2′′)), 22.8 (*C*(7′′′′′)), 20.0 (d, *J* = 142 Hz, *C*(1′′)), 16.6 (d, *J* = 6.0 Hz, *C*(2), *C*(2′)), 14.3 (*C*(8′′′′′))

<u>^{31}P NMR:</u> (162 MHz, $CDCl_3$)

33.2

<u>HRMS:</u> (ESI^+)

Calcd for $C_{24}H_{45}NO_4P$: 442.3086, found: 442.3087

<u>Data for **18**:</u>

<u>^{1}H NMR:</u> (400 MHz, $CDCl_3$)

7.15–7.02 (m, 4H, C(2′)*H*, C(3′)*H*, C(5′)*H*, C(6′)*H*), 3.67–3.56 (m, 1H, C(1)H_A), 3.35–3.25 (m, 1H, C(1)H_B), 2.91–2.79 (m, 1H, C(2)*H*), 2.75–2.53 (m, 4H, C(4)H_2, C(1′′)H_2), 1.89–1.56 (m, 7H, C(3)H_2, C(2′′)H_2, O*H*, NH_2), 1.36–1.25 (m, 10H, C(3′′)H_2, C(4′′)H_2, C(5′′)H_2, C(6′′)H_2, C(7′′)H_2), 0.90–0.85 (m, 3H, C(8′′)H_3)

<u>^{13}C NMR:</u> (101 MHz, $CDCl_3$)

140.7 (*C*(1′) or *C*(4′)), 138.9 (*C*(1′) or *C*(4′)), 128.6 (*C*(2′), *C*(6′) or *C*(3′),*C*(5′)), 128.3 (*C*(2′), *C*(6′) or *C*(3′),*C*(5′)), 66.9 (*C*(1)), 52.5 (*C*(2)), 36.4 (*C*(3)), 35.7 (*C*(1′′)), 32.1 (*C*(2′′) or *C*(6′′)), 32.0 (*C*(2′′) or *C*(6′′)), 37.1 (*C*(4)), 29.6 (*C*(3′′) or *C*(4′′), or *C*(5′′)), 29.5 (*C*(3′′) or *C*(4′′), or *C*(5′′)), 29.4 (*C*(3′′) or *C*(4′′), or *C*(5′′)), 22.8 (*C*(7′′), 14.3 (*C*(8′′))

<u>HRMS:</u> (ESI^+)

Calcd for $C_{18}H_{32}NO$: 278.2484, found: 278.2484

*E.2.4. One-Pot Synthesis of Compound **20**: Addition of Vinylphosphonate **19** and Styrene **17** Simultaneously*

Preparation of diethyl (*RS*)-(3-amino-3-(hydroxymethyl)-5-(4-octylphenyl)pentyl)-phosphonate (20)

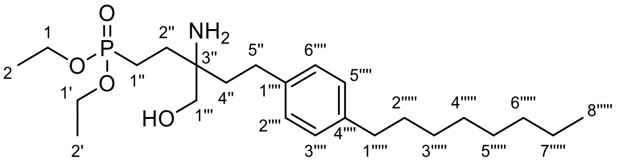

20

A 20-mL scintillation vial equipped with a stirrer bar was transferred to a nitrogen-filled purge box. The vial was charged with stock solutions of 3DPA2FBN (2.80 mM in DMF, 1.60 mL, 4.5 μmol, 1 mol%), tetrabutylammonium azide (70.3 mM in DMF, 1.28 mL, 90 μmol, 20 mol%) and additional anhydrous DMF (120 μL). Ethanolamine (**1r**) (82 μL, 83 mg, 0.45 mmol, 1.0 equiv), 4-octylstyrene (**17**) (112 μL, 97 mg, 0.45 mmol, 1.0 equiv) and vinylphosphonate (**19**) (70 μL, 74 mg, 0.45 mmol, 1.0 equiv) were then transferred into the vial by microlitre syringe and the vial was sealed using a B24 rubber septa. It was then removed from the purge box and transferred to a photoreactor and irradiated (with stirring) for 20 h at 425 nm. Fan cooling was used to maintain an external temperature of 25–26 °C. Following irradiation, the reaction mixture was concentrated *in vacuo* on a spiral evaporator and purified *via* flash column chromatography on silica gel (12 g) in CH_2Cl_2 (5 CV) then 100:0:0→95:4.5:0.5 CH_2Cl_2–MeOH–aq. NH_4OH (over 20 CV) then 95:4.5:0.5 CH_2Cl_2–MeOH–aq. NH_4OH (5 CV) to give **20** as an orange oil (25.1 mg, 13%).

F. Application to Synthesis of THQs and THNs

F.1. Synthesis of THQs (8)

Preparation of 3′,4′-dihydro-1′*H*-spiro[cyclohexane-1,2′-quinoline] (8ac)

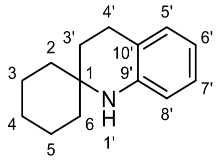

8ac

Following ***General Procedure 2***, **7ac** (0.40 mM in PhMe, 28 mg, 0.10 mmol, 1.0 equiv), NaO*t*-Bu (14 mg, 0.15 mmol, 1.5 equiv), $Pd_2(dba)_2$ (0.02 mM in PhMe, 250 µL, 5.0 µmol, 5 mol%), RuPhos (0.04 mM in PhMe, 250 µL, 10 µmol, 10 mol%) and anhydrous PhMe (250 µL) were reacted for 20 h at 110 °C. The mixture was passed though Celite, concentrated *in vacuo* and purified *via* flash column chromatography on silica gel (12 g) in 40-60° petroleum ether (5 CV) then 100:0→0:100 40-60° petroleum ether–EtOAc (over 20 CV) then EtOAc (10 CV) to give **8ac** as a brown oil (15.0 mg, 75%).

Data for **8ac**:

^{1}H NMR: (400 MHz, $CDCl_3$)
6.99 - 6.93 (m, 2H, C(5′)*H*, C(7′)*H*), 6.62–6.56 (m, 1H, C(6′)*H*), 6.51–6.45 (m, 1H, C(8′)*H*), 3.92 (br s, 1H, N*H*), 2.74 (t, *J* = 6.8 Hz, 2H, C(4′)H_2), 1.73 (t, *J* = 6.8 Hz, 2H, C(3′)H_2), 1.56–1.41 (m, 10H, C(2)H_2, C(3)H_2, C(4)H_2, C(5)H_2, C(6)H_2,)

^{13}C NMR: (101 MHz, $CDCl_3$)
143.9 (*C*(9′)), 129.4 (*C*(5′)), 126.8 (*C*(7′)), 120.7 (*C*(10′)), 116.6 (*C*(6′)), 114.3 (*C*(8′)), 50.3 (*C*(1)), 37.5 (*C*(2), *C*(6)), 32.5 (br s, *C*(3′)), 26.1 (*C*(4)), 23.5 (*C*(4′)), 22.0 (*C*(3), *C*(5))

IR: (neat)
3411 (w), 3013 (w), 2922 (m), 2849 (w), 2192 (w), 1605 (m), 1585 (w), 1500 (w), 1480 (m), 1462 (m), 1449 (m), 1345 (w), 1311 (m), 1275 (w), 1259 (w), 1169 (w), 1114 (w), 1092 (w), 1047 (w), 998 (w), 923 (w), 893 (w), 844 (w), 742 (s)

MS: (ESI^+)
203 (17%), 202 ($[M+H]^+$, 100%), 192 (14%)

HRMS: (ESI+)

Calcd for $C_{14}H_{20}N$: 202.1590, found: 202.1597

Preparation of (*RS*)-2-cyclohexyl-1,2,3,4-tetrahydroquinoline (8cc)

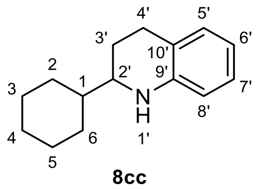

8cc

Following ***General Procedure 2***, **7cc** (0.35 mM in PhMe, 26 mg, 0.087 mmol, 1.0 equiv), NaO*t*-Bu (14 mg, 0.15 mmol, 1.7 equiv), $Pd_2(dba)_2$ (0.02 mM in PhMe, 250 µL, 5.0 µmol, 6 mol%), RuPhos (0.04 mM in PhMe, 250 µL, 10 µmol, 12 mol%) and anhydrous PhMe (250 µL) were reacted for 20 h at 110 °C. The mixture was passed though Celite, concentrated *in vacuo* and purified *via* flash column chromatography on silica gel (12 g) in 40-60° petroleum ether (5 CV) then 100:0→0:100 40-60° petroleum ether–EtOAc (over 20 CV) then EtOAc (10 CV), followed by reversed-phase flash column chromatography on C18 silica gel (15.5 g) in 98:2 H_2O–MeOH (5 CV) then 98:2→0:100 H_2O–MeOH (over 15 CV) then MeOH (5 CV) to give **8cc** as a yellow oil (11.6 mg, 61%). The NMR spectroscopic data was in accordance with the literature.[13]

Data for **8cc**:

^{1}H NMR: (400 MHz, $CDCl_3$)

7.02–6.88 (m, 2H, C(5′)*H*, C(7′)*H*), 6.65–6.52 (m, 1H, C(6′)*H*), 6.51–6.43 (m, 1H, C(8′)*H*), 3.81 (br s, 1H, N*H*), 3.09–2.98 (m, 1H, C(2′)*H*), 2.84–2.68 (m, 2H, C(4′)H_2), 1.95–1.88 (m, 1H, C(3′)H_A), 1.88–1.74 (m, 4H, C(2)H_A, C(6)H_A, C(3)H_B, C(5)H_B), 1.74–1.64 (m, 2H, C(4)H_A, C(3′)H_B), 1.42–1.33 (m, 1H, C(1)*H*), 1.29–0.99 (m, 5H, C(2)H_B, C(3)H_B, C(4)H_B, C(5)H_B, C(6)H_B)

^{13}C NMR: (101 MHz, $CDCl_3$)

145.1 (*C*(9′)), 129.3 (*C*(5′)), 126.8 (*C*(7′)), 121.6 (*C*(10′)), 116.8 (*C*(6′)), 114.1 (*C*(8′)), 56.7 (*C*(2′)), 42.6 (*C*(1)), 29.3 (*C*(2) or *C*(6)), 28.9 (*C*(2) or *C*(6)), 26.8 (*C*(3) or *C*(4) or *C*(5) or *C*(4′)), 26.7 (*C*(3) or *C*(4) or *C*(5) or *C*(4′)), 26.5 (*C*(3) or *C*(4) or *C*(5) or *C*(4′)), 26.5 (*C*(3) or *C*(4) or *C*(5) or *C*(4′)), 24.8 (*C*(3′))

IR: (neat)

3013 (w), 2921 (m), 2849 (w), 1607 (w), 1585 (w), 1481 (m), 1448 (w), 1374 (w), 1351 (w), 1310 (w), 1275 (w), 1253 (w), 1209 (w), 1188 (w), 1154 (w), 1109 (w), 1075 (w), 1034 (w), 1005 (w), 926 (w), 893 (w), 844 (w), 825 (w), 743 (m), 716 (w)

MS: (ESI^+)

304 (11%), 282 (26%), 217 (16%), 216 ($[M+H]^+$, 100%), 126 (18%)

HRMS: (ESI^+)

Calcd for $C_{15}H_{22}N$: 216.1725, found: 216.1753

Preparation of (*RS*)-2-methyl-2-phenethyl-1,2,3,4-tetrahydroquinoline (8ec)

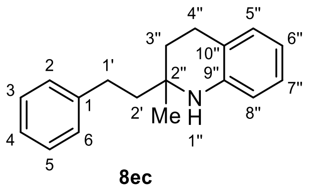

8ec

Following ***General Procedure 2***, **7ec** (0.40 mM in PhMe, 33 mg, 0.10 mmol, 1.0 equiv), NaO*t*-Bu (14 mg, 0.15 mmol, 1.5 equiv), $Pd_2(dba)_2$ (0.02 mM in PhMe, 250 µL, 5.0 µmol, 5 mol%), RuPhos (0.04 mM in PhMe, 250 µL, 10 µmol, 10 mol%) and anhydrous PhMe (250 µL) were reacted for 20 h at 110 °C. The mixture was passed though Celite, concentrated *in vacuo* and purified *via* flash column chromatography on silica gel (12 g) in 40-60° petroleum ether (5 CV) then 100:0→0:100 40-60° petroleum ether–EtOAc (over 20 CV) then EtOAc (10 CV) to give **8ec** as a yellow oil (19.0 mg, 76%).

Data for **8ec**:

^{1}H NMR: (400 MHz, $CDCl_3$)

7.30–7.26 (m, 2H, C(3)*H*, C(5)*H*), 7.21–7.16 (m, 3H, C(2)*H*, C(4)*H*, C(6)*H*), 7.01–6.95 (m, 2H, C(5″)*H*, C(7″)*H*), 6.64–6.59 (m, 1H, C(6″)*H*), 6.46–6.42 (m, 1H, C(8″)*H*), 3.65 (br s, 1H, N*H*), 2.82–2.77 (m, 2H, C(4″)H_2), 2.76 - 2.62 (m, 2H, C(1′)H_2), 1.88–1.69 (m, 4H, C(2′)H_2, C(3″)H_2), 1.26 (s, 3H, C(2″)*Me*)

^{13}C NMR: (101 MHz, $CDCl_3$)

143.9 (*C*(9″)), 142.6 (*C*(1)), 129.4 (*C*(5″)), 128.6 (*C*(2), *C*(6) or *C*(3), *C*(5)), 128.5 (*C*(2), *C*(6) or *C*(3), *C*(5)), 126.9 (*C*(7″)), 125.9 (*C*(4)), 120.3 (*C*(1)),

116.8 (C(6″)), 114.5 (C(8″)), 51.3 (C(2″)), 43.6 (C(2′)H_2), 32.5 (C(3″)), 30.4 (C(1′)), 26.9 (C(2″)*Me*), 24.0 (C(4″))

IR: (neat)

3382 (w), 3025 (w), 2922 (w), 2852 (w), 2365 (w), 2175 (w), 1606 (w), 1584 (w), 1484 (w), 1454 (w), 1375 (w), 1312 (w), 1274 (w), 1259 (w), 1154 (w), 1066 (w), 1041 (w), 839 (w), 742 (m), 712 (w), 697 (m)

MS: (ESI^+)

253 (21%), 252 ($[M+H]^+$, 100%)

HRMS: (ESI^+)

Calcd for $C_{18}H_{22}N$: 252.1747, found: 252.1751

Preparation of (1*RS*,2*RS*,4*SR*)-3',4'-dihydro-1'*H*-spiro[bicyclo[2.2.1]heptane-2,2'-quinoline] (8gc)

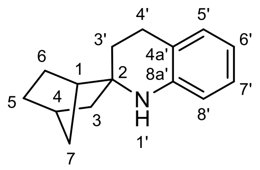

8gc

Following ***General Procedure 2***, **7gc** (0.40 mM in PhMe, 29 mg, 0.10 mmol, 1.0 equiv), NaO*t*-Bu (14 mg, 0.15 mmol, 1.5 equiv), $Pd_2(dba)_2$ (0.02 mM in PhMe, 250 μL, 5.0 μmol, 5 mol%), RuPhos (0.04 mM in PhMe, 250 μL, 10 μmol, 10 mol%) and anhydrous PhMe (250 μL) were reacted for 20 h at 110 °C. The mixture was passed though Celite, concentrated *in vacuo* and purified *via* flash column chromatography on silica gel (12 g) in 40-60° petroleum ether (5 CV) then 100:0→0:100 40-60° petroleum ether–EtOAc (over 20 CV) then EtOAc (10 CV) to give **8gc** as a yellow oil (10.0 mg, 47%).

Data for **8gc**:

^{1}H NMR: (400 MHz, $CDCl_3$)

6.99–6.92 (m, 2H, C(5′)*H*, C(7′)*H*), 6.62–6.55 (m, 1H, C(6′)*H*), 6.54–6.43 (m, 1H, C(8′)*H*), 3.90 (br s, 1H, N*H*), 2.85–2.68 (m, 2H, C(4′)H_2), 2.35–2.20 (m, C(4)*H*), 2.19–2.14 (m, 1H, C(1)*H*), 1.88–1.81 (m, 1H, C(3′)H_A), 1.79–1.61 (m, 5H, C(3′)H_B, C(3)H_A, C(5)H_A, C(6)H_A, C(7)H_A,), 1.47–1.39 (m, 1H, C(7)H_B), 1.32–1.26 (m, 2H, C(5)H_B, C(6)H_B), 1.10–1.05 (m, 1H, C(3)H_B)

^{13}C NMR: (101 MHz, $CDCl_3$)

144.7 (*C*(9′)), 129.2 (*C*(5′)), 126.7 (*C*(7′)), 120.8 (*C*(10′)), 116.6 (*C*(6′)), 114.1 (*C*(8′)), 58.4 (*C*(2)), 47.7 (*C*(3)), 45.5 (*C*(1)), 37.8 (*C*(6)), 37.1 (*C*(4)), 33.0 (*C*(3′)), 29.0 (*C*(5)), 24.4 (*C*(4′)), 22.8 (*C*(7))

IR: (neat)

3409 (w), 2949 (m), 2870 (w), 1606 (m), 1586 (w), 1486 (m), 1474 (m), 1459 (m), 1424 (w), 1342 (w), 1322 (w), 1310 (m), 1274 (w), 1262 (w), 1167 (w), 1122 (w), 1104 (w), 1074 (w), 1052 (w), 998 (w), 925 (w), 911 (w), 873 (w), 836 (w), 741 (s), 719 (w), 700 (w), 686 (w), 668 (w)

MS: (ESI$^+$)

215 (16%), 214 ([M+H]$^+$, 100%), 125 (11%),

HRMS: (ESI$^+$)

Calcd for $C_{15}H_{20}N$: 214.1590, found: 214.1596

Preparation of 2-((2*RS*,3*rs*,5*rs*,7*rs*)-adamantan-1-yl)-2-methyl-1,2,3,4-tetrahydro-quinoline (8hc)

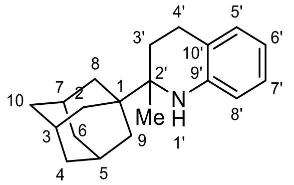

8hc

Following ***General Procedure 2***, **7hc** (0.40 mM in PhMe, 36 mg, 0.10 mmol, 1.0 equiv), NaO*t*-Bu (14 mg, 0.15 mmol, 1.5 equiv), $Pd_2(dba)_2$ (0.02 mM in PhMe, 250 µL, 5.0 µmol, 5 mol%), RuPhos (0.04 mM in PhMe, 250 µL, 10 µmol, 10 mol%) and anhydrous PhMe (250 µL) were reacted for 20 h at 110 °C. The mixture was passed though Celite, concentrated *in vacuo* and purified *via* flash column chromatography on silica gel (12 g) in 40-60° petroleum ether (5 CV) then 100:0→0:100 40-60° petroleum ether–EtOAc (over 20 CV) then EtOAc (10 CV) to give **8hc** as a yellow oil (20 mg, 71%).

Data for **8hc**:

^{1}H NMR: (400 MHz, $CDCl_3$)

7.07–6.86 (m, 2H, C(5″)*H*, C(7″)*H*), 6.64–6.52 (m, 1H, C(6′)*H*), 6.50–6.44 (m, 1H, C(8′)*H*), 3.76 (br s, 1H, N*H*), 2.83–2.64 (m, 2H, C(4′)H_2), 2.10–2.00 (m,

3H, C(3)*H*, C(5)*H*, C(7)*H*), 1.85–1.58 (m, 14H, C(3′)H_2, C(2)H_2, C(4)H_2, C(6)H_2, C(8)H_2, C(9)H_2, C(10)H_2), 1.08 (s, 3H, C(2′)*Me*)

^{13}C NMR: (101 MHz, $CDCl_3$)

145.0 (*C*(9′)), 128.9 (*C*(5′)), 126.9 (*C*(7′)), 121.2 (*C*(10′)), 116.3 (*C*(6′)), 114.4 (*C*(8′)), 55.8 (*C*(2′)), 38.8 (*C*(1)), 37.3 (*C*(4), *C*(6), *C*(10)), 36.0 (*C*(2), *C*(8), *C*(9)), 28.8 (*C*(3), *C*(5), *C*(7)), 26.5 (*C*(3′)), 24.3 (*C*(4′)), 20.0 (C(2′)*Me*)

IR: (neat)

2901 (m), 2847 (m), 1606 (w), 1586 (w), 1500 (w), 1479 (m), 1422 (w), 1370 (w), 1361 (w), 1347 (w), 1313 (m), 1259 (w), 1210 (w), 1190 (w), 1153 (w), 1105 (w), 1086 (w), 1059 (w), 1041 (w), 1009 (w), 992 (w), 970 (w), 928 (w), 837 (w), 817 (w), 793 (w), 742 (m), 700 (w), 667 (w)

MS: (ESI^+)

283 (21%), 282 ($[M+H]^+$, 100%), 126 (43%), 85 (12%)

HRMS: (ESI^+)

Calcd for $C_{20}H_{28}N$: 282.2216, found: 282.2221

Preparation of 2,3,3′,4′,5,6-hexahydro-1′*H*-spiro[pyran-4,2′-quinoline] (8ic)

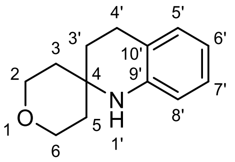

8ic

Following ***General Procedure 2***, **7ic** (0.40 mM in PhMe, 50 mg, 0.18 mmol, 1.0 equiv), NaO*t*-Bu (25 mg, 0.26 mmol, 1.5 equiv), $Pd_2(dba)_2$ (0.02 mM in PhMe, 437 µL, 8.7 µmol, 5 mol%), RuPhos (0.04 mM in PhMe, 437 µL, 18 µmol, 10 mol%) and anhydrous PhMe (437 µL) were reacted for 20 h at 110 °C. The mixture was passed though Celite, concentrated *in vacuo* and purified *via* flash column chromatography on silica gel (12 g) in 40-60° petroleum ether (5 CV) then 100:0→0:100 40-60° petroleum ether–EtOAc (over 20 CV) then EtOAc (10 CV) to give **8ic** as a white solid (18.8 mg, 53%).

Data for **8ic**:

mp: 102–104 °C (CH_2Cl_2)

<u>^{1}H NMR:</u> (400 MHz, $CDCl_3$)

7.04–6.92 (m, 2H, C(5′)*H*, C(7′)*H*), 6.67–6.59 (m, 1H, C(6′)*H*), 6.55–6.50 (m, 1H, C(8′)*H*), 3.98 (s, 1H, N*H*), 3.81–3.70 (m, 4H, C(2)H_2, C(6)H_2), 2.77 (t, *J* = 6.8 Hz, 2H, C(4′)H_2), 1.80 (t, *J* = 6.8 Hz, 2H, C(3′)H_2), 1.76–1.69 (m, 2H, C(3)H_A, C(5)H_A), 1.64–1.59 (m, 2H, C(3)H_B, C(5)H_B)

<u>^{13}C NMR:</u> (101 MHz, $CDCl_3$)

143.2 (*C*(9′)), 129.5 (*C*(5′)), 127.0 (*C*(7′)), 120.5 (*C*(10′)), 117.2 (*C*(6′)), 114.6 (*C*(8′)), 64.0 (*C*(2), *C*(6)), 48.2 (*C*(4)), 37.7 (*C*(3), *C*(5)), 32.4 (*C*(3′)), 23.1 (*C*(4′))

<u>IR:</u> (neat)

3366 (w), 3327 (m), 3015 (w), 2949 (w), 2917 (w), 2851 (w), 1605 (m), 1586 (w), 1491 (m), 1470 (m), 1440 (w), 1383 (w), 1355 (w), 1316 (m), 1291 (w), 1276 (w), 1258 (w), 1222 (w), 1192 (w), 1171 (w), 1143 (w), 1112 (w), 1097 (m), 1046 (w), 1035 (w), 1025 (w), 1014 (w), 993 (w), 939 (w), 907 (w), 862 (w), 842 (w), 832 (m), 743 (m), 711 (w)

<u>MS:</u> (ESI^+)

282 (11%), 205 (14%), 204 ($[M+H]^+$, 100%), 126 (21%)

<u>HRMS:</u> (ESI^+)

Calcd for $C_{13}H_{18}NO$: 204.1388, found: 204.1391

Preparation of *tert*-butyl 3′,4′-dihydro-1′*H*-spiro[piperidine-4,2′-quinoline]-1-carboxylate (8kc)

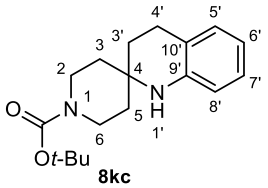

8kc

Following ***General Procedure 2***, **7kc** (0.40 mM in PhMe, 38 mg, 0.10 mmol, 1.0 equiv), NaO*t*-Bu (14 mg, 0.15 mmol, 1.5 equiv), $Pd_2(dba)_2$ (0.02 mM in PhMe, 250 μL, 5.0 μmol, 5 mol%), RuPhos (0.04 mM in PhMe, 250 μL, 10 μmol, 10 mol%) and anhydrous PhMe (250 μL) were reacted for 20 h at 110 °C. The mixture was passed though Celite, concentrated *in vacuo* and purified *via* flash column chromatography on silica gel (12 g) in 40-60° petroleum ether (5 CV) then 100:0→0:100 40-60° petroleum ether–EtOAc (over 20 CV) then EtOAc (10 CV) to give **8kc** as a yellow oil (19.6 mg, 65%).

Data for **8kc**:

^{1}H NMR: (400 MHz, $CDCl_3$)

7.01–6.95 (m, 2H, C(5′)*H*, C(7′)*H*), 6.66–6.59 (m, 1H, C(6′)*H*), 6.52–6.48 (m, 1H, C(8′)*H*), 3.88 (s, 1H, N*H*), 3.67–3.53 (m, 2H, C(2)H_A, C(6)H_A), 3.39–3.31 (m, 2H, C(2)H_B, C(6)H_B), 2.76 (t, *J* = 6.7 Hz, 2H, C(4′)H_2), 1.77 (t, *J* = 6.7 Hz, 2H, C(3′)H_2), 1.62–1.57 (m, 4H, C(3)H_2, C(5)H_2), 1.47 (s, 9H, OC(CH_3)$_3$)

^{13}C NMR: (101 MHz, $CDCl_3$)

155.0 (*C*=O), 143.2 (*C*(9′)), 129.5 (*C*(5′)), 127.0 (*C*(7′)), 120.5 (*C*(10′)), 117.3 (*C*(6′)), 114.6 (*C*(8′)), 79.8 (O*C*(CH_3)$_3$), 49.0 (*C*(4)), 39.7 (br s, *C*(2), *C*(6)), 36.6 (*C*(3), *C*(5)), 31.9 (*C*(3′)), 28.6 (*C*(4′)), 23.3 (OC(*C*H_3)$_3$)

IR: (neat)

3368 (w), 2975 (w), 2931 (w), 2852 (w), 1674 (m), 1607 (w), 1587 (w), 1481 (w), 1421 (m), 1393 (w), 1365 (w), 1313 (w), 1277 (w), 1262 (w), 1250 (m), 1221 (w), 1208 (w), 1156 (m), 1111 (w), 1096 (w), 1076 (w), 1044 (w), 1025 (w), 997 (w), 978 (w), 959 (w), 909 (w), 864 (w), 823 (w), 731 (m)

MS: (ESI$^+$)

304 (22%), 303 ($[M+H]^+$, 100%), 288 (29%), 86 (29%), 248 (12%), 247 (765)

HRMS: (ESI$^+$)

Calcd for $C_{18}H_{27}N_2O_2$: 303.2073, found: 303.2074

Preparation of *tert*-butyl 3′,4′-dihydro-1′*H*-spiro[azetidine-3,2′-quinoline]-1-carboxylate (8lc)

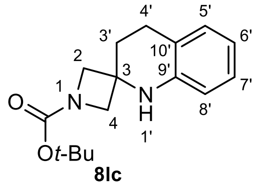

8lc

Following ***General Procedure 2***, **7lc** (0.40 mM in PhMe, 36 mg, 0.10 mmol, 1.0 equiv), NaO*t*-Bu (14 mg, 0.15 mmol, 1.5 equiv), $Pd_2(dba)_2$ (0.02 mM in PhMe, 250 µL, 5.0 µmol, 5 mol%), RuPhos (0.04 mM in PhMe, 250 µL, 10 µmol, 10 mol%) and anhydrous PhMe (250 µL) were reacted for 20 h at 110 °C. The mixture was passed though Celite, concentrated *in vacuo* and purified *via* flash column chromatography on silica gel (12 g) in 40-60° petroleum ether (5 CV) then 100:0→0:100 40-60° petroleum ether–EtOAc (over 20 CV) then EtOAc (10 CV) to give **8lc** as a yellow oil (6.7 mg, 24%).

Data for **8lc**:

^{1}H NMR: (400 MHz, $CDCl_3$)

7.03–6.95 (m, 2H, C(5′)*H*, C(7′)*H*), 6.69–6.62 (m, 1H, C(6′)*H*), 6.54–6.48 (m, 1H, C(8′)*H*), 4.18 (s, 1H, N*H*), 3.87 (d, *J* = 8.9 Hz, 2H, C(2)H_A, C(4)H_A), 3.81 (d, *J* = 8.9 Hz, 2H, C(2)H_B, C(4)H_B), 2.79 (t, *J* = 6.5 Hz, 2H, C(4′)H_2), 2.05 (t, *J* = 6.5 Hz, 2H, C(3′)H_2), 1.45 (s, 9H OC(CH_3)$_3$)

^{13}C NMR: (101 MHz, $CDCl_3$)

156.6 (*C*=O), 142.6 (*C*(9′)), 129.2 (*C*(5′)), 127.2 (*C*(7′)), 120.6 (*C*(10′)), 118.1 (*C*(6′)), 114.5 (*C*(8′)), 79.8 (O*C*(CH_3)$_3$), 63.4 (br s, *C*(2), *C*(4)), 50.8 (*C*(3)), 30.8 (*C*(3′)), 28.5 (OC(*C*H_3)$_3$), 23.8 (*C*(4′))

IR: (neat)

3352 (w), 2976 (w), 2939 (w), 2874 (w), 1683 (m), 1609 (w), 1589 (w), 1491 (m), 1475 (w), 1404 (m), 1366 (m), 1346 (w), 1321 (w), 1304 (w), 1279 (w), 1255 (w), 1220 (w), 1148 (m), 1116 (m), 1080 (m), 1039 (w), 911 (w), 858 (w), 770 (w), 749 (m), 732 (m), 707 (w)

MS: (ESI^+)

495 (20%), 494 (55%), 282 (11%), 220 (13%), 219 ([M–*t*-Bu+H]$^+$, 100%), 126 (12%)

HRMS: (ESI^+)

Calcd for $C_{16}H_{22}N_2NaO_2$: 297.1579, found: 297.1575

Preparation of (*RS*)-2-(3,3-diethoxypropyl)-1,2,3,4-tetrahydroquinoline (8mc)

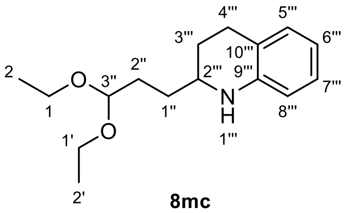

8mc

Following ***General Procedure 2***, **7mc** (0.40 mM in PhMe, 34 mg, 0.1 mmol, 1.0 equiv), NaO*t*-Bu (14 mg, 0.15 mmol, 1.5 equiv), $Pd_2(dba)_2$ (0.02 mM in PhMe, 250 µL, 5.0 µmol, 5 mol%), RuPhos (0.04 mM in PhMe, 250 µL, 10 µmol, 10 mol%) and anhydrous PhMe (250 µL) were reacted for 20 h at 110 °C. The mixture was passed though Celite, concentrated *in vacuo* and purified *via* flash column chromatography on silica gel (12 g) in 40-60° petroleum ether (5 CV)

then 100:0→0:100 40-60° petroleum ether–EtOAc (over 20 CV) then EtOAc (10 CV) to give **8mc** as a yellow oil (12.0 mg, 46%).

Data for **8mc**:

^{1}H NMR: (400 MHz, $CDCl_3$)
6.98–6.93 (m, 2H, C(5‴)*H*, C(7‴)*H*), 6.62–6.57 (m, 1H, C(6‴)*H*), 6.48–6.45 (m, 1H, C(8‴)*H*), 4.53–4.49 (m, 1H, C(3″)*H*), 3.88 (s, 1H, N*H*), 3.71–3.63 (m, 2H, C(1)H_2 or C(1′)H_2), 3.55–3.47 (m, 2H, C(1)H_2 or C(1′)H_2), 3.30–3.23 (m, 1H, C(2‴)H_2), 2.85–2.69 (m, 2H, C(4‴)H_2), 1.99–1.92 (m, 1H, C(4‴)H_A), 1.77–1.70 (m, 2H, C(2″)H_2), 1.64–1.55 (m, 3H, C(1″)H_2, C(4‴)H_B), 1.24–1.19 (m, 6H, C(2)H_3, C(2′)H_3)

^{13}C NMR: (101 MHz, $CDCl_3$)
144.8 (*C*(9‴)), 129.4 (*C*(5‴)), 126.9 (*C*(7‴)), 121.4 (*C*(10‴)), 117.1 (*C*(6‴)), 114.2 (*C*(8‴)), 103.1 (*C*(3″)), 61.4 (*C*(1) or *C*(1′)), 61.3 (*C*(1) or *C*(1′)), 51.5 (*C*(2‴)), 31.7 (*C*(1″)), 30.1 (*C*(2″)), 28.1 (*C*(3‴)), 26.5 (*C*(4‴)), 15.5 (*C*(2), *C*(2′))

IR: (neat)
3728 (w), 3377 (w), 2974 (w), 2926 (w), 2360 (w), 1607 (w), 1586 (w), 1485 (m), 1447 (w), 1372 (w), 1349 (w), 1310 (w), 1276 (w), 1207 (w), 1122 (m), 1055 (m), 1000 (m), 931 (w), 882 (w), 847 (w), 744 (m), 717 (w)

MS: (ESI^+)
265 (17%), 264 ($[M+H]^+$, 100%), 218 (50%), 172 (17%), 125 (14%)

HRMS: (ESI^+)
Calcd for $C_{16}H_{26}NO_2$: 264.1964, found: 264.1962

Preparation of (*RS*)-2-(methoxymethyl)-2-methyl-1,2,3,4-tetrahydroquinoline (8qc)

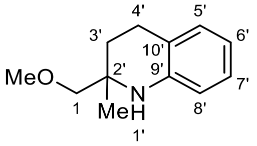

8qc

Following ***General Procedure 2***, **7qc** (0.40 mM in PhMe, 54 mg, 0.20 mmol, 1.0 equiv), NaO*t*-Bu (19 mg, 0.30 mmol, 1.5 equiv), $Pd_2(dba)_2$ (0.02 mM in PhMe, 500 µL, 10 µmol, 5 mol%), RuPhos (0.04 mM in PhMe, 500 µL, 20 µmol, 10 mol%) and anhydrous PhMe (500 µL) were

reacted for 20 h at 110 °C. The mixture was passed though Celite, concentrated *in vacuo* and purified *via* flash column chromatography on silica gel (12 g) in 40-60° petroleum ether (5 CV) then 100:0→0:100 40-60° petroleum ether–EtOAc (over 20 CV) then EtOAc (10 CV) to give **8qc** as a yellow oil (20.0 mg, 52%).

Data for **8qc**:

^{1}H NMR: (400 MHz, $CDCl_3$)
7.00–6.94 (m, 2H, C(5′)*H*, C(7′)*H*), 6.65–6.55 (m, 1H, C(6′)*H*), 6.51–6.45 (m, 1H, C(8′)*H*), 4.02 (br s, 1H, N*H*), 3.38 (s, 3H, O*Me*), 3.31 (d, *J* = 8.6 Hz, 1H, C(1)H_A), 3.21 (d, *J* = 8.6 Hz, 1H, C(1)H_B), 2.86–2.66 (m, 2H, C(4)H_2), 1.81–1.74 (m, 1H, C(3)H_A), 1.67–1.60 (m, 1H, C(3)H_B), 1.23 (s, 3H, C(2′)*Me*)

^{13}C NMR: (101 MHz, $CDCl_3$)
143.7 (*C*(9′)), 129.3 (*C*(5′)), 126.9 (*C*(7′)), 120.4 (*C*(10′)), 116.8 (*C*(6′)), 114.5 (*C*(8′)), 80.3 (*C*(1)), 59.6 (O*Me*), 51.7 (*C*(2′)), 30.2 (*C*(3′)), 24.5 (C(2′)*Me*), 23.6 (*C*(4′))

IR: (neat)
3396 (w), 2924 (w), 2878 (w), 1607 (w), 1586 (w), 1483 (m), 1453 (m), 1422 (w), 1386 (w), 1365 (w), 1343 (w), 1313 (m), 1275 (w), 1261 (w), 1194 (w), 1177 (w), 1155 (w), 1102 (s), 1041 (w), 1024 (w), 965 (w), 931 (w), 907 (w), 841 (w), 743 (s), 716 (m)

MS: (ESI^+)
193 (13%), 192 ($[M+H]^+$, 100%)

HRMS: (ESI^+)
Calcd for $C_{12}H_{18}NO$: 192.1383, found: 192.1387

Preparation of (*RS*)-2-(thiophen-2-ylmethyl)-1,2,3,4-tetrahydroquinoline (8uc)

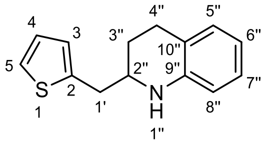

8uc

Following ***General Procedure 2***, **7uc** (0.40 mM in PhMe, 17 mg, 0.06 mmol, 1.0 equiv), NaO*t*-Bu (8 mg, 0.08 mmol, 1.5 equiv), $Pd_2(dba)_2$ (0.02 mM in PhMe, 139 μL, 2.8 μmol, 5 mol%), RuPhos (0.04 mM in PhMe, 139 μL, 5.3 μmol, 10 mol%) and anhydrous PhMe (139 μL) were reacted for 20 h at 110 °C. The mixture was passed though Celite, concentrated *in vacuo* and

purified *via* flash column chromatography on silica gel (12 g) in 40-60° petroleum ether (5 CV) then 100:0→0:100 40-60° petroleum ether–EtOAc (over 20 CV) then EtOAc (10 CV) to give **8uc** as a yellow oil (4 mg, 31%).

Data for **8uc**:

^{1}H NMR: (400 MHz, $CDCl_3$)
7.24–7.15 (m, 1H, C(5)*H*), 7.06–6.91 (m, 3H, C(4)*H*, C(5″)*H*, C(7″)*H*), 6.91–6.84 (m, 1H, C(3)*H*), 6.68–6.55 (m, 1H, C(6″)*H*), 6.51–6.38 (m, 1H, C(8″)*H*), 3.90 (br s, 1H, N*H*), 3.59–3.45 (m, 1H, C(2″)*H*), 3.10–3.00 (m, 1H, C(1′)H_A), 3.00–2.91 (m, 1H, C(1′)H_B), 2.90–2.73 (m, 2H, C(4″)H_2), 2.10–2.01 (m, 1H, C(3″)H_A), 1.78–1.68 (m, 1H, C(3″)H_B)

^{13}C NMR: (101 MHz, $CDCl_3$)
144.3 (*C*(2) or *C*(9″)), 141.1 (*C*(2) or *C*(9″)), 129.4 (*C*(5″)), 127.2 (*C*(4) or *C*(7″)), 126.9 (*C*(4) or *C*(7″)), 126.0 (*C*(2)), 124.4 (*C*(5)), 121.3 (*C*(10″)), 117.4 (*C*(6″)), 114.5 (*C*(8″)), 53.0 (*C*(2″)), 37.2 (*C*(1′)), 28.2 (*C*(3″)), 26.2 (*C*(4″))

IR: (neat)
3388 (w), 3014 (w), 2922 (w), 2842 (w), 1606 (m), 1585 (w), 1482 (m), 1433 (w), 1348 (w), 1309 (m), 1274 (w), 1251 (m), 1208 (w), 1170 (w), 1154 (w), 1141 (w), 1122 (w), 1077 (w), 1051 (w), 1035 (w), 1006 (w), 930 (w), 903 (w), 850 (w), 829 (w), 806 (w), 746 (m), 695 (m)

MS: (ESI^+)
494 (18%), 282 (10%), 231 (16%), 230 ($[M+H]^+$, 100%), 132 (15%)

HRMS: (ESI^+)
Calcd for $C_{14}H_{16}NS$: 230.1003, found: 230.1003

Preparation of 5′-chloro-3′,4′-dihydro-1′*H*-spiro[cyclohexane-1,2′-quinoline] (8an) and 3′,4′-dihydro-1′*H*-spiro[cyclohexane-1,2′-quinoline] (8ac)

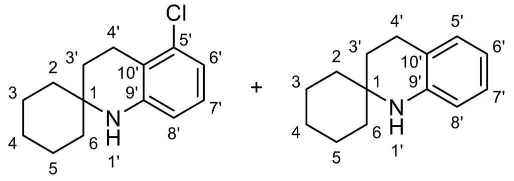

8an **8ac**

Following ***General Procedure 2***, **7an** (0.40 mM in PhMe, 32 mg, 0.10 mmol, 1.0 equiv), NaO*t*-Bu (14 mg, 0.15 mmol, 1.5 equiv), $Pd_2(dba)_2$ (0.02 mM in PhMe, 250 µL, 5.0 µmol, 5 mol%),

RuPhos (0.04 mM in PhMe, 250 μL, 10 μmol, 10 mol%) and anhydrous PhMe (250 μL) were reacted for 20 h at 110 °C. The mixture was passed though Celite, concentrated *in vacuo* and purified *via* flash column chromatography on silica gel (12 g) in 40-60° petroleum ether (5 CV) then 100:0→0:100 40-60° petroleum ether–EtOAc (over 20 CV) then EtOAc (10 CV) then purified further *via* flash column chromatography on silica gel (3.5 cm) in a Pasteur pipette in 98:2 40-60° petroleum ether–EtOAc to give an inseparable 75:25 mixture of **8an** to **8ac** as a brown oil [13.0 mg, containing 10.1 mg of **8an** (43%) and 2.9 mg of **8ac** (14%)].

Data for **8an**:

^{1}H NMR: (400 MHz, $CDCl_3$)
6.88 (dd, *J* = 8.0, 7.9 Hz, 1H, C(7′)*H*), 6.65 (dd, *J* = 7.9, 1.1 Hz, 1H, C(6′)*H*), 6.38 (dd, *J* = 8.0, 1.1 Hz, 1H, C(8′)*H*), 4.00 (br s, 1H, N*H*), 2.78–2.71 (m, 2H, C(4′)H_2), 1.77–1.71 (m, 2H, C(3′)H_2), 1.55–1.42 (m, 10H, C(2)H_2, C(3)H_2, C(4)H_2, C(5)H_2, C(6)H_2)

^{13}C NMR: (101 MHz, $CDCl_3$)
145.4 (*C*(9′)), 134.9 (*C*(5′)), 127.3 (*C*(7′)), 118.6 (*C*(10′)), 117.1 (*C*(6′)), 112.7 (*C*(8′)), 50.1 (*C*(1)), 37.0 (*C*(2), *C*(6)), 32.1 (*C*(3′)), 26.0 (*C*(4)), 22.1 (*C*(4′)), 21.6 (*C*(3), *C*(5))

HRMS: (ESI^+)
Calcd for $C_{14}H_{19}ClN$: 236.1201, found: 236.1206

Data for **8ac**:

^{1}H NMR: (400 MHz, $CDCl_3$)
7.02–6.91 (m, 2H, C(5′)*H*, C(7′)*H*), 6.61–6.56 (m, 1H, C(6′)*H*), 6.51–6.46 (m, 1H, C(8′)*H*), 4.00 (br s, 1H, N*H*), 2.78–2.71 (m, 2H, C(4′)H_2), 1.77–1.71 (m, 2H, C(3′)H_2), 1.55–1.42 (m, 10H, C(2)H_2, C(3)H_2, C(4)H_2, C(5)H_2, C(6)H_2)

^{13}C NMR: (101 MHz, $CDCl_3$)
143.9 (*C*(9′)), 129.4 (*C*(5′)), 126.8 (*C*(7′)), 120.7 (*C*(10′)), 116.6 (*C*(6′)), 114.3 (*C*(8′)), 50.3 (*C*(1)), 37.5 (*C*(2), *C*(6)), 32.5 (br s, *C*(3′)), 26.1 (*C*(4)), 23.5 (*C*(4′)), 22.0 (*C*(3), *C*(5))

HRMS: (ESI^+)
Calcd for $C_{14}H_{20}N$: 202.1590, found: 202.1595

F.2. Synthesis of THN (9at)

Preparation of 3′,4′-dihydro-1′*H*-spiro[cyclohexane-1,2′-[1,8]naphthyridine] (9at)

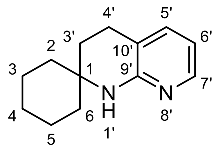

9at

A microwave vial equipped with a stirrer bar was charged with **7at** (44 mg, 0.20 mmol, 1.0 equiv), DIPEA (39 mg, 52 μL, 0.30 mmol, 1.5 equiv), anhydrous DMF (2 mL) and was capped with an aluminium crimp seal. The mixture was heated to 120 °C for 20 h, then concentrated *in vacuo* on a spiral evaporator and purified *via* flash column chromatography on silica gel (12 g) in 40-60° petroleum ether (5 CV) then 100:0→0:100 40-60° petroleum ether–EtOAc (over 20 CV) then EtOAc (10 CV) to give **9at** as a white, amorphous solid (37.2 mg, 92%).

Data for **9at**:

^{1}H NMR: (400 MHz, $CDCl_3$)
7.86 (dd, *J* = 5.0, 1.7 Hz, 1H, C(7′)*H*), 7.16 (dd, 1H, *J* = 7.2, 1.7 Hz, C(5′)*H*), 6.47 (dd, *J* = 7.2, 5.0 Hz, 1H, C(6′)*H*), 5.00 (s, 1H, N*H*), 2.70 (t, *J* = 6.7 Hz, 2H, C(4′)H_2), 1.70 (t, *J* = 6.7 Hz, 2H, C(3′)H_2), 1.59–1.35 (m, 10H, C(2)H_2, C(3)H_2, C(4)H_2, C(5)H_2, C(6)H_2)

^{13}C NMR: (101 MHz, $CDCl_3$)
155.7 (*C*(9′)), 146.2 (*C*(7′)), 136.2 (*C*(5′)), 115.6 (*C*(10′)), 112.5 (*C*(6′)), 51.2 (*C*(1)), 37.8 (*C*(2), *C*(6)), 32.4 (br s, *C*(3′)), 26.0 (*C*(4)), 22.9 (*C*(4′)), 21.9 (*C*(3), *C*(5))

IR: (neat)
3248 (w), 2927 (m), 2850 (w), 1601 (m), 1581 (w), 1518 (m), 1451 (m), 1435 (m), 1354 (w), 1342 (w), 1324 (w), 1291 (w), 1278 (w), 1263 (w), 1247 (w), 1233 (w), 1193 (w), 1178 (w), 1168 (w), 1125 (w), 1105 (w), 1092 (w), 912 (w), 899 (w), 760 (m), 732 (w)

MS: (ESI^+)
204 (15%), 203 ($[M+H]^+$, 100%)

HRMS: (ESI^+)
Calcd for $C_{13}H_{19}N_2$: 203.1548, found: 203.1547

G. Mechanistic Studies

G.1. Irreversibility of HAT Step

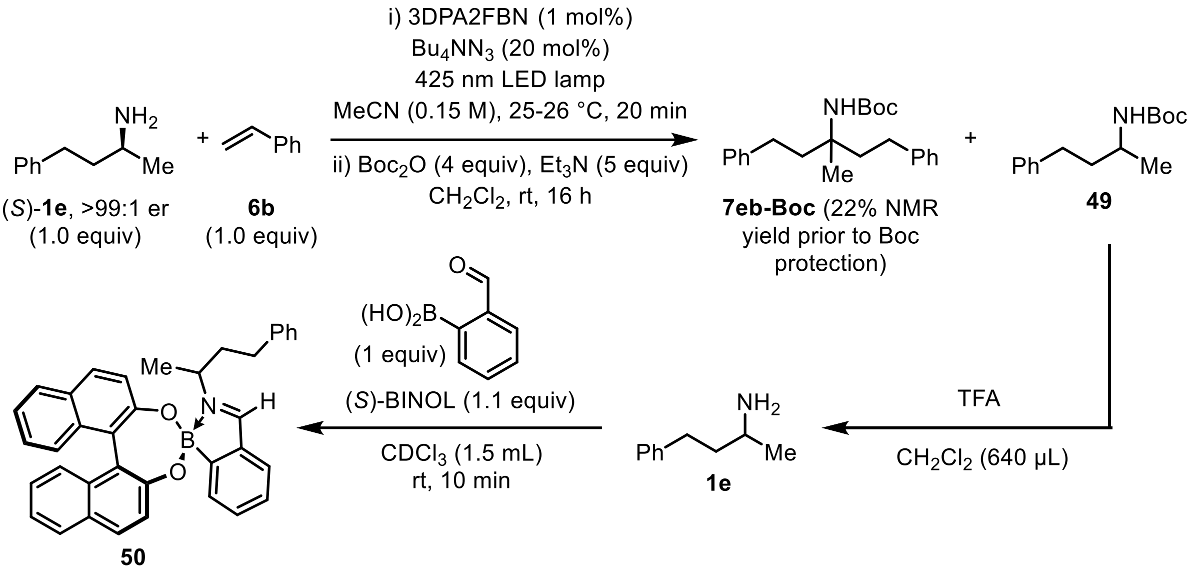

A 20-mL scintillation vial equipped with a stirrer bar was transferred to a nitrogen-filled purge box and charged with stock solutions of 3DPA2FBN (2.62 mM in MeCN, 1.72 mL, 4.5 µmol, 1 mol%) and tetrabutylammonium azide (70.3 mM in MeCN, 1.28 mL, 90 µmol, 20 mol%). (*S*)-(+)-1-Methyl-3-phenylpropylamine (*S*)-**1e** (71.7 µL, 0.45 mmol, 1.0 equiv) and styrene (51.6 µL, 0.45 mmol, 1.0 equiv) were added *via* microlitre syringe and the vial was sealed using a B24 rubber septa. It was then removed from the purge box and transferred to a photoreactor and irradiated (with stirring) for 20 min at 425 nm. Fan cooling was used to maintain an external temperature of 25–26 °C. Following irradiation, the mixture was concentrated *in vacuo* before subjection to ^{1}H NMR analysis. An NMR yield for **7eb** (22%) was calculated using the tetrabutylammonium ion as an internal standard, using the resonance at δH = 3.35 (8H, m) as a reference peak.

Di-*tert*-butyl dicarbonate (393 mg, 1.80 mmol, 4 equiv) and Et_3N (314 µL, 2.25 mmol, 5 equiv) were added to the crude mixture in CH_2Cl_2 (5.6 mL) and the resultant solution was stirred at rt for 16 h. The mixture was concentrated *in vacuo* and the crude material was purified *via* flash column chromatography on silica gel (12 g) in 40-60° petroleum ether (3 CV) then 100:0→60:40 40-60° petroleum ether–EtOAc (over 20 CV) then 60:40 petroleum ether – EtOAc →100% EtOAc (over 10 CV) to give **49** as an orange oil (>90% purity, 97.8 mg). After dissolution in CH_2Cl_2 (640 µL), trifluoroacetic acid (320 µL, 0.42 mmol) was added and the mixture was stirred at rt for 16 h. After concentration *in vacuo*, H_2O (12 mL) was added and

the mixture was extracted with CH_2Cl_2 (10 mL). The pH of the aqueous layer was adjusted to pH 12, then extracted with CH_2Cl_2 (3 × 10 mL) and the combined organic layers were concentrated *in vacuo* to give **1e** as a yellow oil (yield not determined).

Using the Bull-James procedure for enantiopurity determination of primary amines,[14] (*S*)-(−)-1,1′-Bi-2-naphthol (31.5 mg, 0.11 mmol, 1.1 equiv) was added to a stirred solution of 2-formylbenzene boronic acid (15 mg, 0.1 mmol, 1.0 equiv) in $CDCl_3$ (1.25 mL) at rt. **1e** (15.9 μL, 0.1 mmol, 1.0 equiv) was then transferred, followed by $CDCl_3$ (250 μL) and the mixture stirred at rt for 10 min. An aliquot (700 μL) of the reaction mixture was removed and subjected to ^1H NMR analysis (400 MHz).

A stacking of the various ^1H NMR spectra for complexes **50** prepared from different samples of amine **1e** (including the re-isolated sample above) are shown below (Figure 8). The peak shown in each case is the imine C–H proton in **50**.

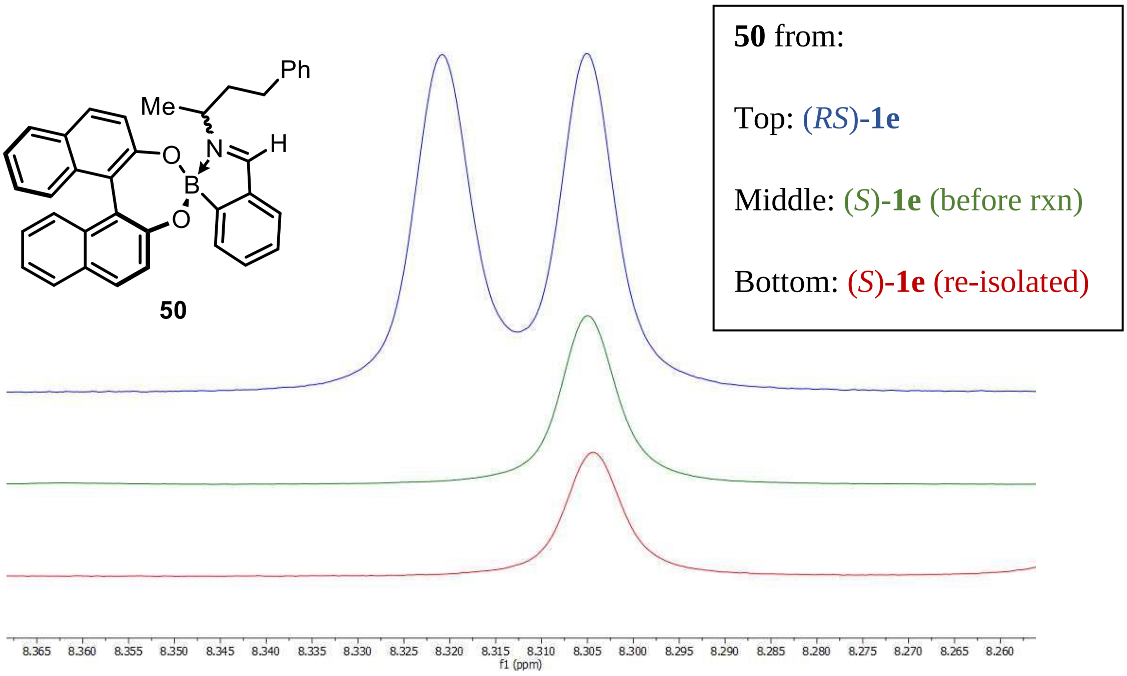

Figure 8. Stacked ^1H NMR spectra for complexes **50** (imine C–H proton region) derived from different samples of amine **1e**, including racemic **1e** (top), enantiopure (*S*)-**1e** (middle), and 1e re-isolated from the hydroaminoalkylation reaction (at 22% conversion to product **7eb**).

The conclusion that can be drawn from this experiment is that the amine (*S*)-**1e** is not subject to erosion of its enantiopurity under the conditions of our hydroaminoalkylation reaction, and so the HAT step of the mechanism must be irreversible under these conditions.

G.2. Variable Time Normalisation Analysis (VTNA)

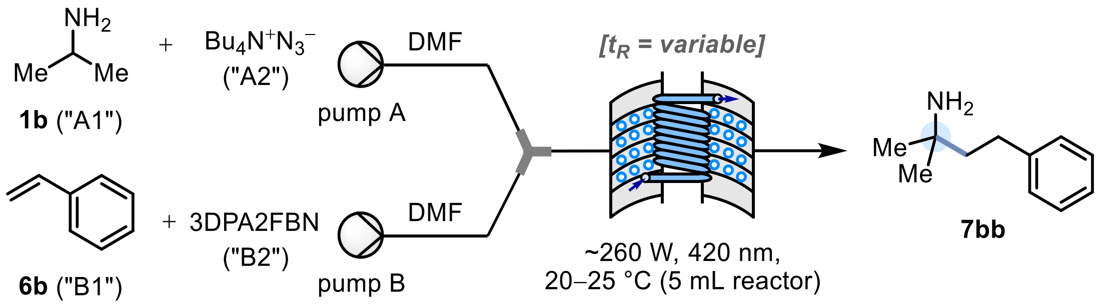

Concentrations at t_R = 0 (after mixing) (A1 and B1 determined by ¹H NMR against Bu_4N^+ ion):

**Run 1**	A1 = 0.151 M	B1 = 0.148 M	A2 = 30.0 mM	B2 = 1.50 mM
**Run 2**	A1 = 0.163 M	B1 = 0.146 M	A2 = 30.0 mM	B2 = 1.50 mM
**Run 3**	A1 = 0.136 M	B1 = 0.110 M	A2 = 30.0 mM	B2 = 1.50 mM
**Run 4**	A1 = 0.146 M	B1 = 0.147 M	A2 = 22.5 mM	B2 = 1.50 mM
**Run 5**	A1 = 0.154 M	B1 = 0.149 M	A2 = 30.0 mM	B2 = 1.25 mM

A Vapourtec RS-400 flow chemistry system and Uniqsis PhotoSyn reactor [LED array cooled with a Julabo FL1203 and reactor cooled with an Isotemp 250LCU] was powered up and the lamp and chillers were left to equilibrate for a minimum of 30 min. In a nitrogen purge box, reagent feeds A and B (*vide infra*) were prepared in 25-mL volumetric flasks, then transferred to 50-mL Schlenk flasks and sealed with rubber septa. Reagent lines for peristaltic pumps A and B were then primed with reagent feeds A and B and the system was purged with dry DMF. A series of automated reactions were then run as follows: portions of the two reagent feeds were injected simultaneously into the photoreactor at various flow rates, mixed in a T-mixer and passed through a 5-mL coil (0.8 mm inner diameter, fluoropolymer tube), irradiated with a 420 nm LED array (~260 W radiant output power) at 20–25 °C. The mixture was then passed through an acid-resistant BPR (6 bar), and 1.5 mL aliquots of the steady-state product mixture were collected to the autosampler. For each mixture, two 300 µL samples were removed and diluted with a solution of 1,3,5-trimethoxybenzene (200 µL, ~0.15 M in MeCN-d_3). D_2O (25 µL) was added to suppress N–H peaks and the samples were subjected to ¹H NMR analysis (400 MHz, with NOESY presaturation). Concentrations of each component were calculated against 1,3,5-trimethoxybenzene using the following resonances:

Component	Resonance (ppm)
1,3,5-trimethoxybenzene	δ = 6.07 (s, 3H)
styrene **6b**	δ = 5.21 (d, 1H)
isopropylamine **1b**	δ = 0.96 (d, 6H)
4-phenyl-2-methylbutan-2-amine **7bb**	δ = 1.06 (s, 6H)

VTNA plots from the data collected above are illustrated below. The data suggest that the reaction is first order in styrene **6b** (a) and zero order in all other components (b–d).

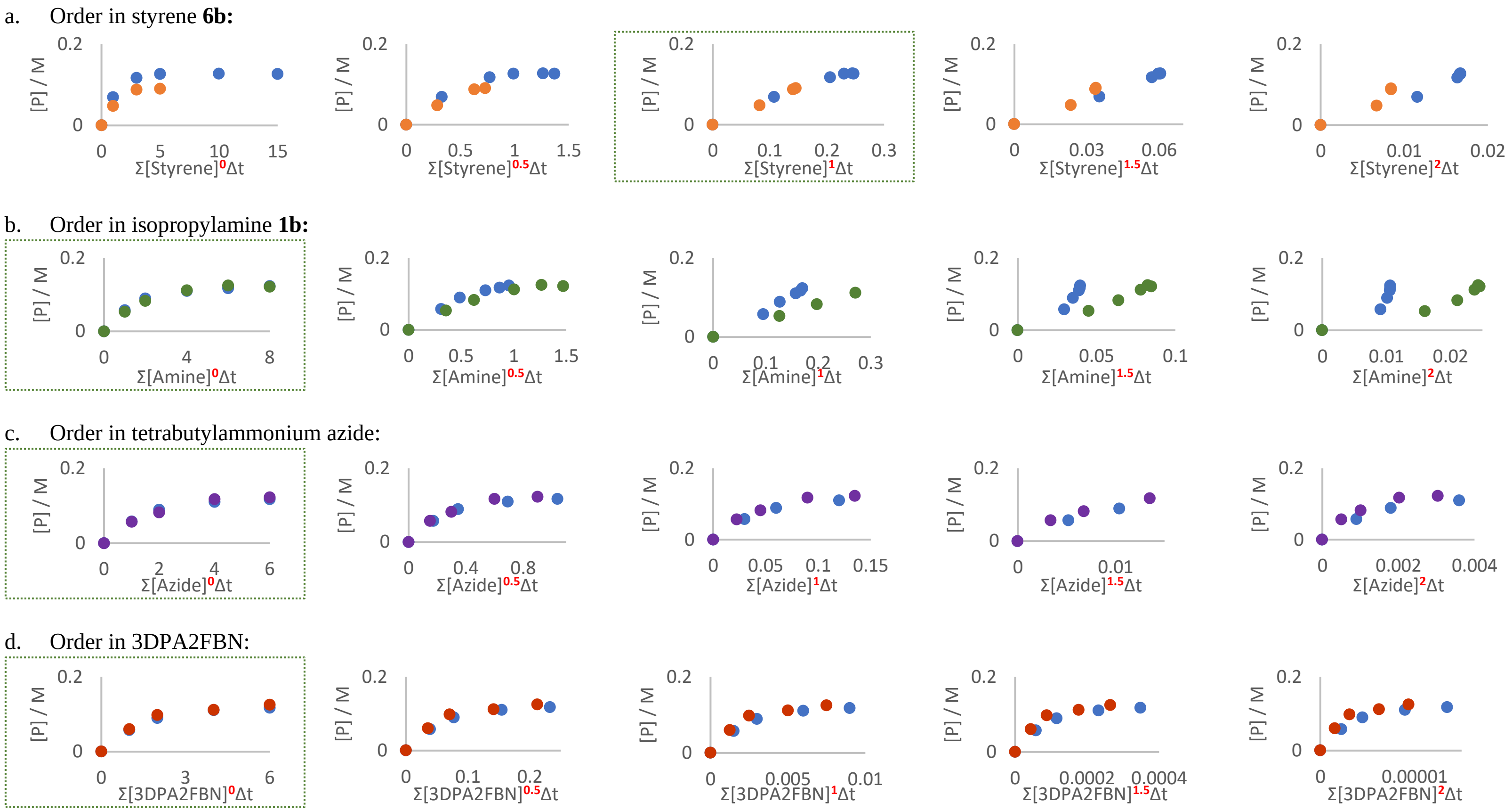

Figure 9. VTNA plots from the data collected by variable residence time continuous flow experiments

G.3. Stern-Volmer Luminescence Quenching Analysis

In a nitrogen-filled purge box, stock solutions of 3DPA2FBN (0.20 mM in DMF), tetrabutylammonium azide (1.50 mM in DMF), cyclohexylamine (**1a**) (100 mM in DMF), and styrene (**6b**) (100 mM in DMF) were prepared. For each possible quencher, solutions with constant 3DPA2FBN concentration (0.015 mM) but varying quencher concentration were prepared and analysed in 10 × 10 mm quartz cuvettes, equipped with a PTFE lid and sealed with parafilm. The emission spectra were recorded using an Agilent Cary Eclipse fluorimeter, equipped with a Xenon pulse lamp, pulse width of approximately 2–3 μs, peak power equivalent to 60–75 kW. The excitation wavelength was fixed at 380 nm while the emission light was acquired from 400 nm to 650 nm (emission light slit regulated to 5 nm, scan rate of 250 nm min^{-1}). Emission intensity at λ = 490 nm was used for quenching data.

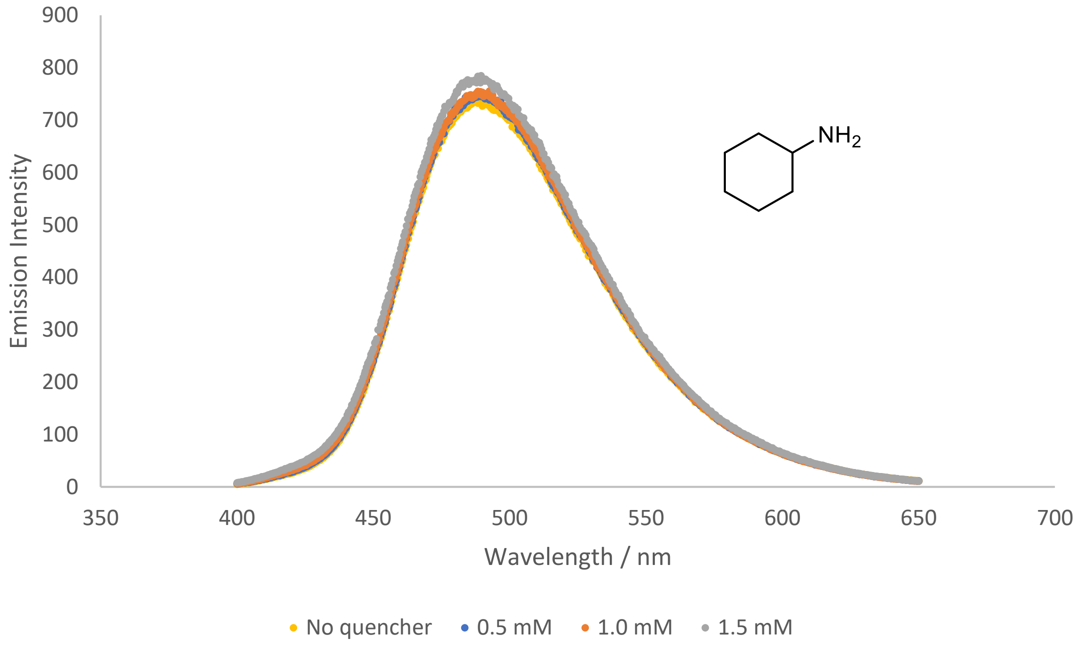

Figure 10. Stacked emission spectra of 3DPA2FBN (0.015 mM in DMF) at different concentrations of cyclohexylamine (**1a**)

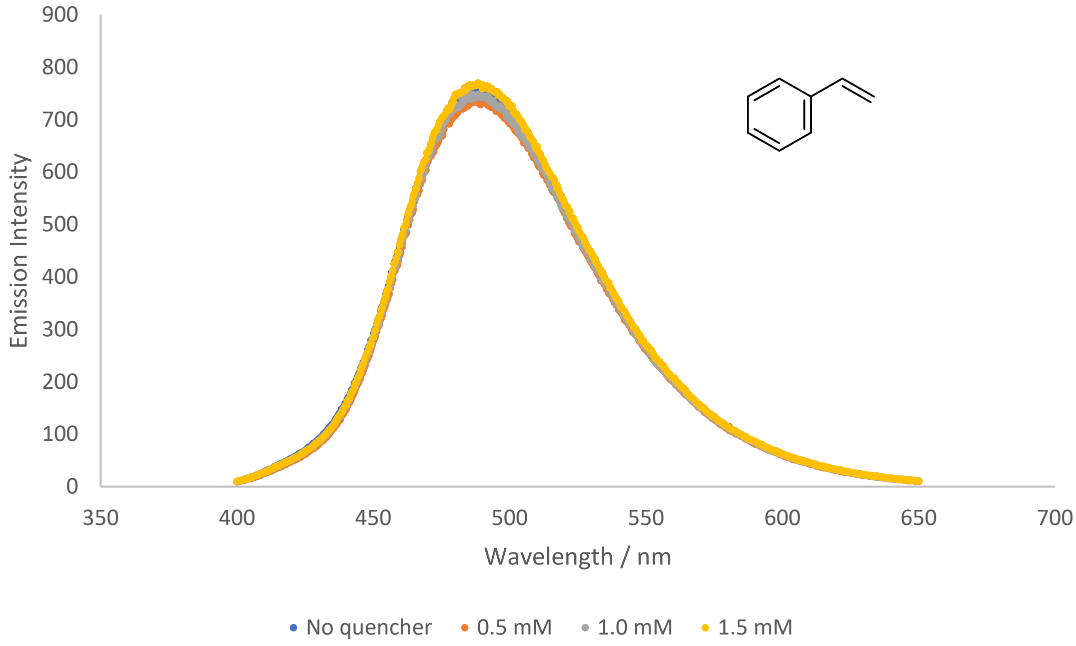

Figure 11. Stacked emission spectra of 3DPA2FBN (0.015 mM in DMF) at different concentrations of styrene (**6b**).

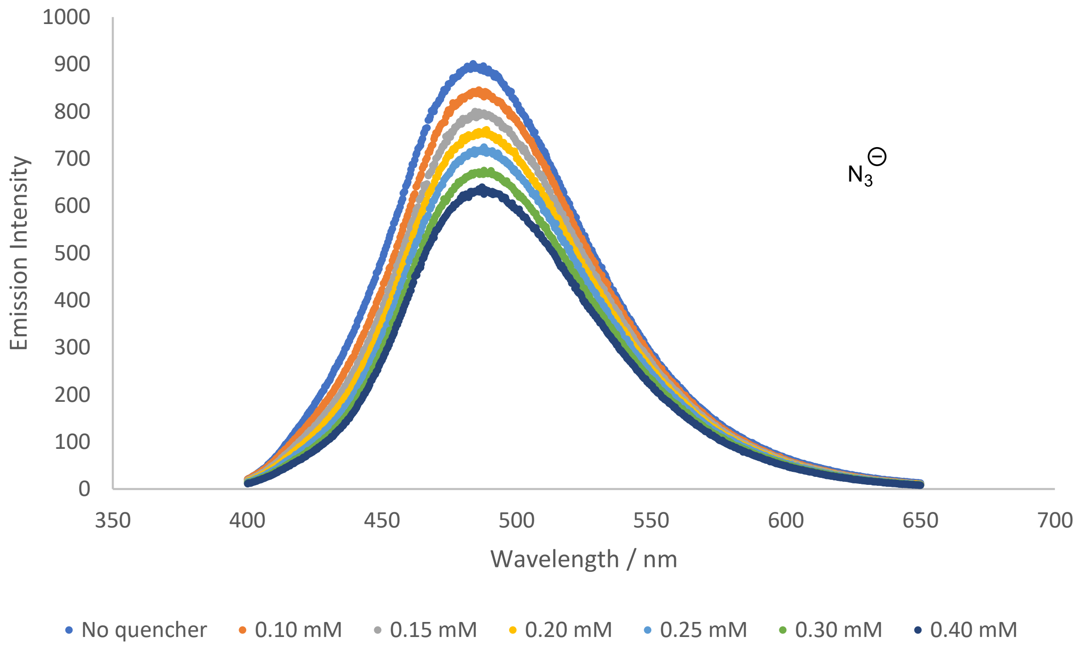

Figure 12. Stacked emission spectra of 3DPA2FBN (0.015 mM in DMF) at different concentrations of tetrabutylammonium azide.

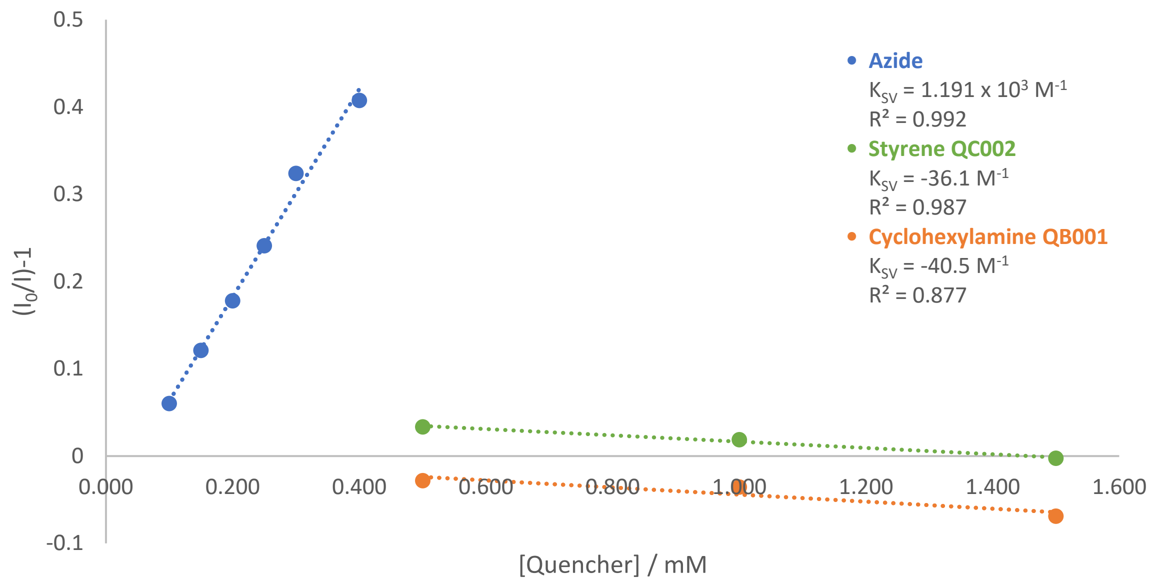

Figure 13. Stern-Volmer plot for the quenching studies above. Emission wavelength fixed at 490 nm. Cyclohexylamine (**1a**) and styrene (**6b**) display no ability to quench the fluorescence of 3DPA2FBN, whilst tetrabutylammonium azide is a much more effective quencher, with the Stern-Volmer constant (K_{SV}) for the quenching by N_3^- being 1.191×10^3 M^{-1}.

G.4. Quantum Yield Measurement

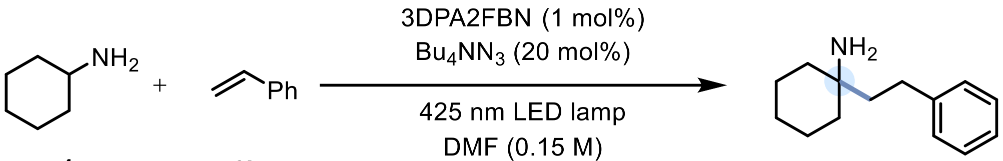

1a (1.0 equiv) **6b** (1.0 equiv) **7ab**

The quantum yield was measured for the reaction of cyclohexylamine (**1a**) with styrene (**6b**). The photon flux of our precise LED set up (with the same Evoluchem 18W LED lamp and identical reactor vial positioning) had been previously determined to be 5.22×10^{-7} mol s^{-1}.[10]

A 20 mL scintillation vial equipped with a stirrer bar was transferred to a nitrogen-filled purge box. The vial was then charged with stock solutions of 3DPA2FBN (2.80 mM in DMF, 1.07 mL, 3.0 µmol, 1 mol%), tetrabutylammonium azide (70.3 mM in DMF, 853 µL, 60 µmol, 20 mol%) and made up to a total volume of 2.0 mL by addition of anhydrous DMF. Cyclohexylamine **1a** (0.3 mmol, 34.4 µL, 1.0 equiv) was then transferred to the vial by microlitre syringe. Finally, styrene **6b** (0.3 mmol, 34.4 µL, 1.0 equiv) was added to the vial and the vial was sealed using a B24 rubber septa. It was then removed from the purge box and transferred to a Evoluchem PhotoRedOx Box reactor equipped with a Evoluchem 18W LED lamp (λ_{max}= 425 nm) where it was irradiated (with stirring) for 20 min. Fan cooling was used to maintain an external temperature of 25–26 ˚C. Following irradiation, the reaction mixture was concentrated *in vacuo* on a spiral evaporator before subjection to ^{1}H NMR analysis. An NMR yield for **7ab** (66%) was calculated using the tetrabutylammonium ion as an internal standard, using the resonance at δH = 3.35 (8H, m) as a reference peak.

The quantum yield (Φ) was then calculated using:

$$\Phi = \frac{mol\, product}{photon\, flux \times t \times f}$$

where t is the time (1200 s) and f is the fraction of light absorbed by the 4CzIPN catalyst at λ = 425 nm, where $f = 1–10^{-A}$ (for a 1.5×10^{-3} M solution in DMF, $A = 1.00$).

$$\Phi = \frac{1.97 \times 10^{-4}}{5.22 \times 10^{-7} \times 1200 \times 1} = 0.31$$

The significance of this result is addressed in the main manuscript.

H. ^{1}H and ^{13}C{^{1}H} NMR Spectra

^{1}H NMR (400 MHz, $CDCl_3$)

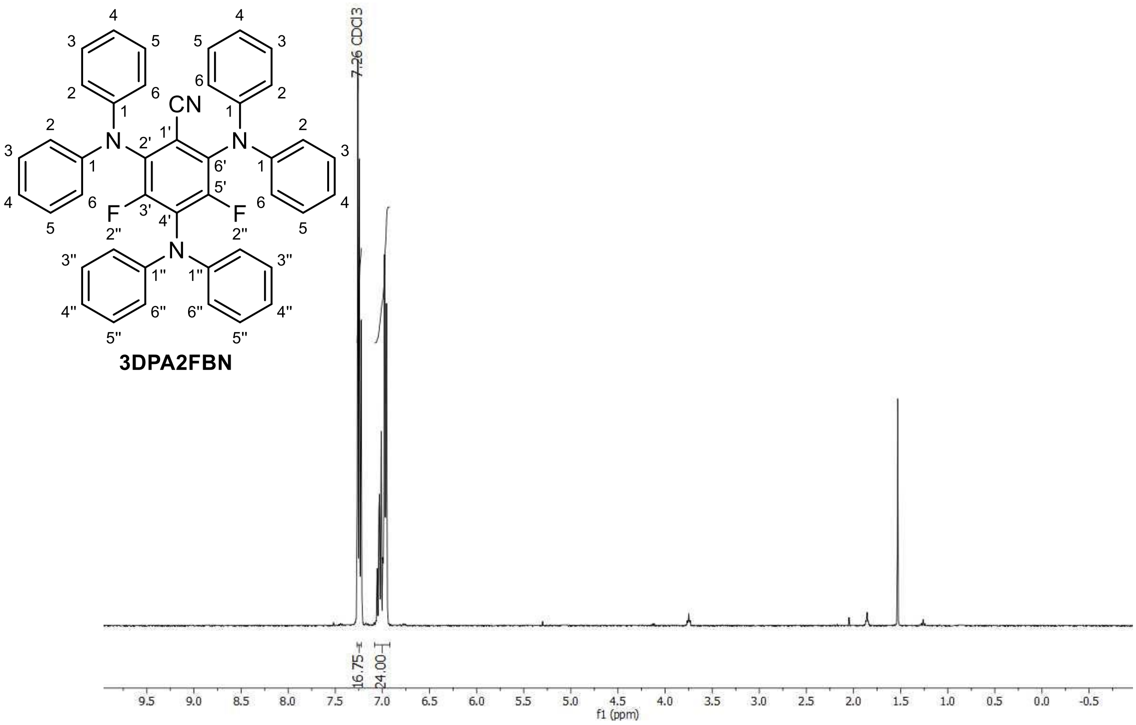

^{13}C{^{1}H} NMR (126 MHz, $CDCl_3$)

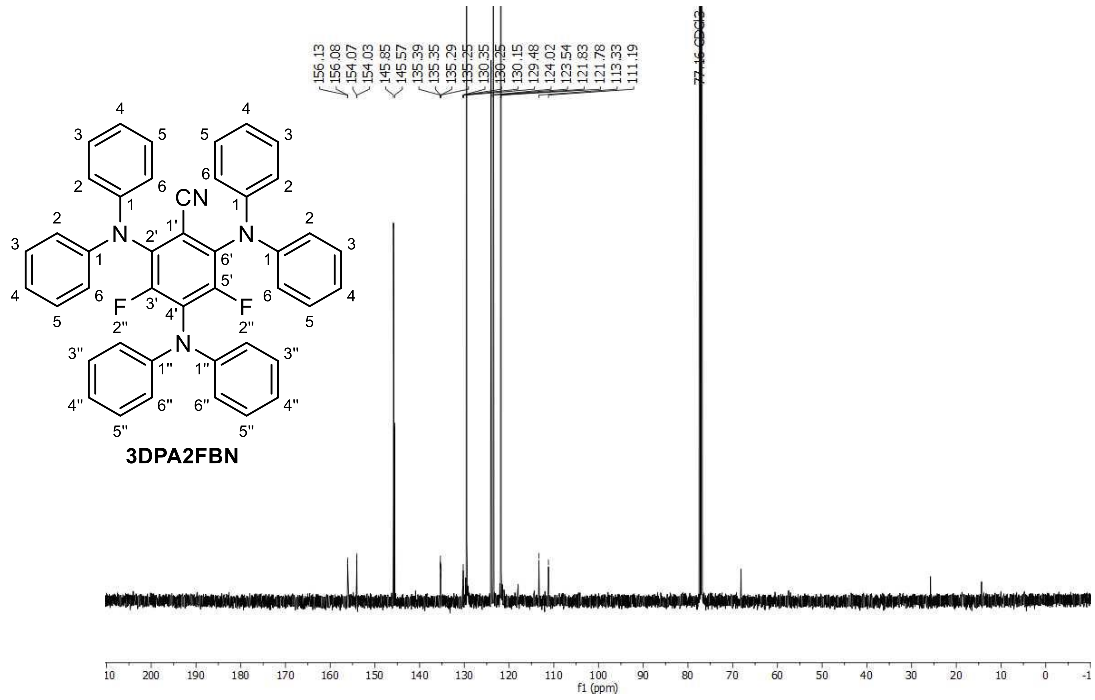

^{1}H NMR (400 MHz, CDCl$_3$)

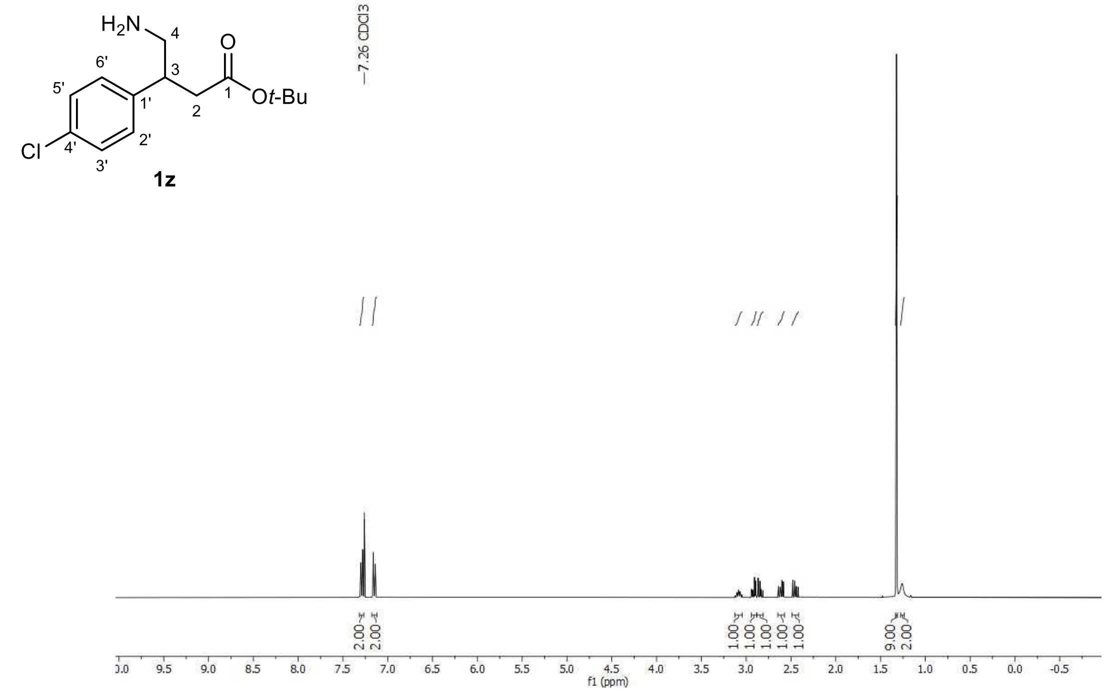

^{13}C{^{1}H} NMR (101 MHz, CDCl$_3$)

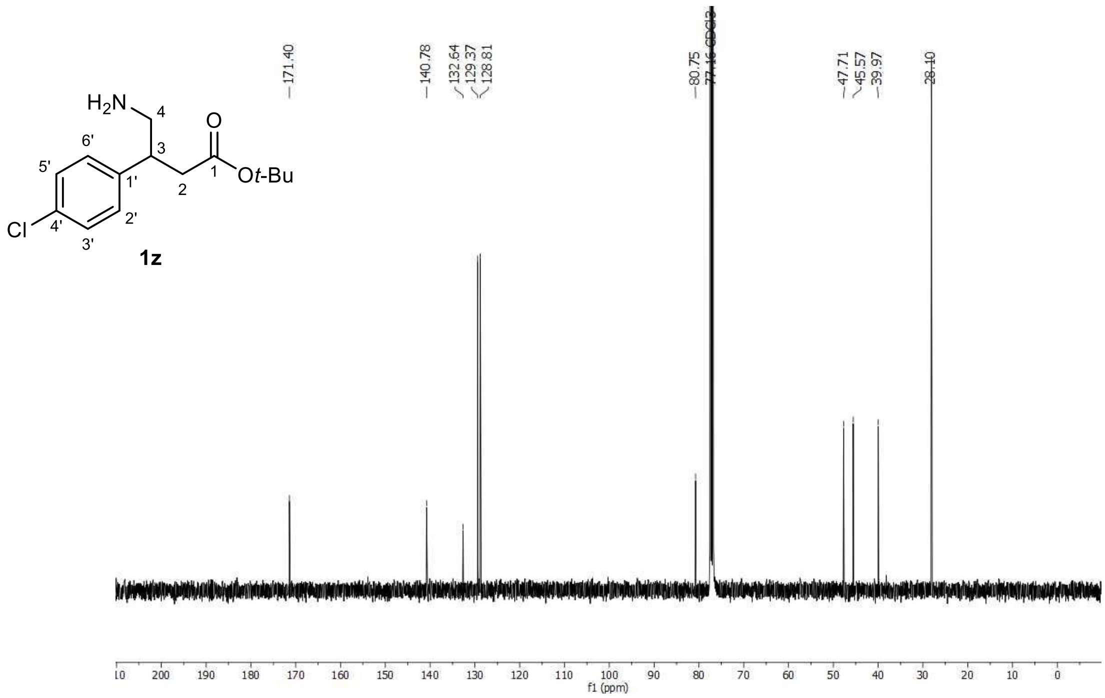

^{1}H NMR (500 MHz, $CDCl_3$)

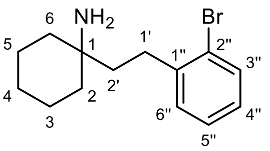

7ac

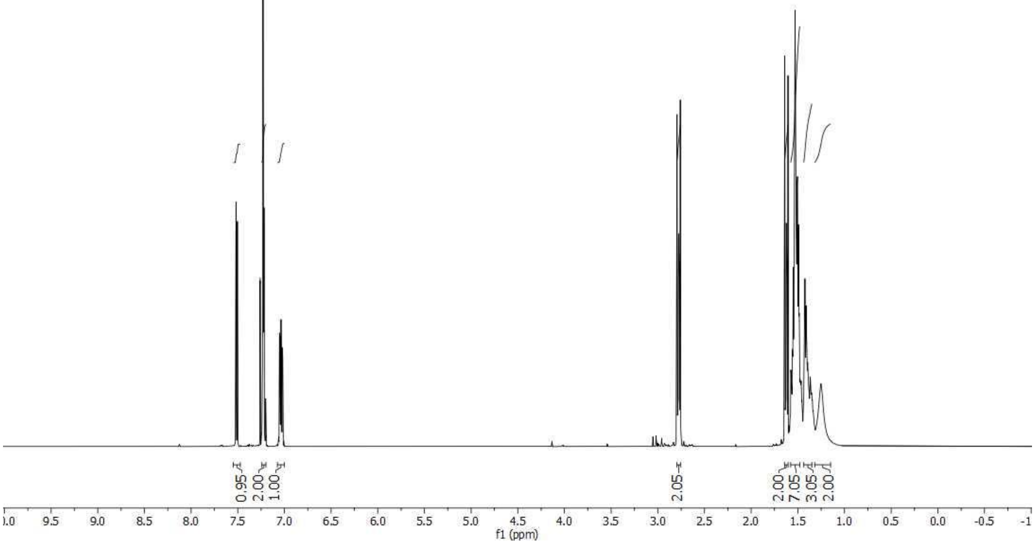

^{13}C{^{1}H} NMR (101 MHz, $CDCl_3$)

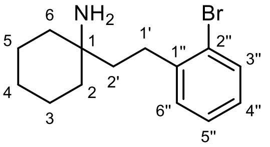

7ac

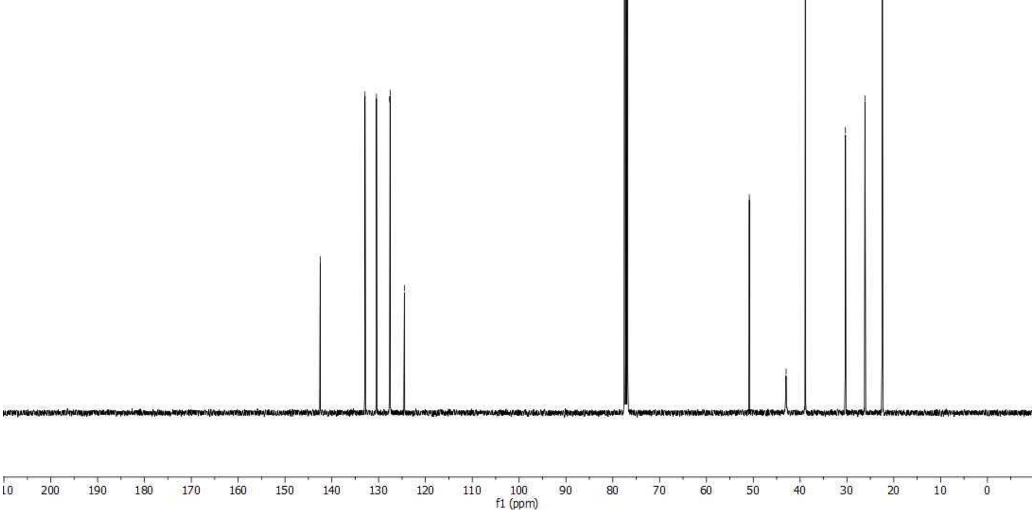

^{1}H NMR (400 MHz, $CDCl_3$)

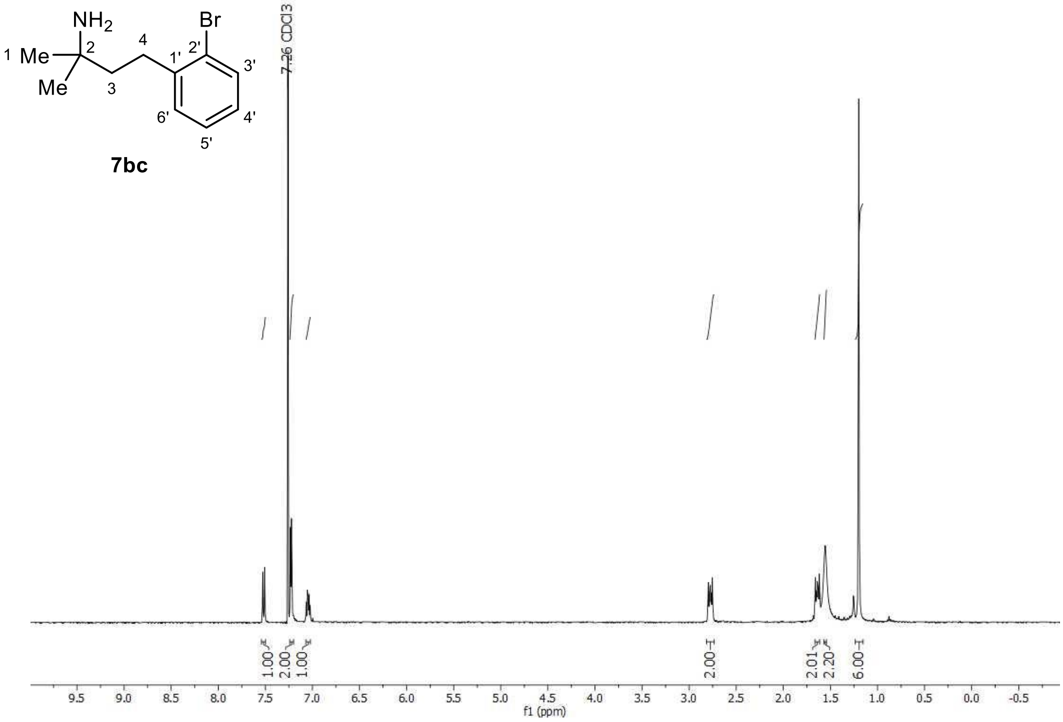

^{13}C{^{1}H} NMR (126 MHz, $CDCl_3$)

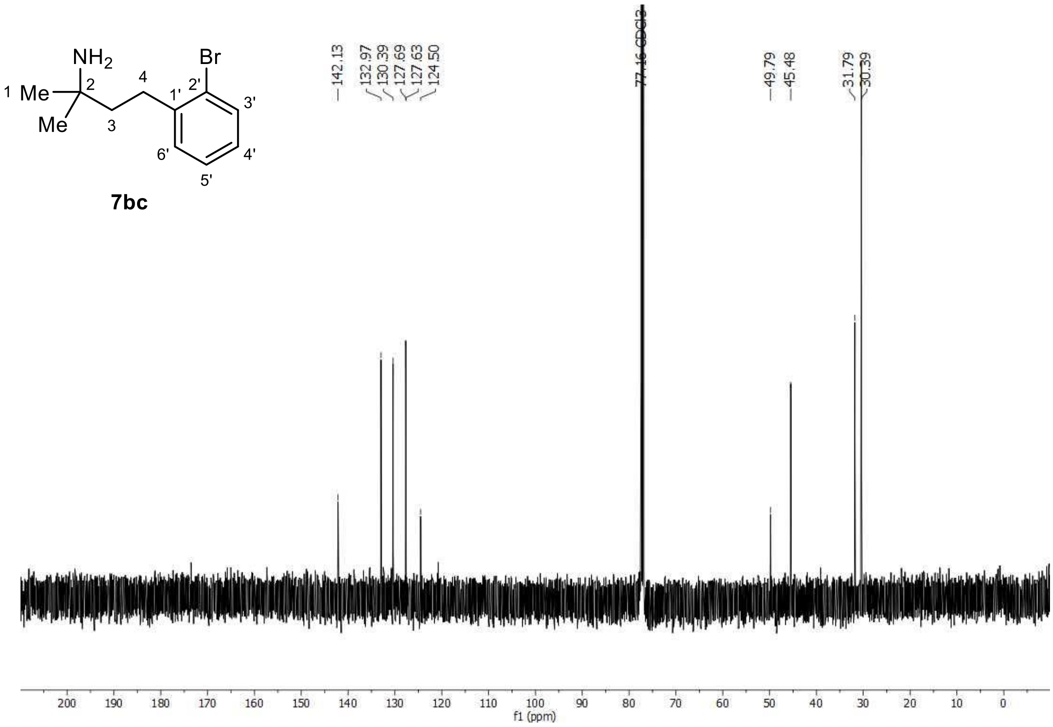

^{1}H NMR (400 MHz, $CDCl_3$)

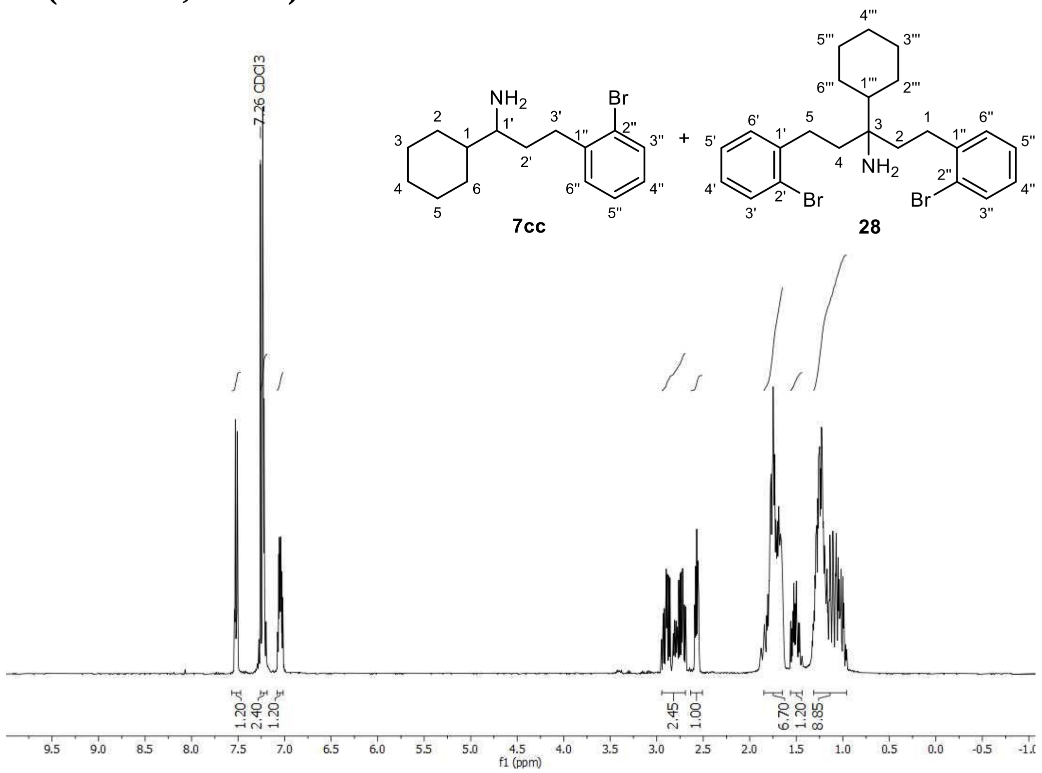

^{13}C{^{1}H} NMR (101 MHz, $CDCl_3$)

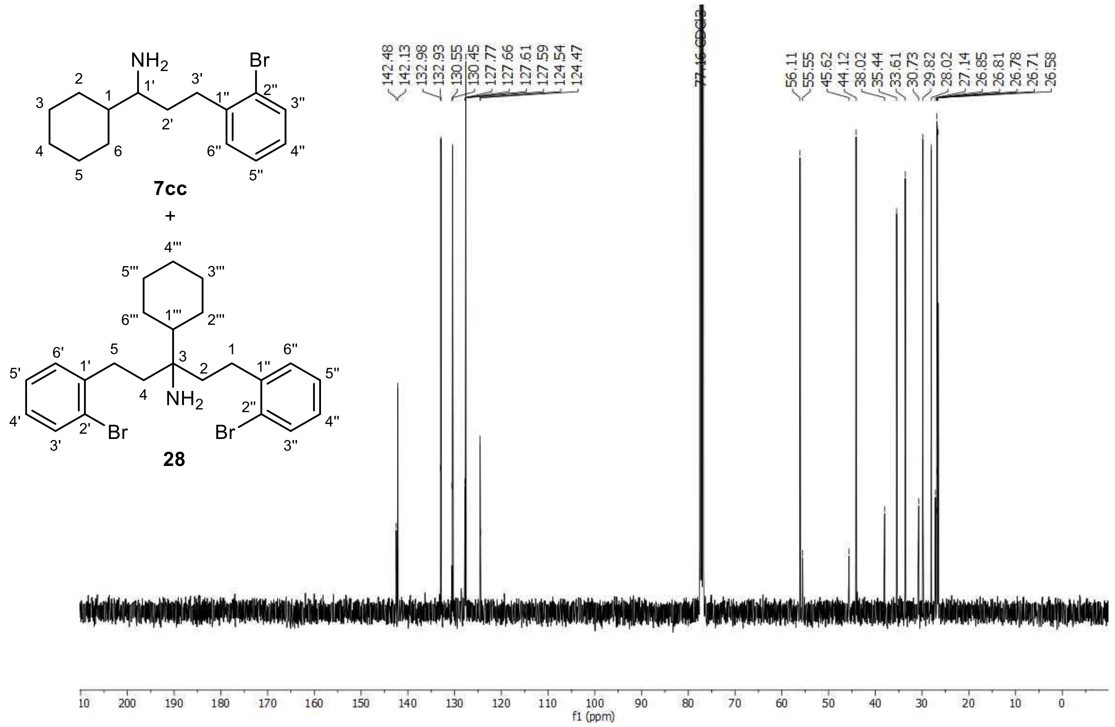

^{1}H NMR (400 MHz, $CDCl_3$)

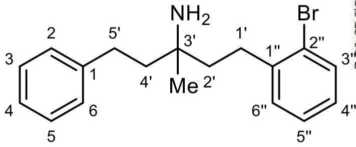

7ec

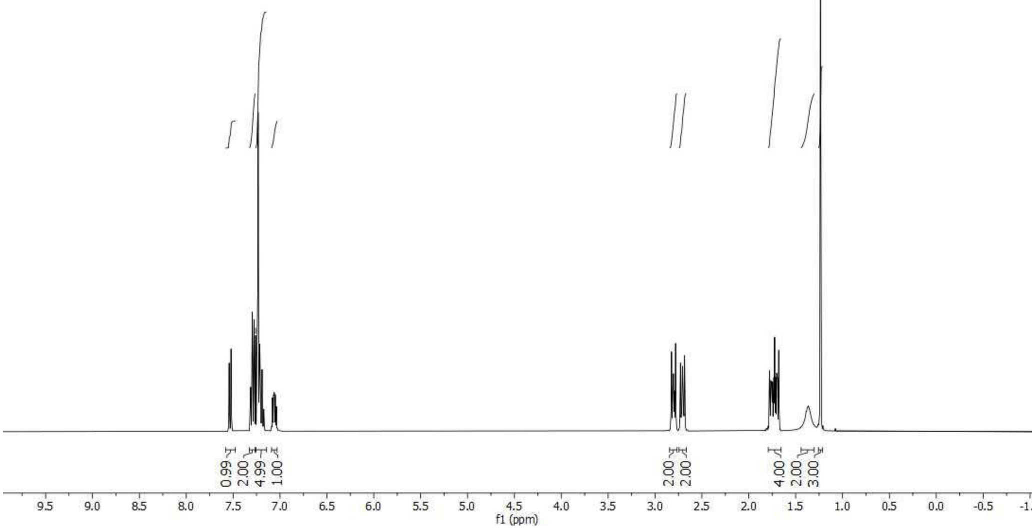

^{13}C{^{1}H} NMR (101 MHz, $CDCl_3$)

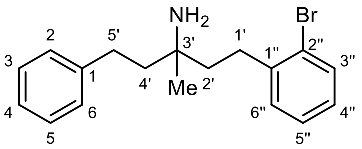

7ec

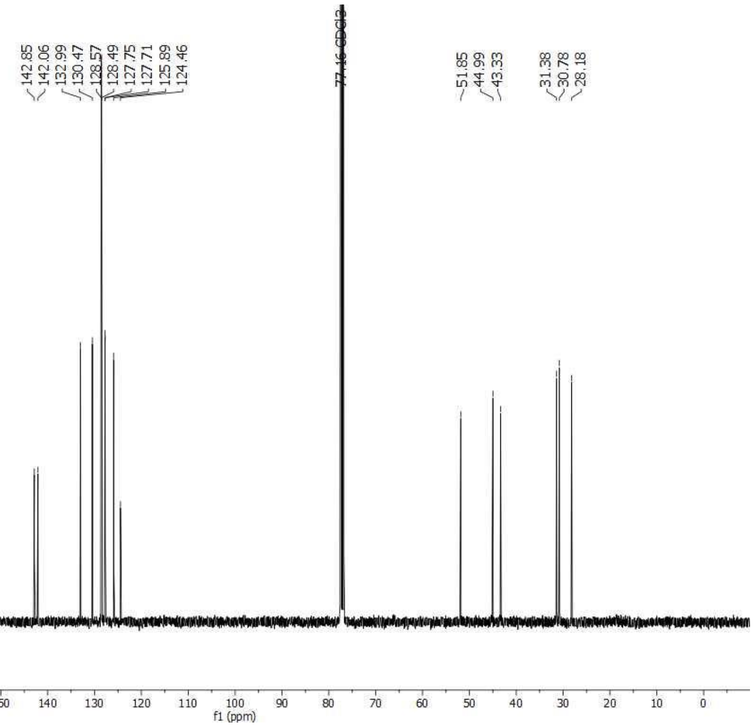

^{1}H NMR (400 MHz, CDCl3)

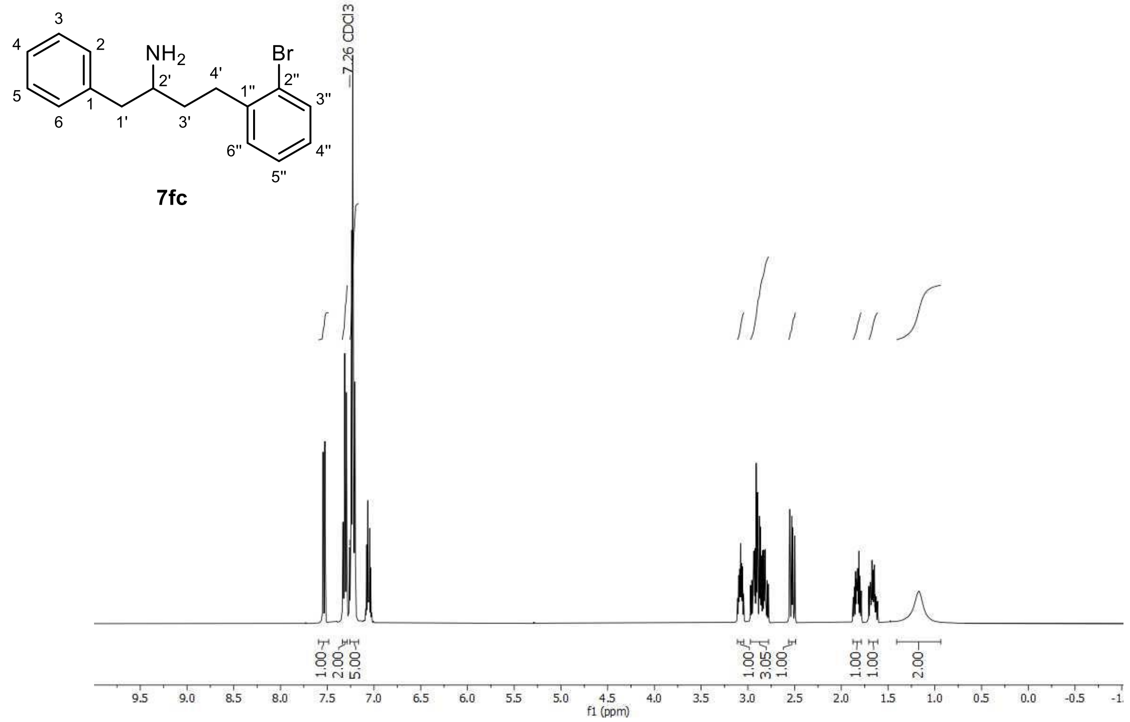

^{13}C{^{1}H} NMR (101 MHz, CDCl3)

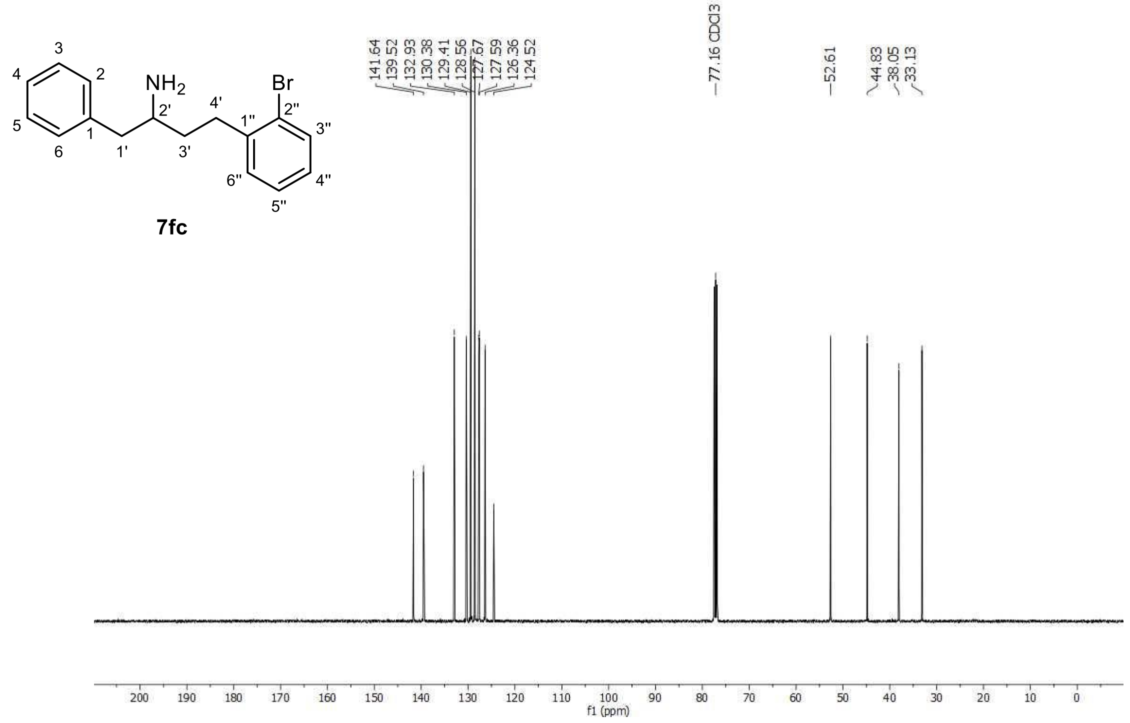

^{1}H NMR (400 MHz, CDCl3)

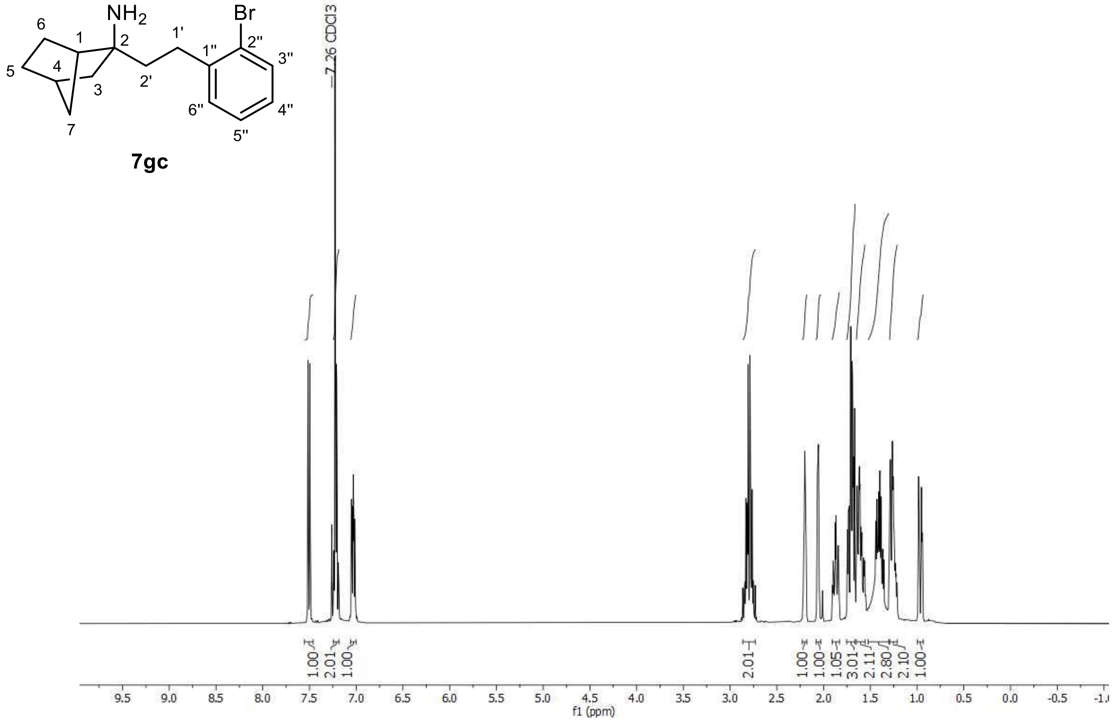

^{13}C{^{1}H} NMR (101 MHz, CDCl3)

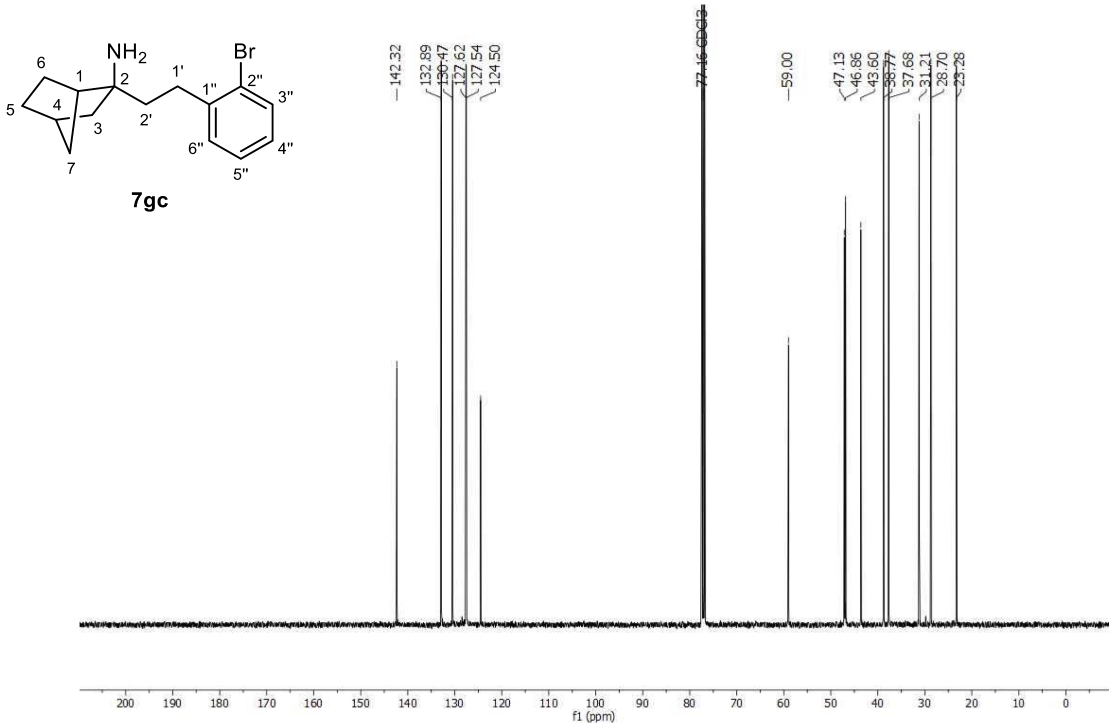

^{1}H NMR (400 MHz, CDCl$_3$)

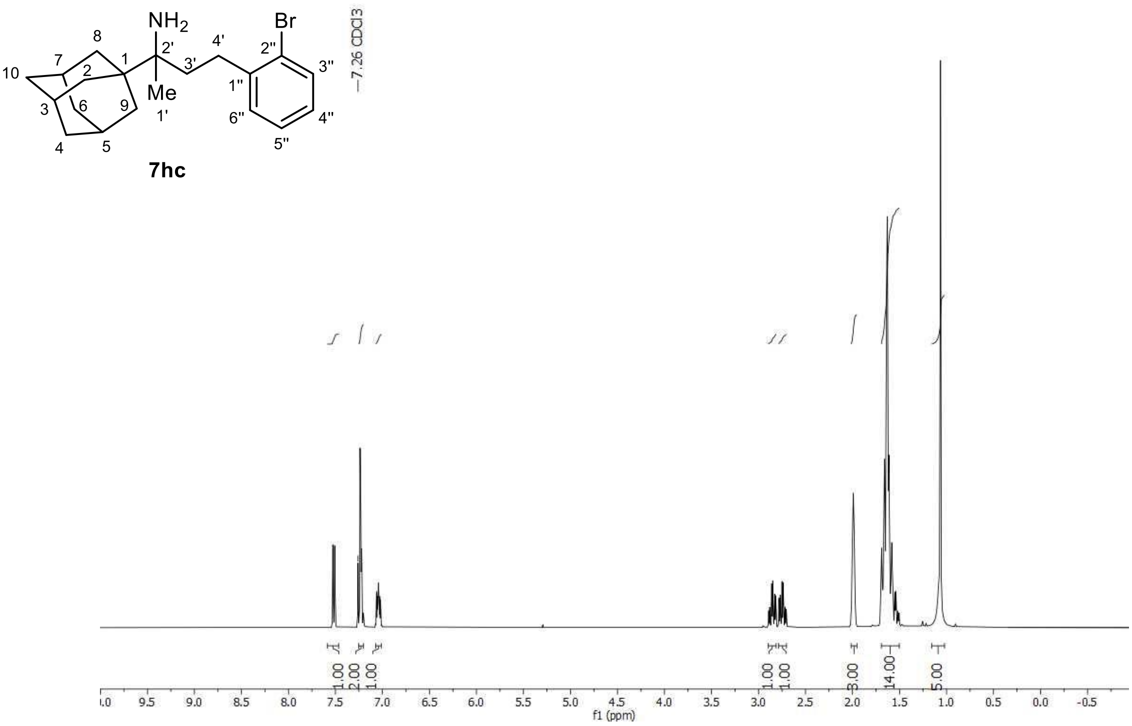

^{13}C{^{1}H} NMR (101 MHz, CDCl$_3$)

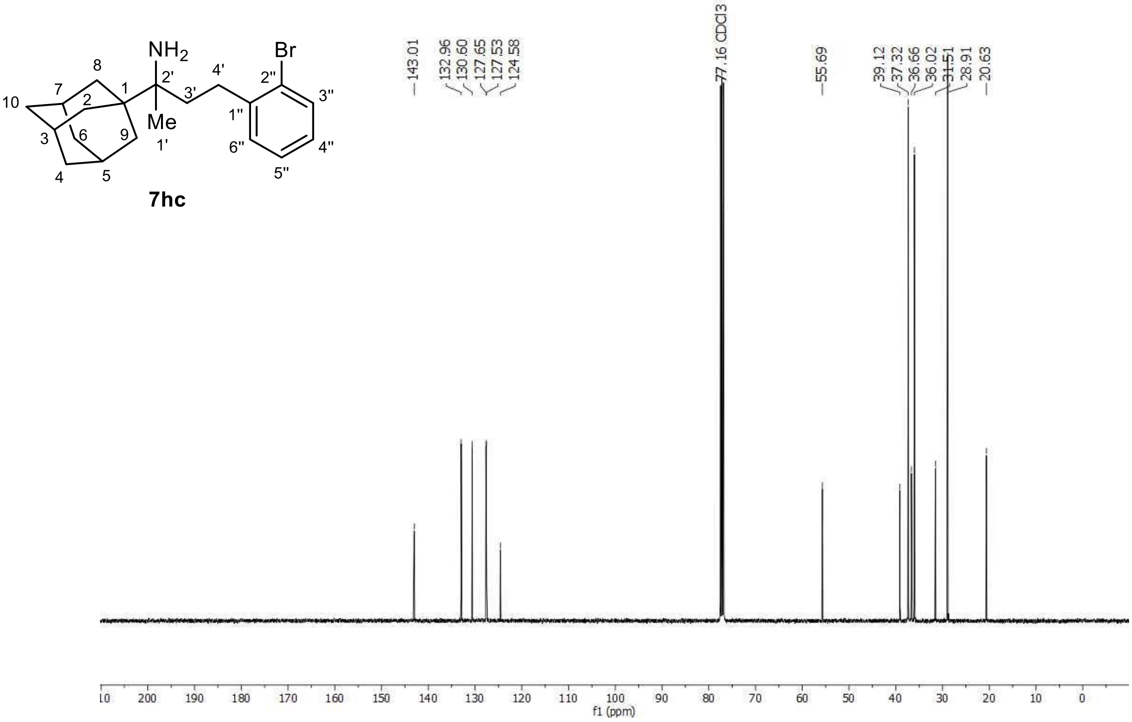

^{1}H NMR (400 MHz, $CDCl_3$)

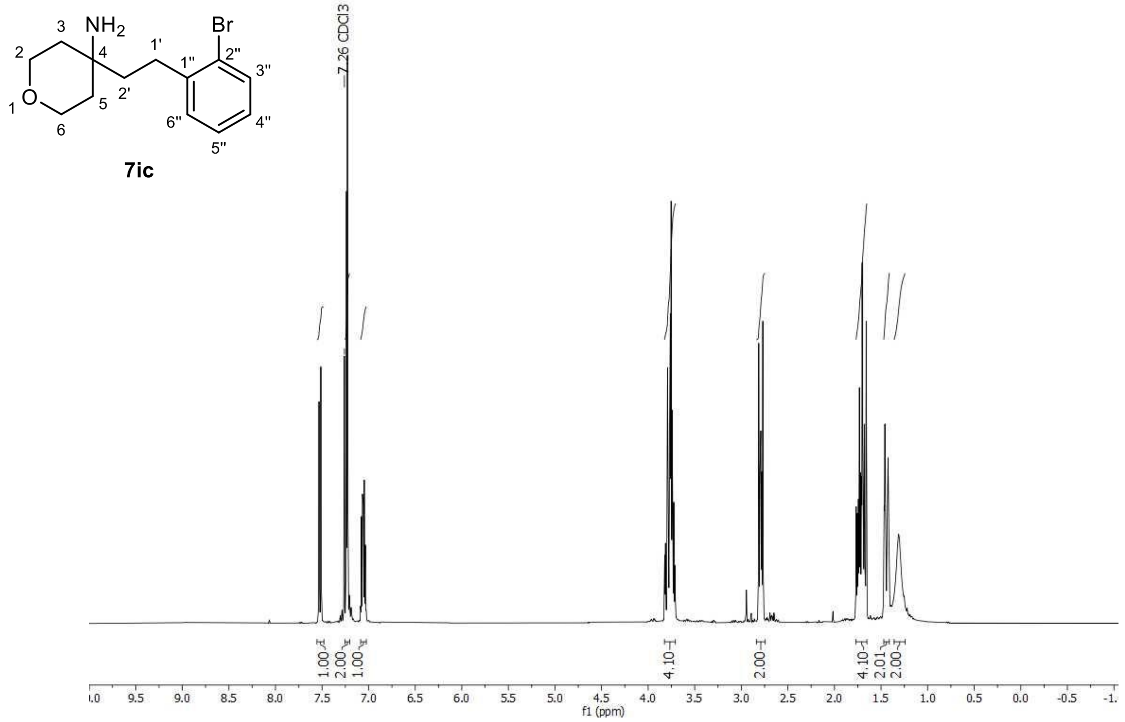

^{13}C{^{1}H} NMR (101 MHz, $CDCl_3$)

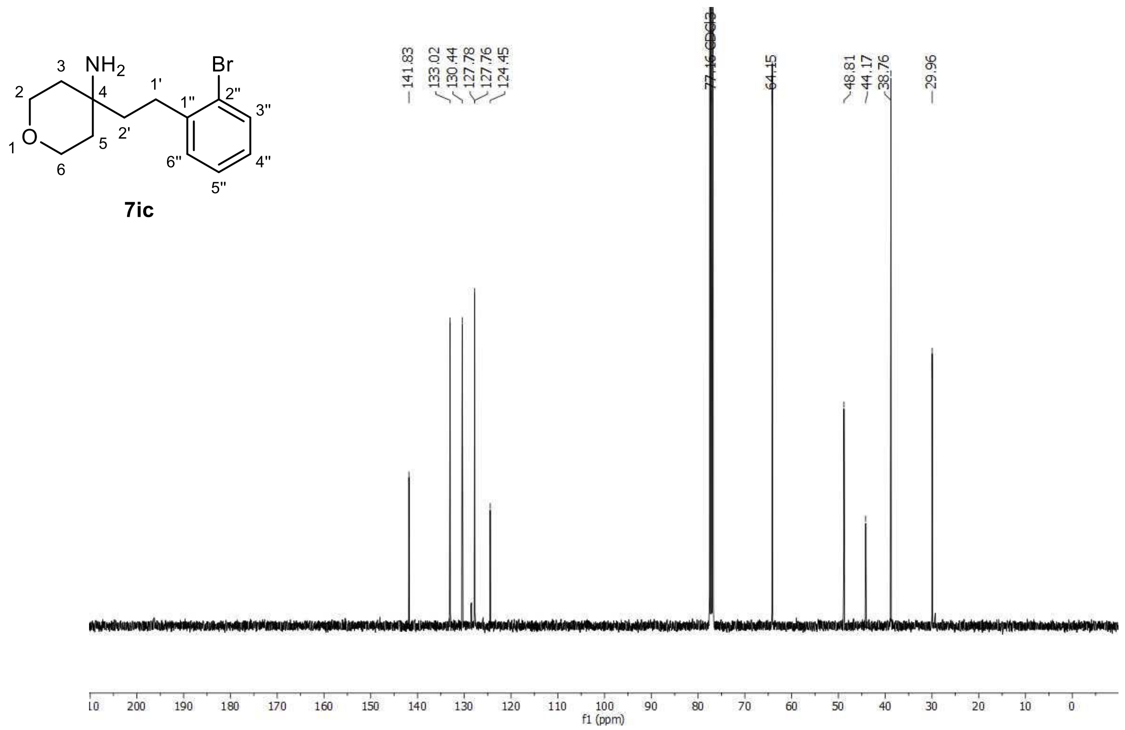

^{1}H NMR (400 MHz, CDCl3)

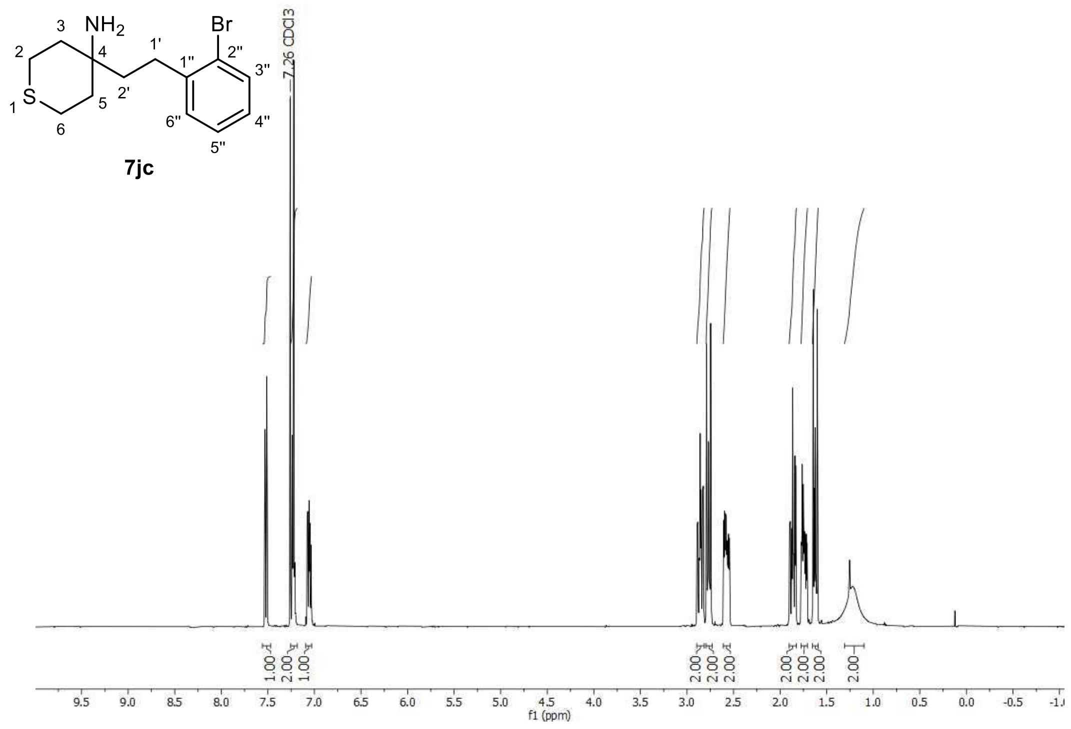

^{13}C{^{1}H} NMR (101 MHz, CDCl3)

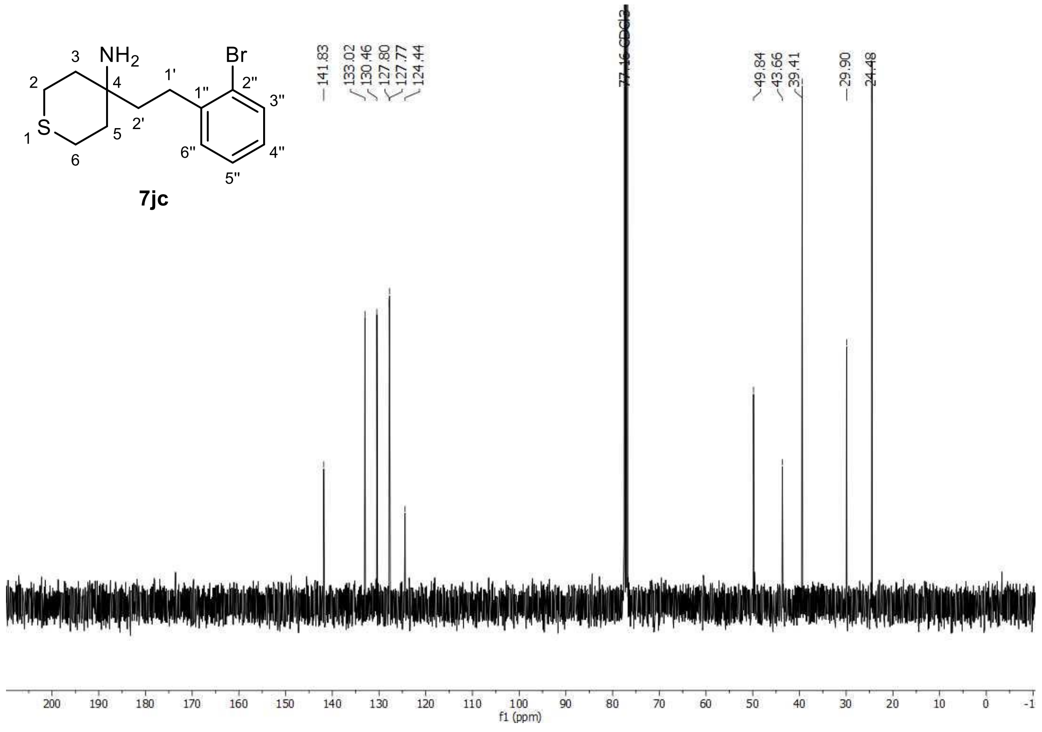

^{1}H NMR (400 MHz, $CDCl_3$)

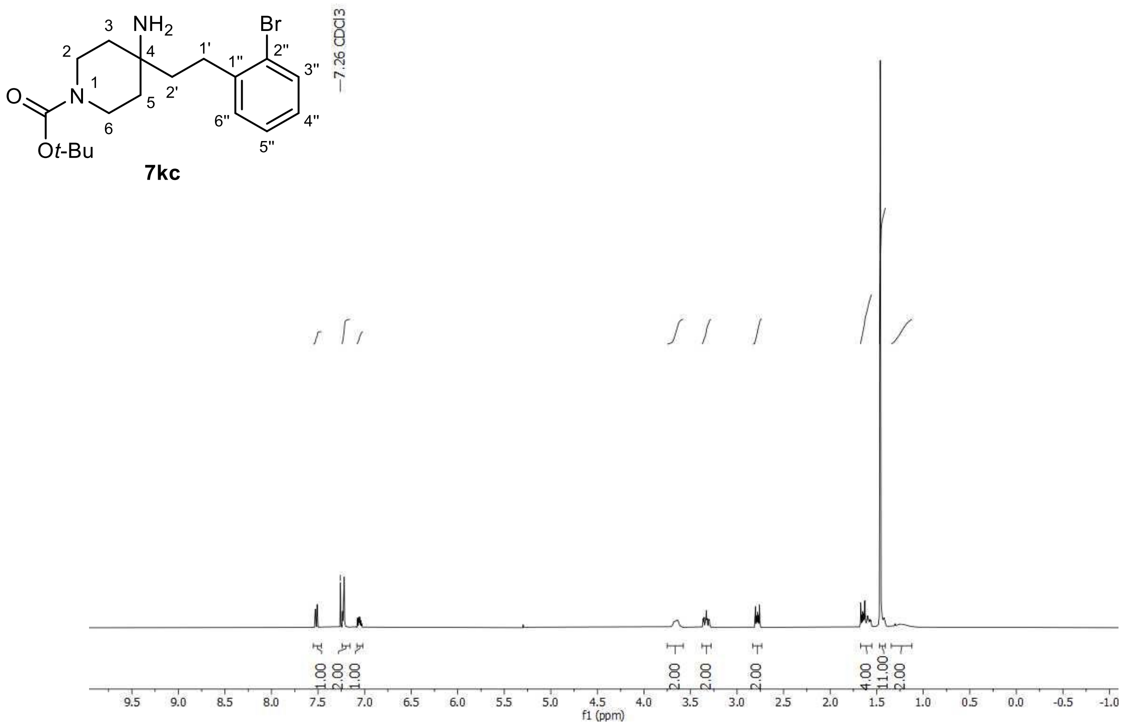

^{13}C{^{1}H} NMR (101 MHz, $CDCl_3$)

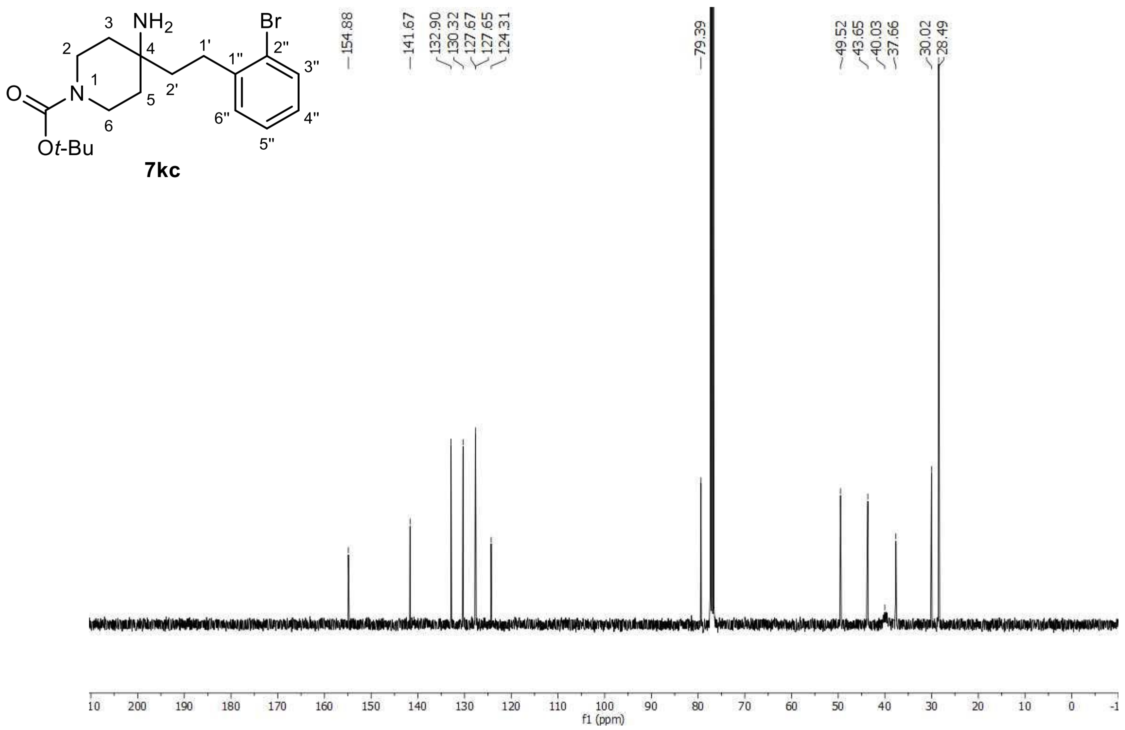

^{1}H NMR (400 MHz, $CDCl_3$)

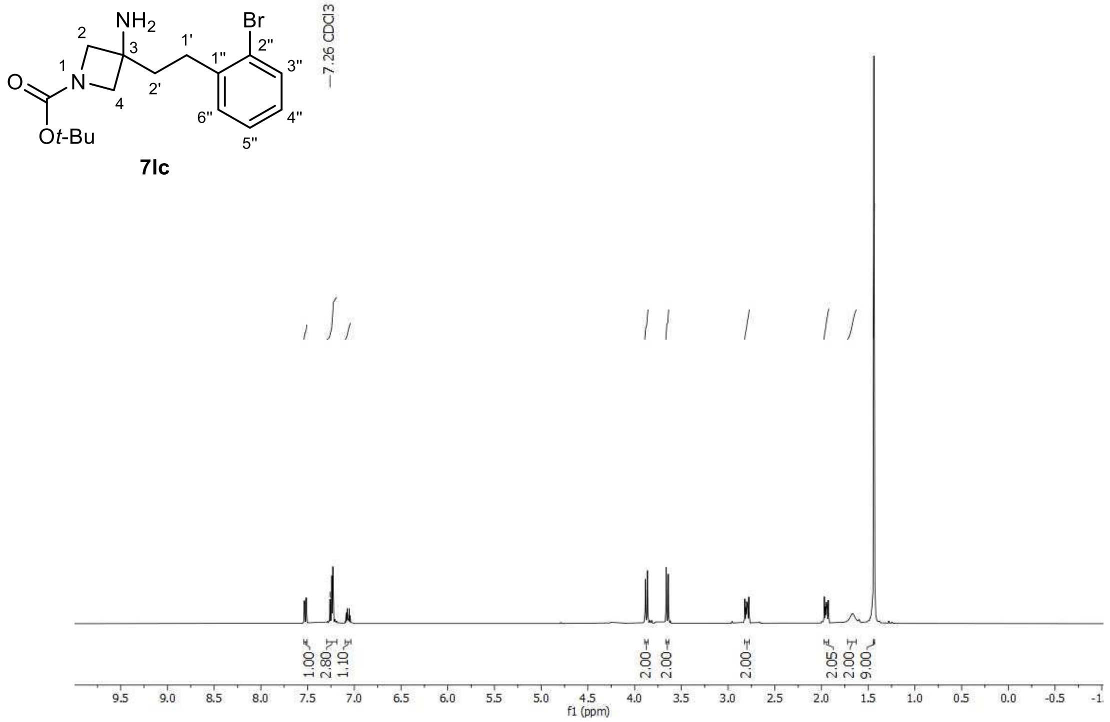

^{13}C{^{1}H} NMR (101 MHz, $CDCl_3$)

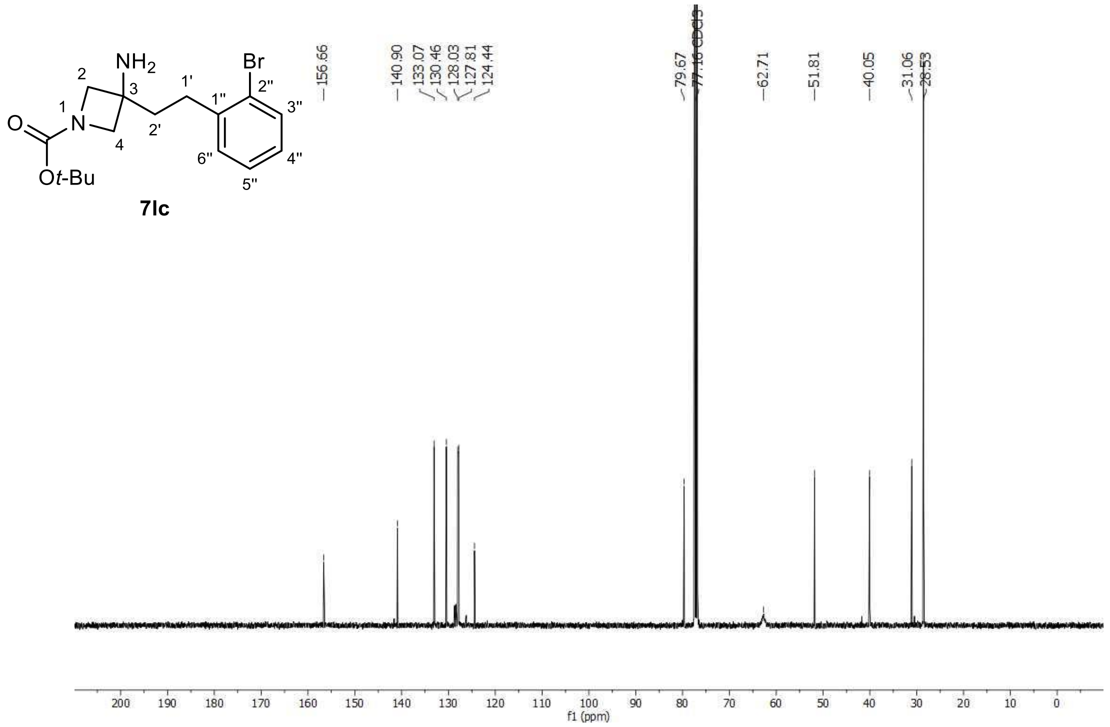

^{1}H NMR (400 MHz, $CDCl_3$)

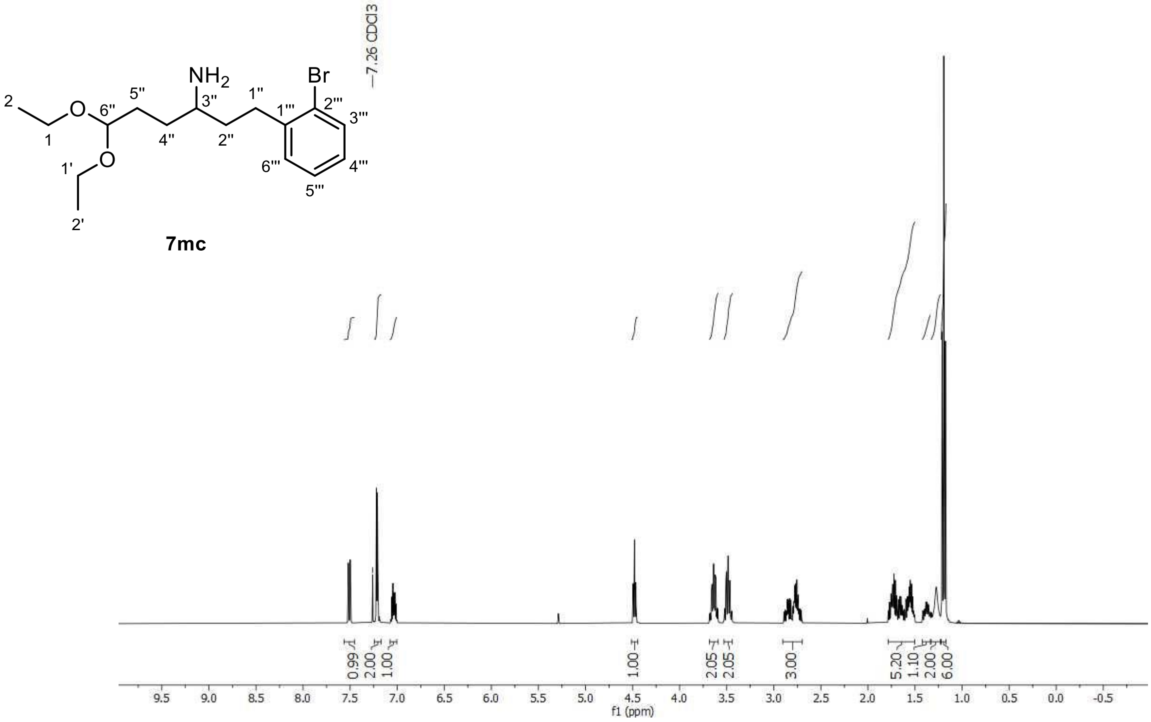

^{13}C{^{1}H} NMR (101 MHz, $CDCl_3$)

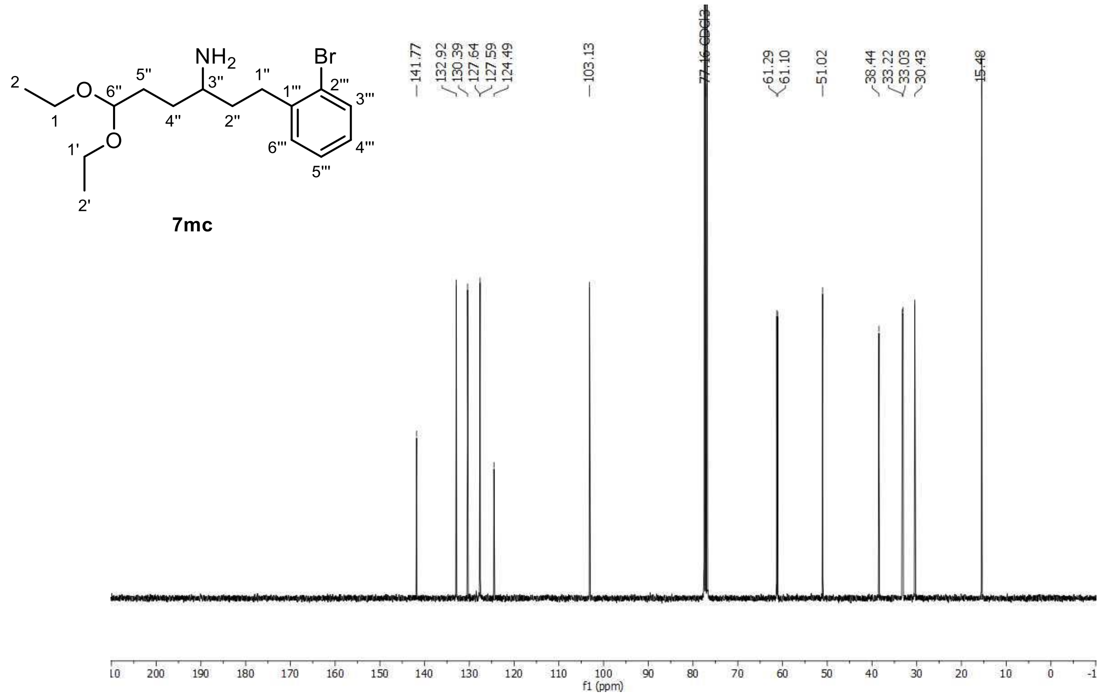

^{1}H NMR (400 MHz, $CDCl_3$)

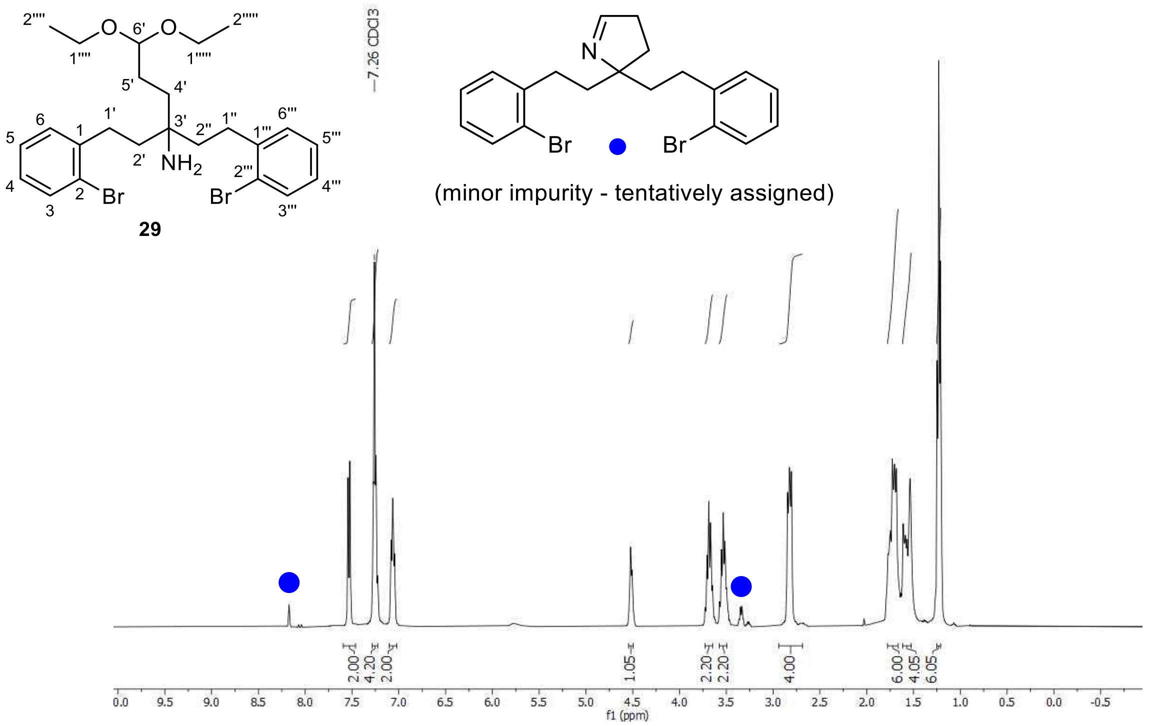

^{13}C{^{1}H} NMR (101 MHz, $CDCl_3$)

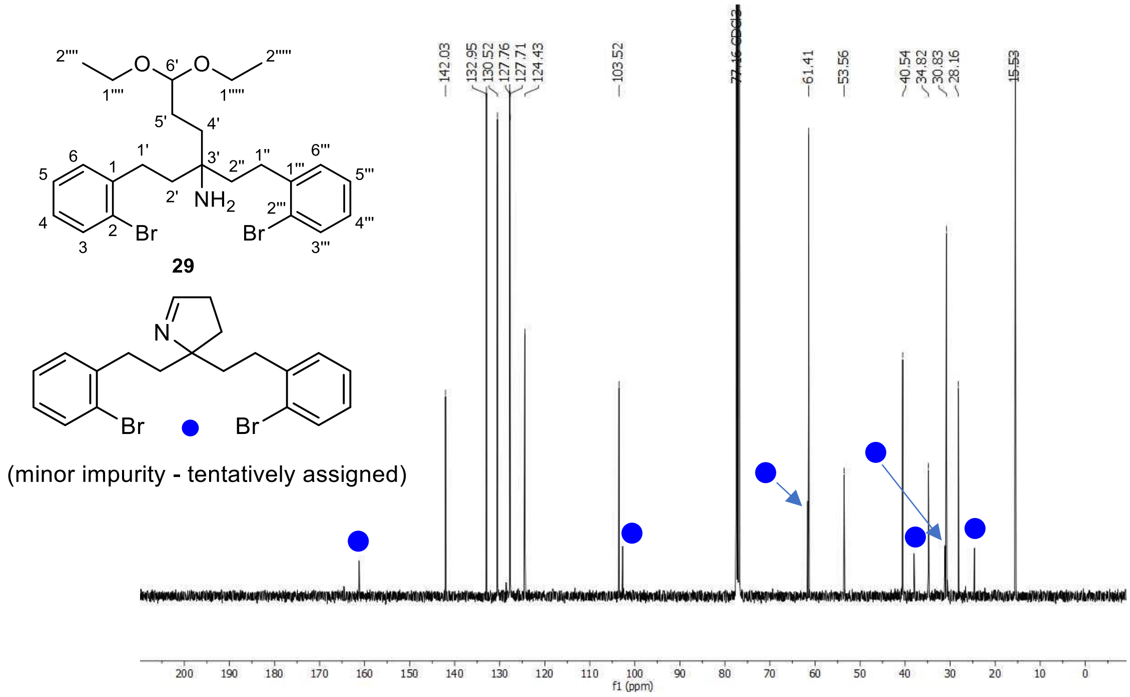

^{1}H NMR (400 MHz, $CDCl_3$)

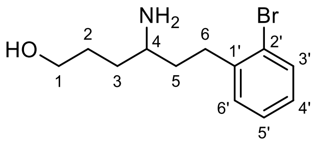

7nc

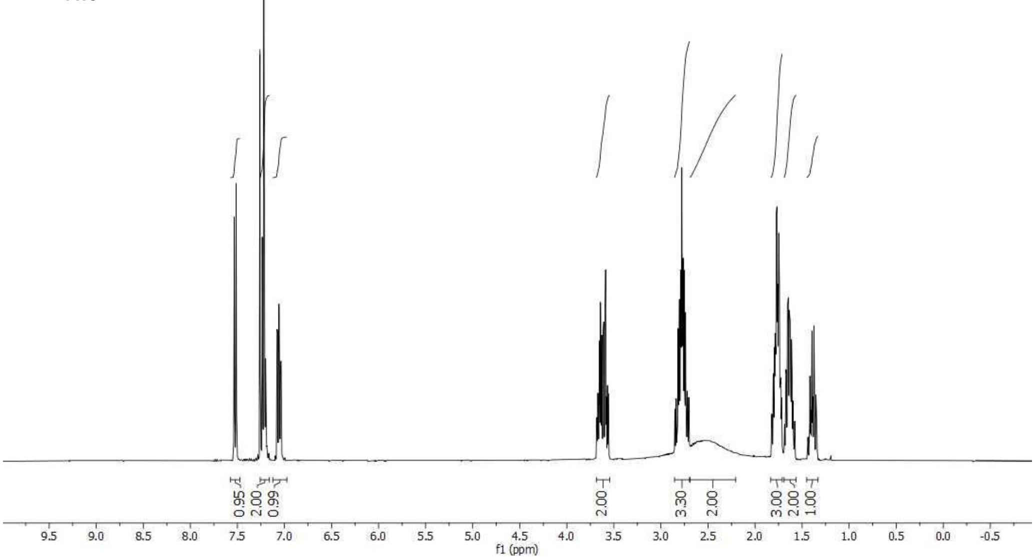

$^{13}C\{^{1}H\}$ NMR (101 MHz, $CDCl_3$)

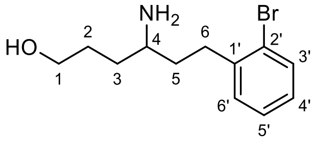

7nc

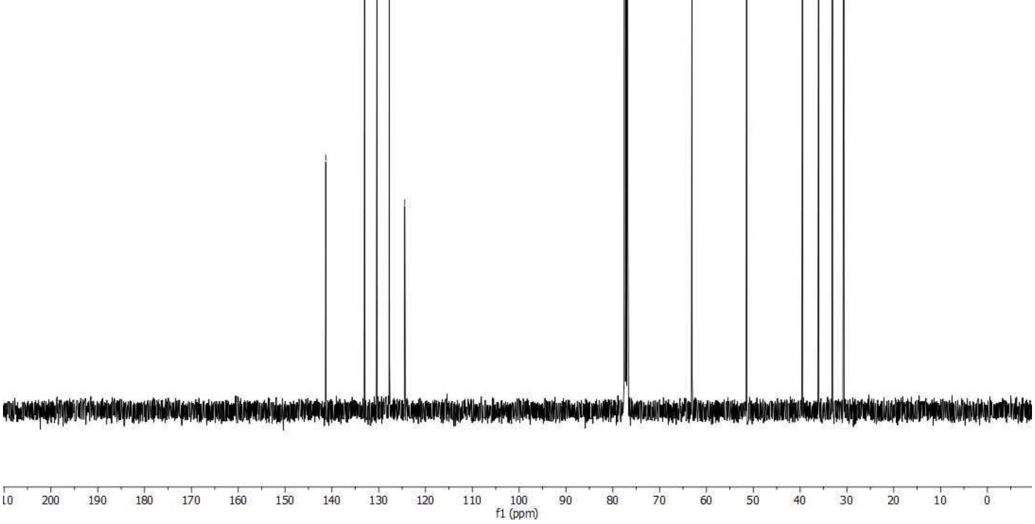

¹H NMR (400 MHz, $CDCl_3$)

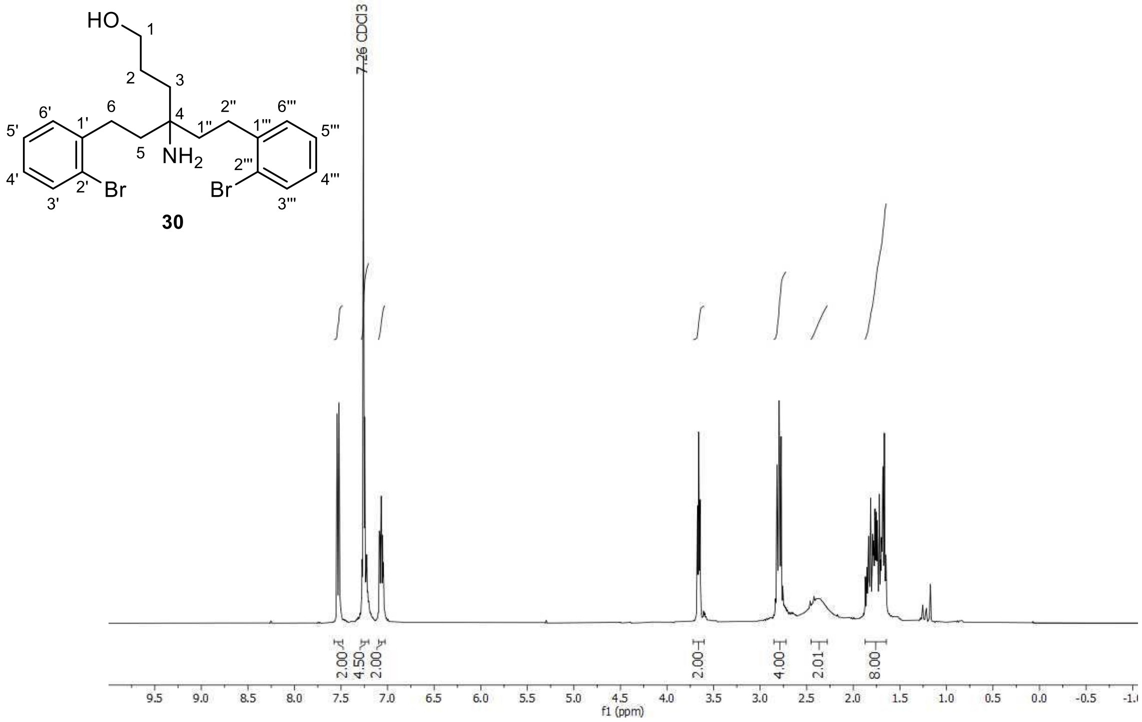

$^{13}C\{^1H\}$ NMR (101 MHz, $CDCl_3$)

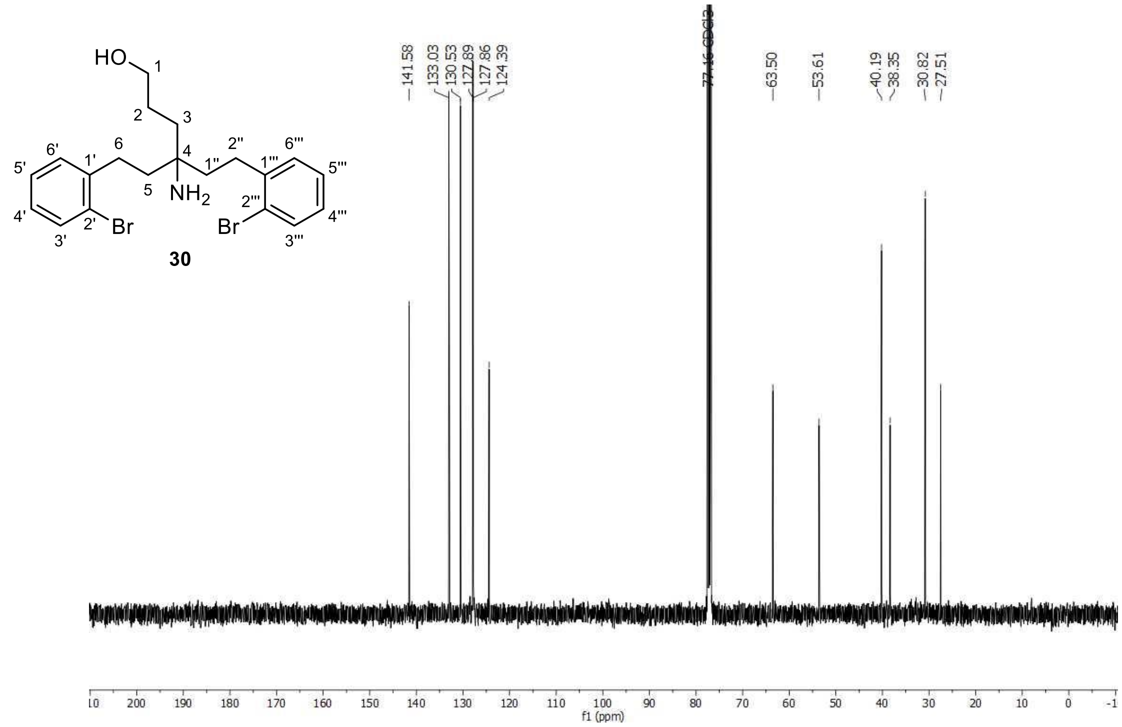

^{1}H NMR (400 MHz, CDCl$_3$)

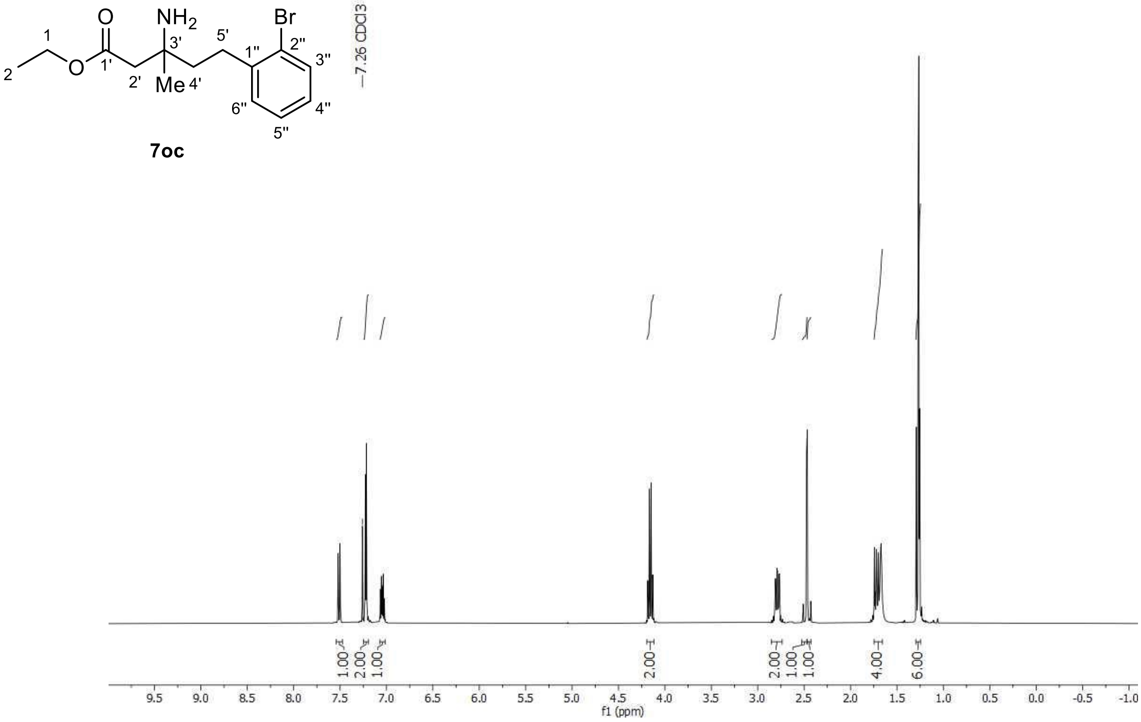

7oc

^{13}C{^{1}H} NMR (101 MHz, CDCl$_3$)

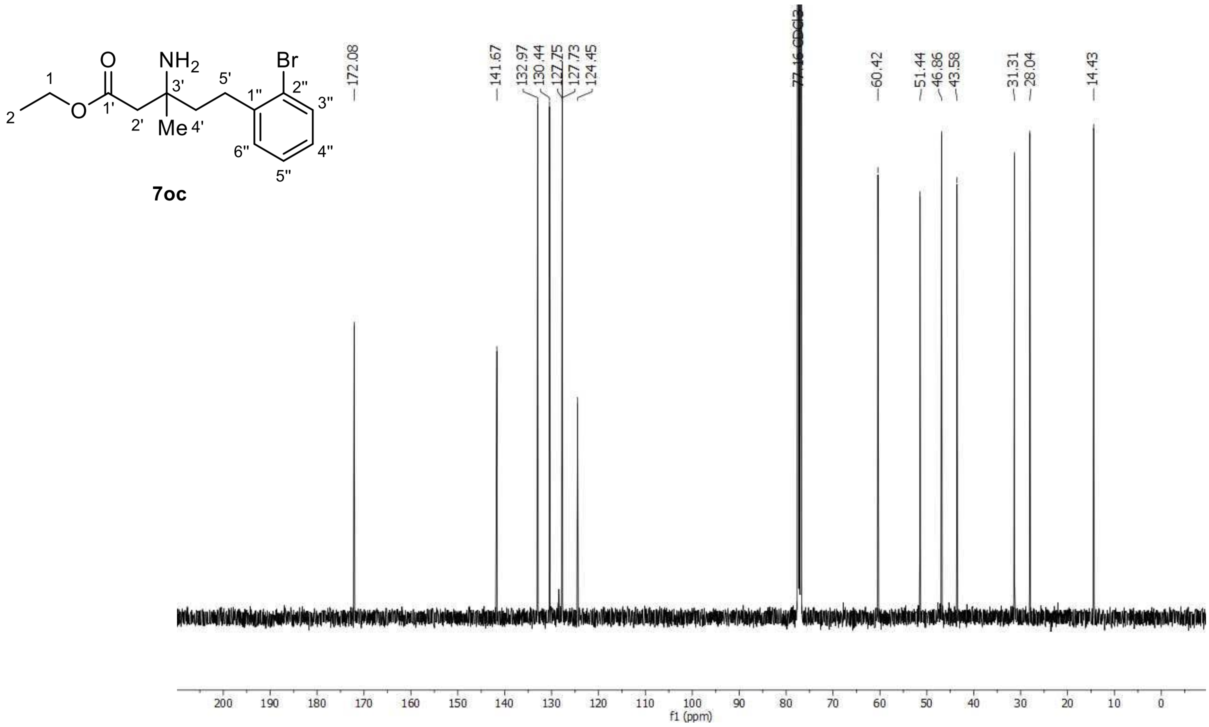

7oc

^{1}H NMR (400 MHz, $CDCl_3$)

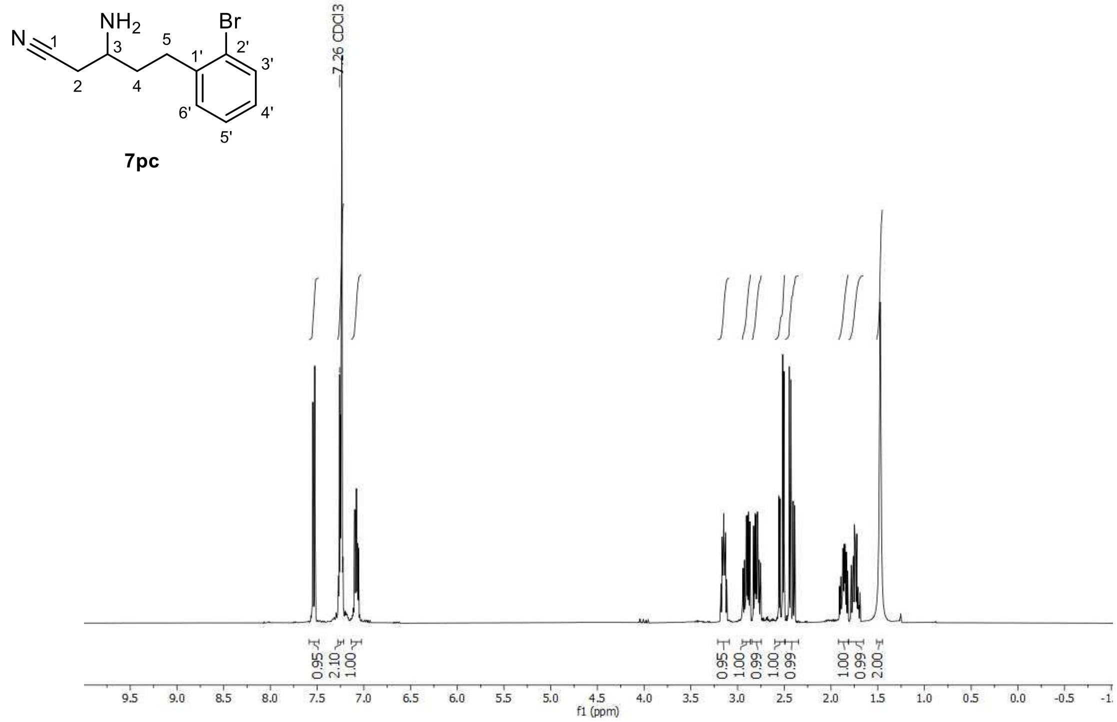

$^{13}C\{^{1}H\}$ NMR (101 MHz, $CDCl_3$)

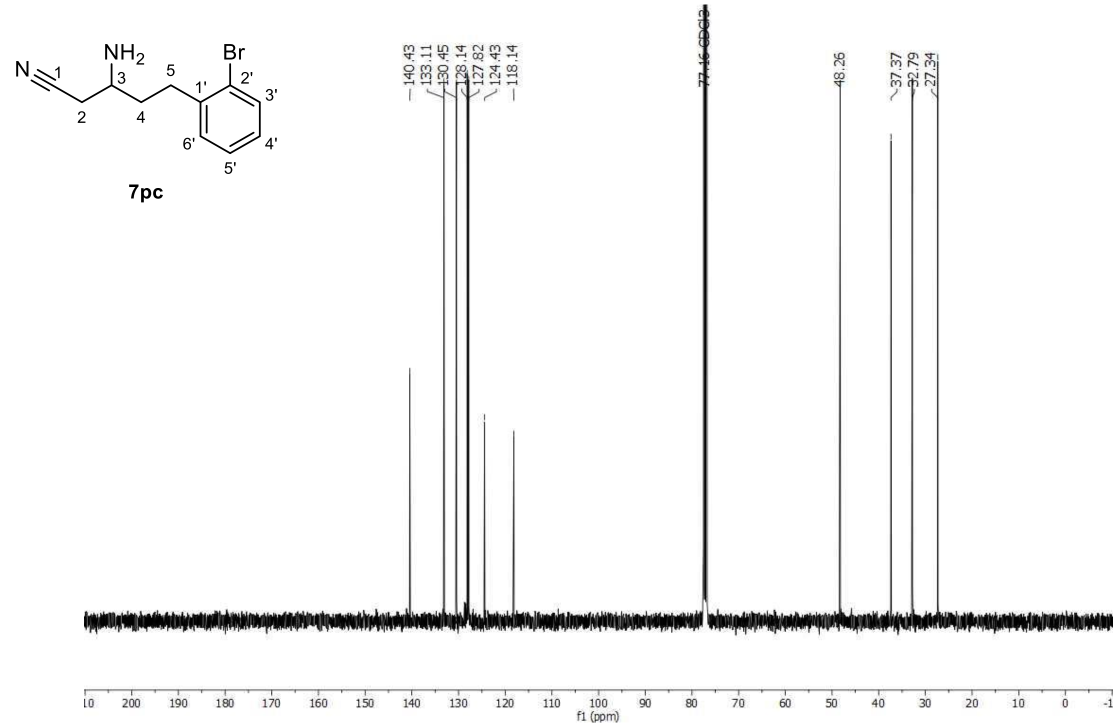

^{1}H NMR (400 MHz, CDCl$_3$)

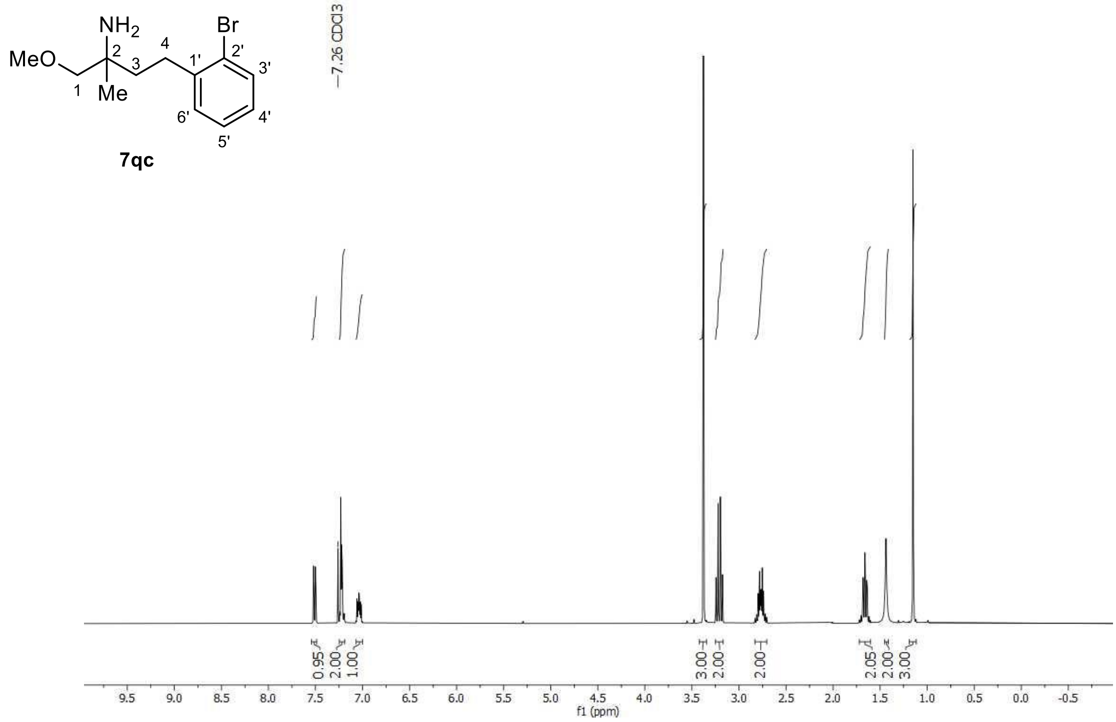

^{13}C{^{1}H} NMR (101 MHz, CDCl$_3$)

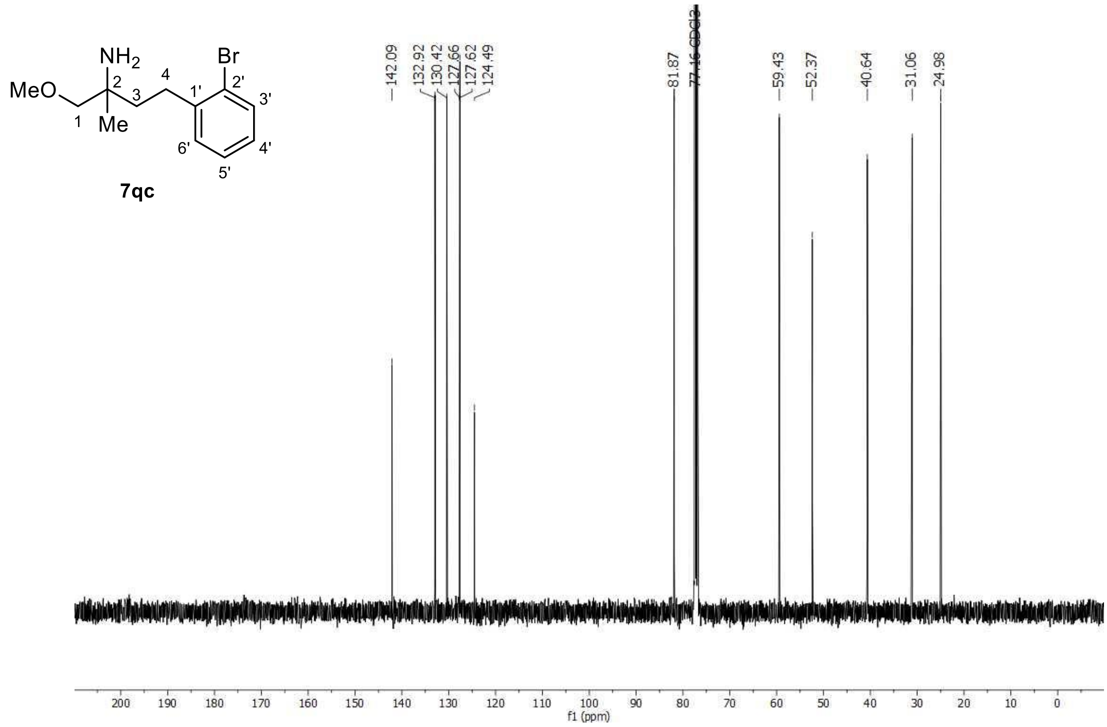

^{1}H NMR (400 MHz, CDCl3)

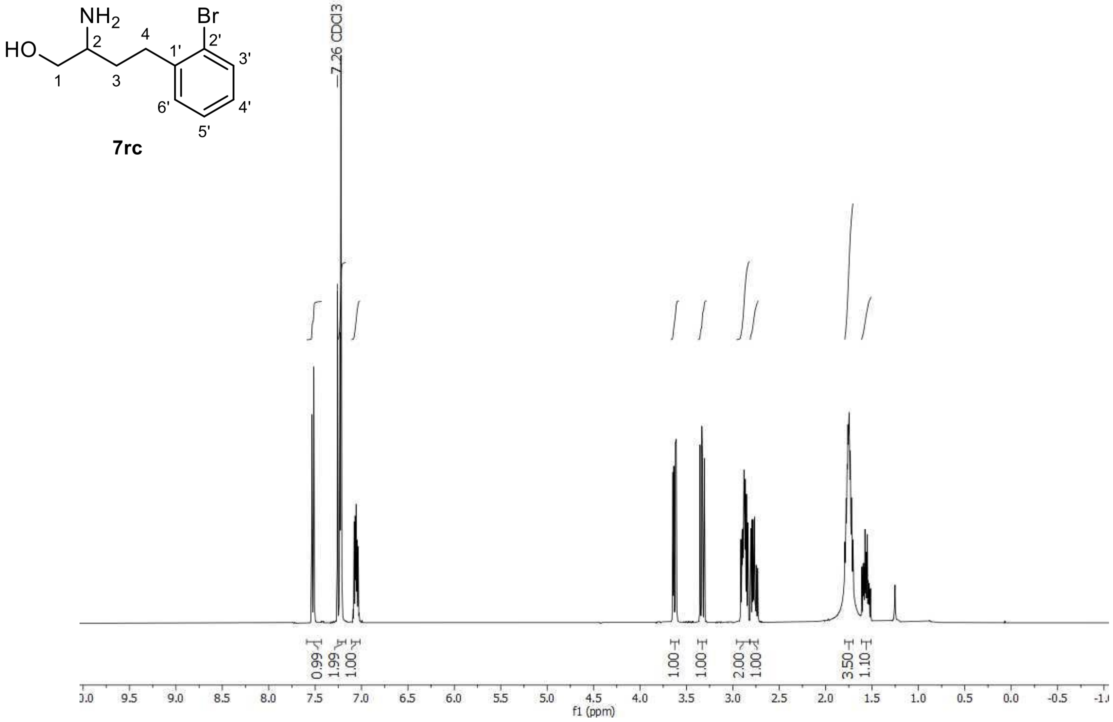

^{13}C{^{1}H} NMR (101 MHz, CDCl3)

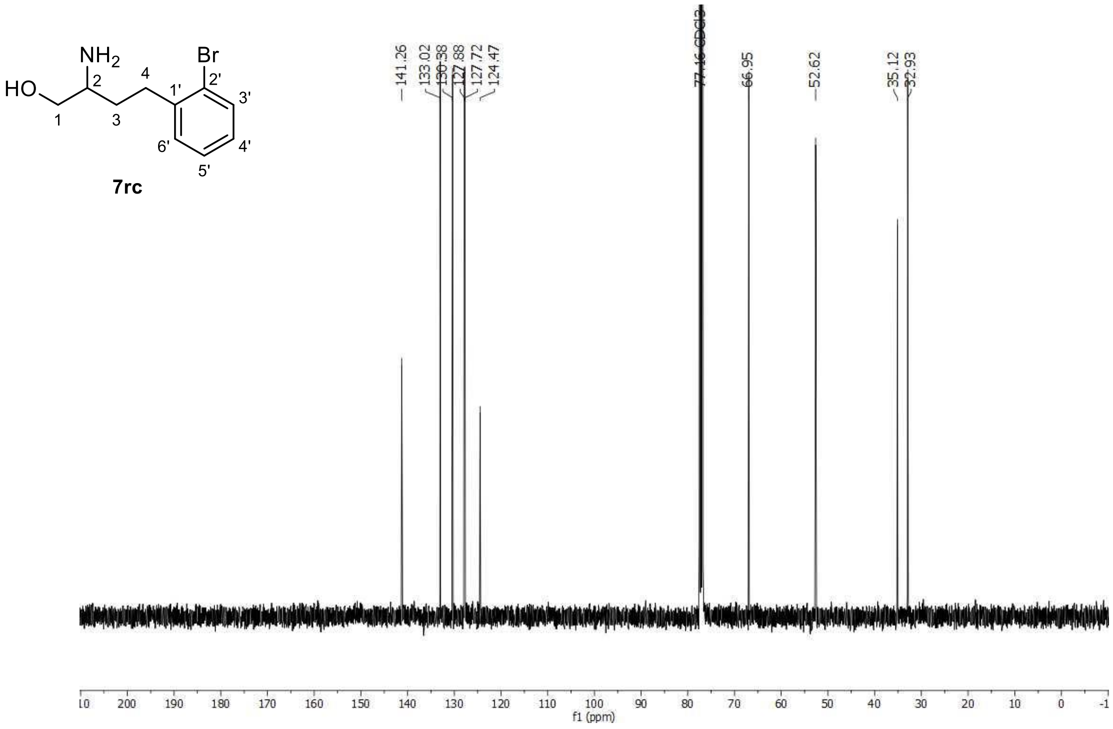

^{1}H NMR (400 MHz, CDCl$_3$)

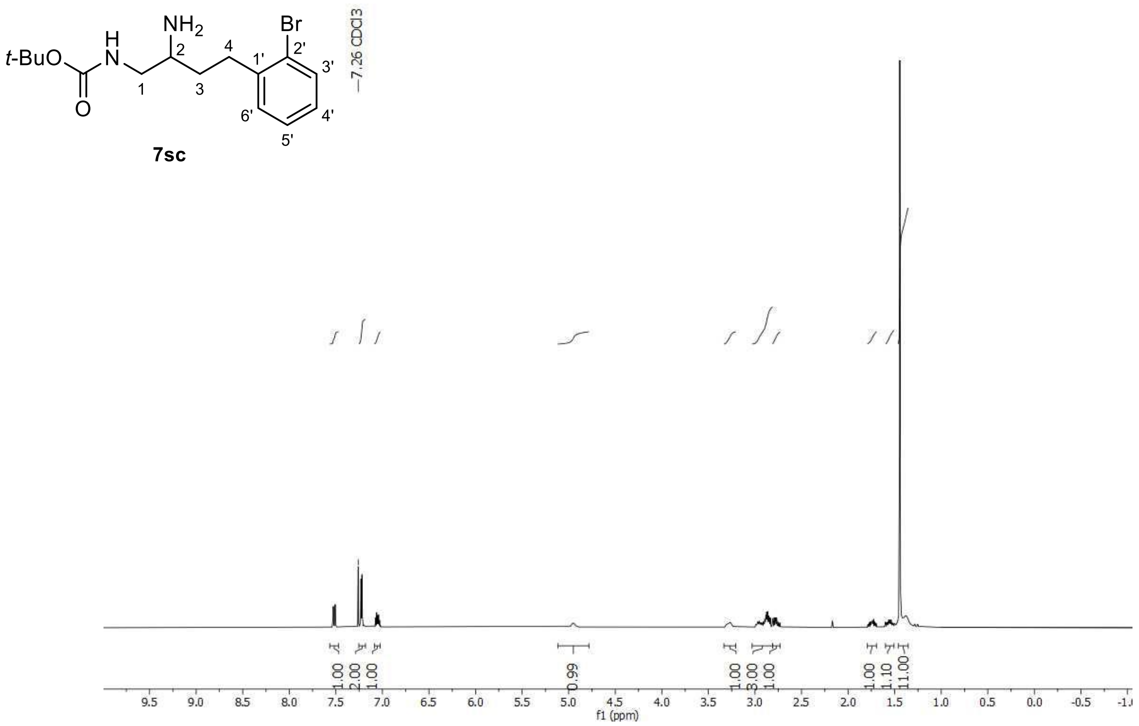

^{13}C{^{1}H} NMR (101 MHz, CDCl$_3$)

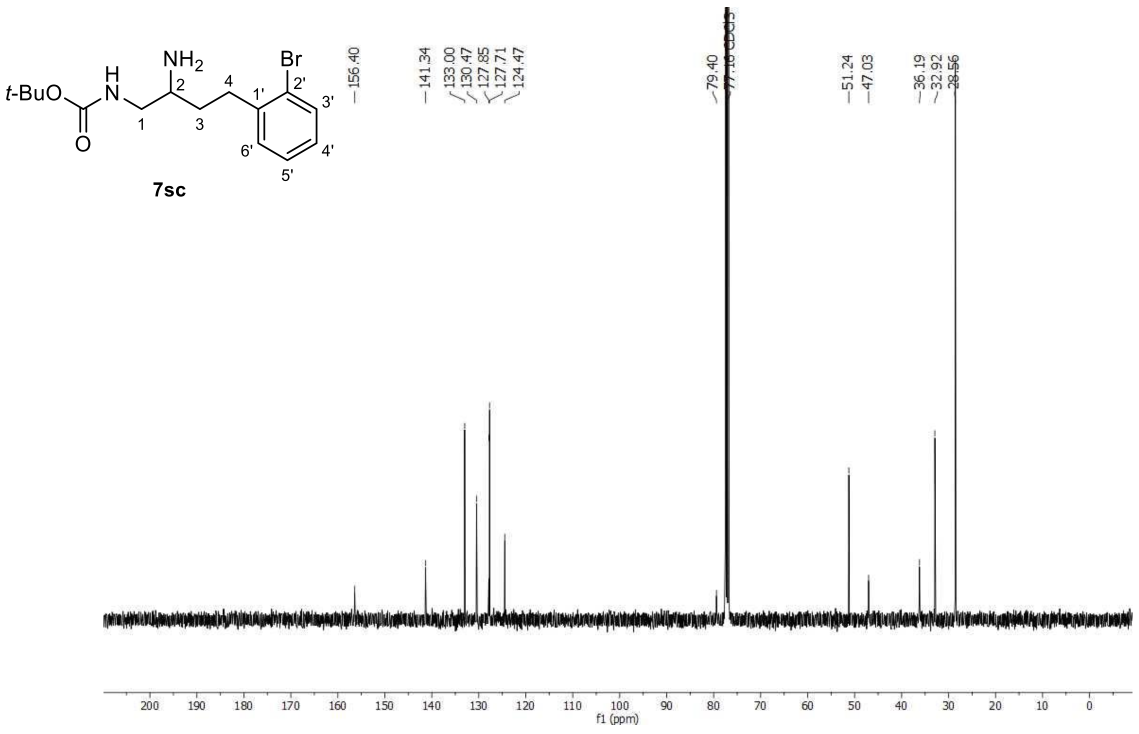

^{1}H NMR (400 MHz, $CDCl_3$)

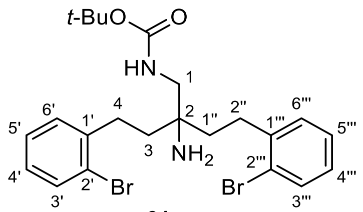

31

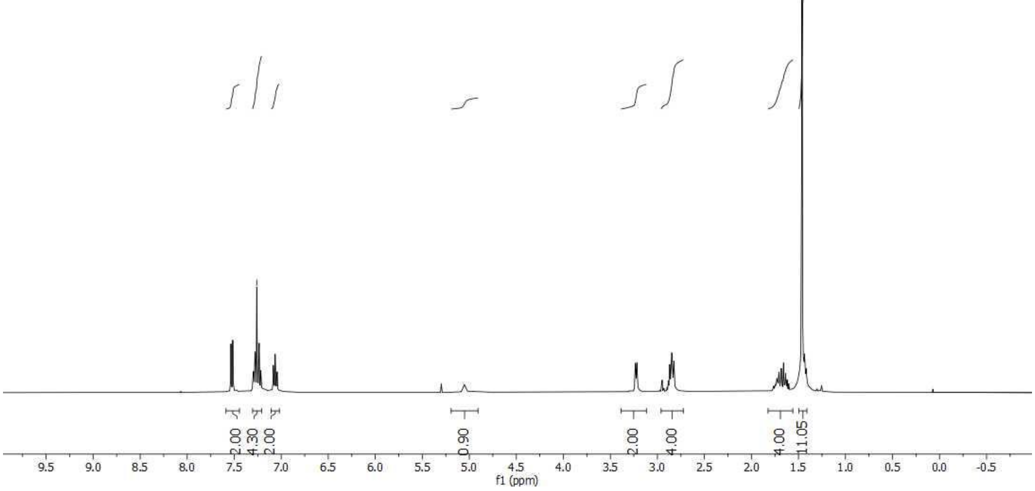

^{13}C{^{1}H} NMR (101 MHz, $CDCl_3$)

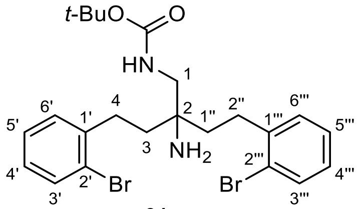

31

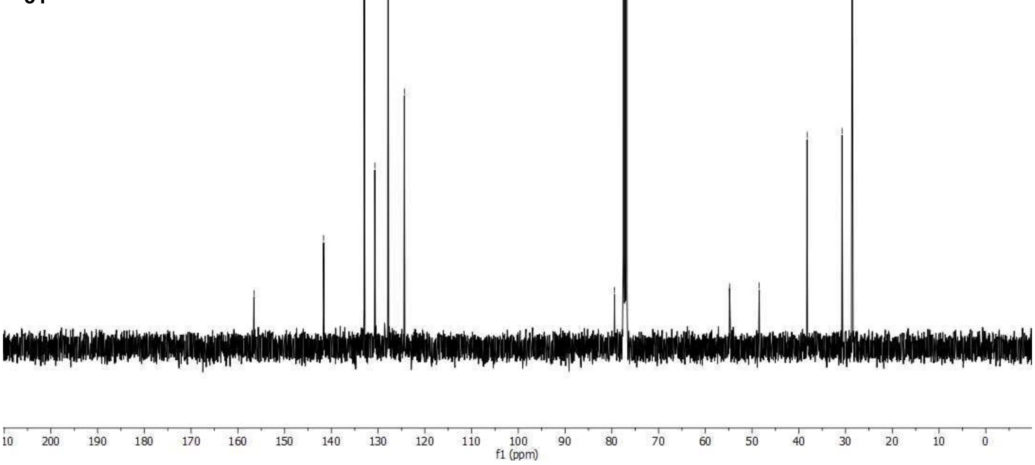

^{1}H NMR (400 MHz, CDCl3)

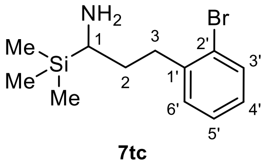

7tc

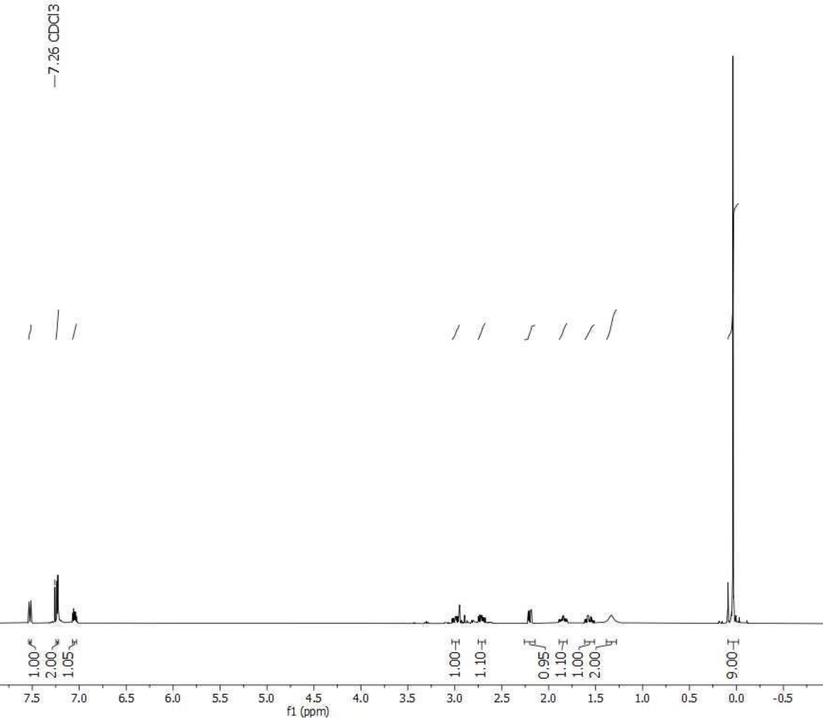

^{13}C{^{1}H} NMR (101 MHz, CDCl3)

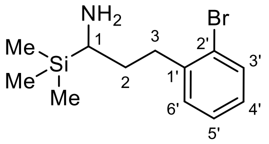

7tc

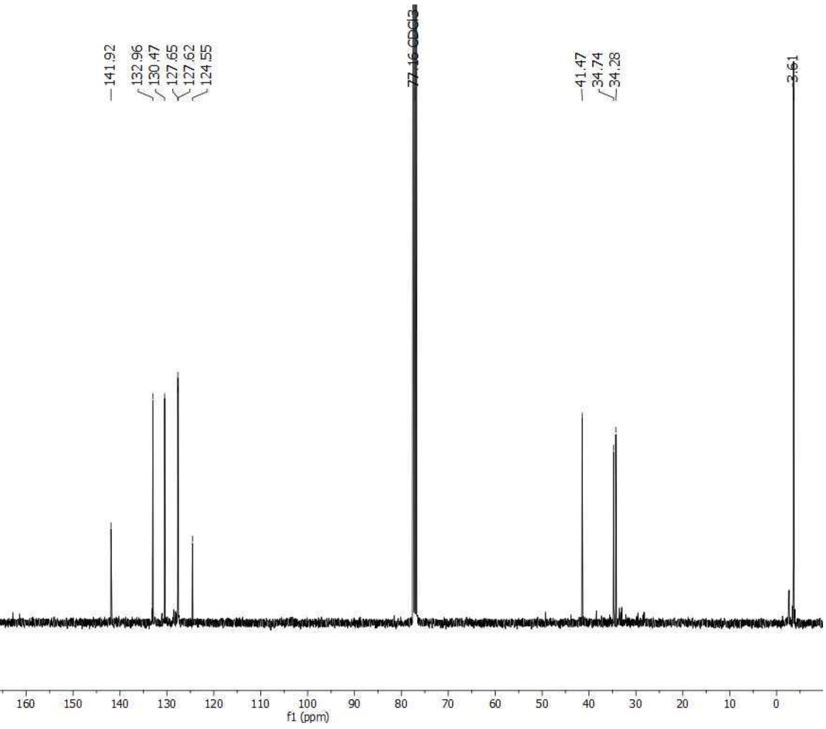

^{1}H NMR (400 MHz, CDCl$_3$)

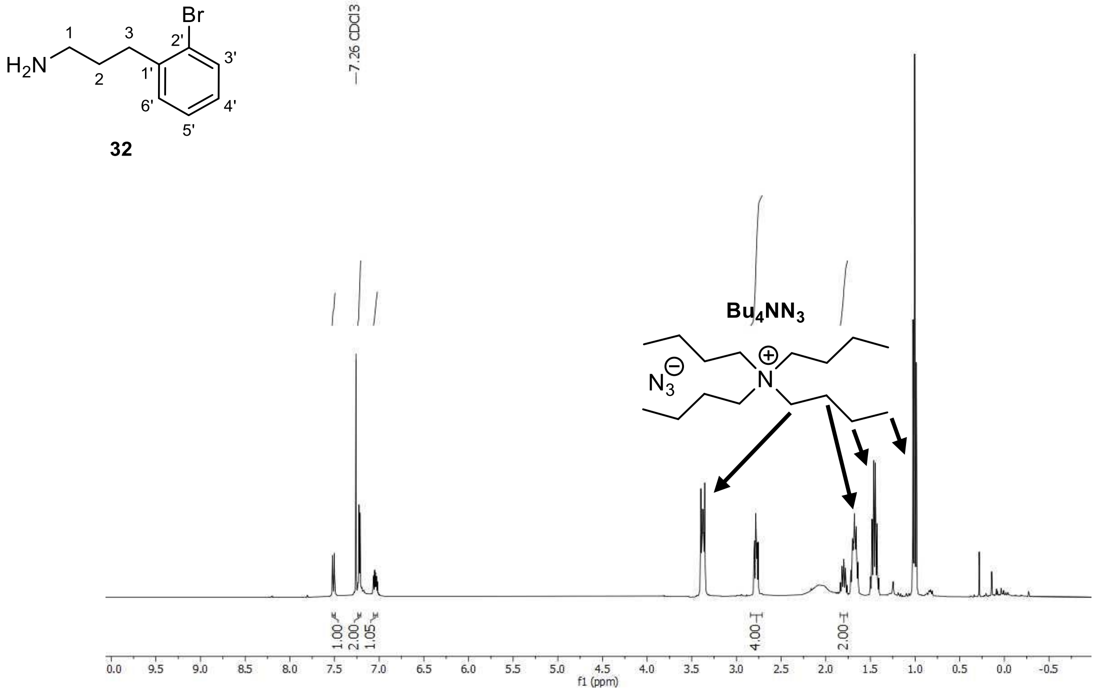

^{13}C{^{1}H} NMR (101 MHz, CDCl$_3$)

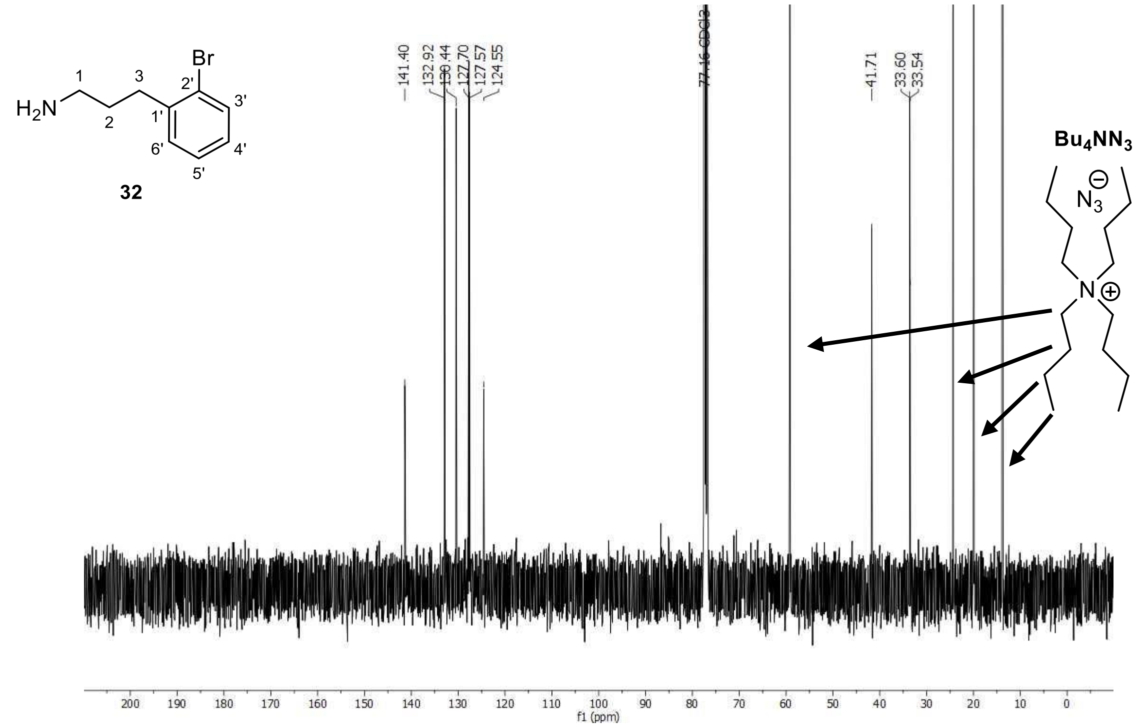

¹H NMR (400 MHz, CDCl₃)

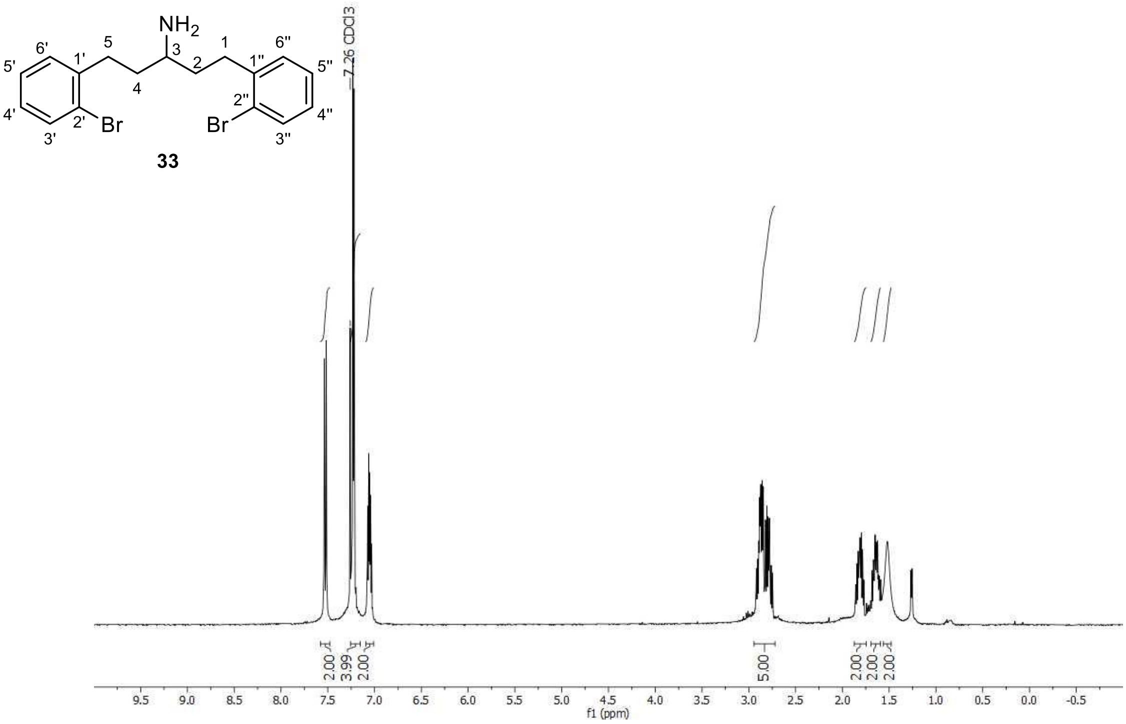

$^{13}C\{^1H\}$ NMR (101 MHz, CDCl₃)

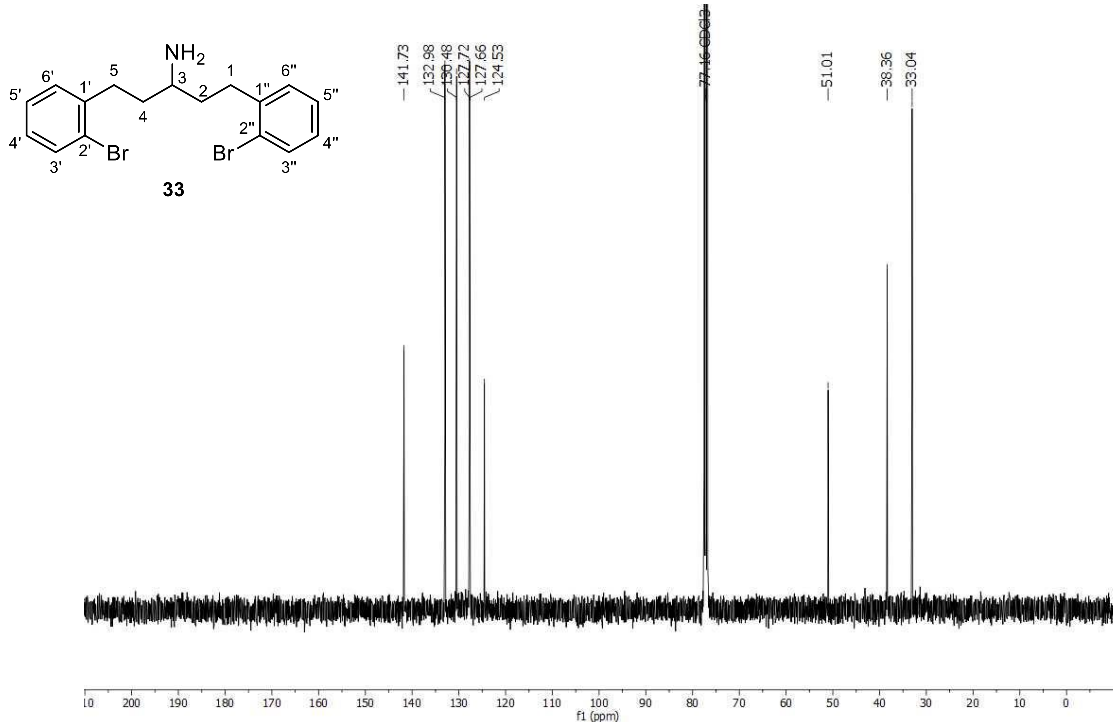

^{1}H NMR (400 MHz, $CDCl_3$)

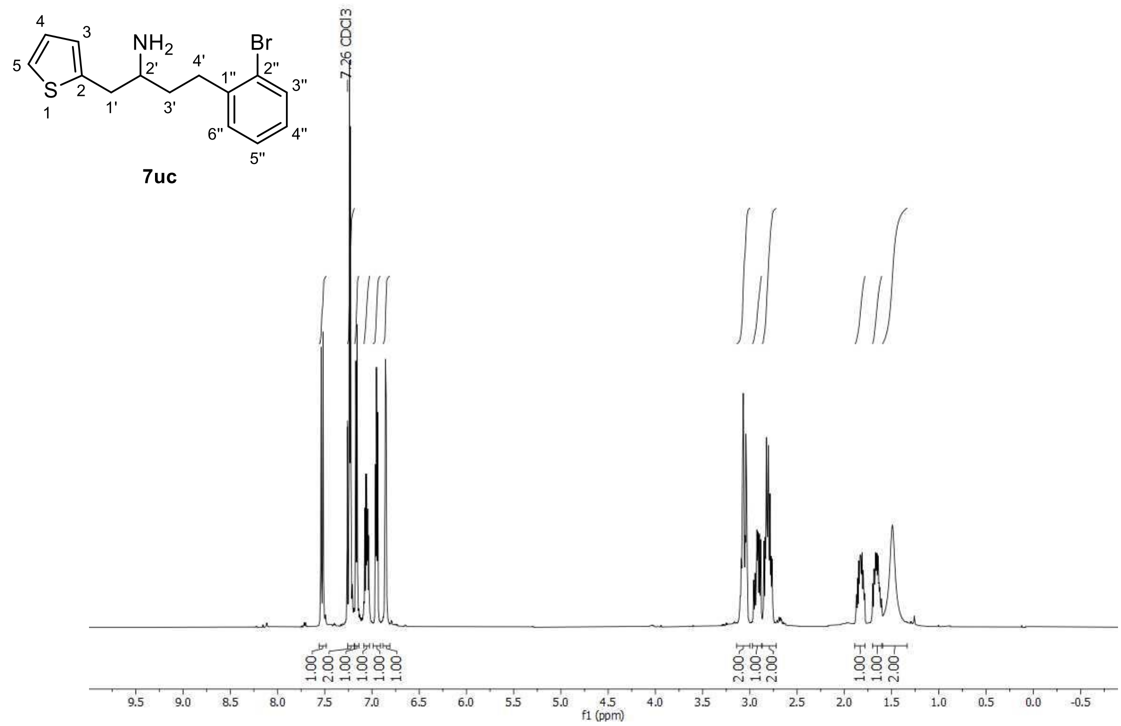

^{13}C{^{1}H} NMR (101 MHz, $CDCl_3$)

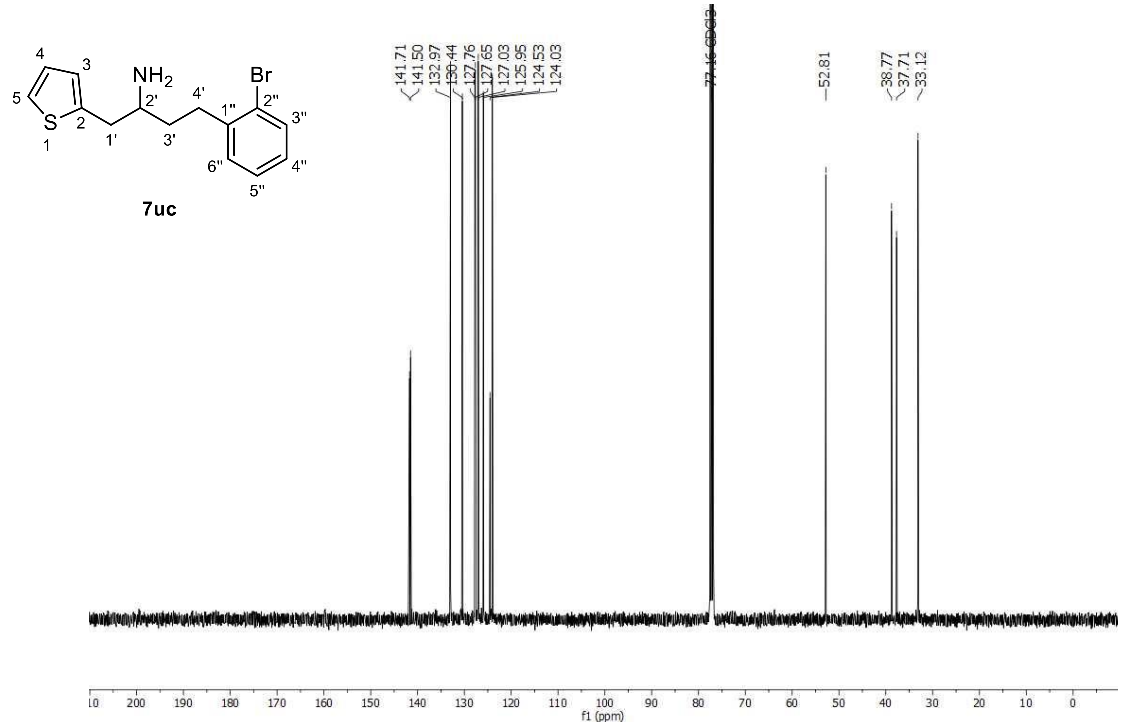

^{1}H NMR (400 MHz, $CDCl_3$)

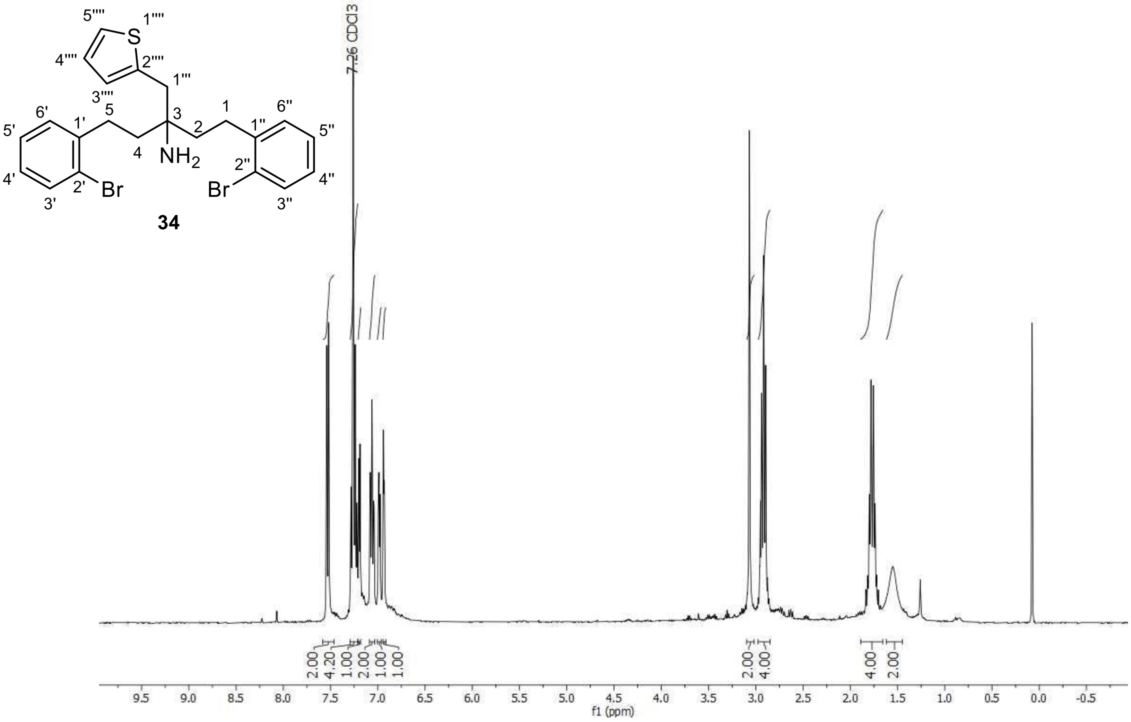

^{13}C{^{1}H} NMR (101 MHz, $CDCl_3$)

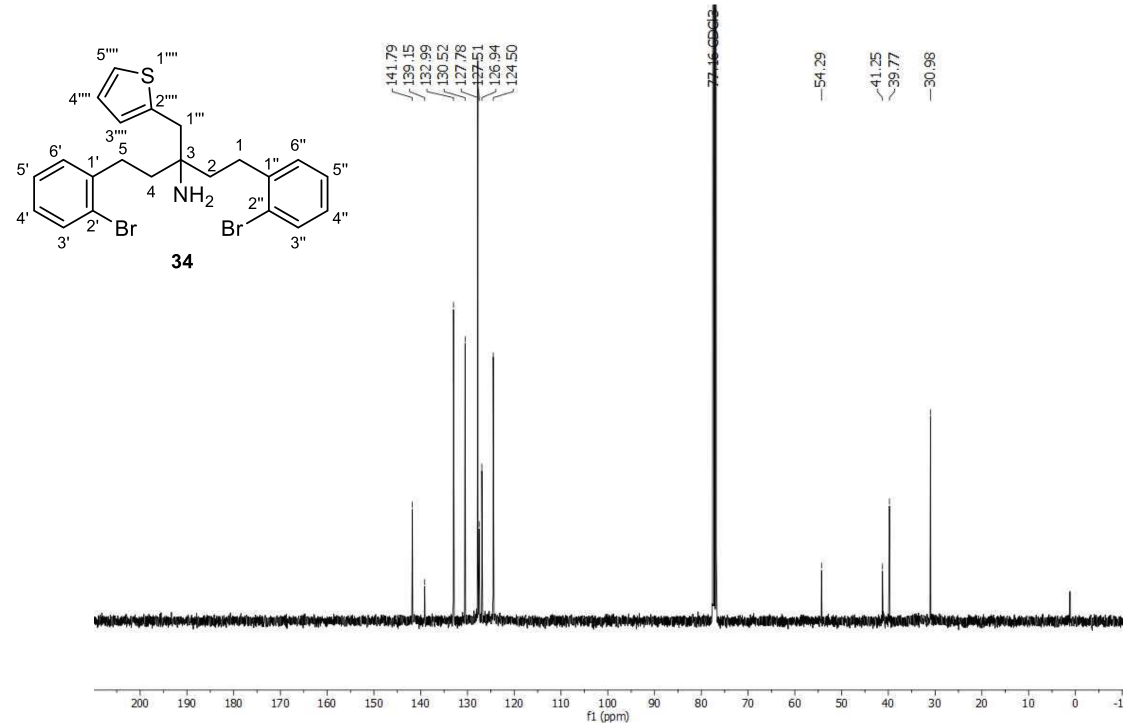

^{1}H NMR (400 MHz, CDCl3)

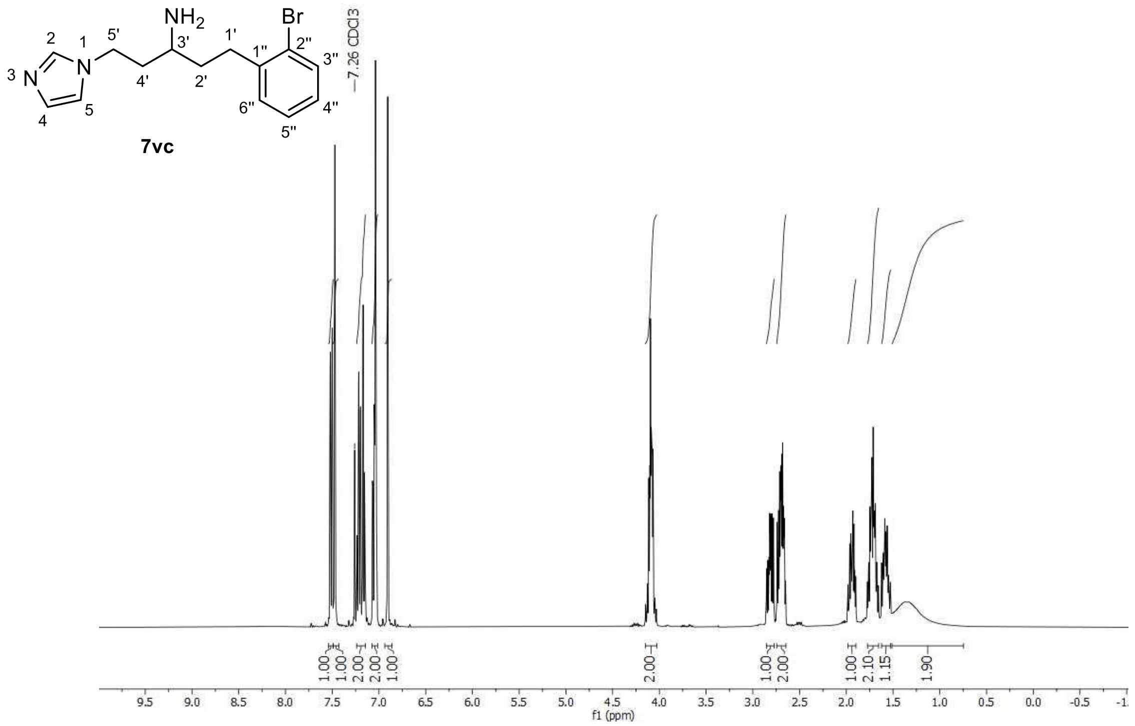

^{13}C{^{1}H} NMR (101 MHz, CDCl3)

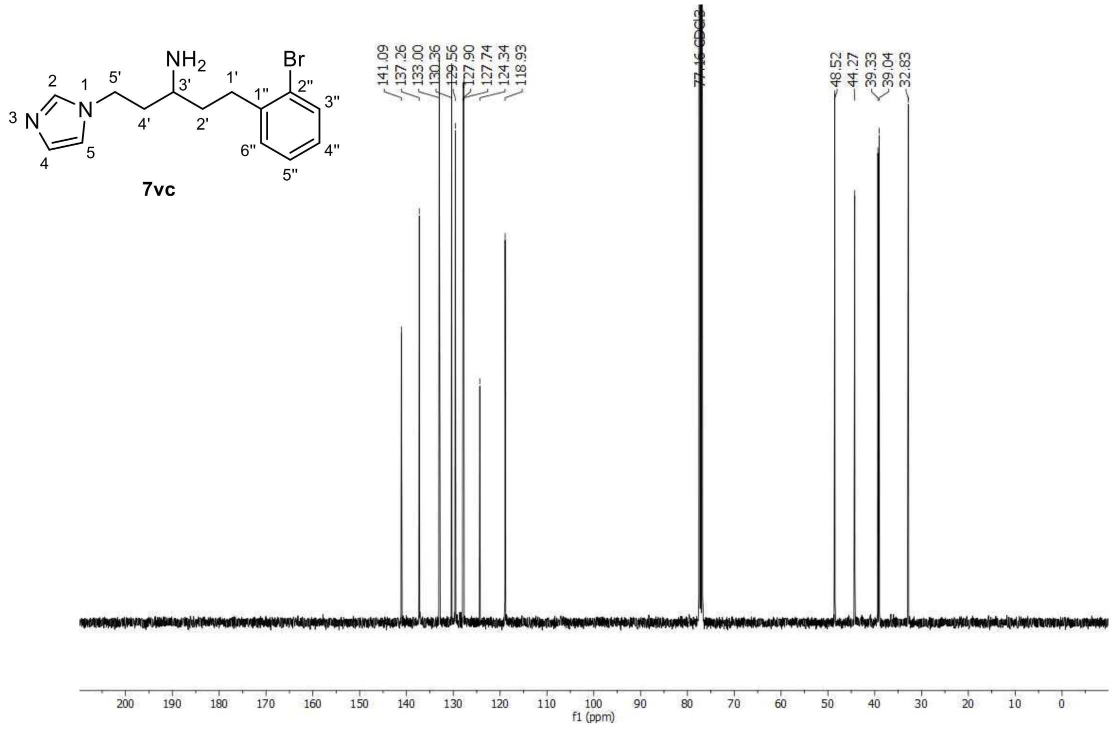

^{1}H NMR (400 MHz, $CDCl_3$)

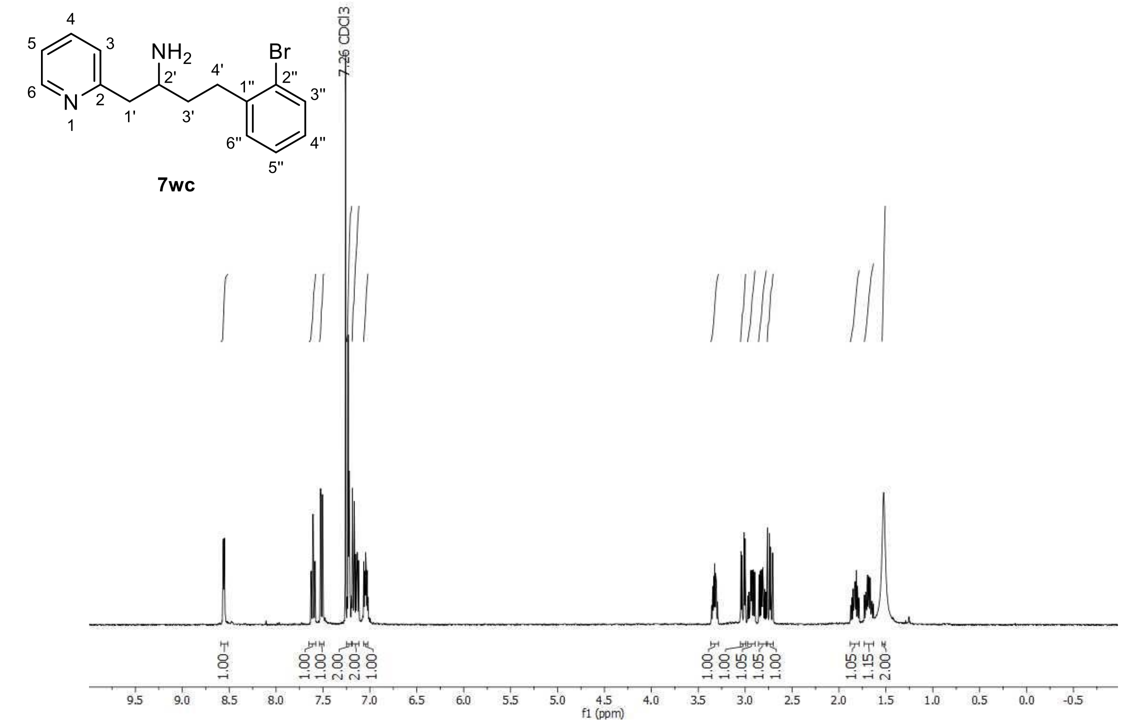

^{13}C{^{1}H} NMR (101 MHz, $CDCl_3$)

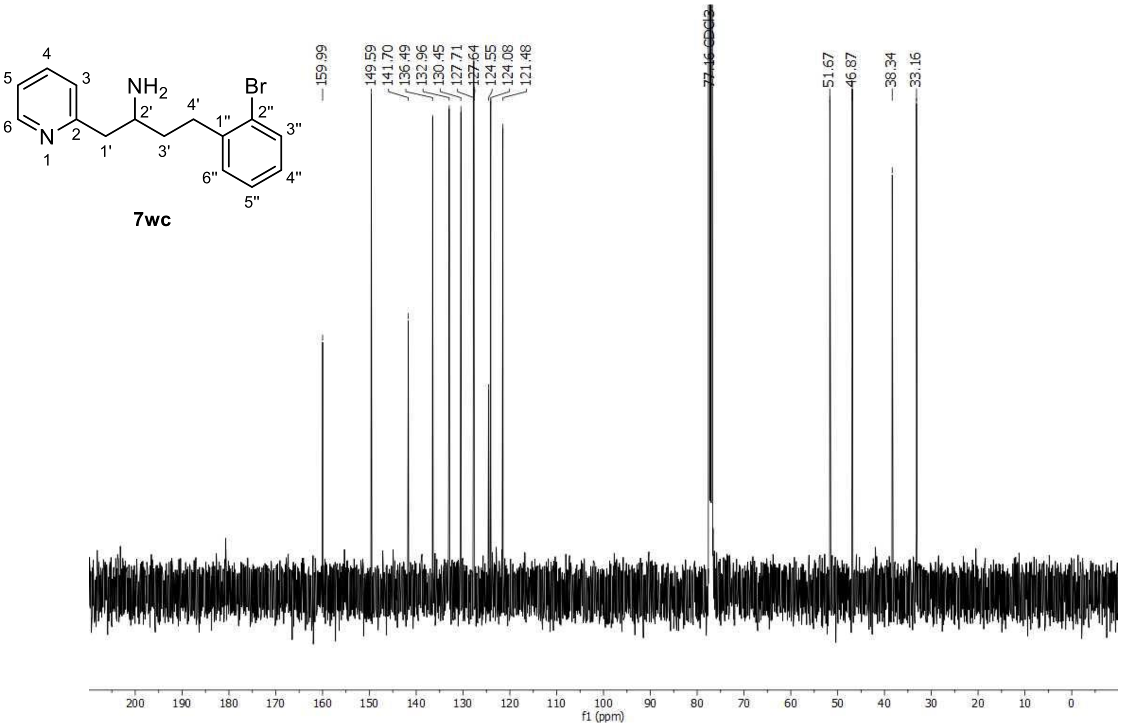

^{1}H NMR (400 MHz, $CDCl_3$)

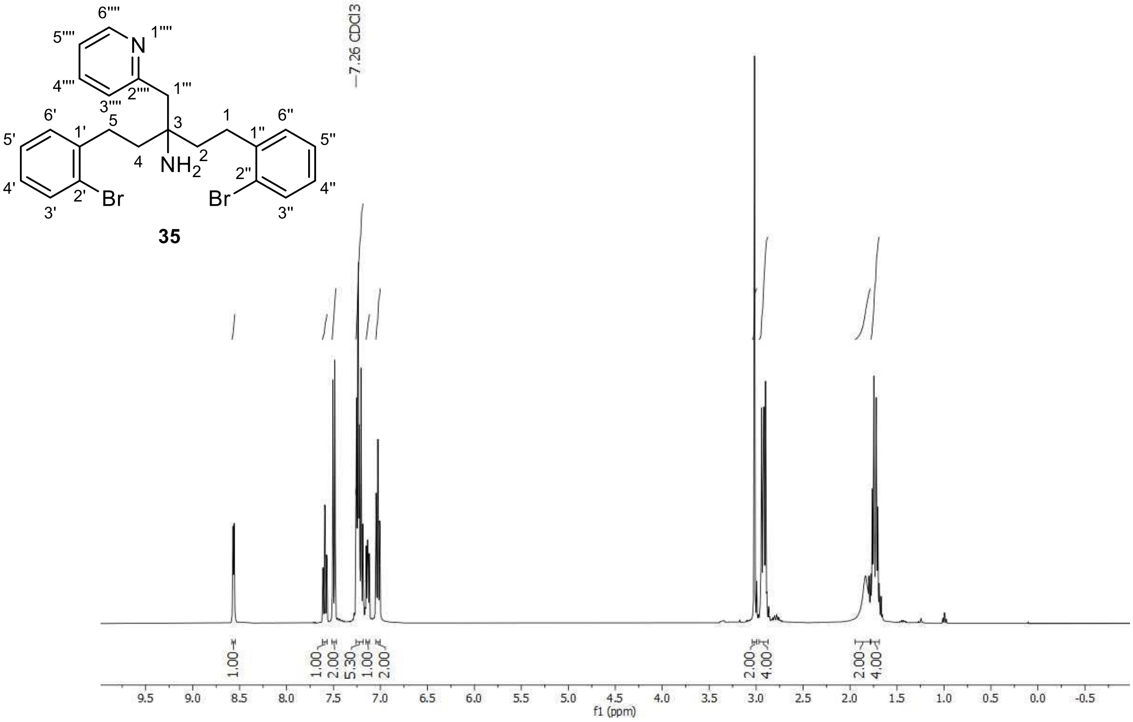

^{13}C{^{1}H} NMR (101 MHz, $CDCl_3$)

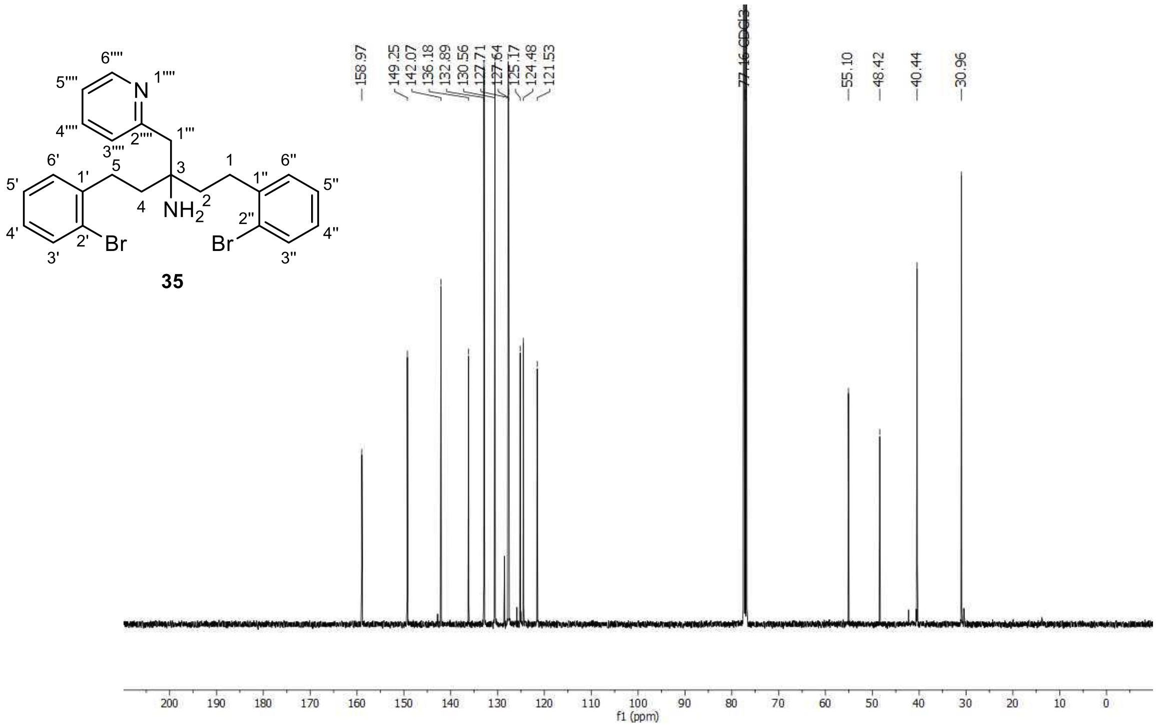

^{1}H NMR (400 MHz, $CDCl_3$)

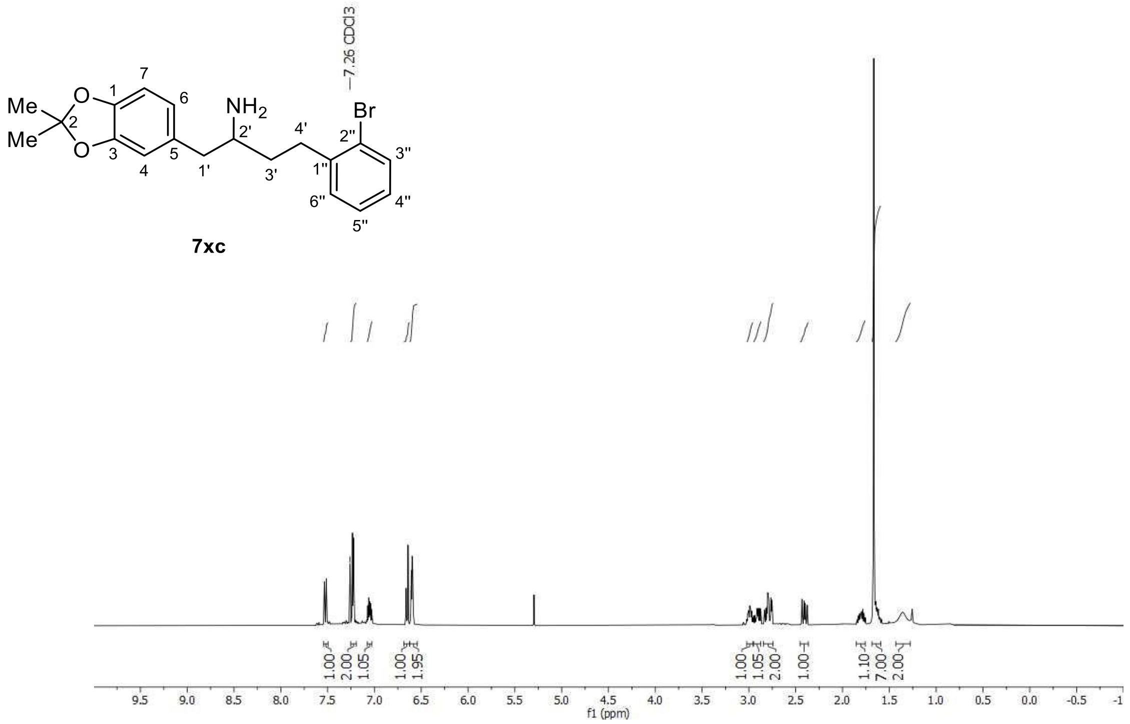

^{13}C{^{1}H} NMR (101 MHz, $CDCl_3$)

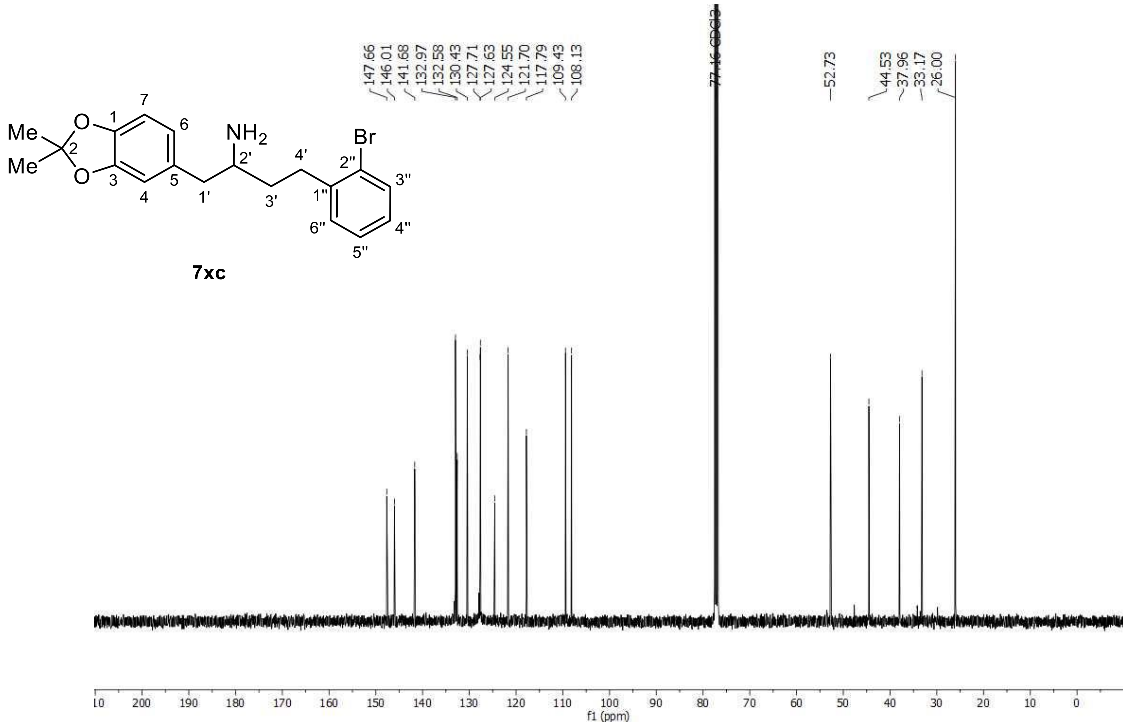

^{1}H NMR (400 MHz, $CDCl_3$)

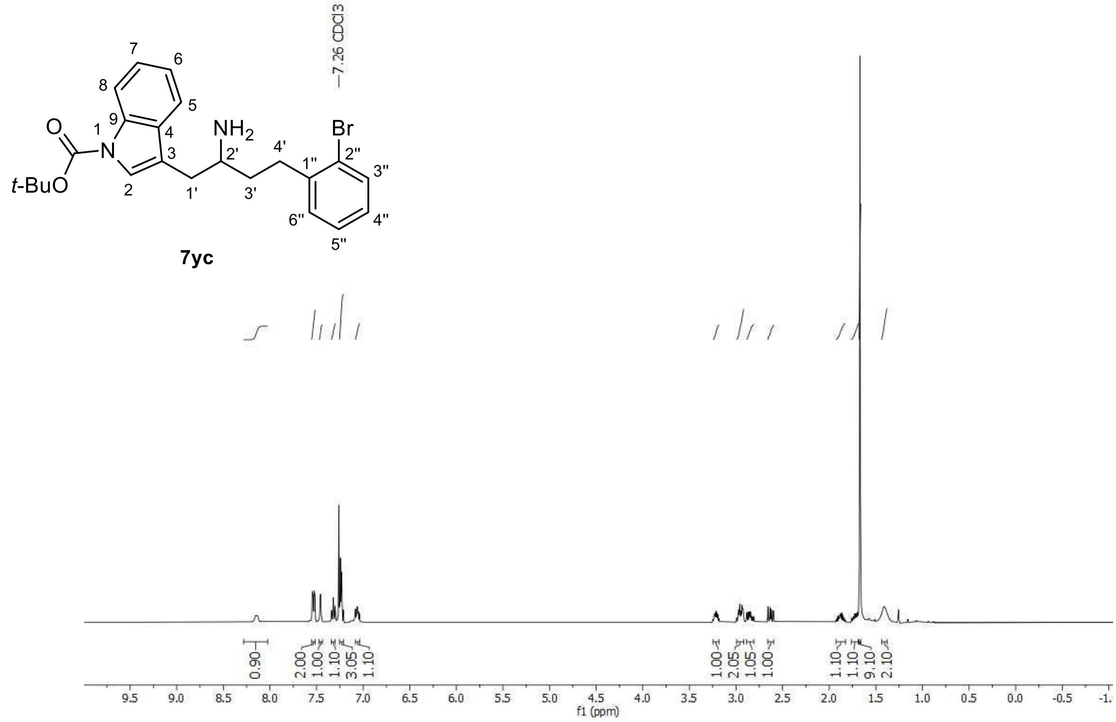

^{13}C{^{1}H} NMR (101 MHz, $CDCl_3$)

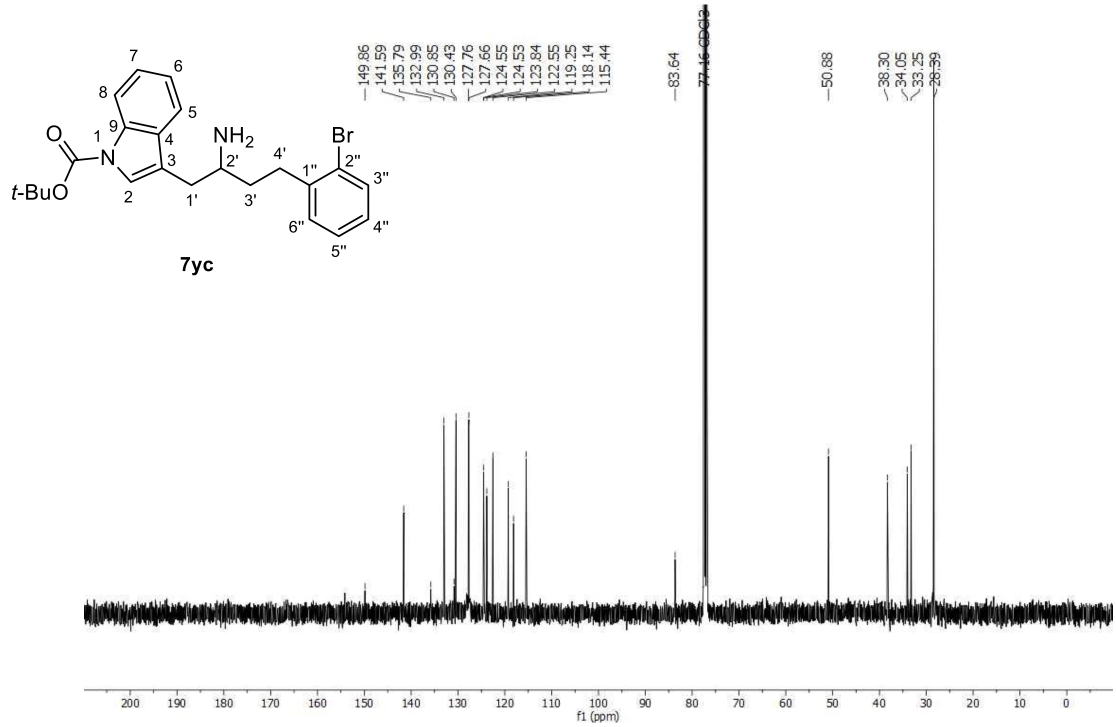

^{1}H NMR (400 MHz, CDCl3)

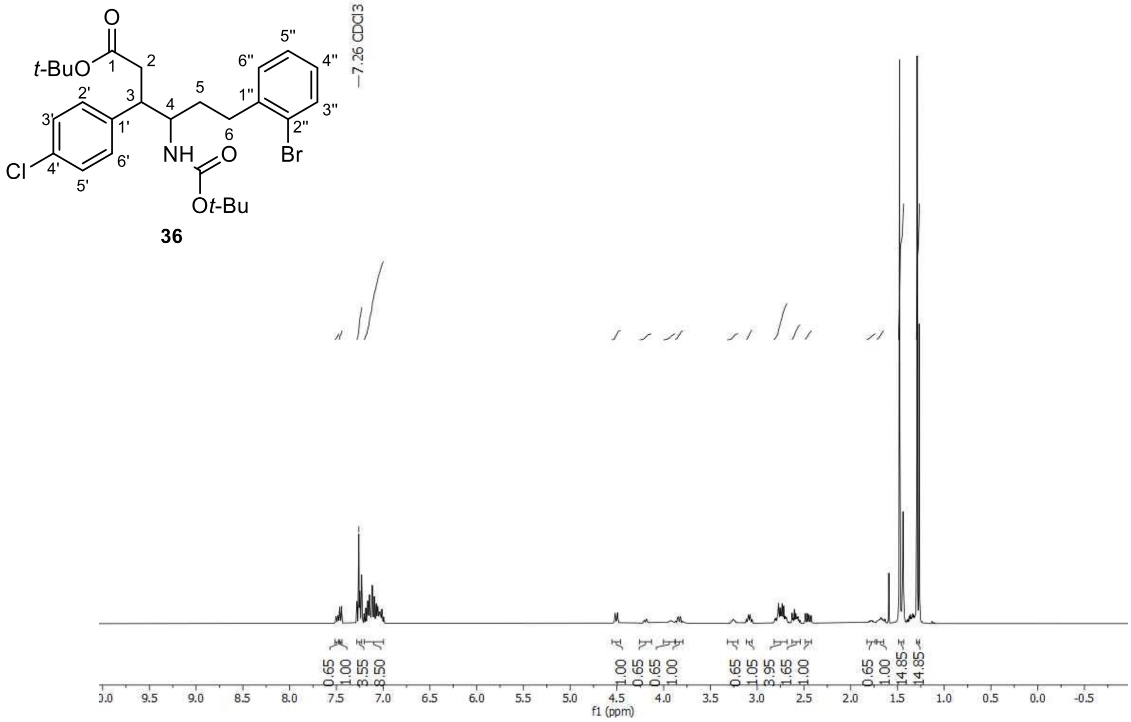

^{13}C{^{1}H} NMR (101 MHz, CDCl3)

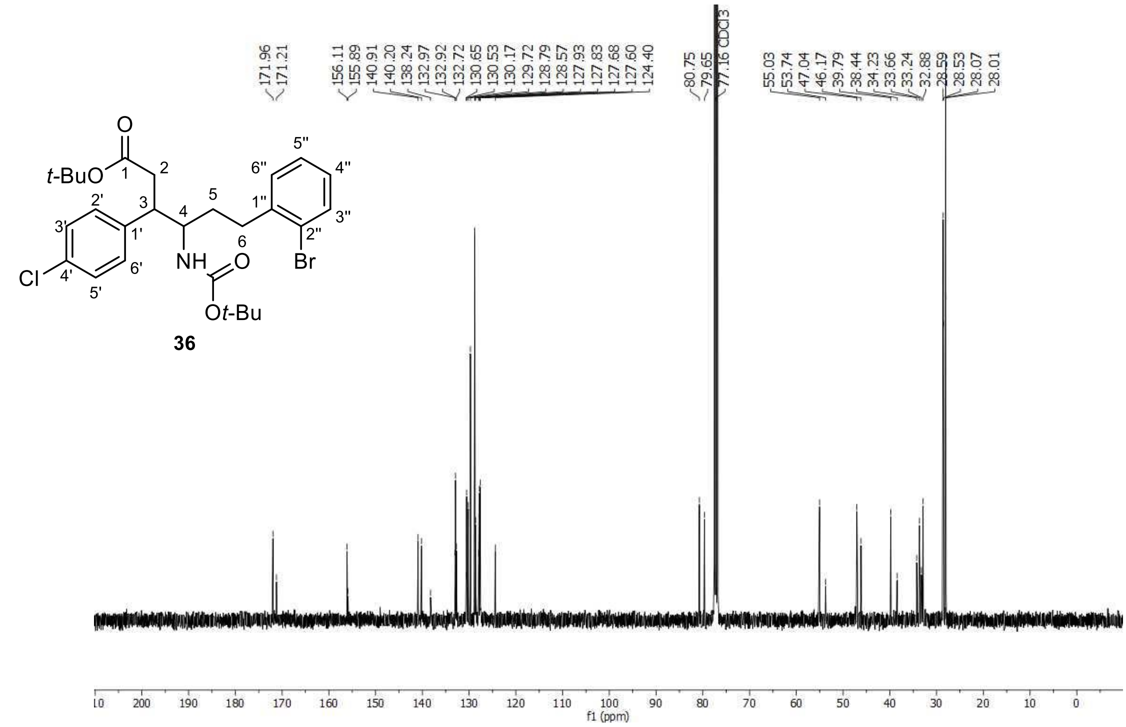

^{1}H NMR (400 MHz, $CDCl_3$)

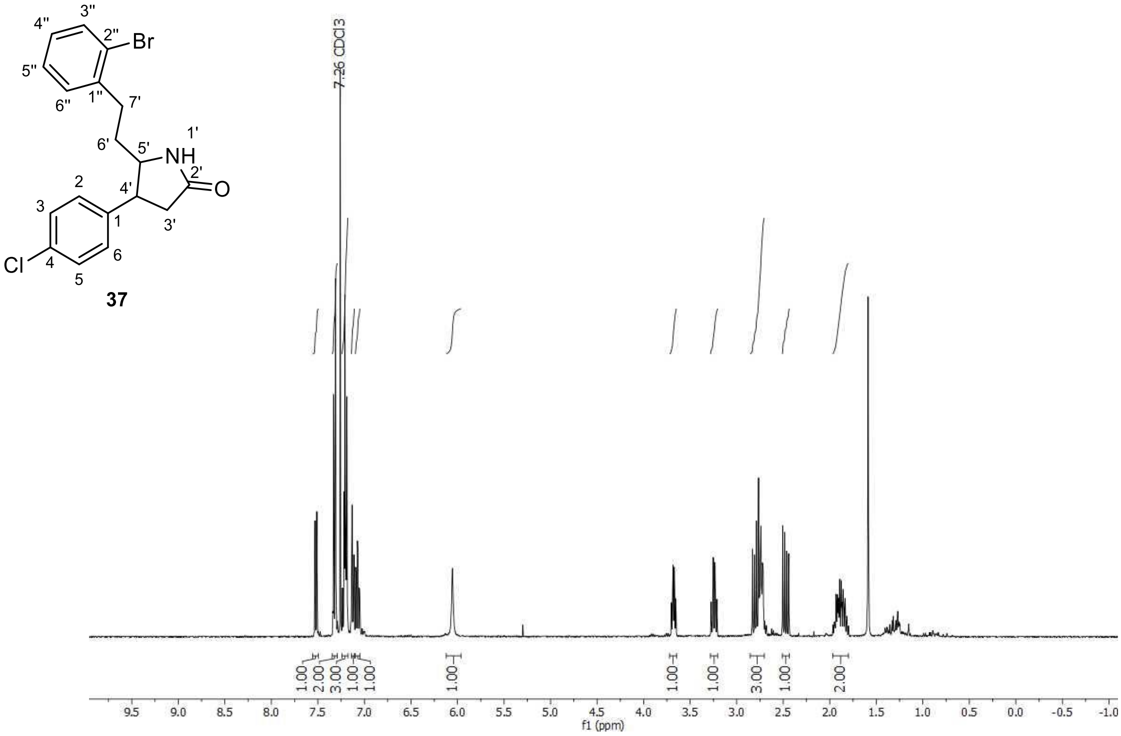

^{13}C{^{1}H} NMR (101 MHz, $CDCl_3$)

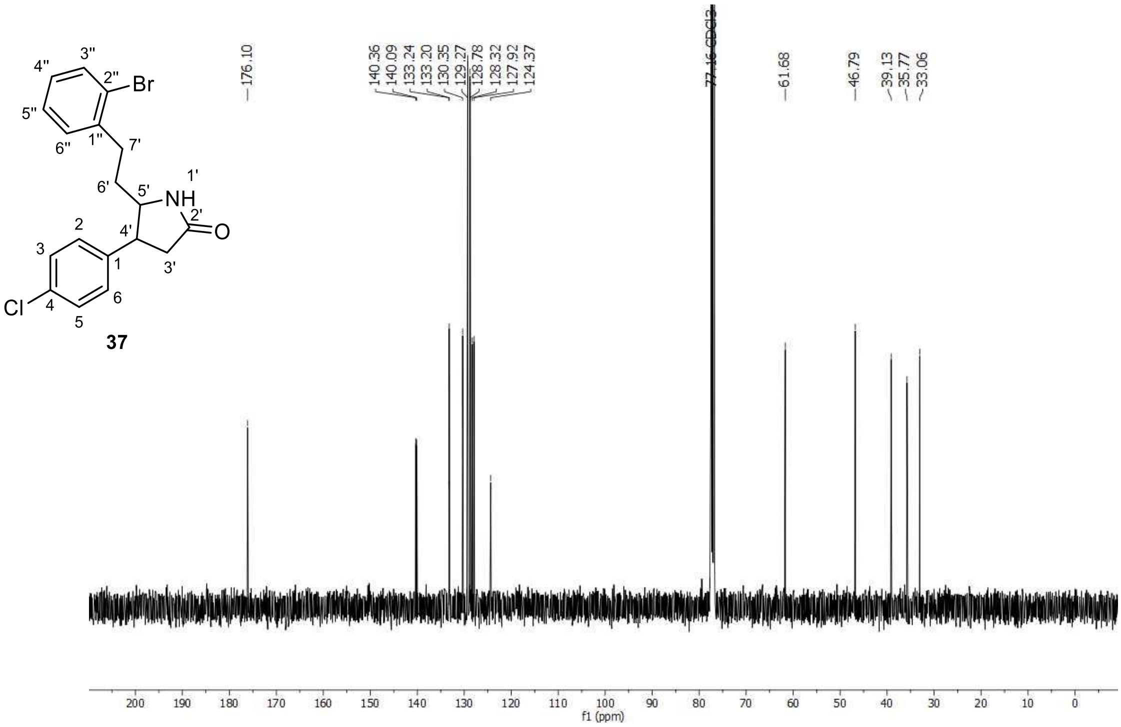

^{1}H NMR (400 MHz, $CDCl_3$)

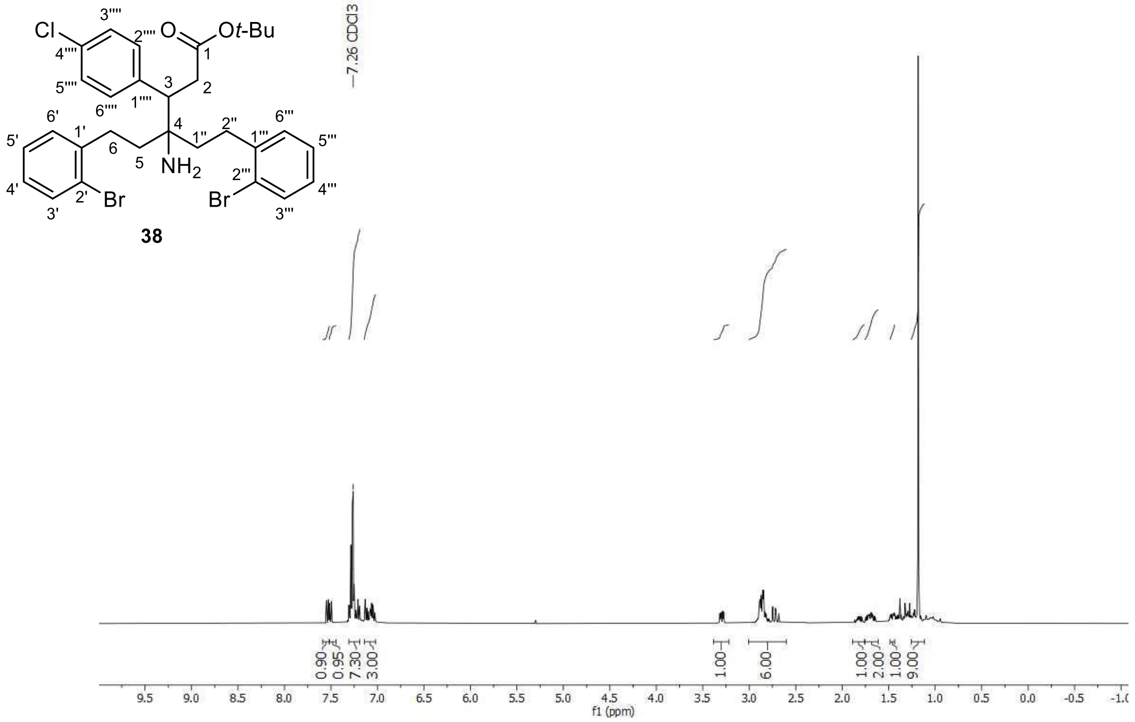

^{13}C{^{1}H} NMR (101 MHz, $CDCl_3$)

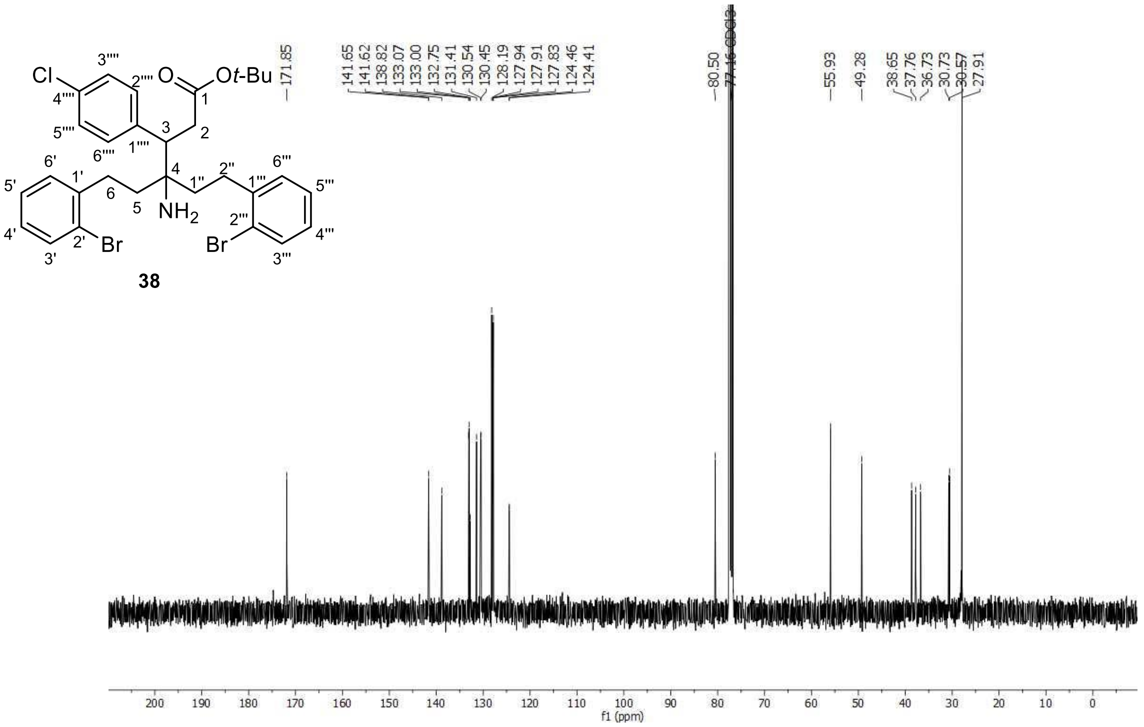

^{1}H NMR (400 MHz, CDCl₃)

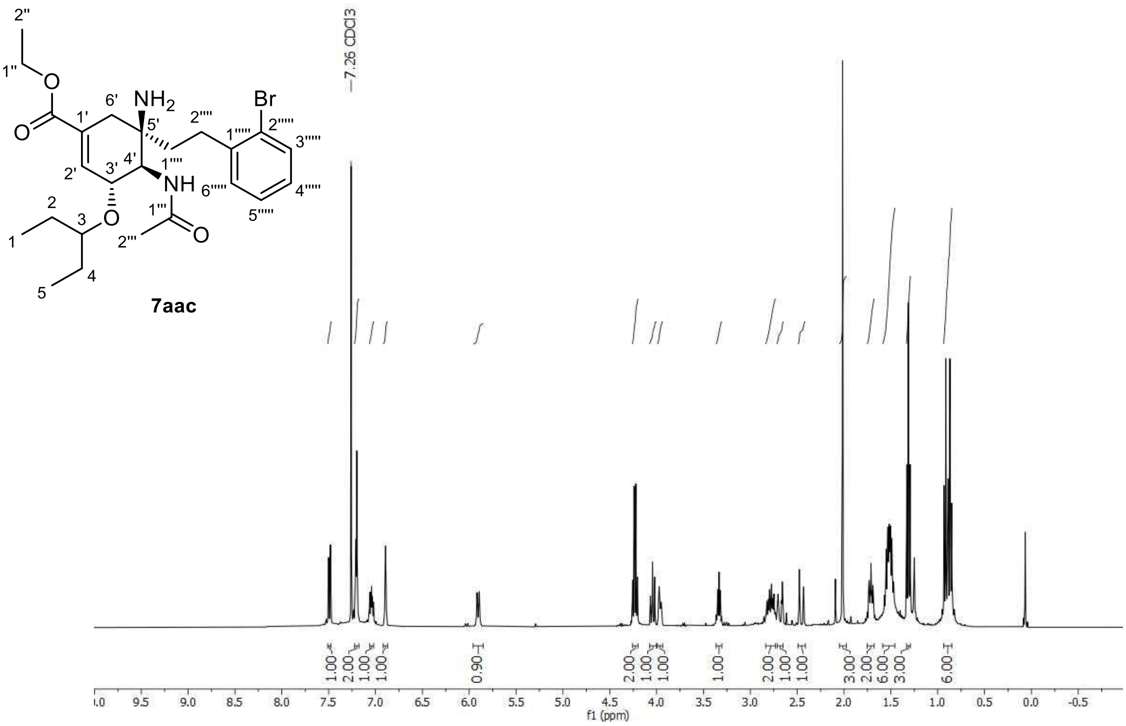

^{13}C{^{1}H} NMR (101 MHz, CDCl₃)

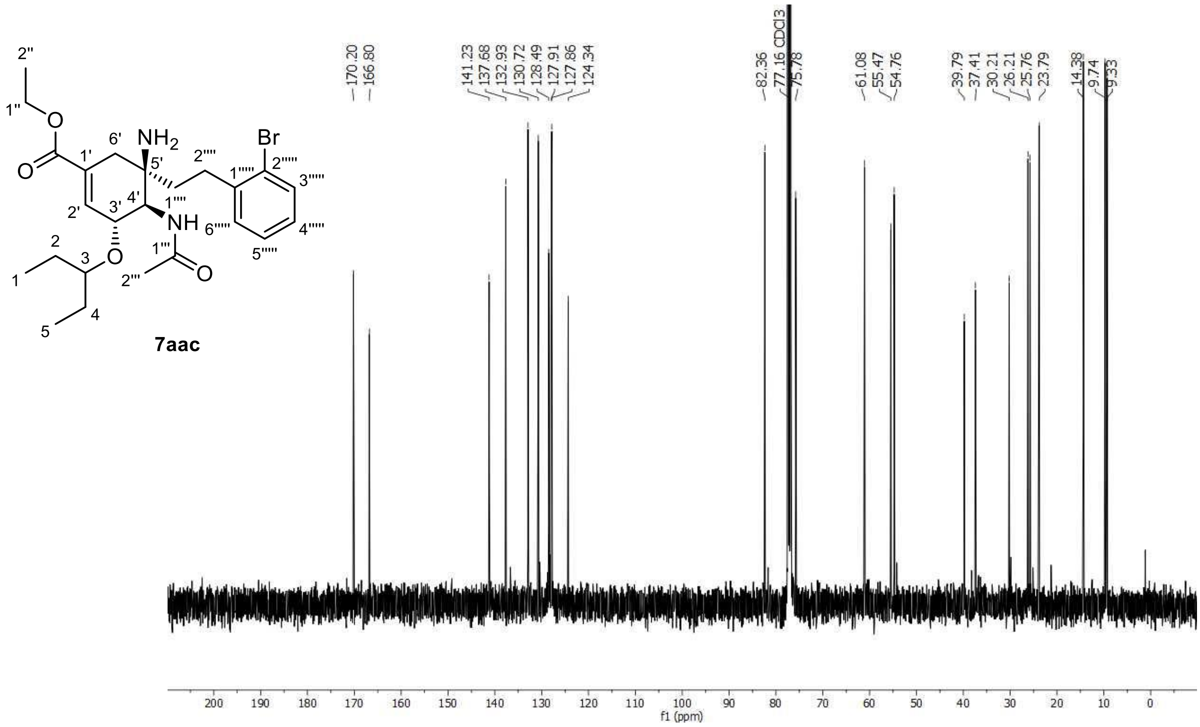

^{1}H NMR (400 MHz, CDCl$_3$)

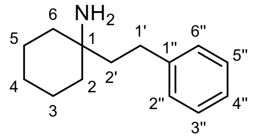

7ab

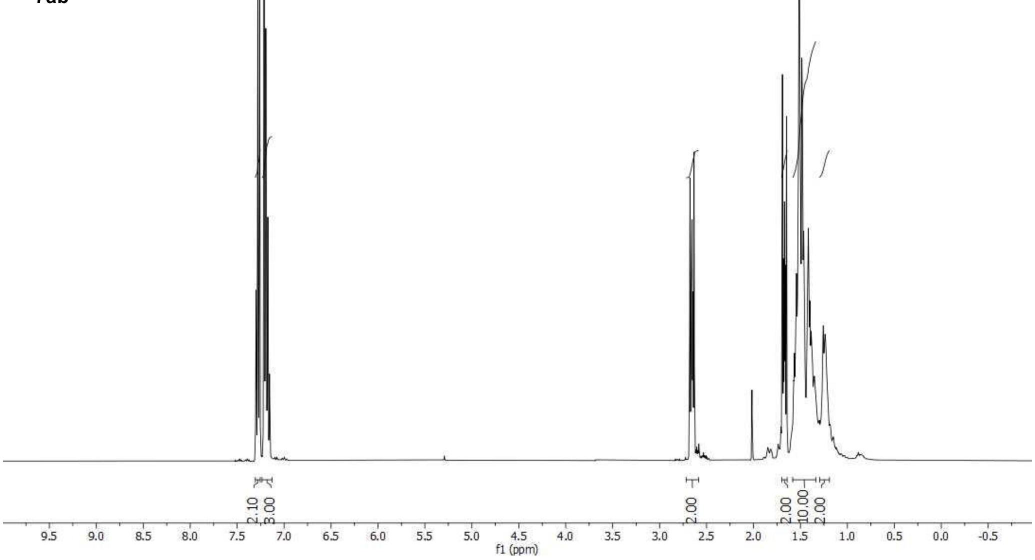

^{13}C{^{1}H} NMR (101 MHz, CDCl$_3$)

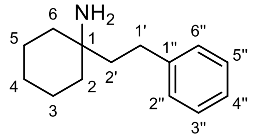

7ab

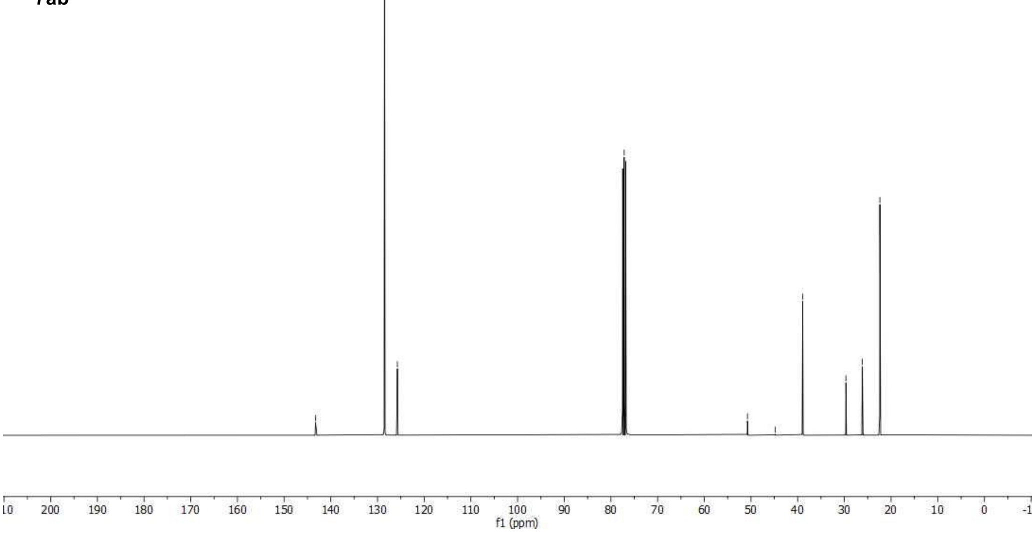

^{1}H NMR (400 MHz, CDCl3)

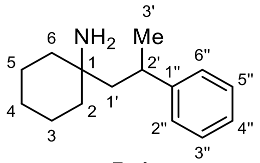

7ad

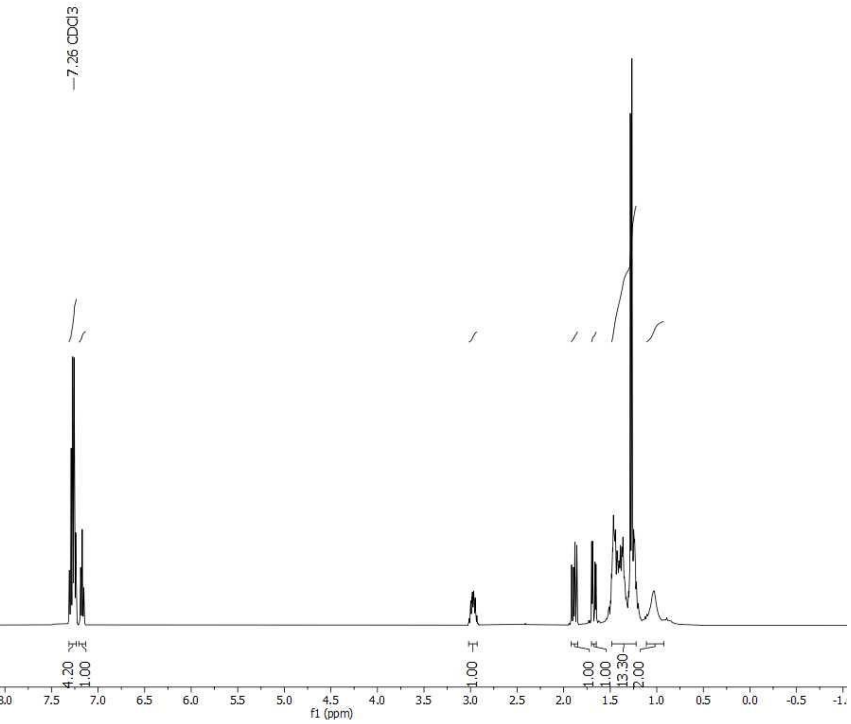

^{13}C{^{1}H} NMR (101 MHz, CDCl3)

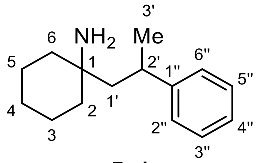

7ad

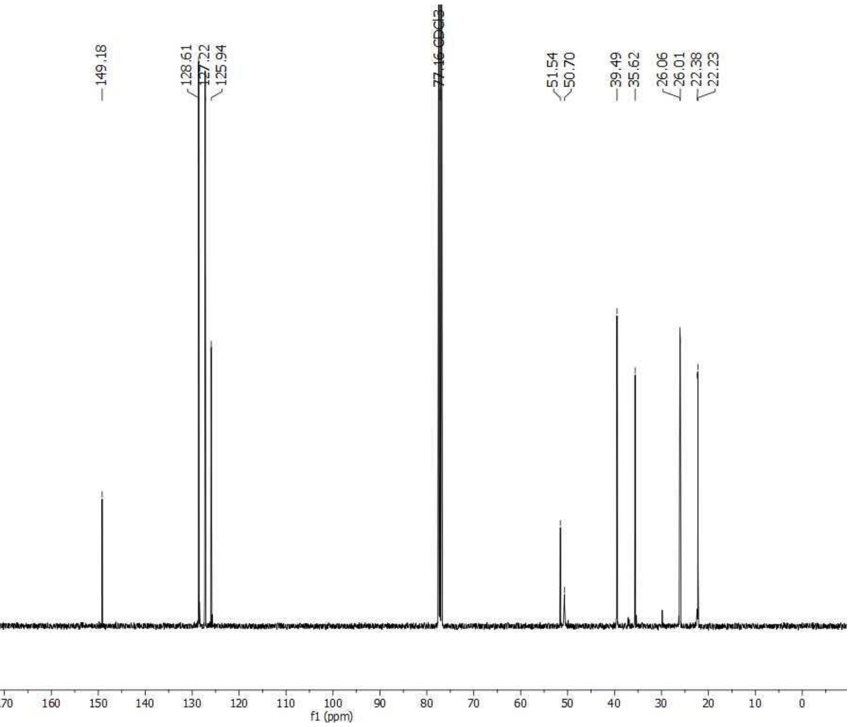

^{1}H NMR (400 MHz, CDCl3)

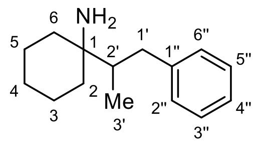

7ae

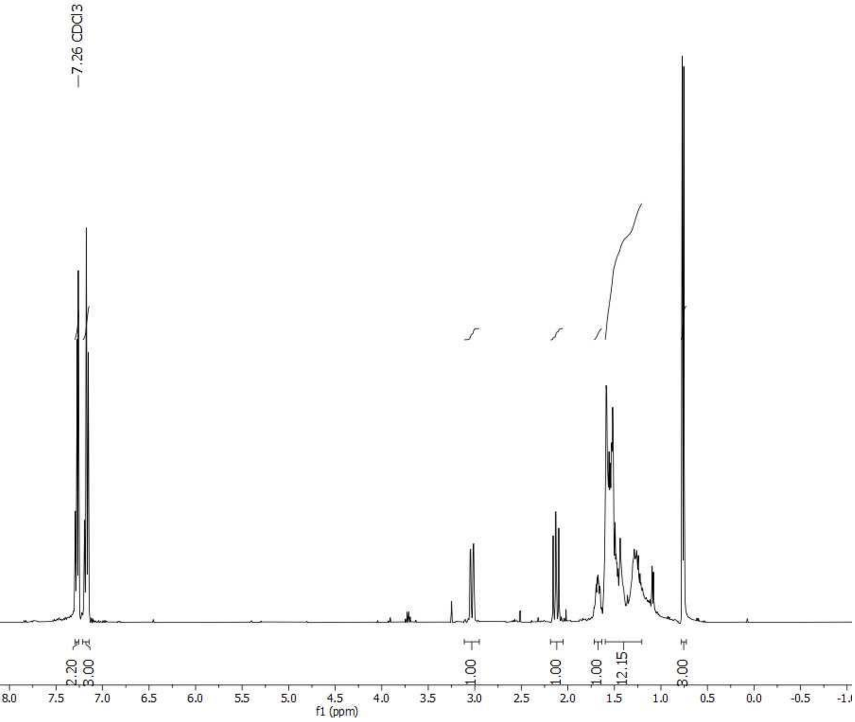

^{13}C{^{1}H} NMR (101 MHz, CDCl3)

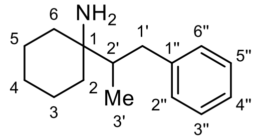

7ae

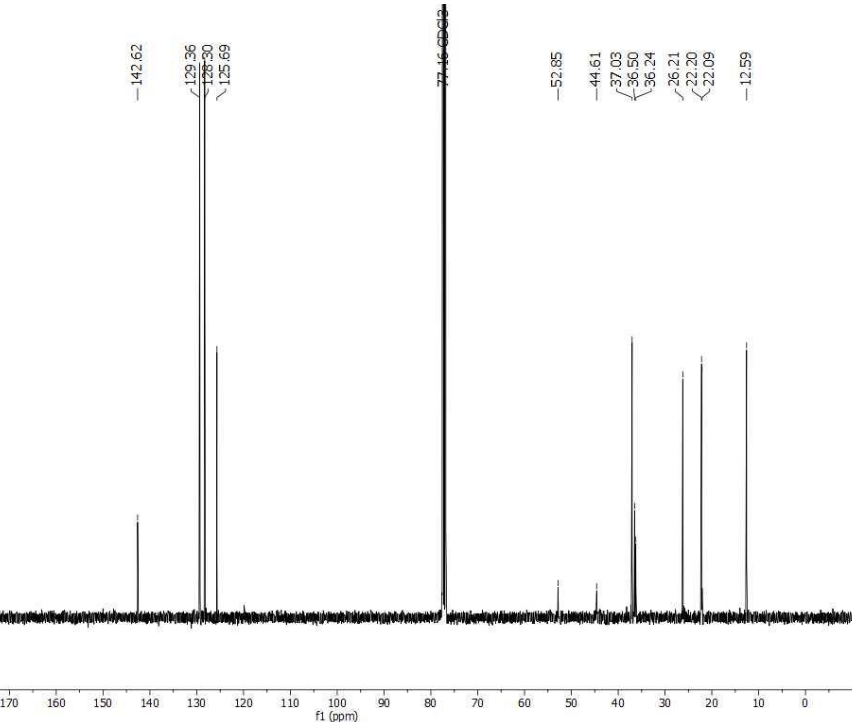

^{1}H NMR (400 MHz, CDCl3)

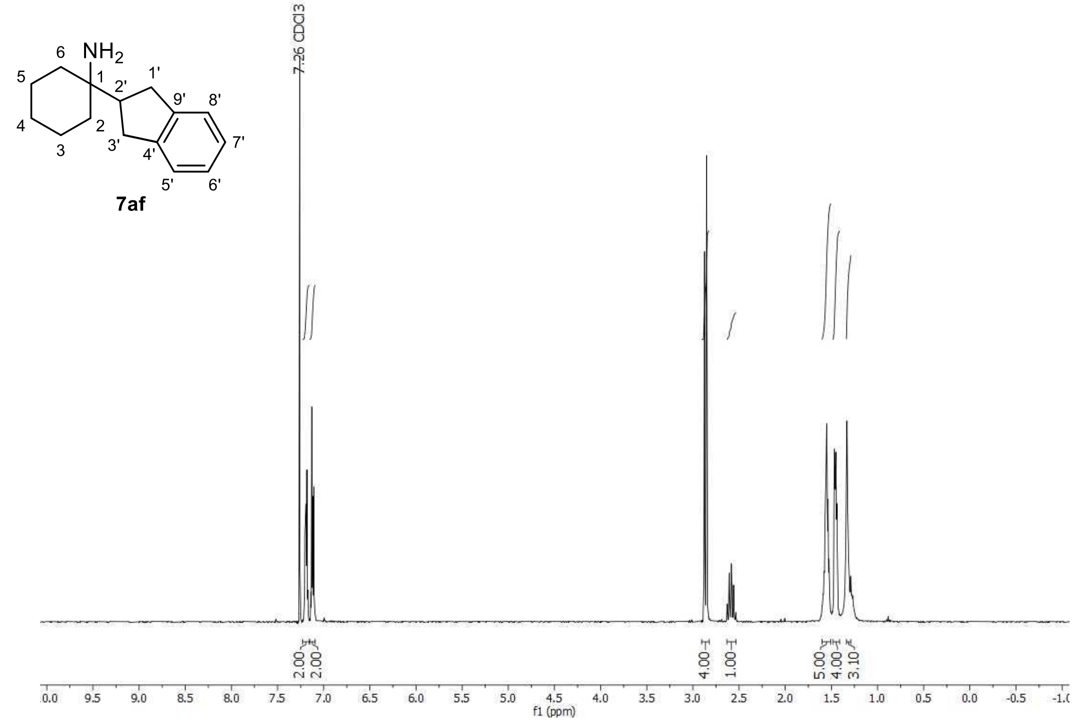

^{13}C{^{1}H} NMR (126 MHz, CDCl3)

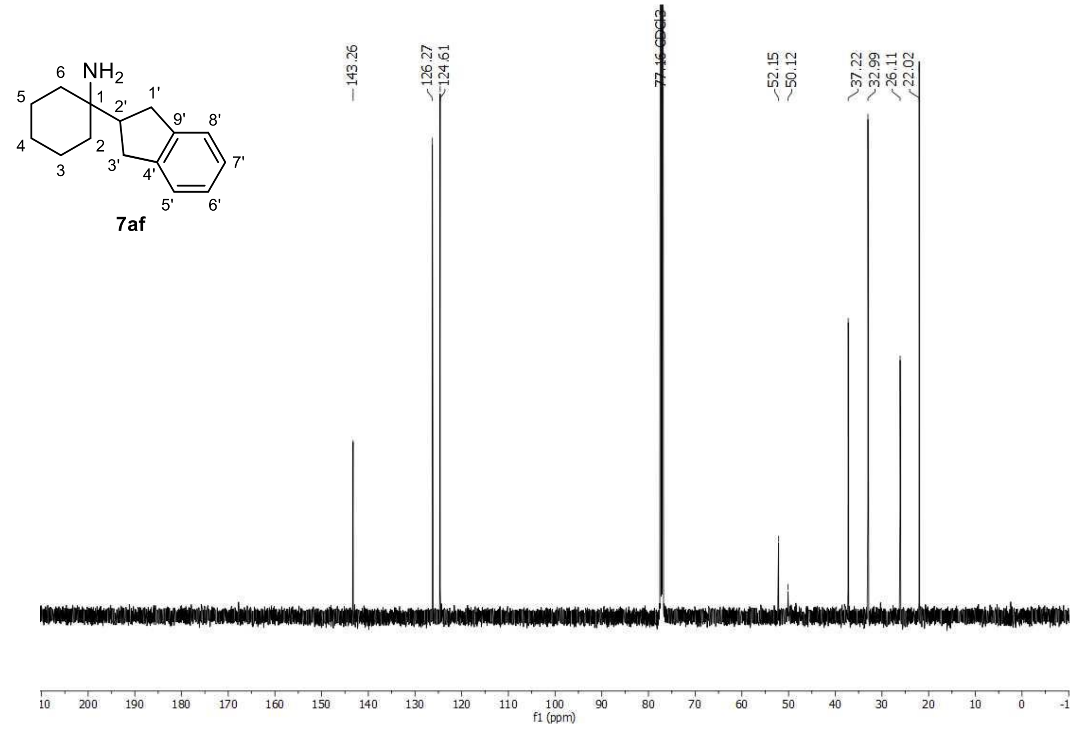

^{1}H NMR (400 MHz, $CDCl_3$)

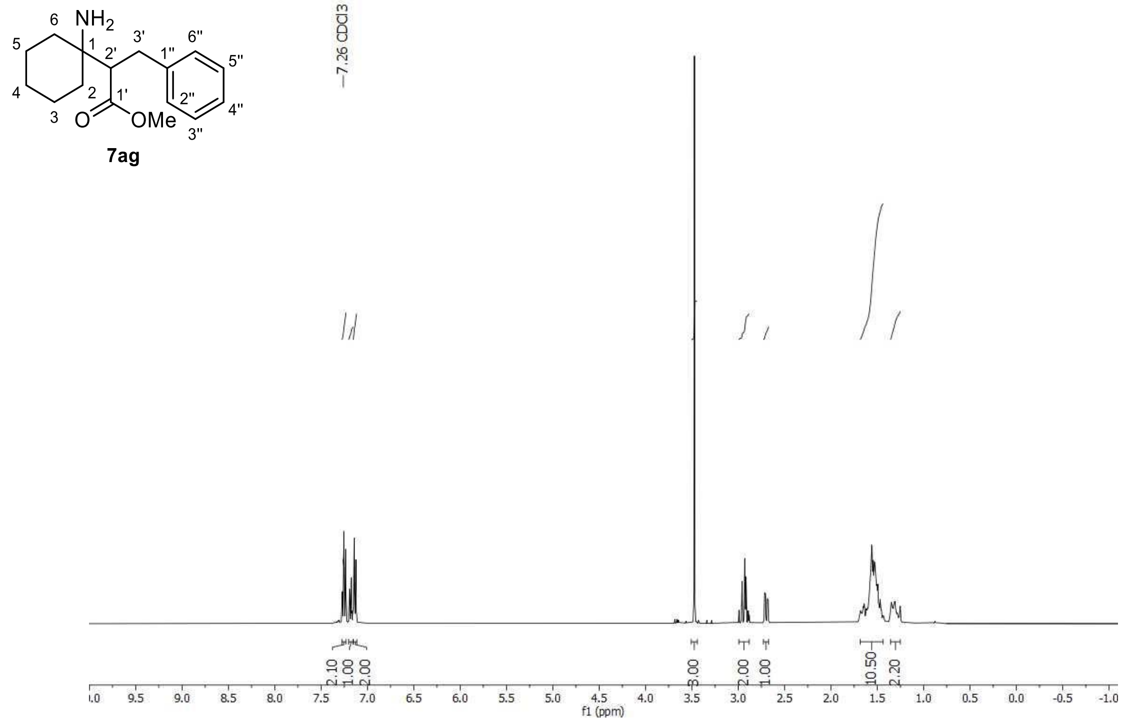

$^{13}C\{^{1}H\}$ NMR (101 MHz, $CDCl_3$)

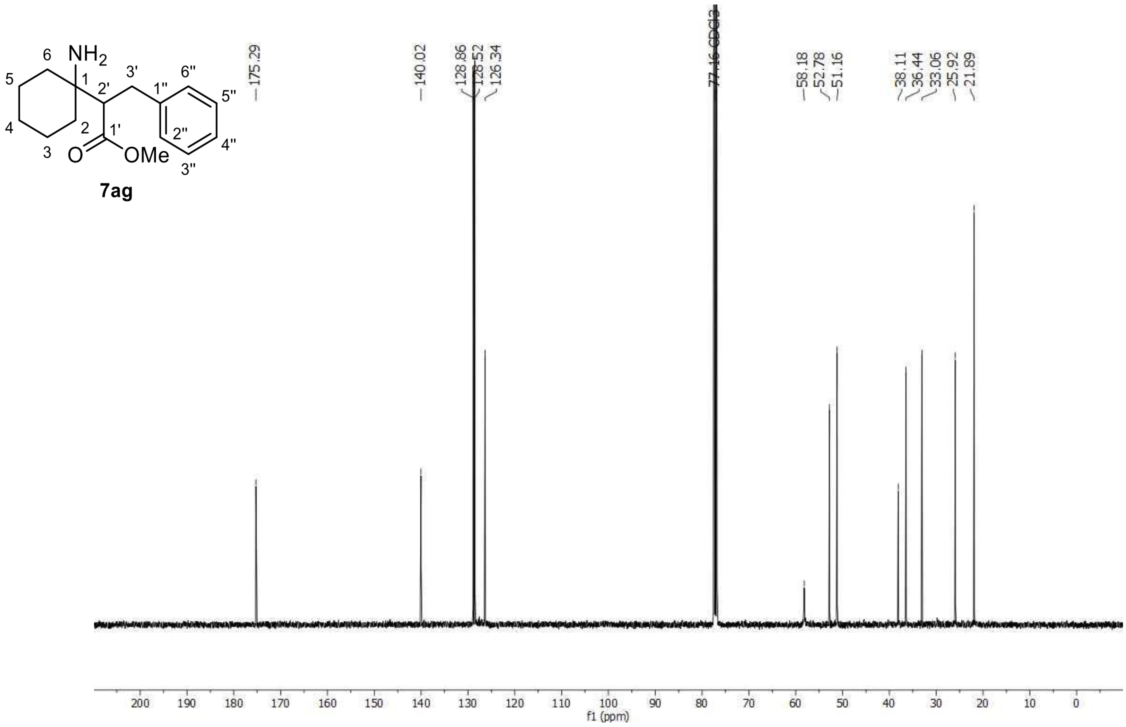

^{1}H NMR (400 MHz, CDCl3)

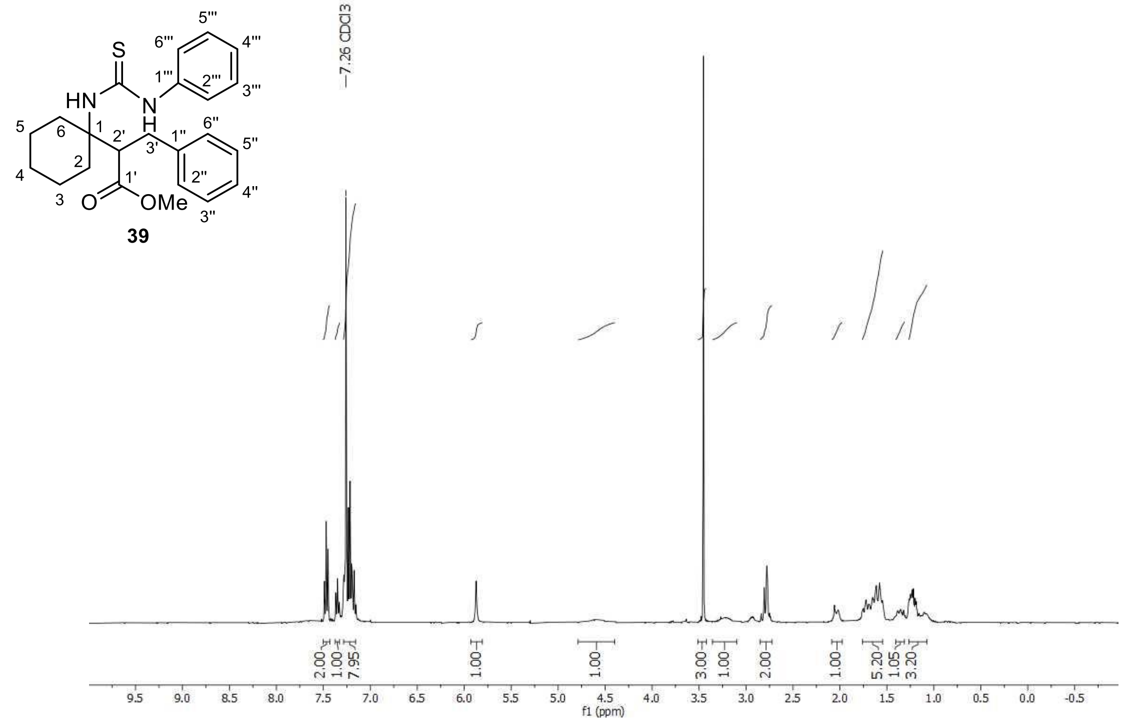

^{13}C{^{1}H} NMR (101 MHz, CDCl3)

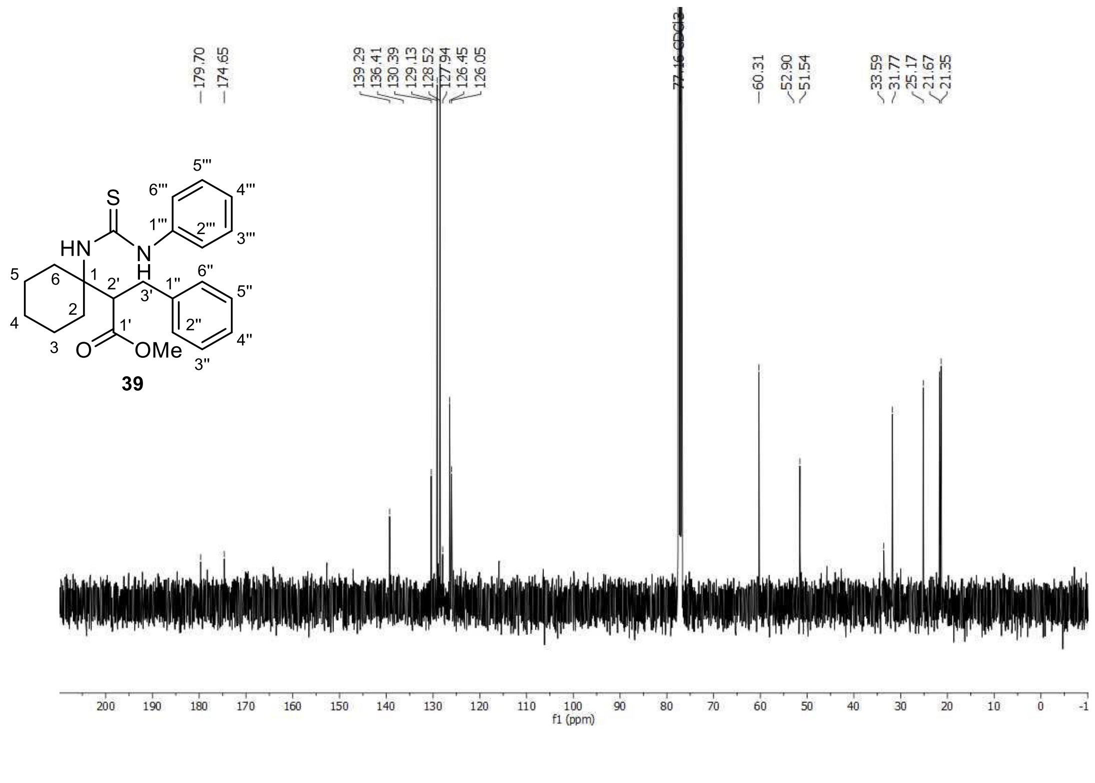

^{1}H NMR (400 MHz, CDCl3)

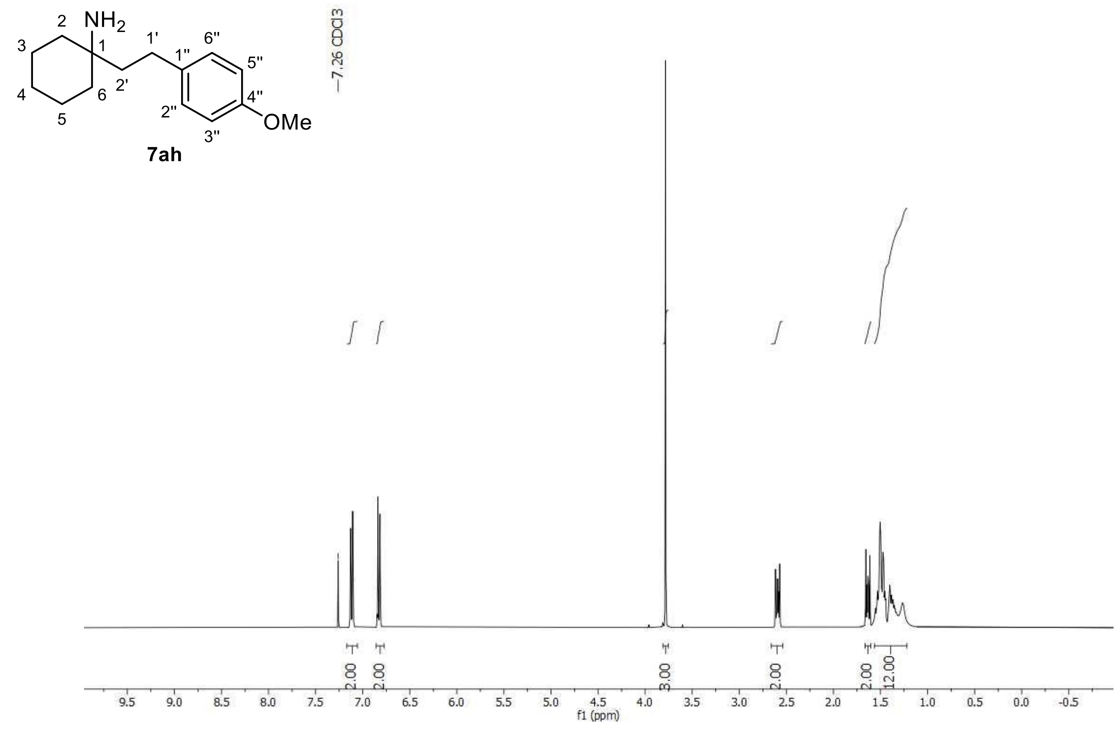

^{13}C{^{1}H} NMR (101 MHz, CDCl3)

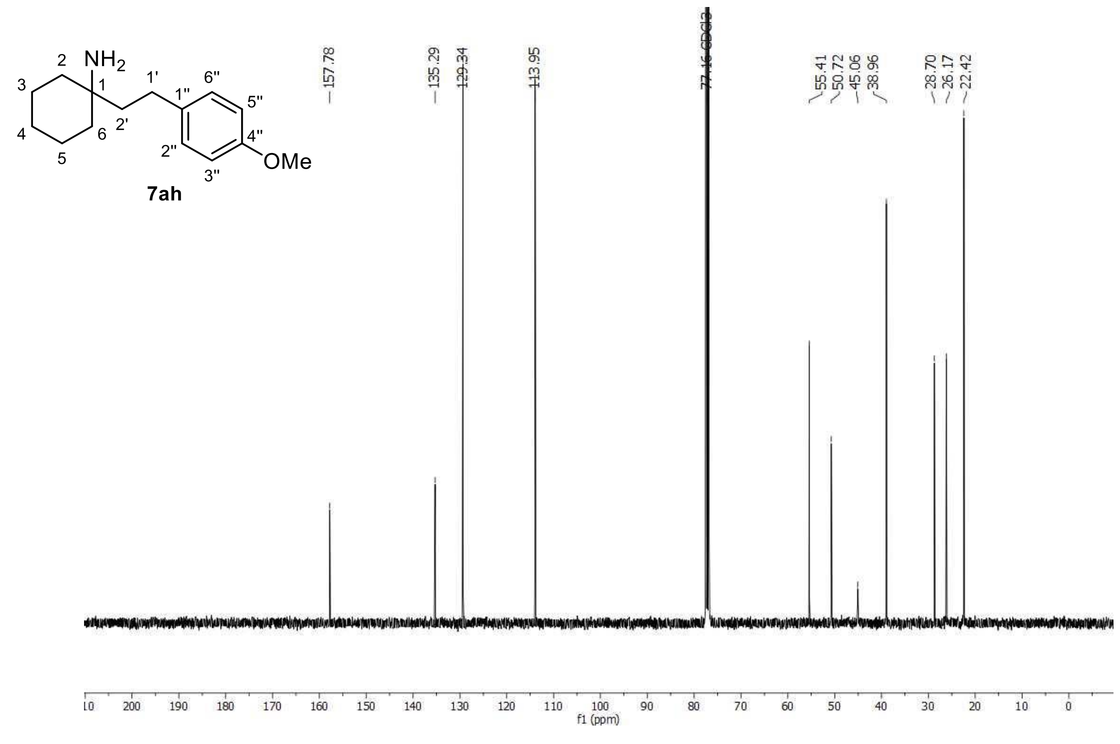

^{1}H NMR (400 MHz, CDCl₃)

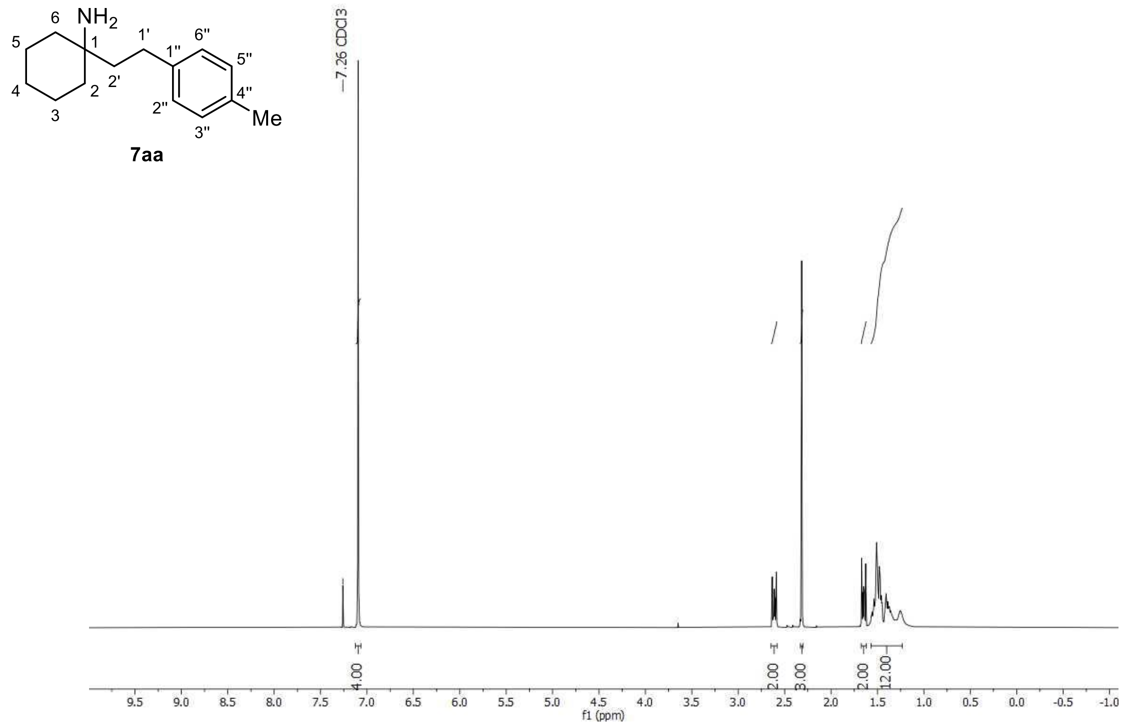

^{13}C{^{1}H} NMR (101 MHz, CDCl₃)

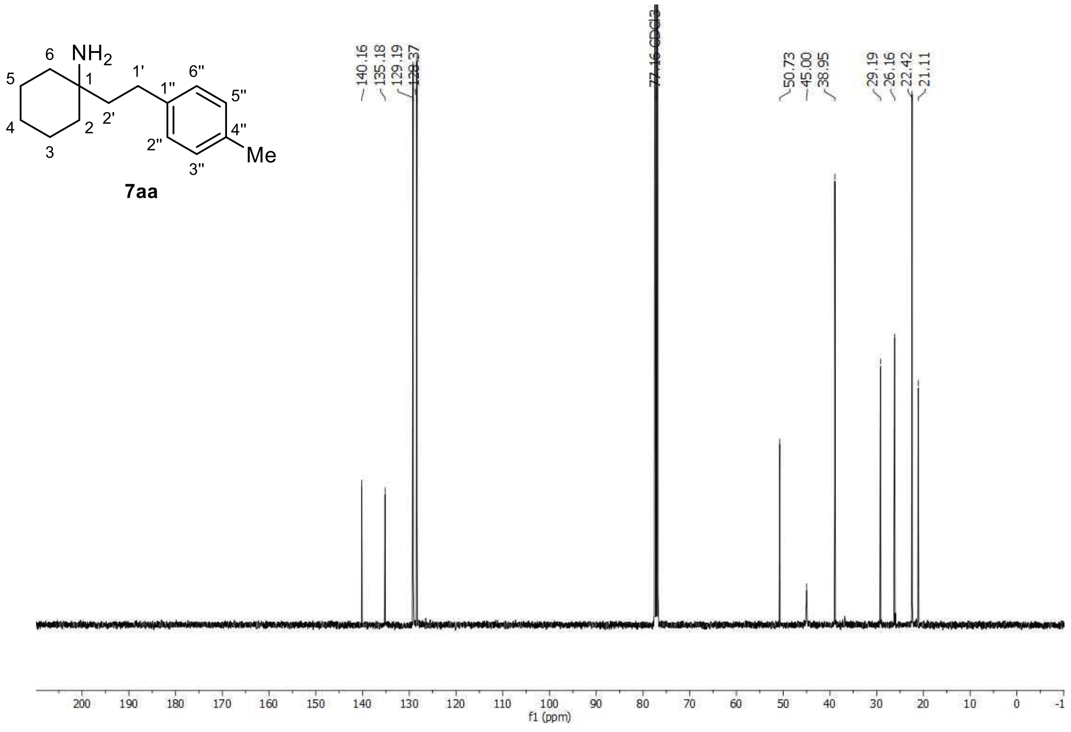

^{1}H NMR (400 MHz, CDCl3)

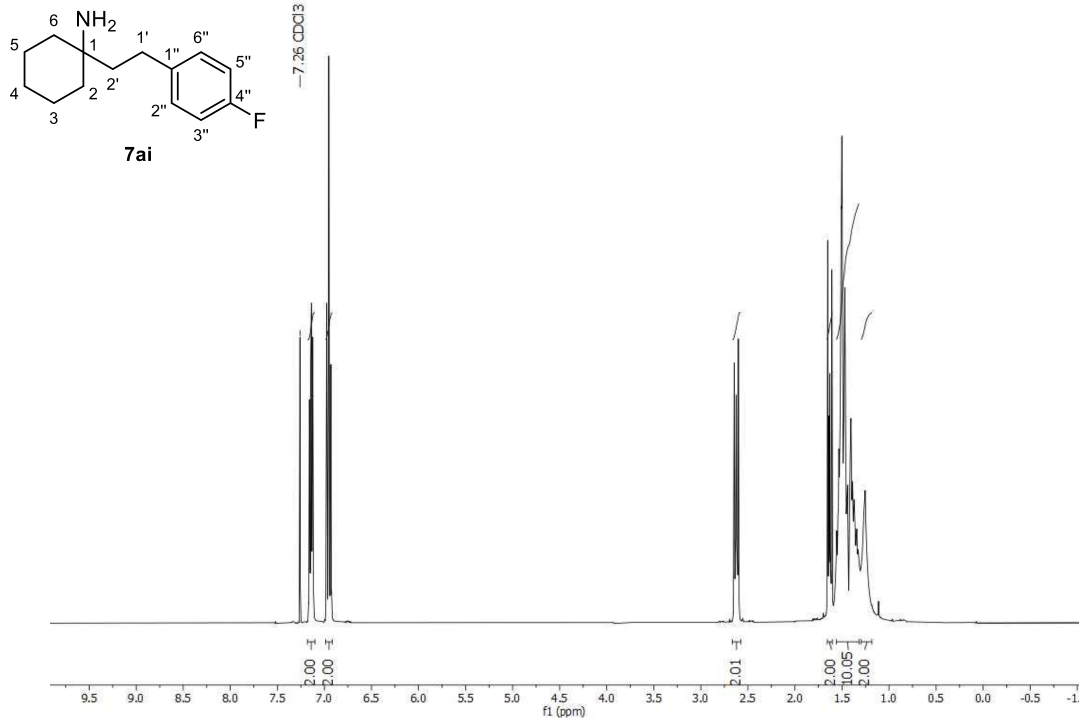

^{13}C{^{1}H} NMR (101 MHz, CDCl3)

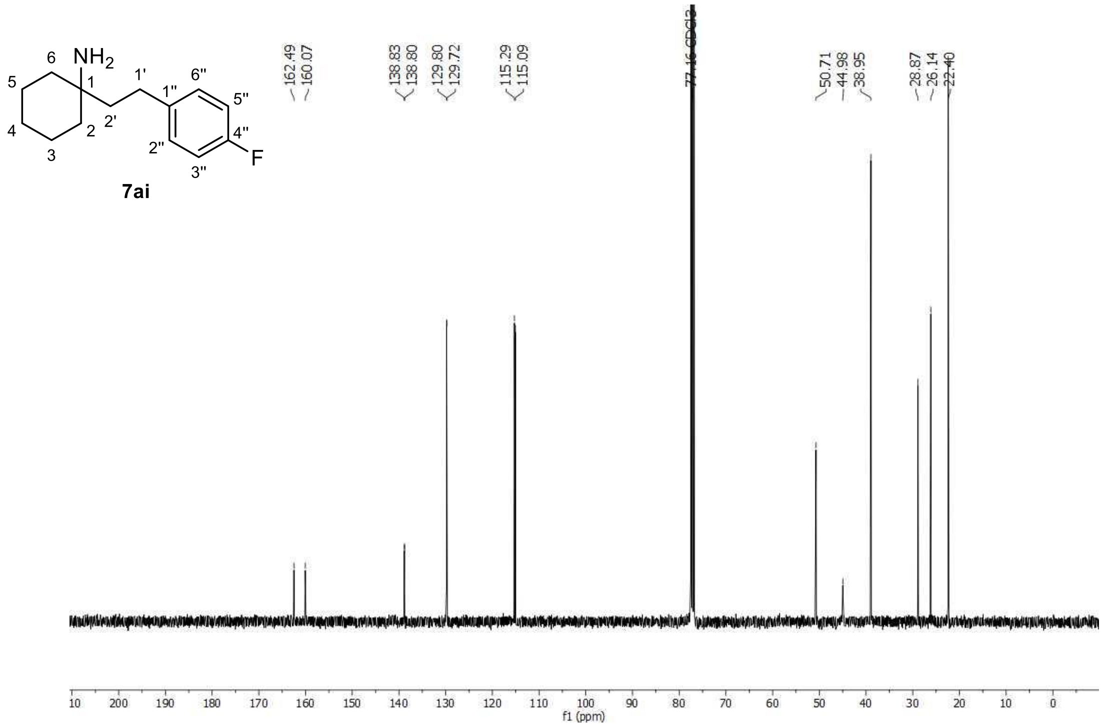

^{1}H NMR (400 MHz, CDCl3)

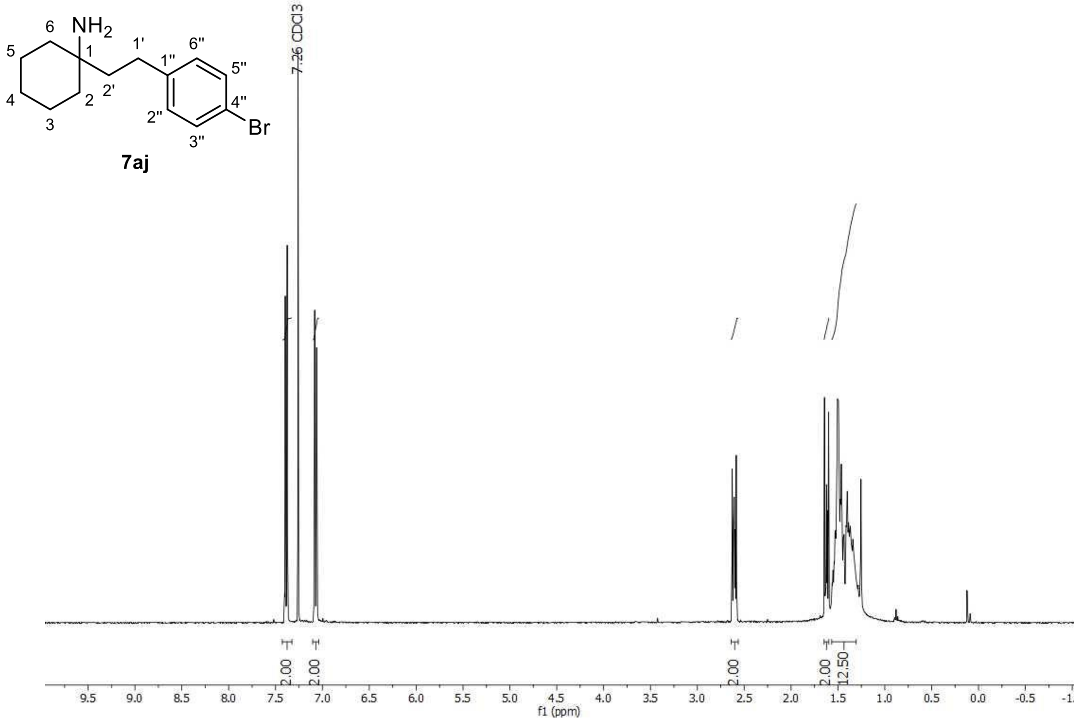

^{13}C{^{1}H} NMR (101 MHz, CDCl3)

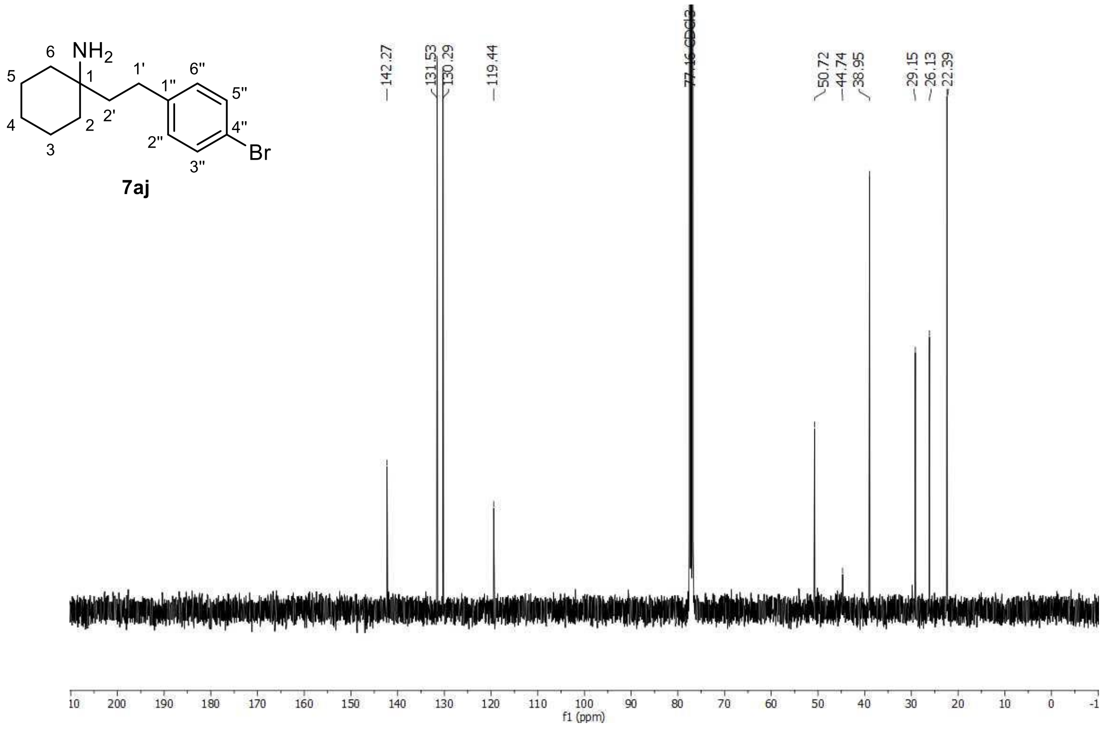

^{1}H NMR (400 MHz, MeOD)

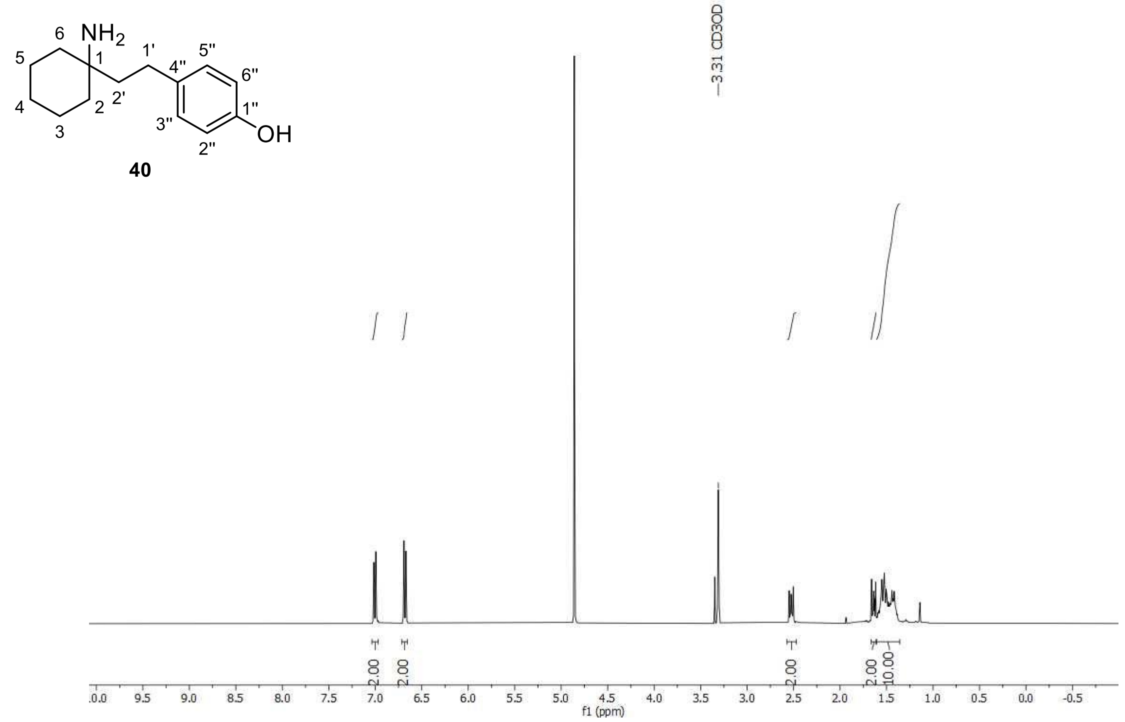

^{13}C{^{1}H} NMR (101 MHz, MeOD)

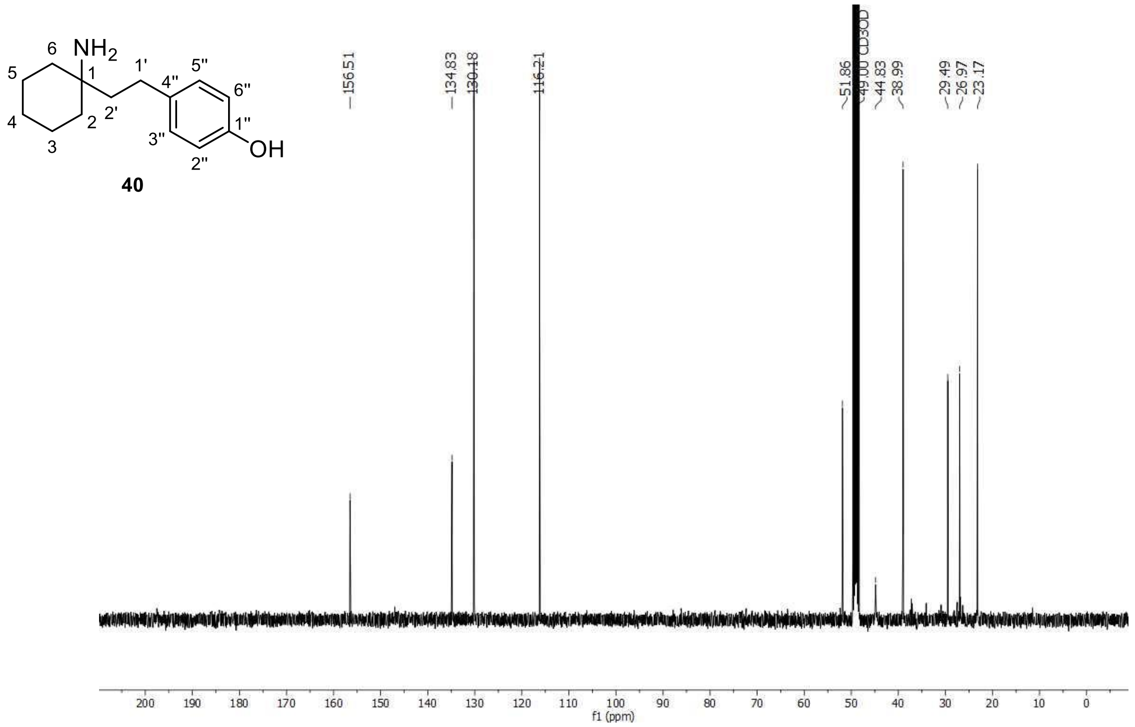

^{1}H NMR (400 MHz, CDCl$_3$)

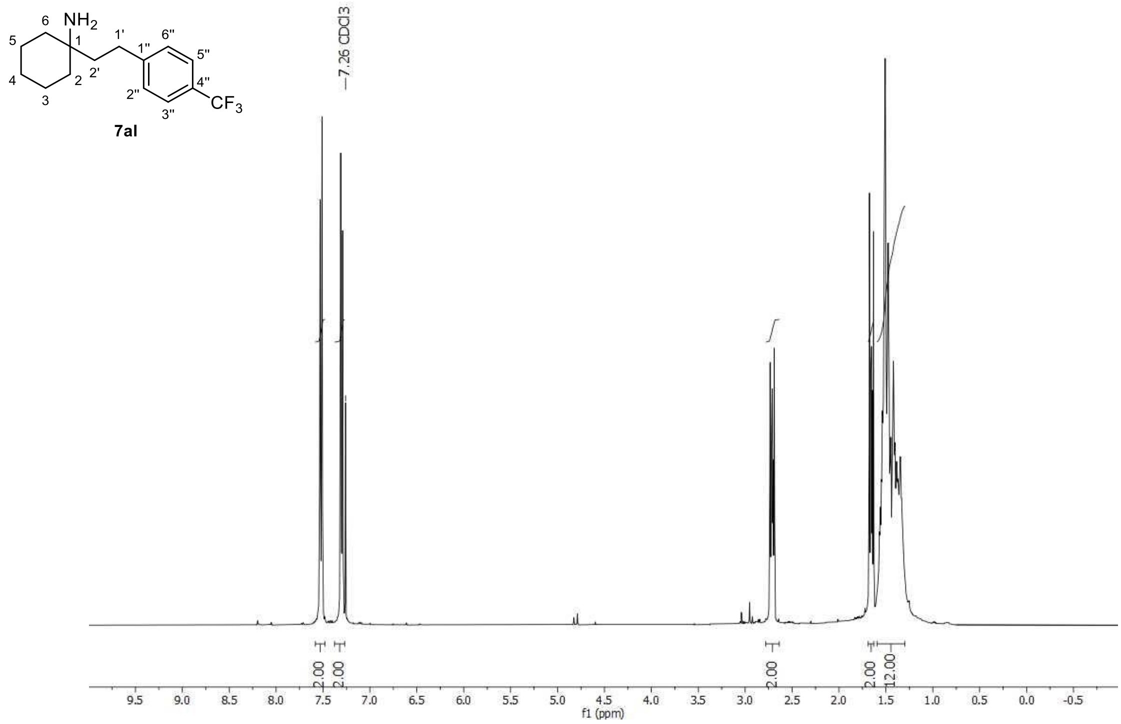

^{13}C{^{1}H} NMR (101 MHz, CDCl$_3$)

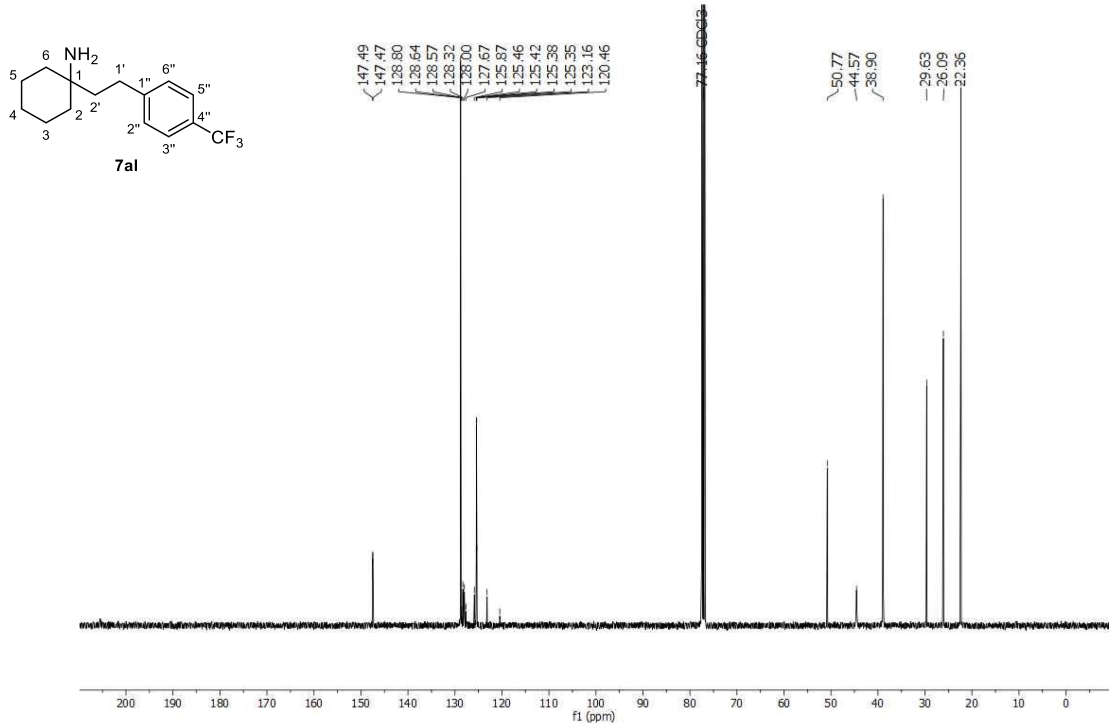

^{1}H NMR (400 MHz, CDCl$_3$)

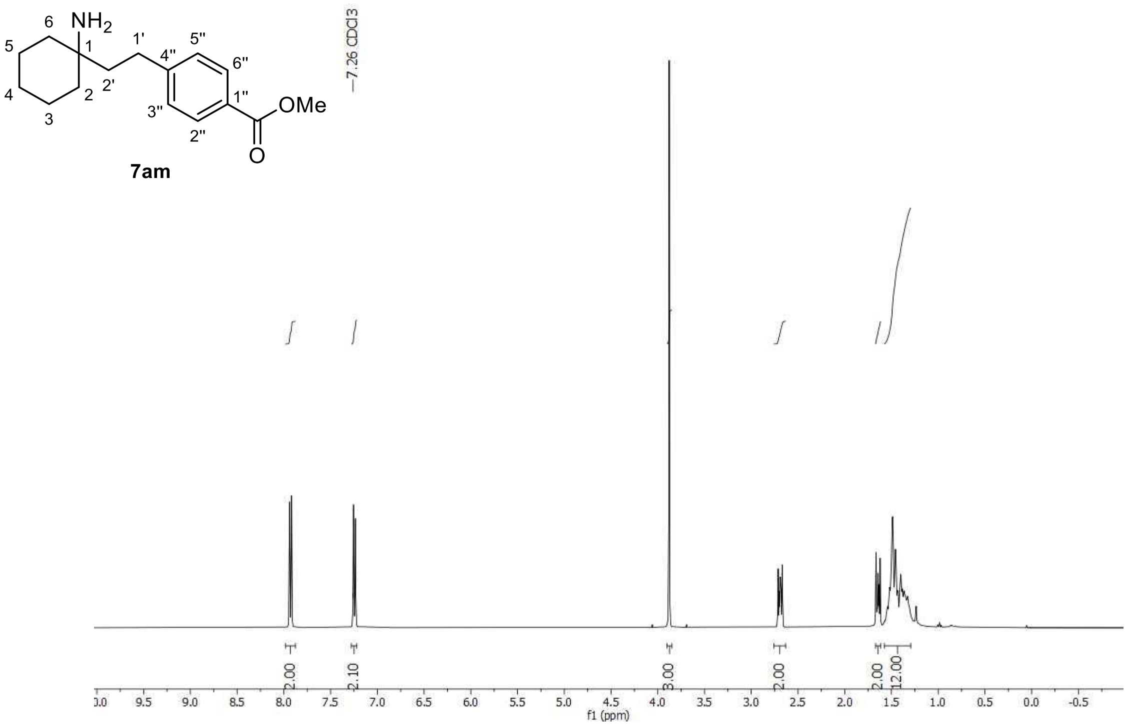

^{13}C{^{1}H} NMR (101 MHz, CDCl$_3$)

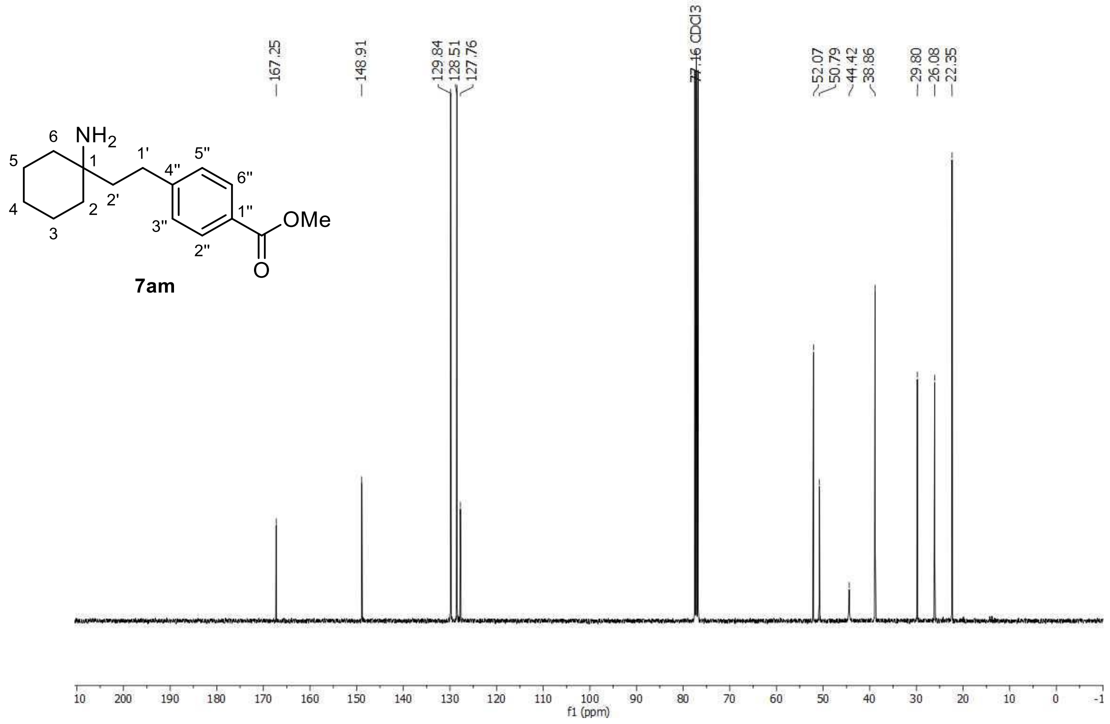

^{1}H NMR (400 MHz, $CDCl_3$)

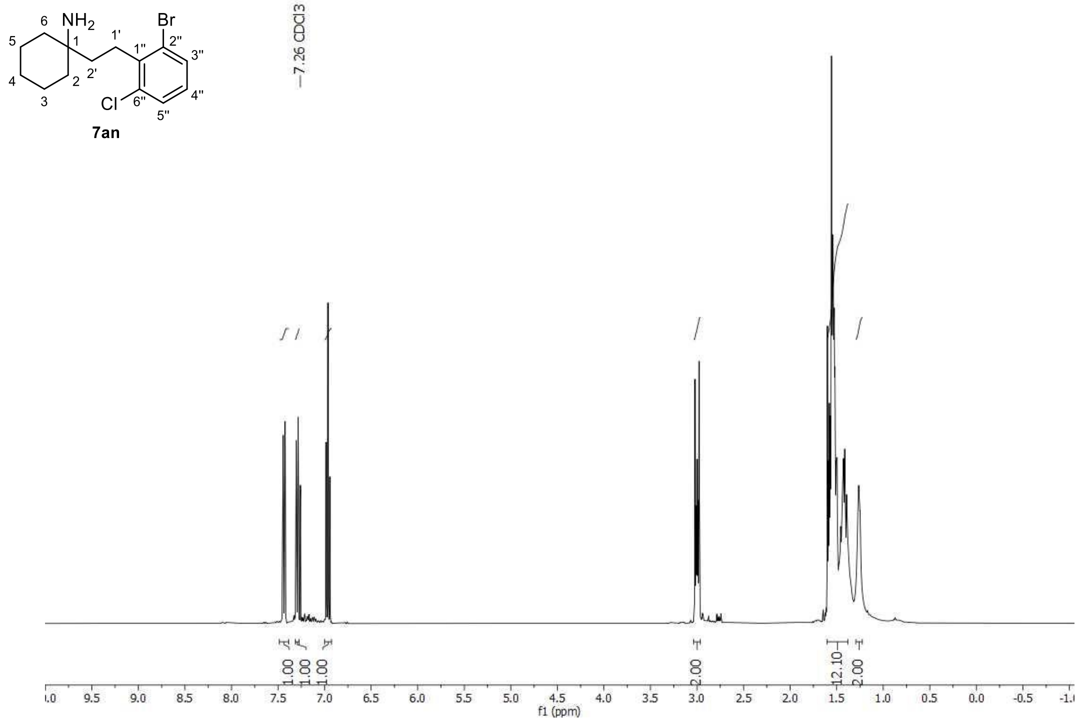

$^{13}C\{^1H\}$ NMR (101 MHz, $CDCl_3$)

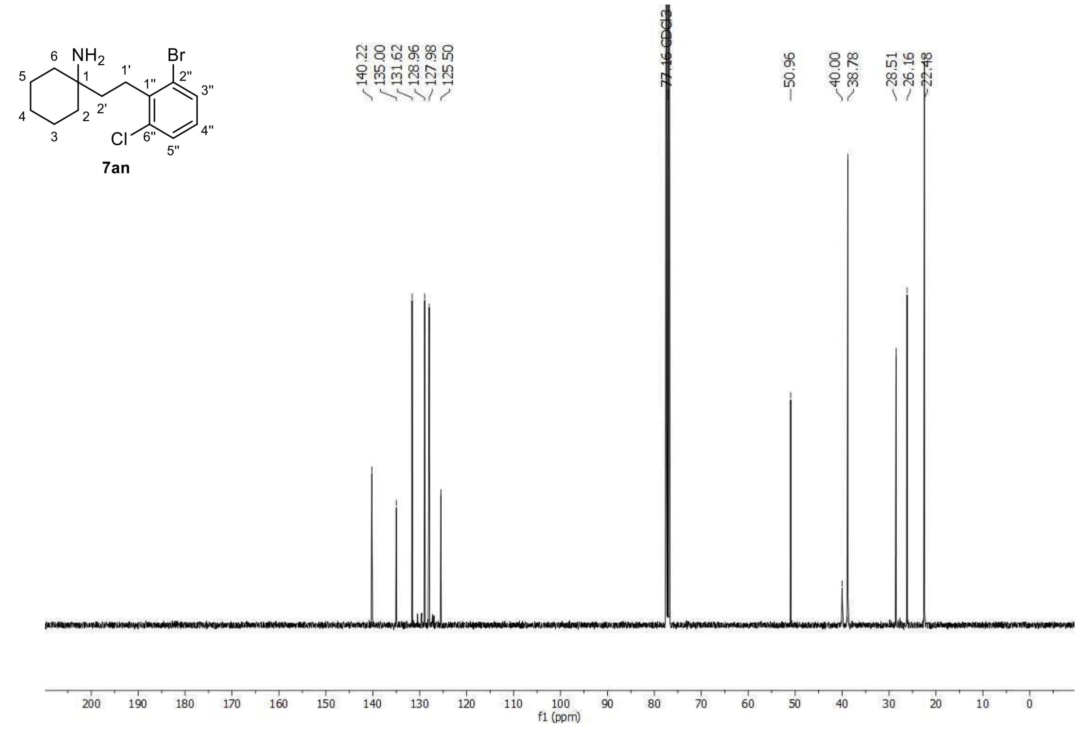

^{1}H NMR (400 MHz, CDCl$_3$)

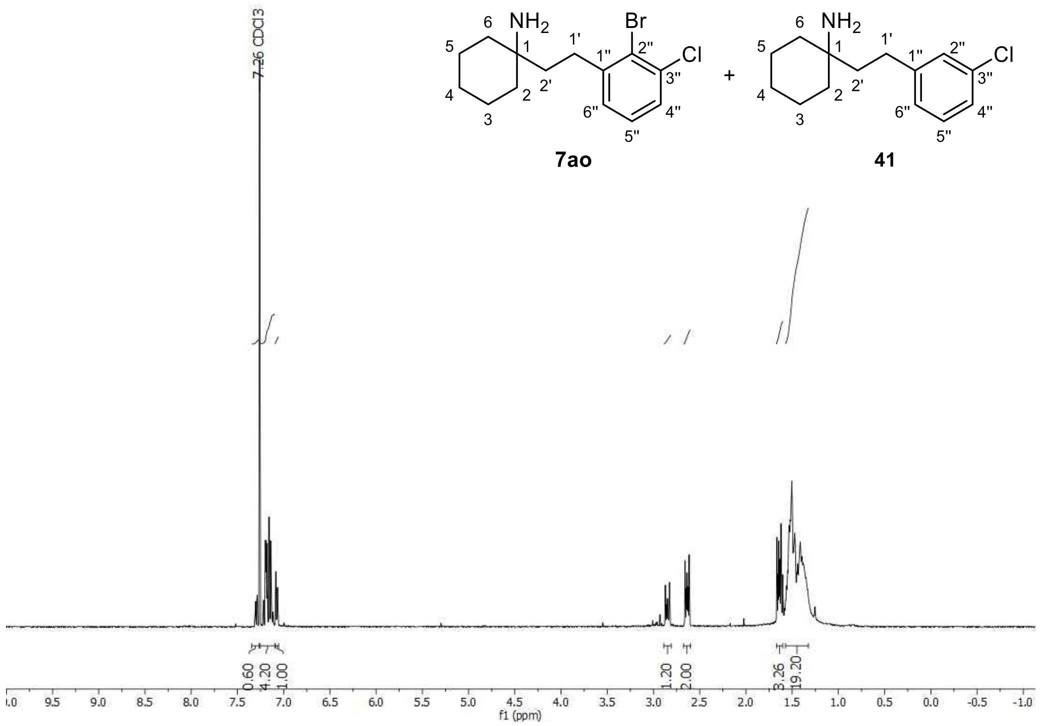

^{13}C{^{1}H} NMR (101 MHz, CDCl$_3$)

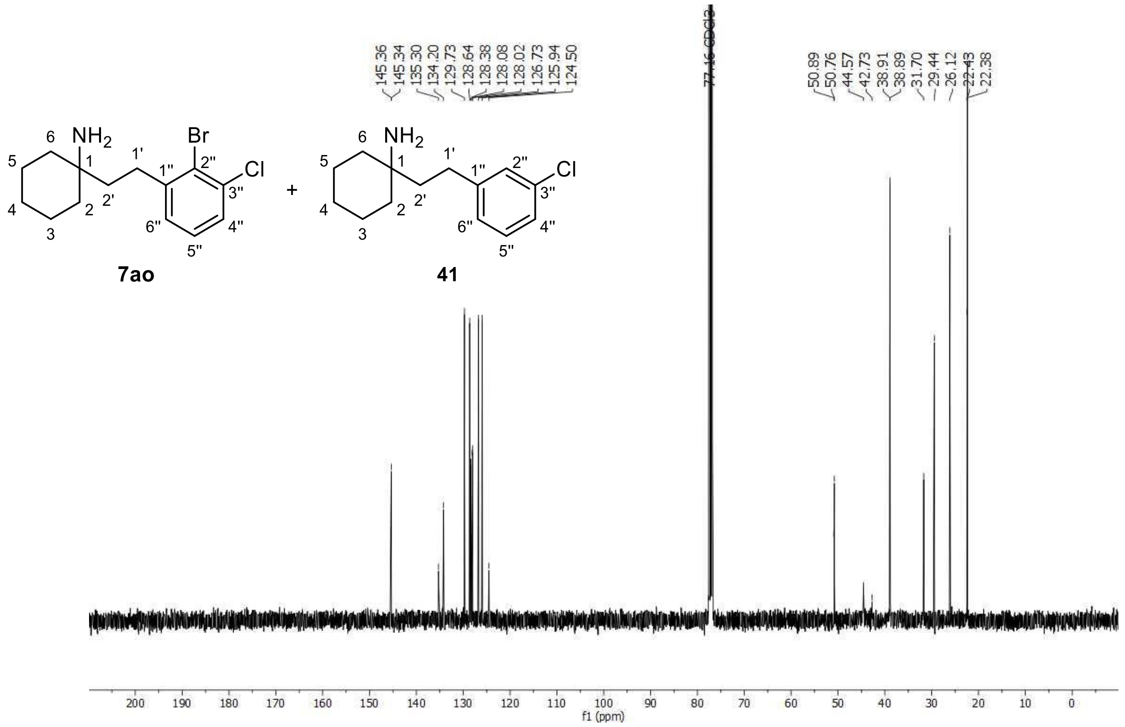

^{1}H NMR (400 MHz, CDCl$_3$)

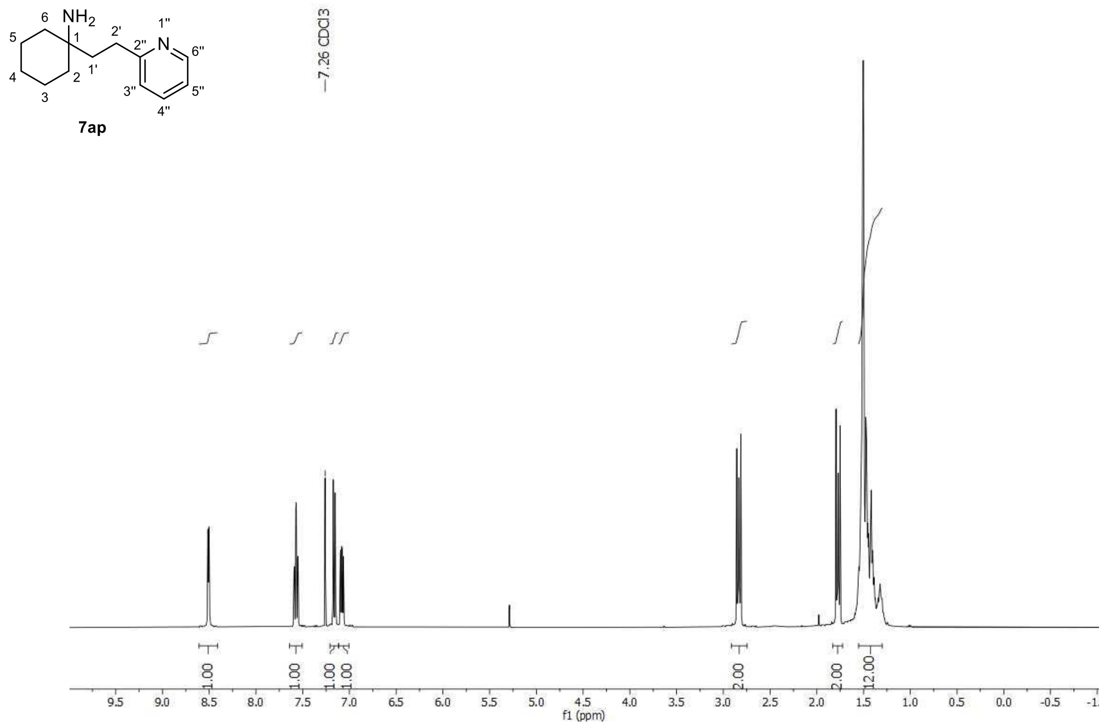

^{13}C{^{1}H} NMR (101 MHz, CDCl$_3$)

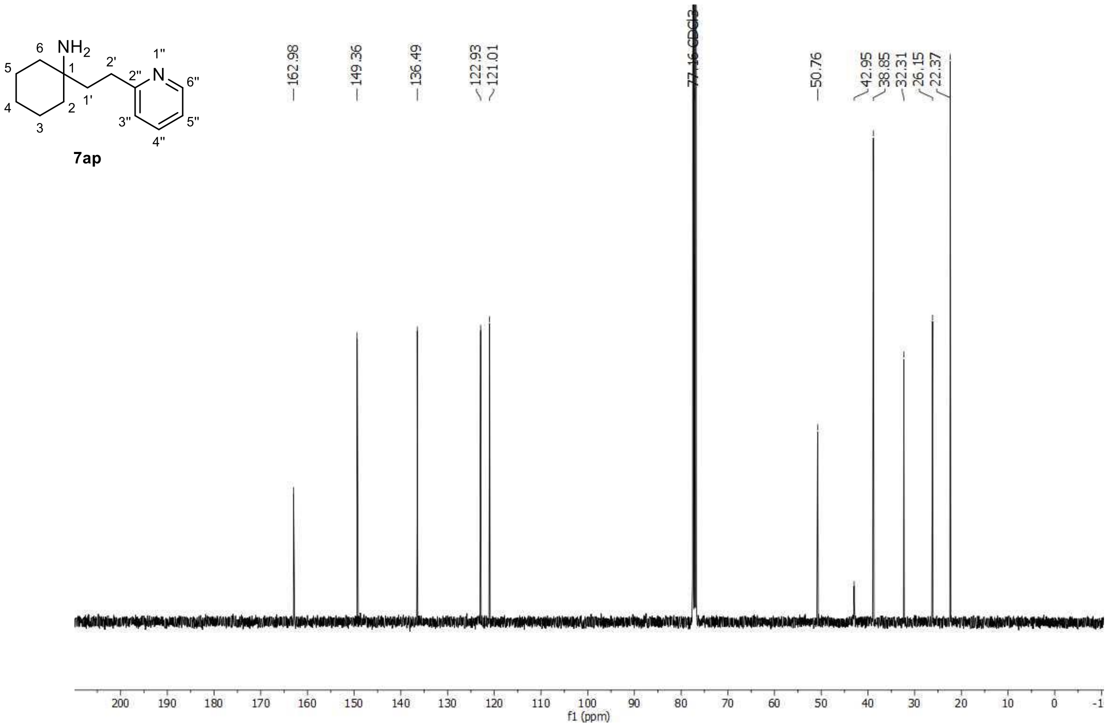

^{1}H NMR (400 MHz, CDCl$_3$)

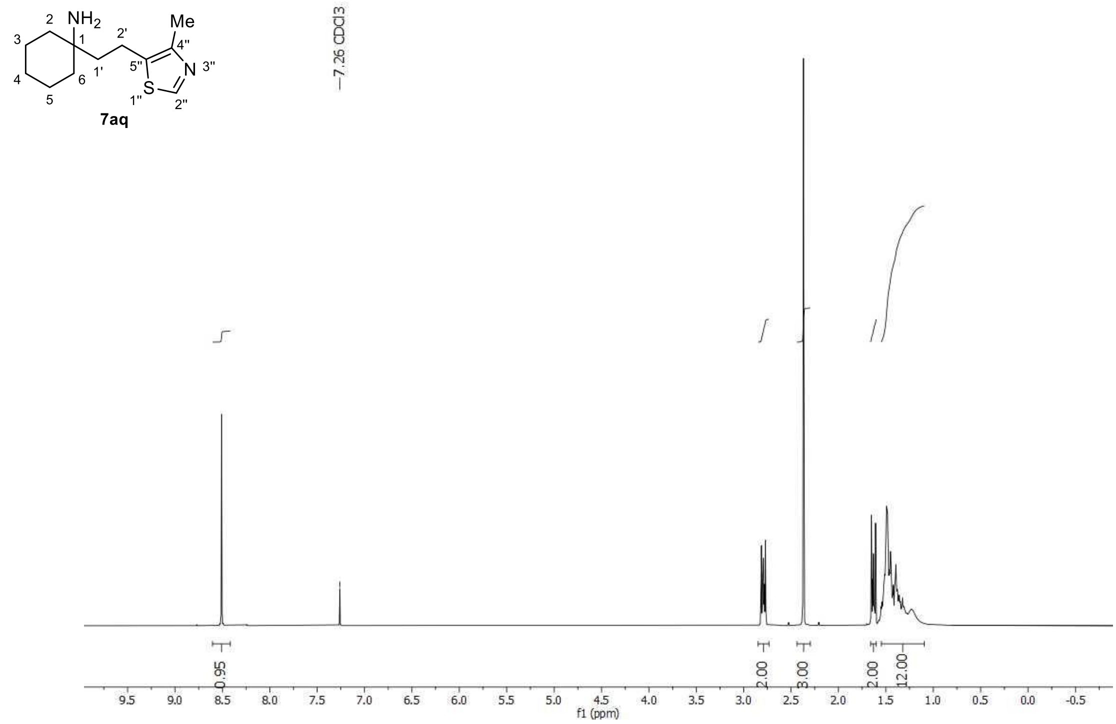

^{13}C{^{1}H} NMR (101 MHz, CDCl$_3$)

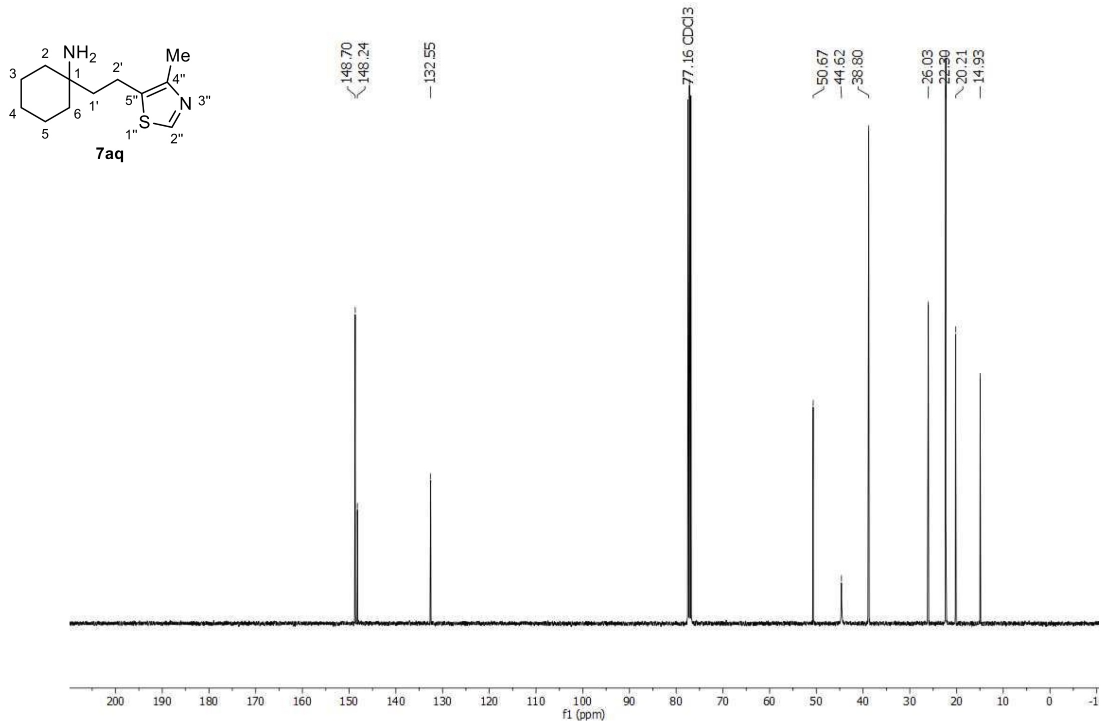

^{1}H NMR (400 MHz, CDCl$_3$)

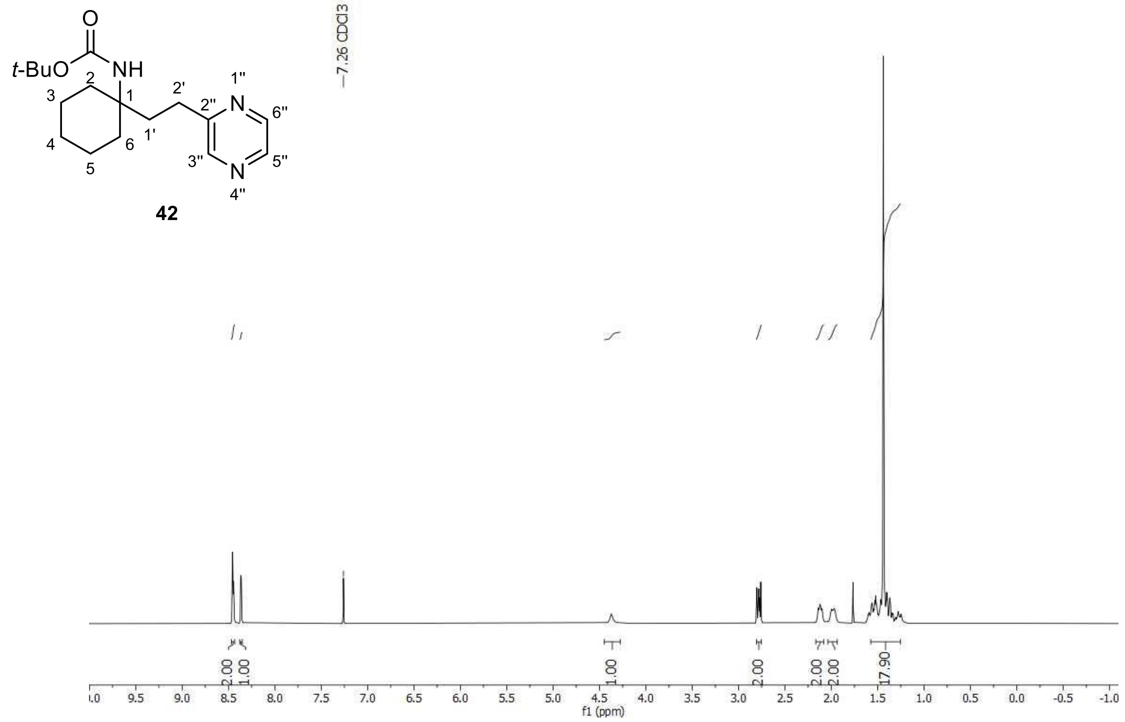

^{13}C{^{1}H} NMR (101 MHz, CDCl$_3$)

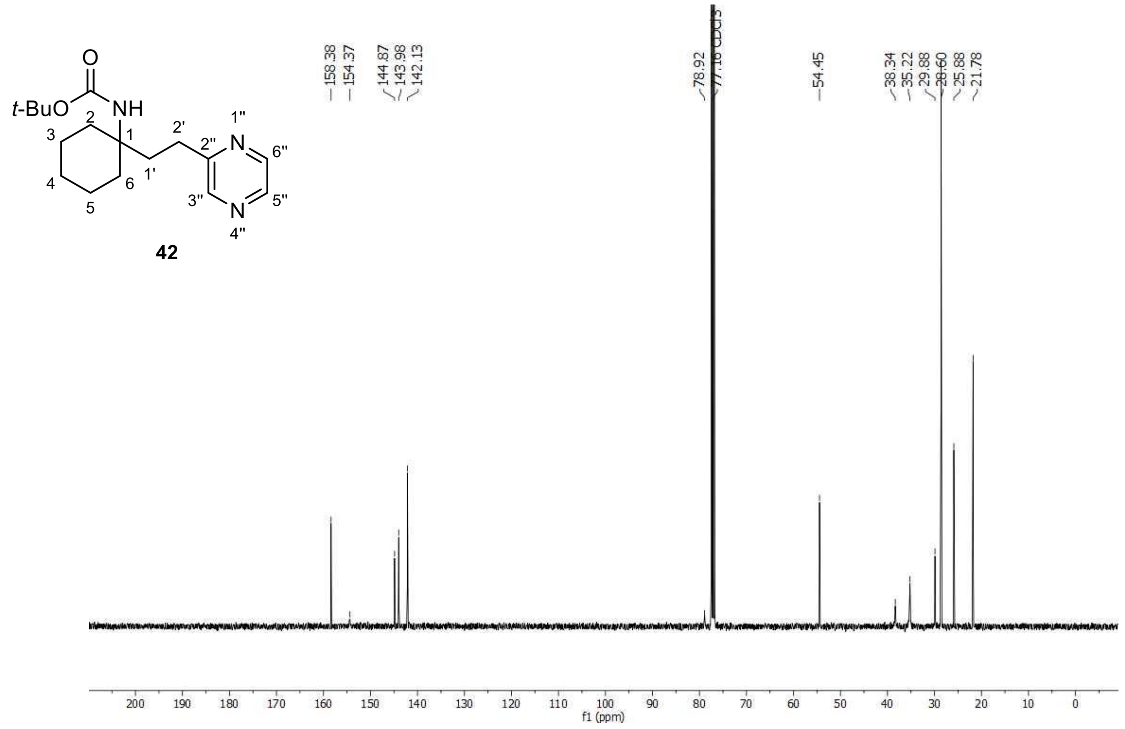

^{1}H NMR (400 MHz, $CDCl_3$)

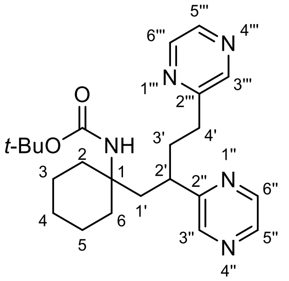

43

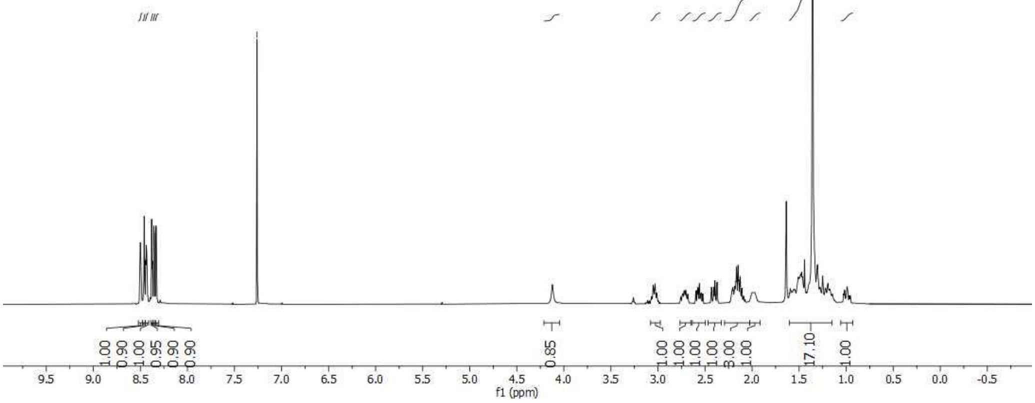

^{13}C{^{1}H} NMR (101 MHz, $CDCl_3$)

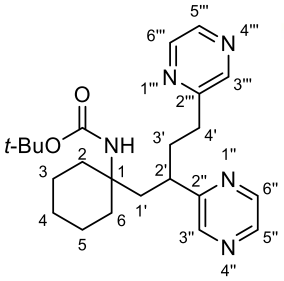

43

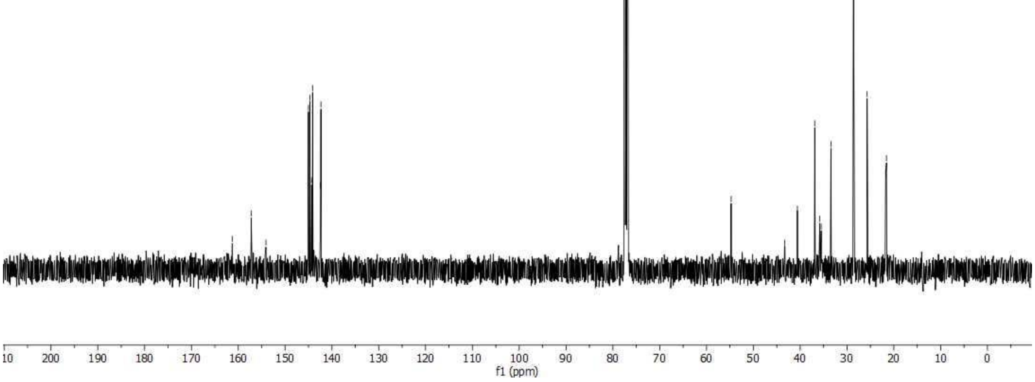

^{1}H NMR (400 MHz, $CDCl_3$)

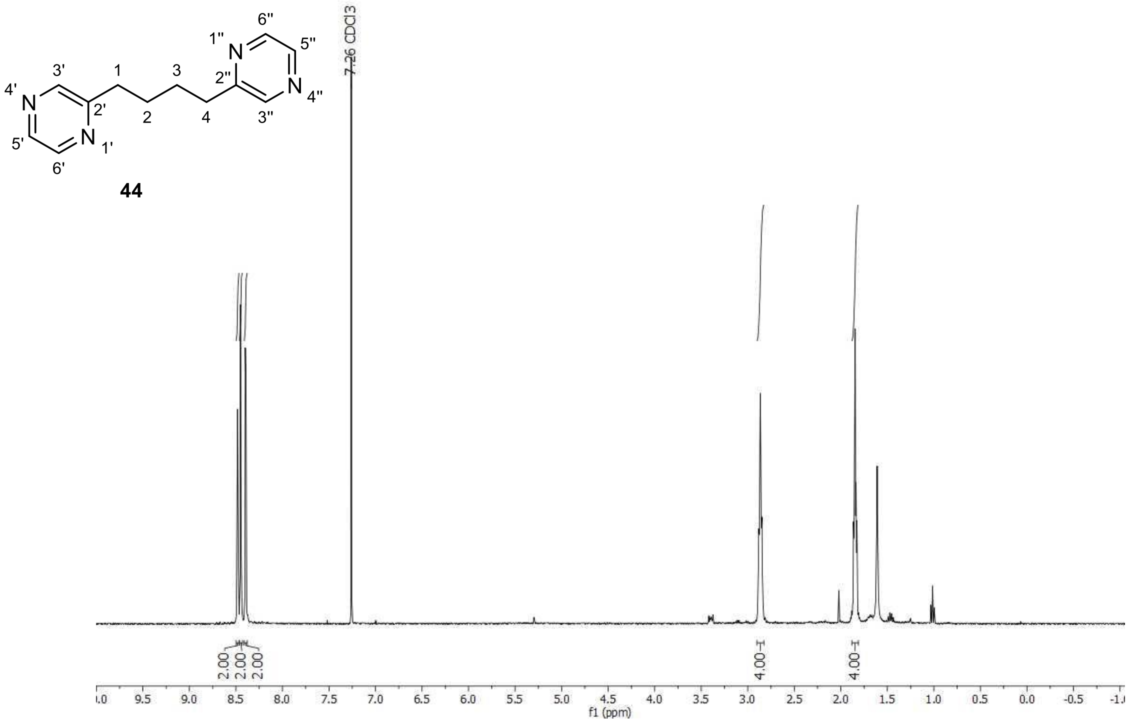

^{13}C{^{1}H} NMR (101 MHz, $CDCl_3$)

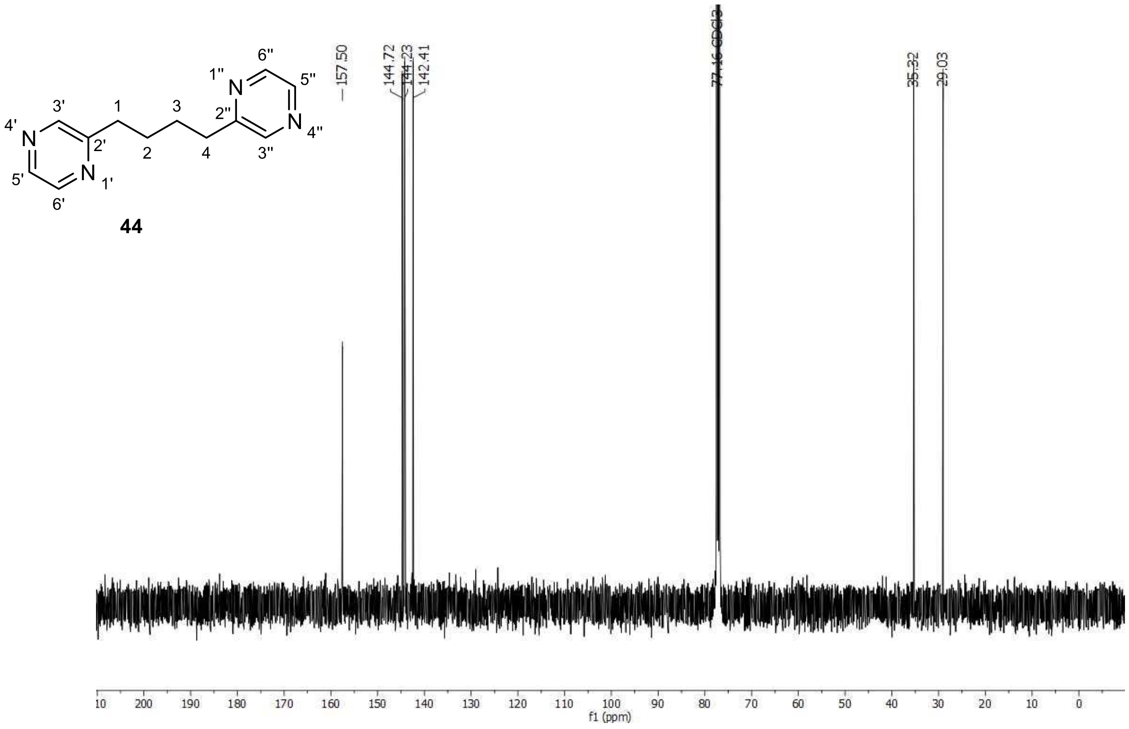

^{1}H NMR (400 MHz, $CDCl_3$)

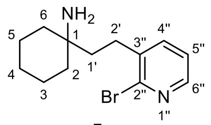

7as

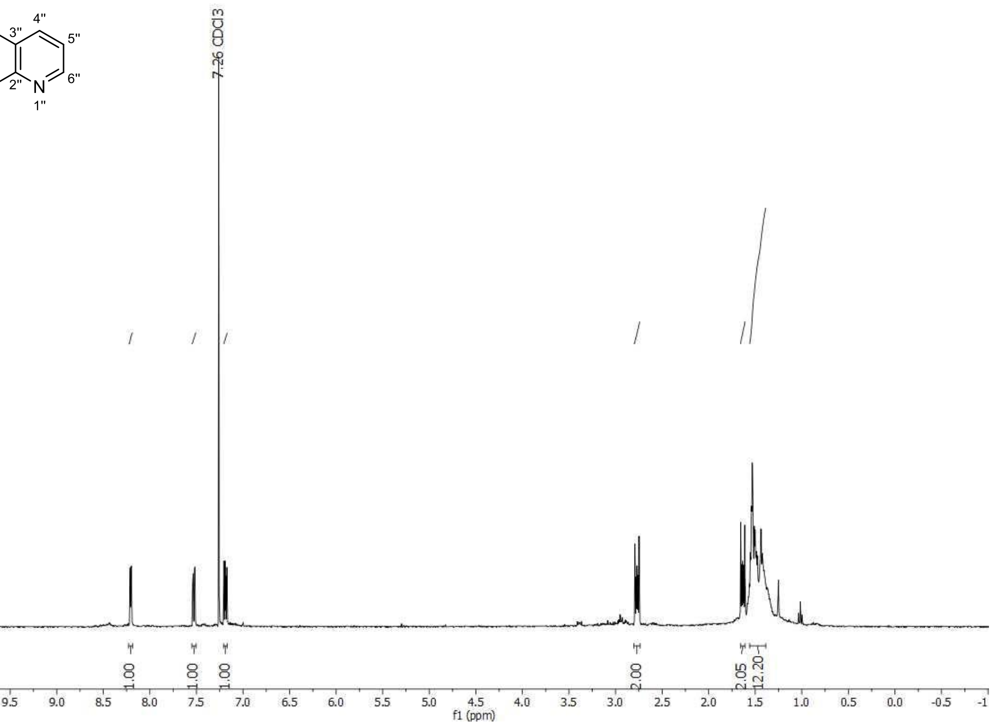

^{13}C{^{1}H} NMR (101 MHz, $CDCl_3$)

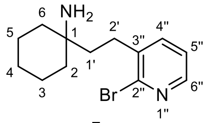

7as

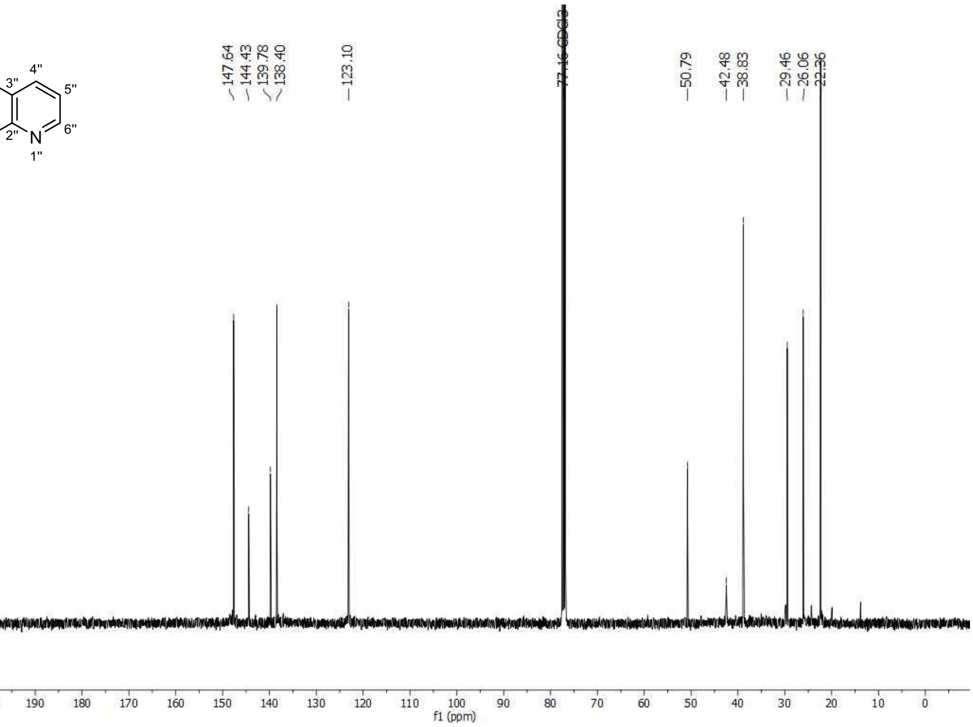

^{1}H NMR (400 MHz, CDCl3)

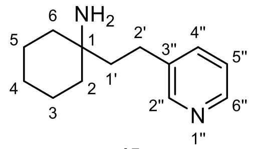

45

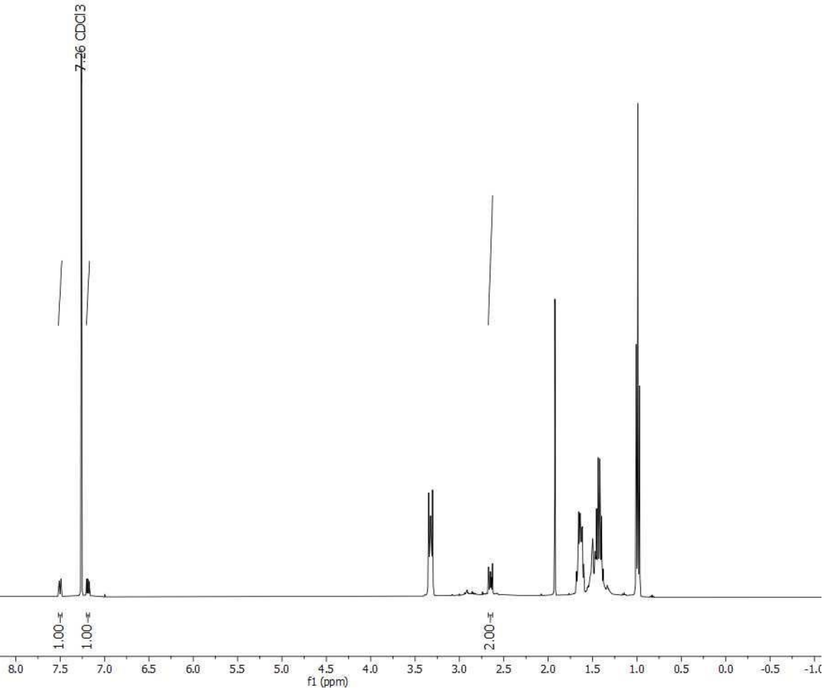

^{13}C{^{1}H} NMR (126 MHz, CDCl3)

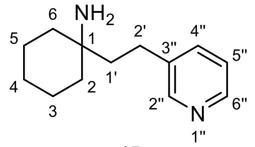

45

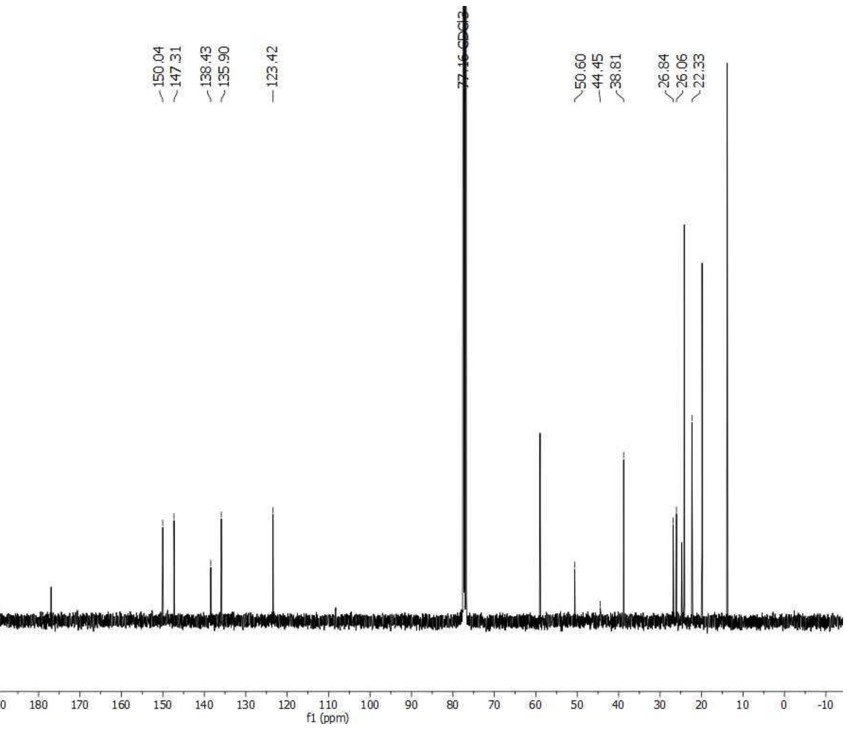

^{1}H NMR (400 MHz, CDCl3)

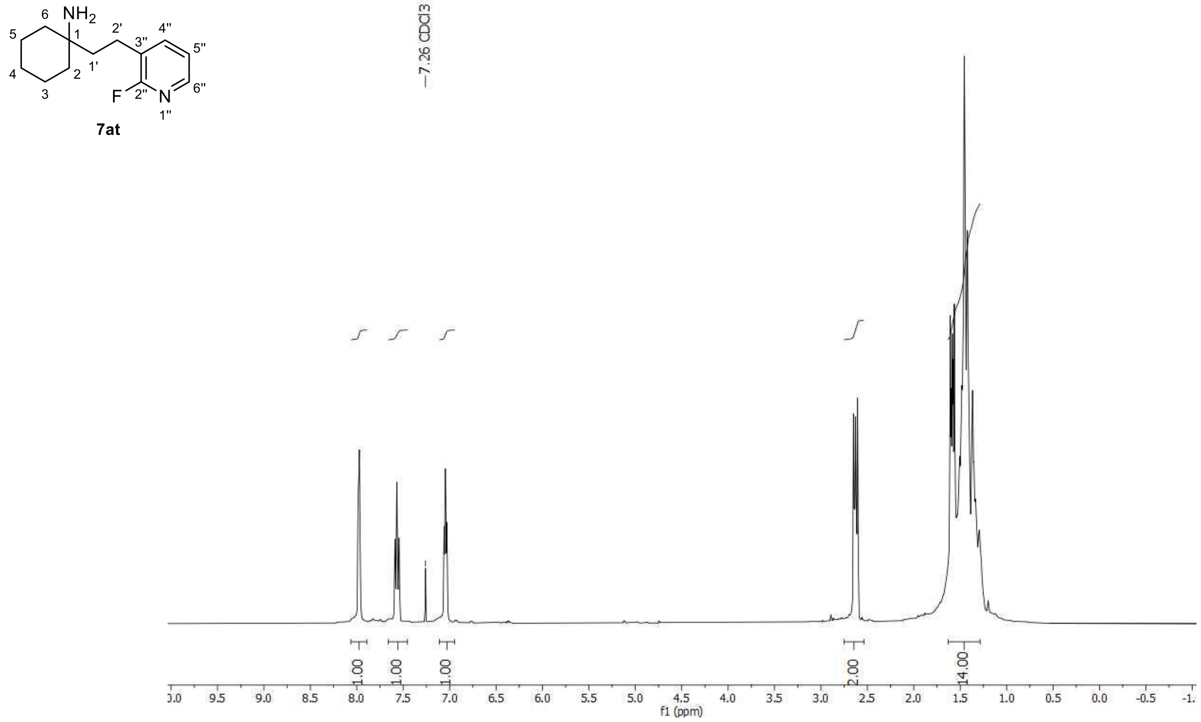

^{13}C{^{1}H} NMR (101 MHz, CDCl3)

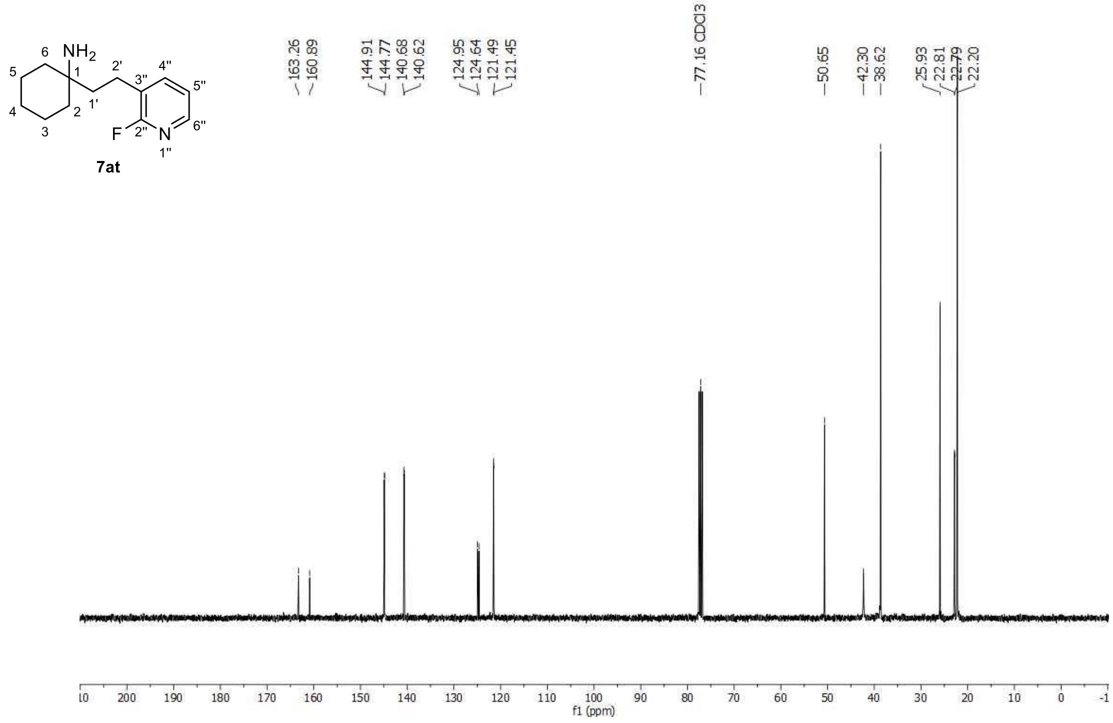

^{1}H NMR (400 MHz, MeOD)

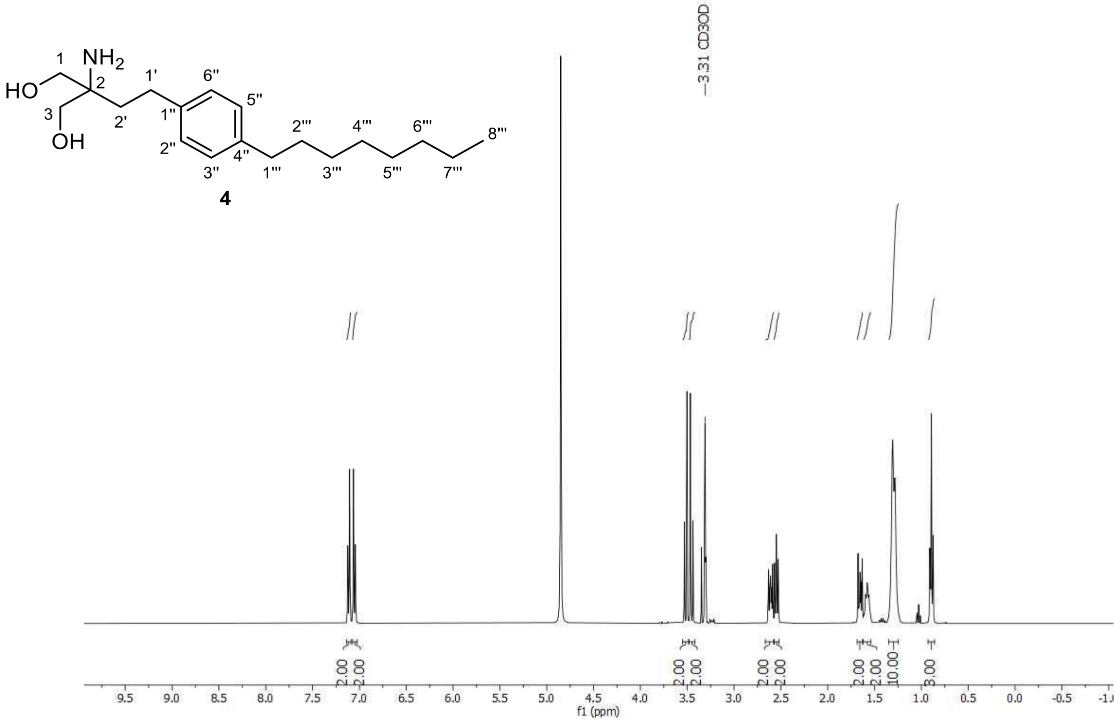

$^{13}C\{^{1}H\}$ NMR (101 MHz, MeOD)

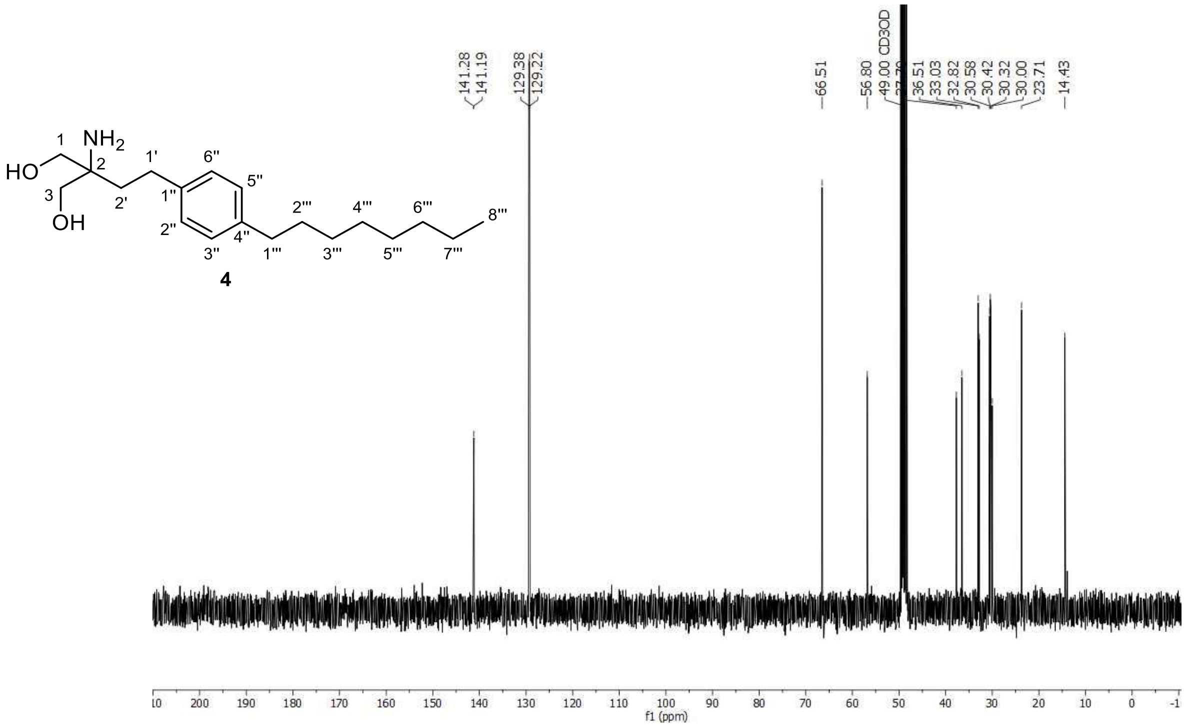

^{1}H NMR (400 MHz, $CDCl_3$)

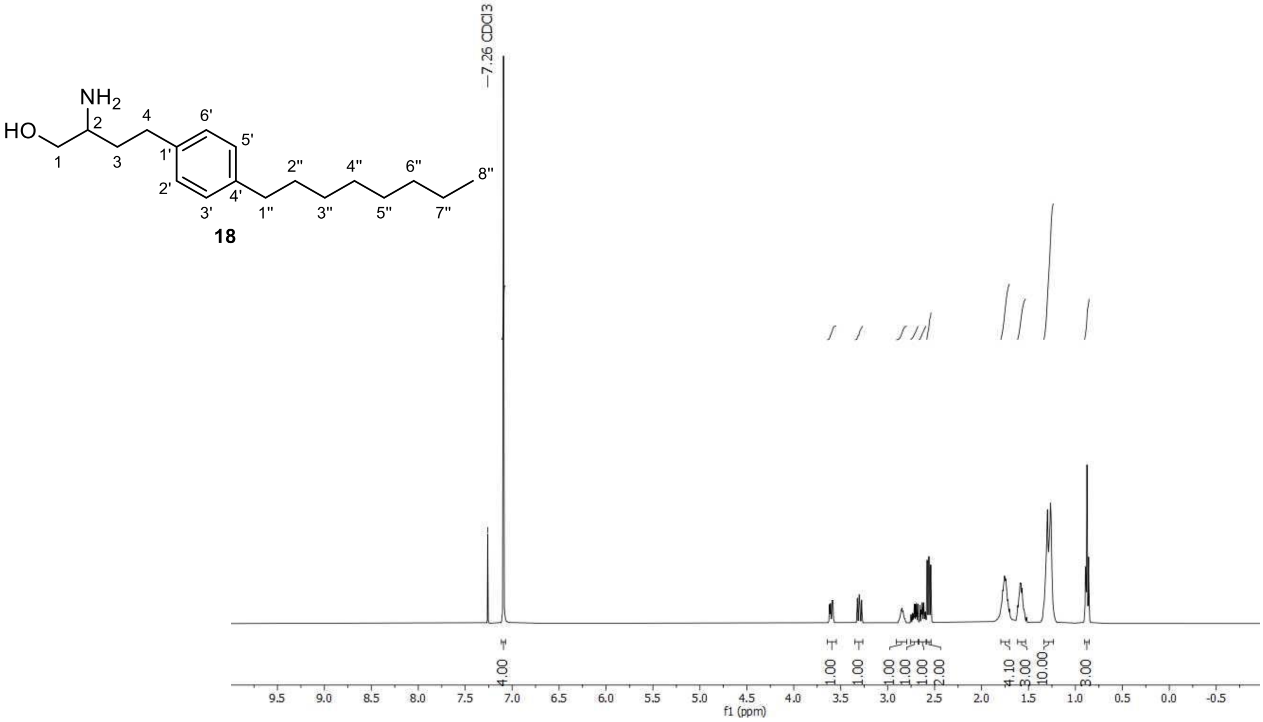

^{13}C{^{1}H} NMR (101 MHz, $CDCl_3$)

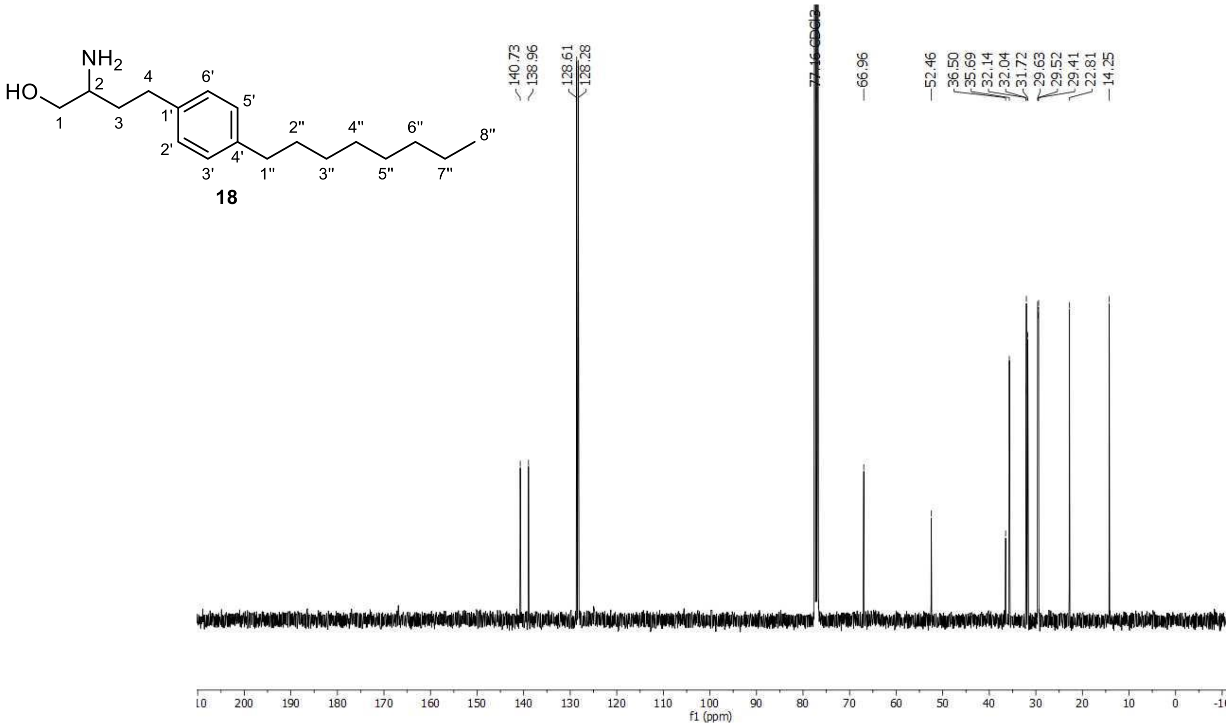

^{1}H NMR (400 MHz, CDCl$_3$)

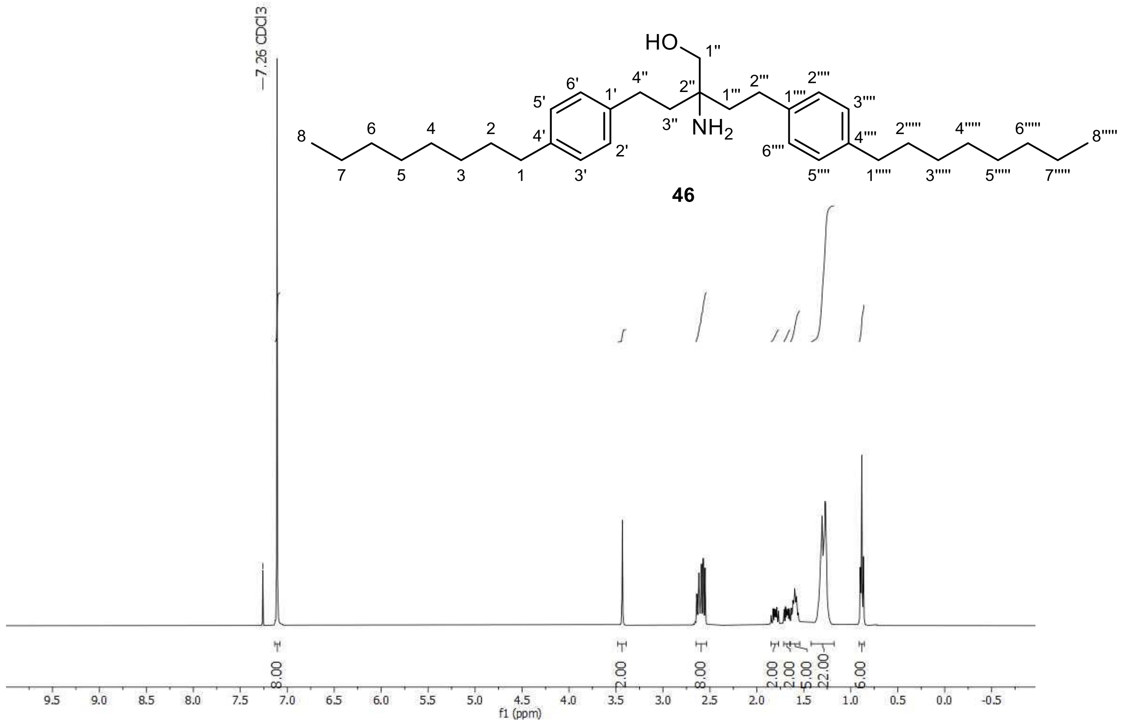

^{13}C{^{1}H} NMR (101 MHz, CDCl$_3$)

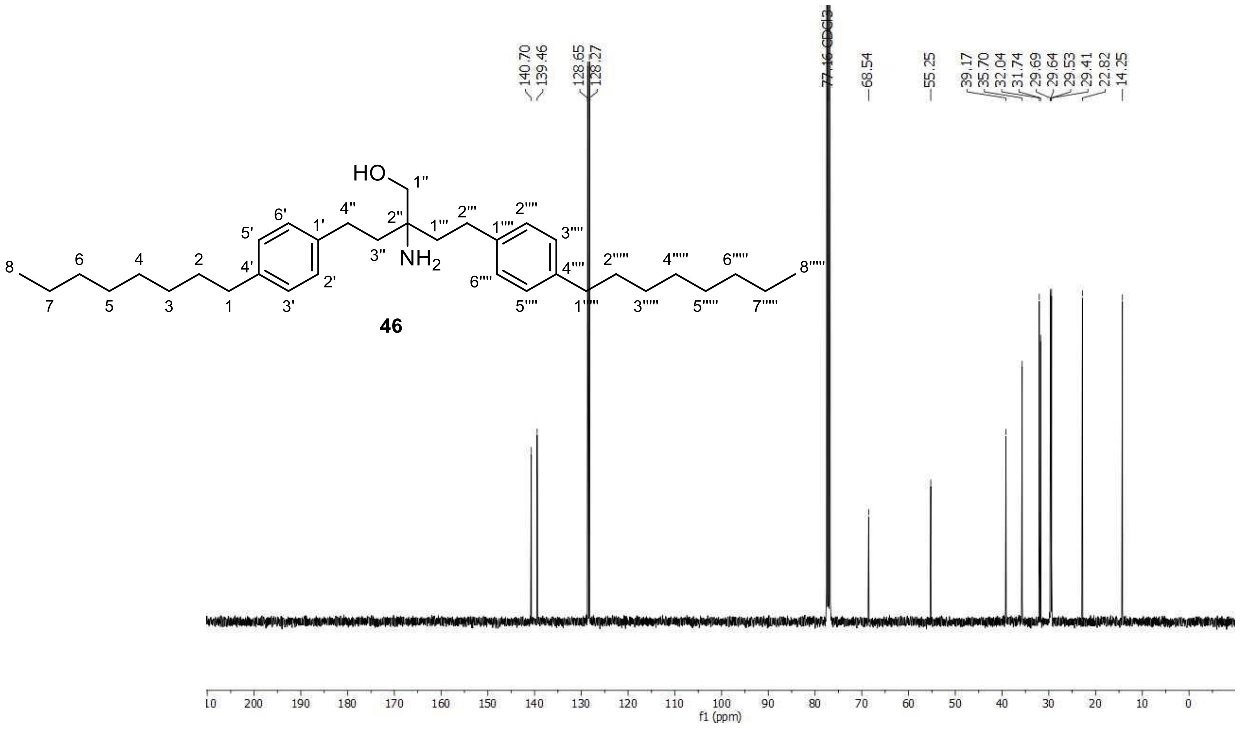

^{1}H NMR (400 MHz, $CDCl_3$)

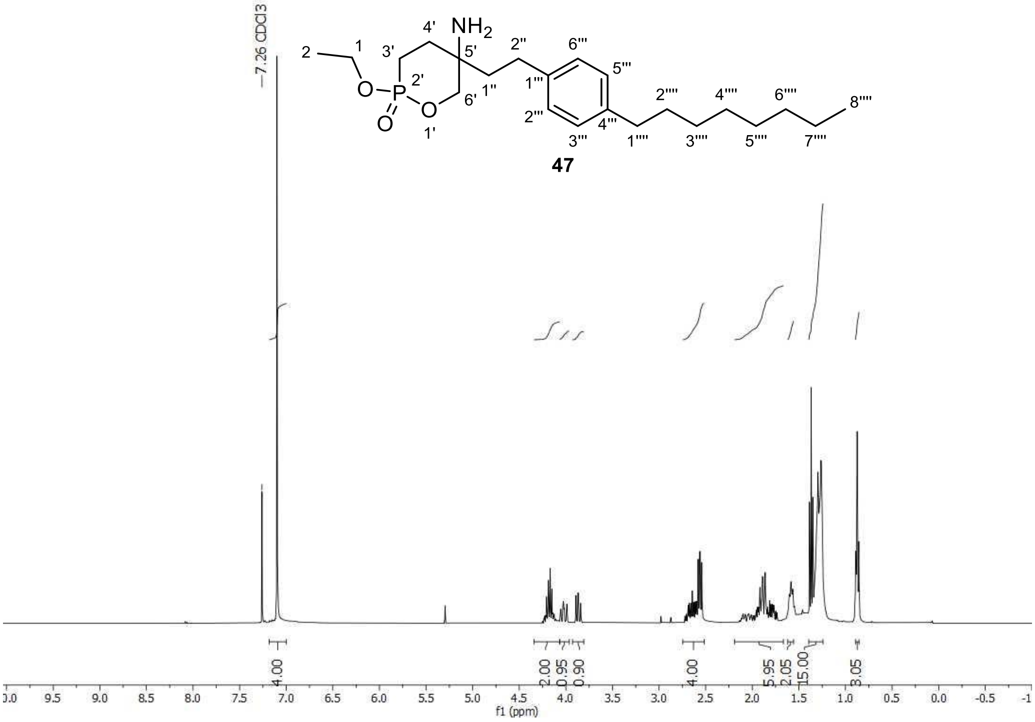

^{13}C{^{1}H} NMR (101 MHz, $CDCl_3$)

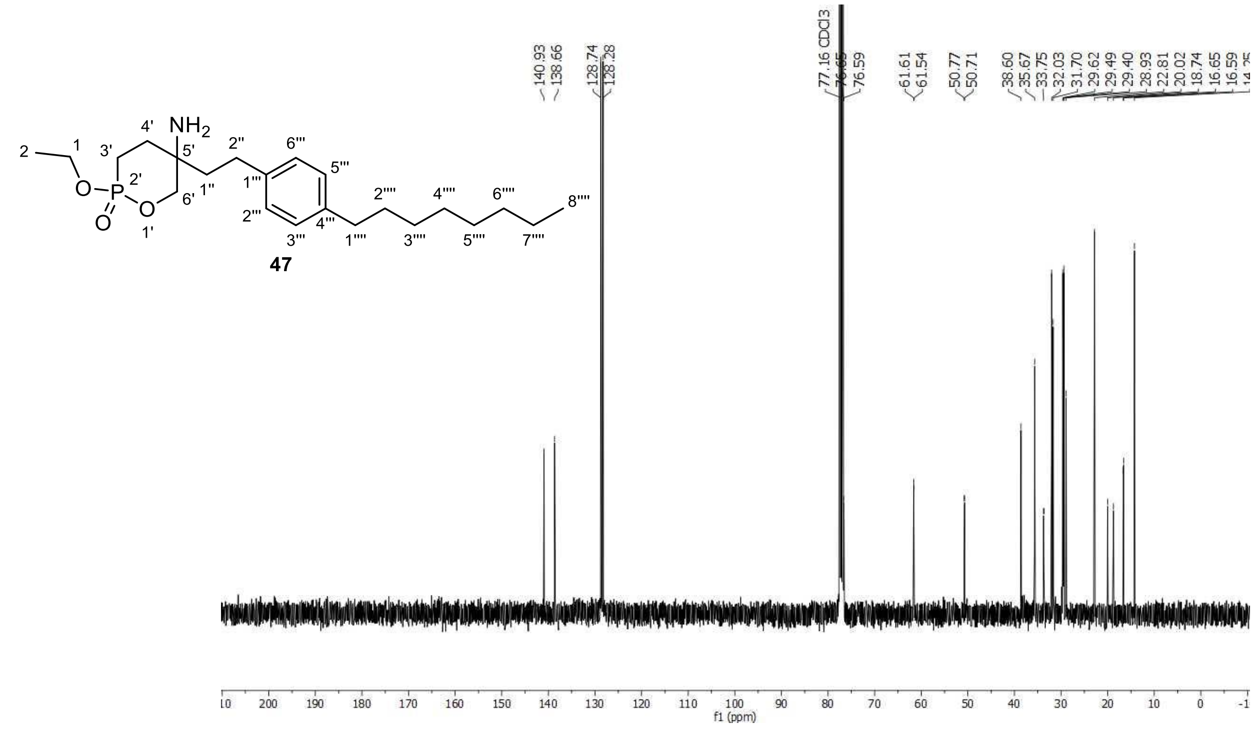

^{1}H NMR (400 MHz, CDCl3)

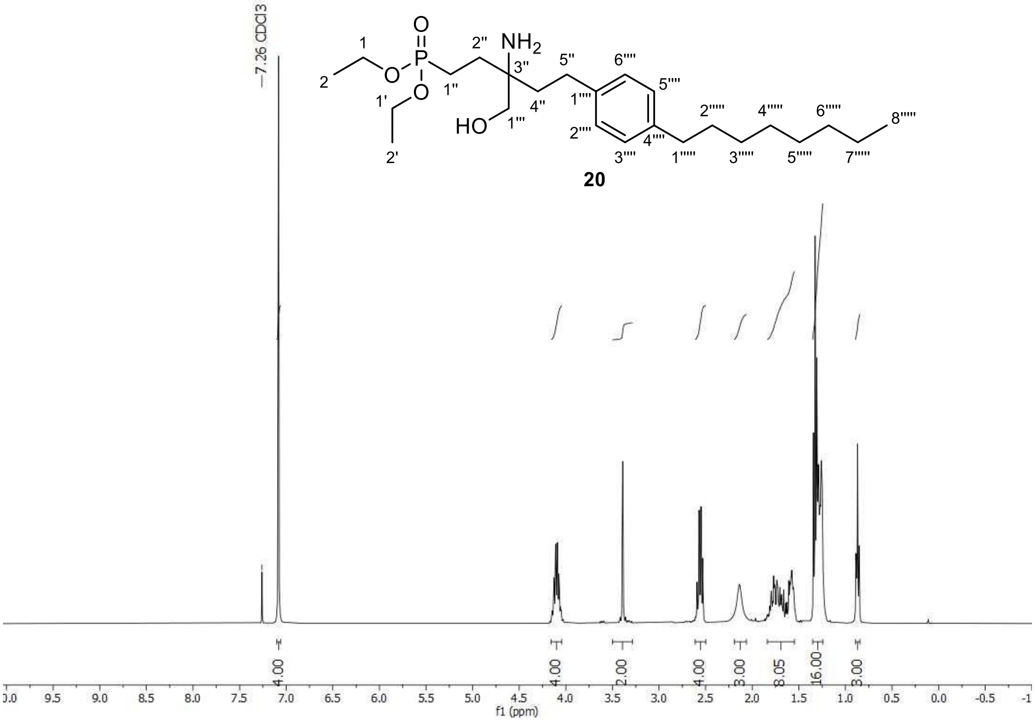

^{13}C{^{1}H} NMR (101 MHz, CDCl3)

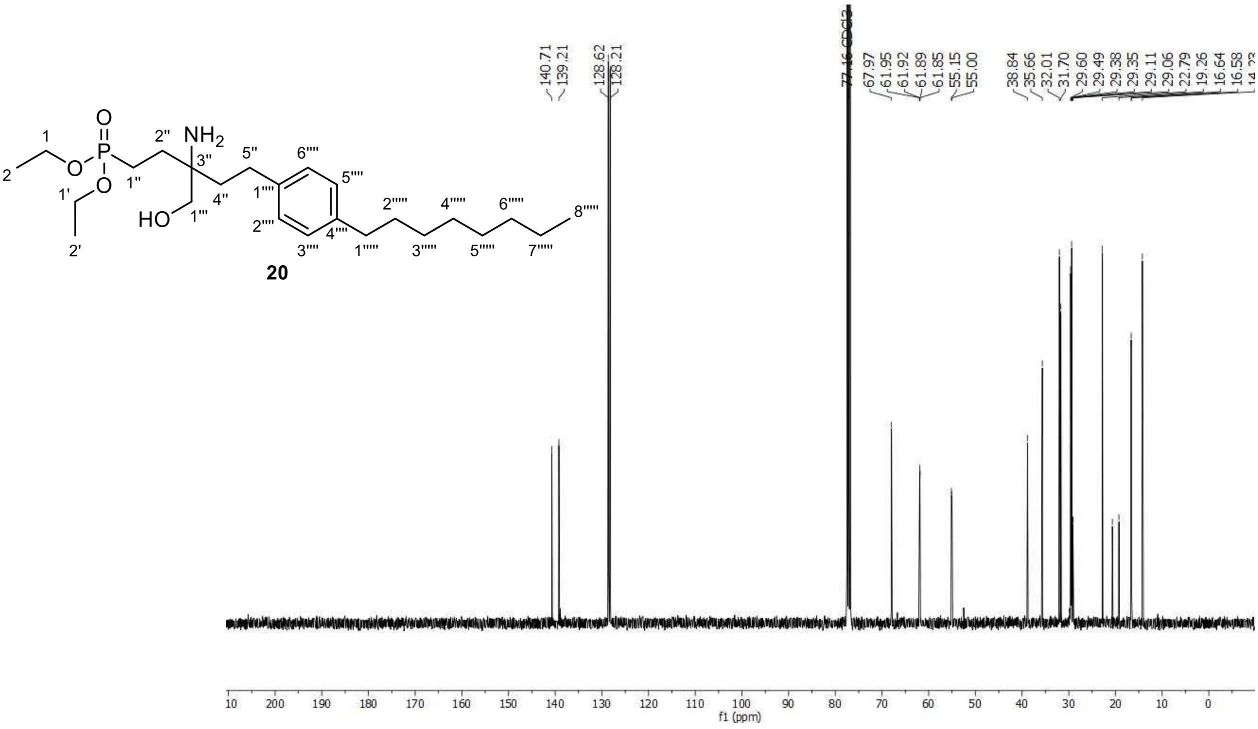

^{1}H NMR (400 MHz, CDCl₃)

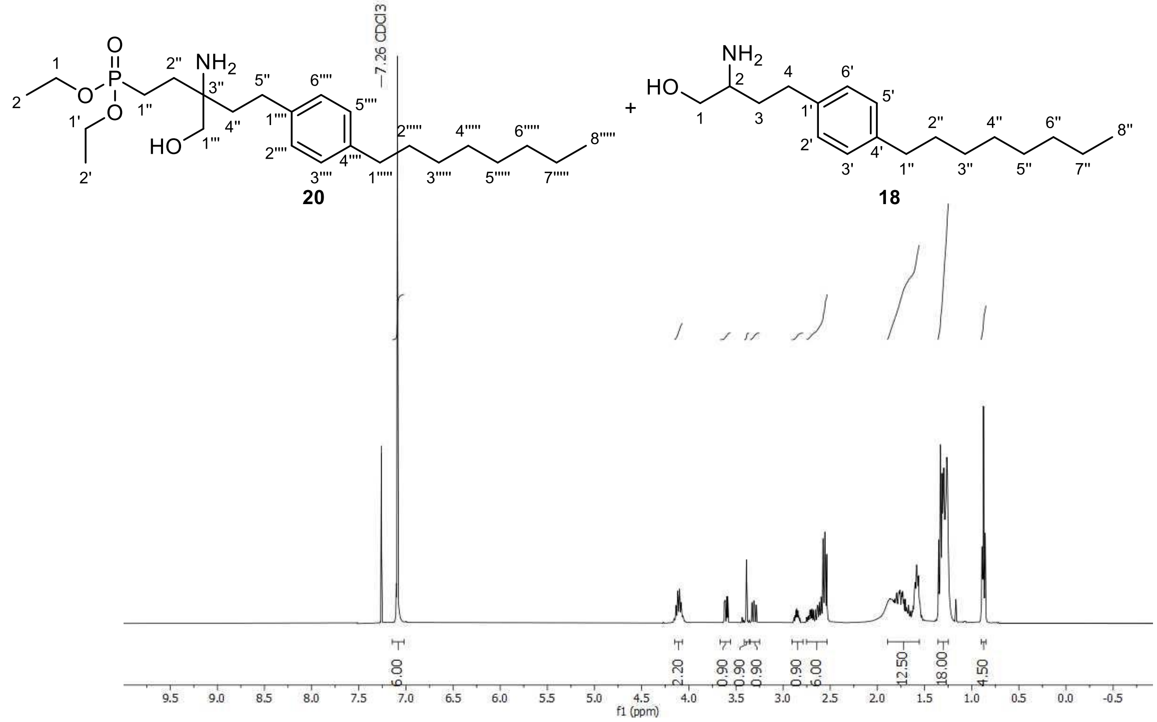

^{13}C{^{1}H} NMR (101 MHz, CDCl₃)

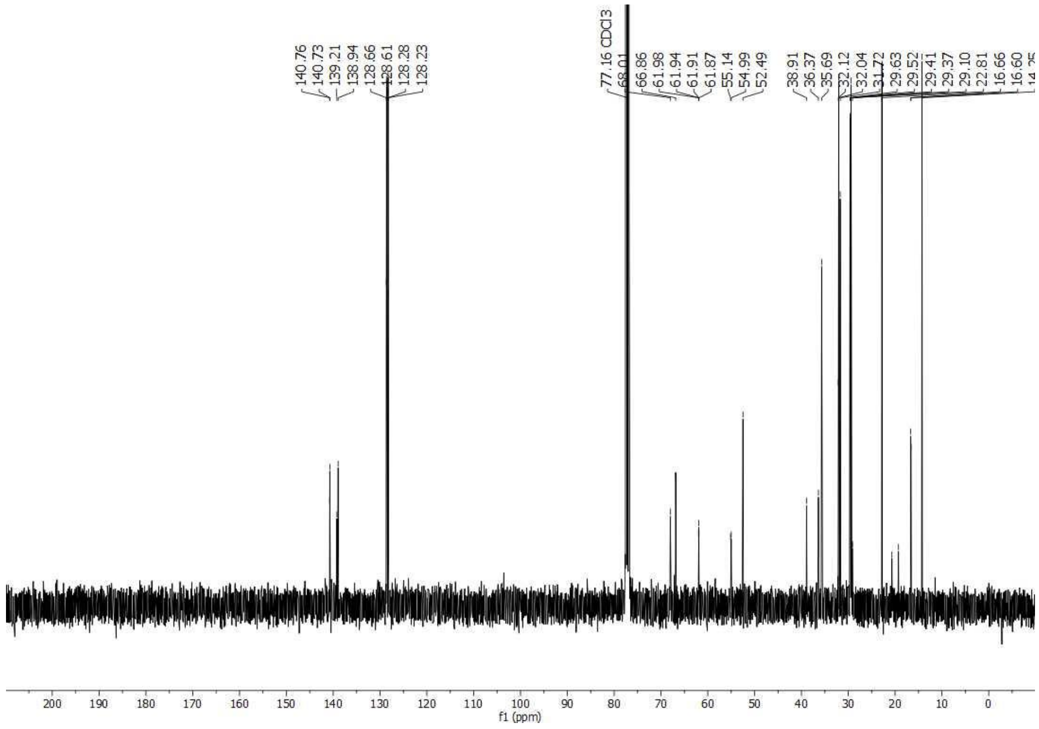

^{1}H NMR (400 MHz, $CDCl_3$)

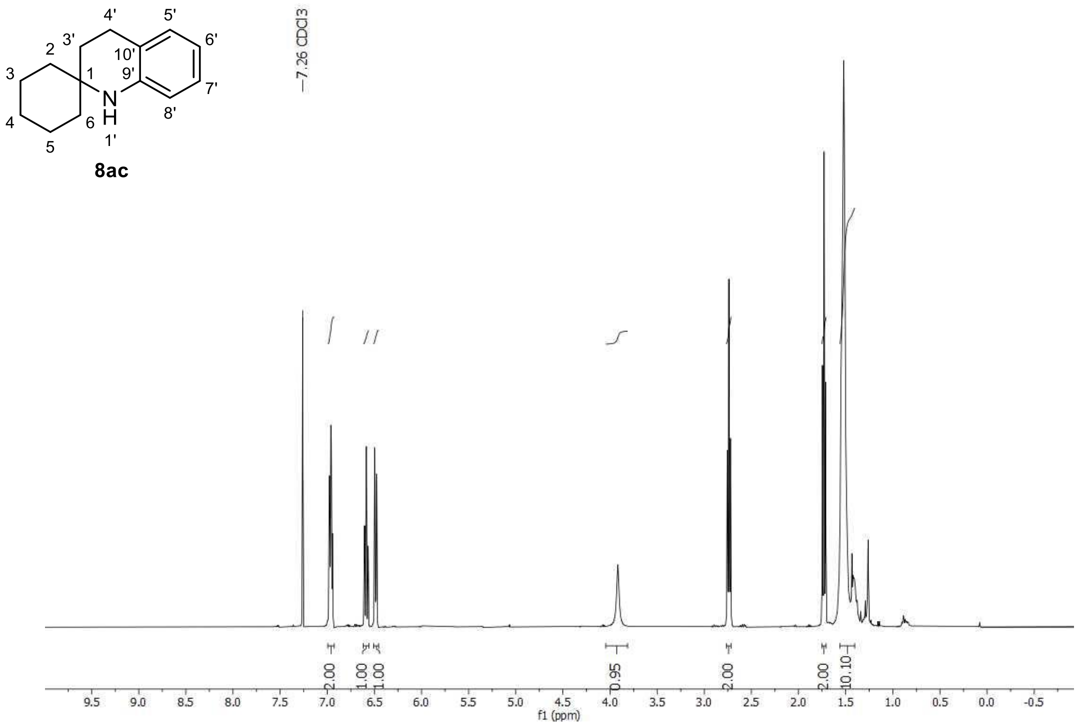

^{13}C{^{1}H} NMR (101 MHz, $CDCl_3$)

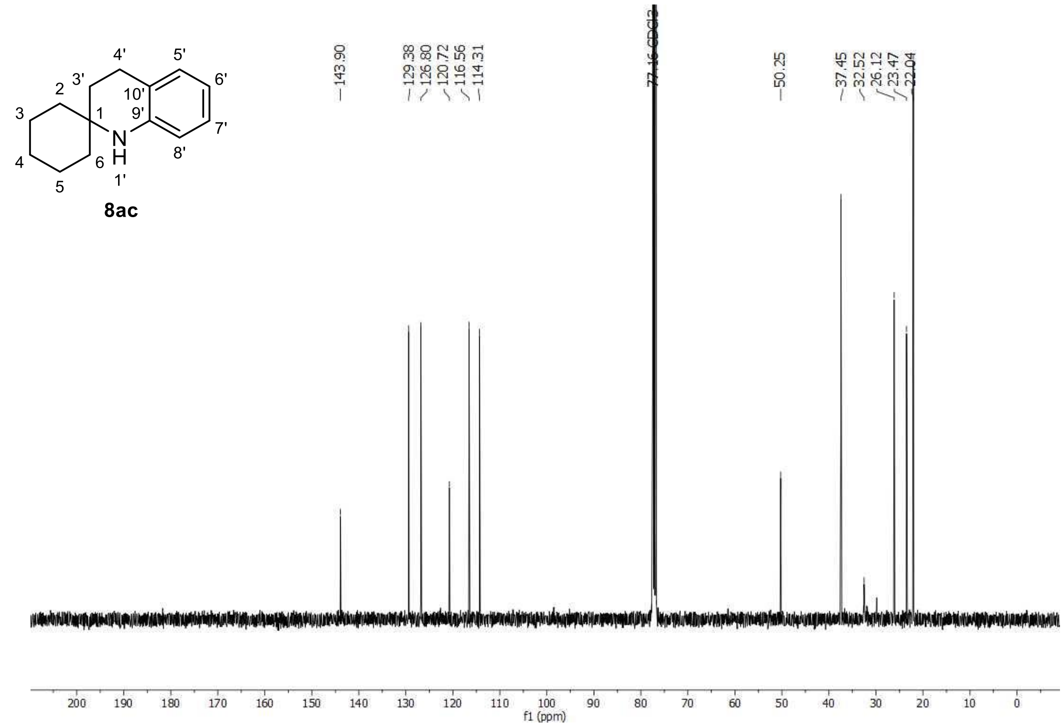

^{1}H NMR (400 MHz, $CDCl_3$)

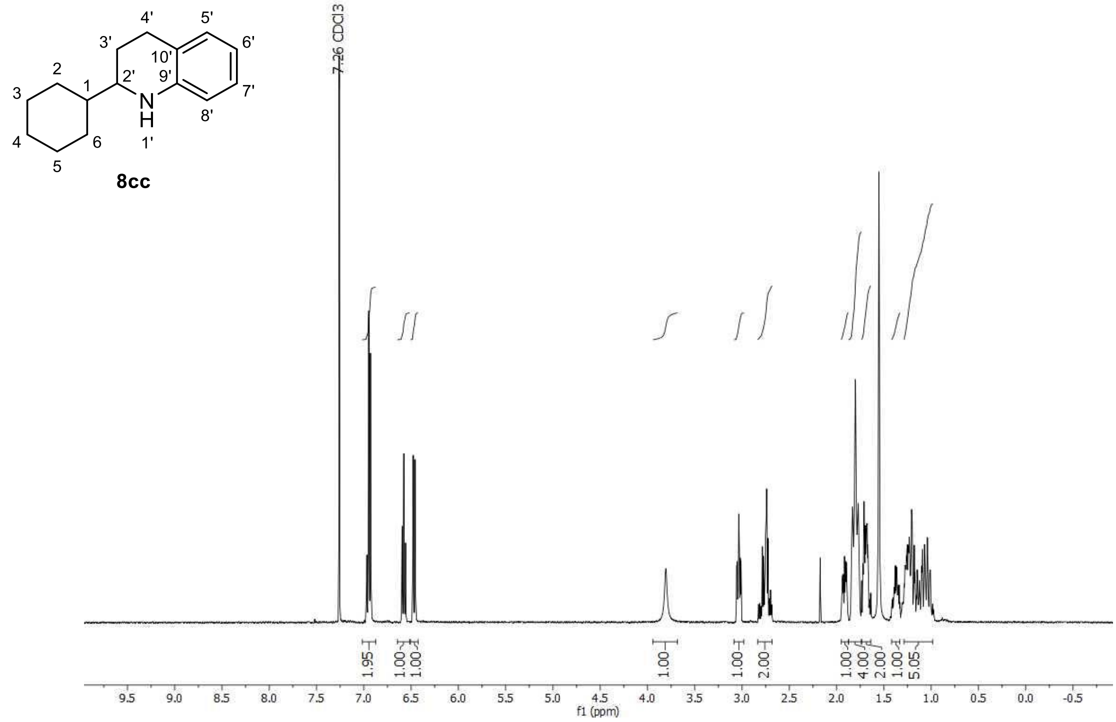

^{13}C{^{1}H} NMR (101 MHz, $CDCl_3$)

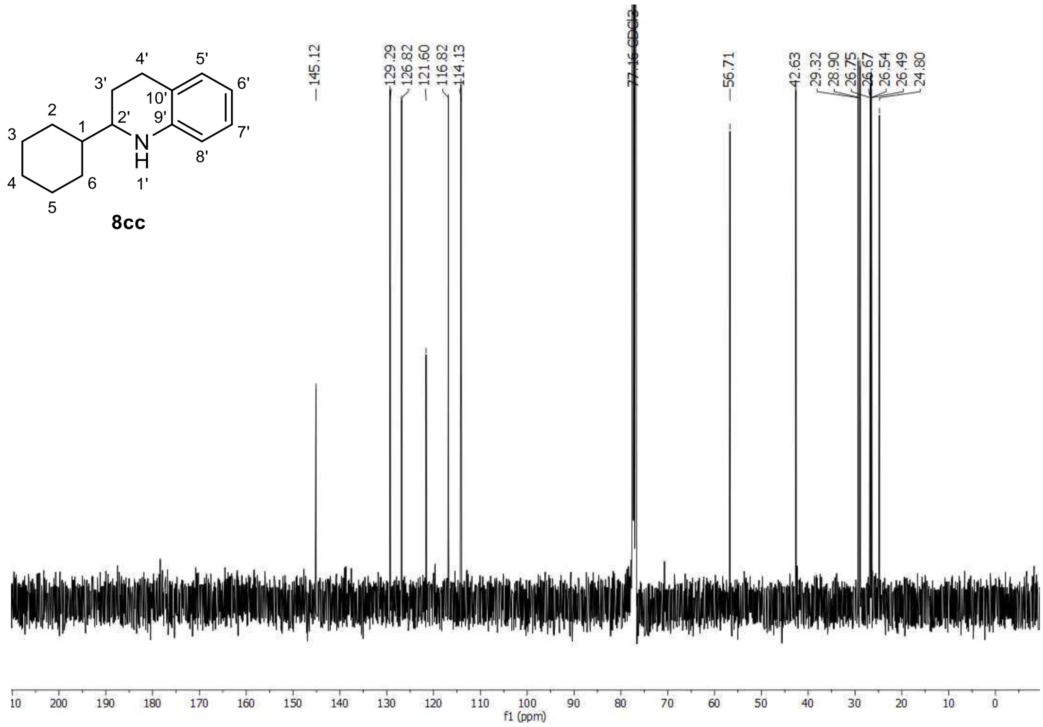

^{1}H NMR (400 MHz, CDCl₃)

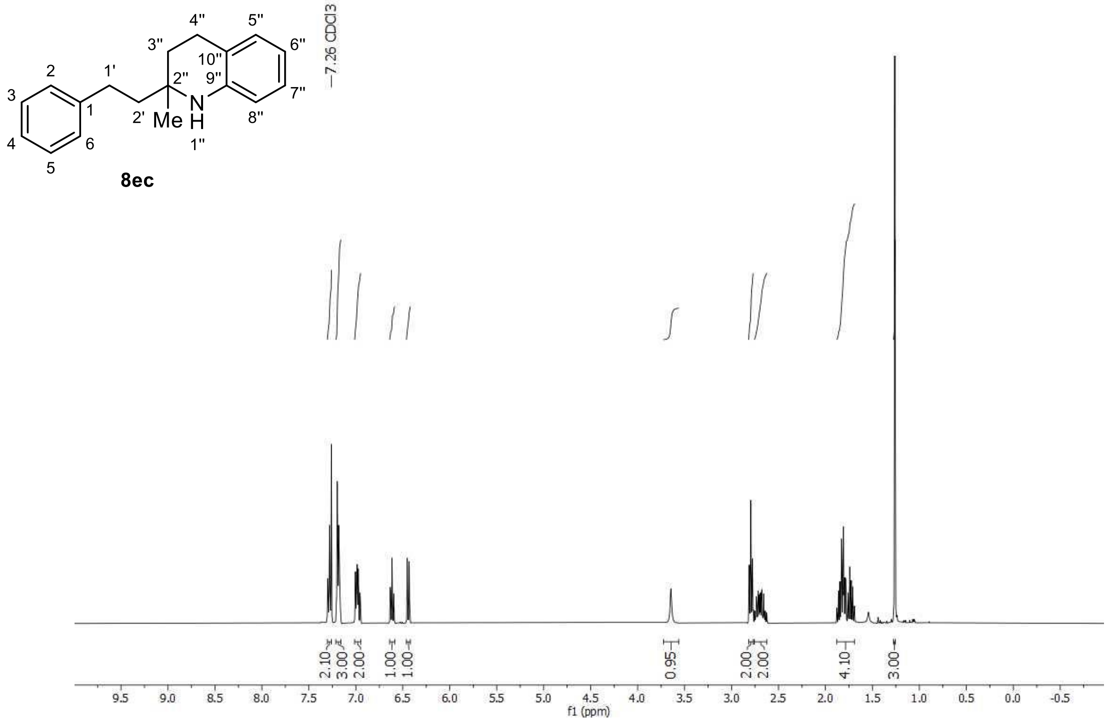

^{13}C{^{1}H} NMR (101 MHz, CDCl₃)

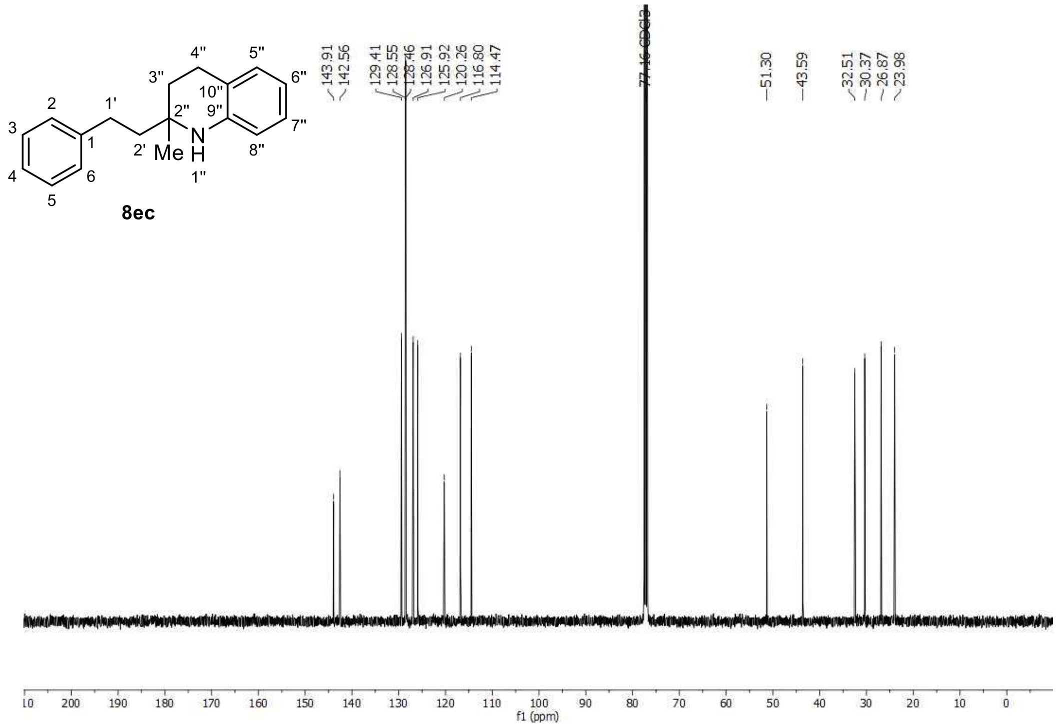

^{1}H NMR (400 MHz, CDCl$_3$)

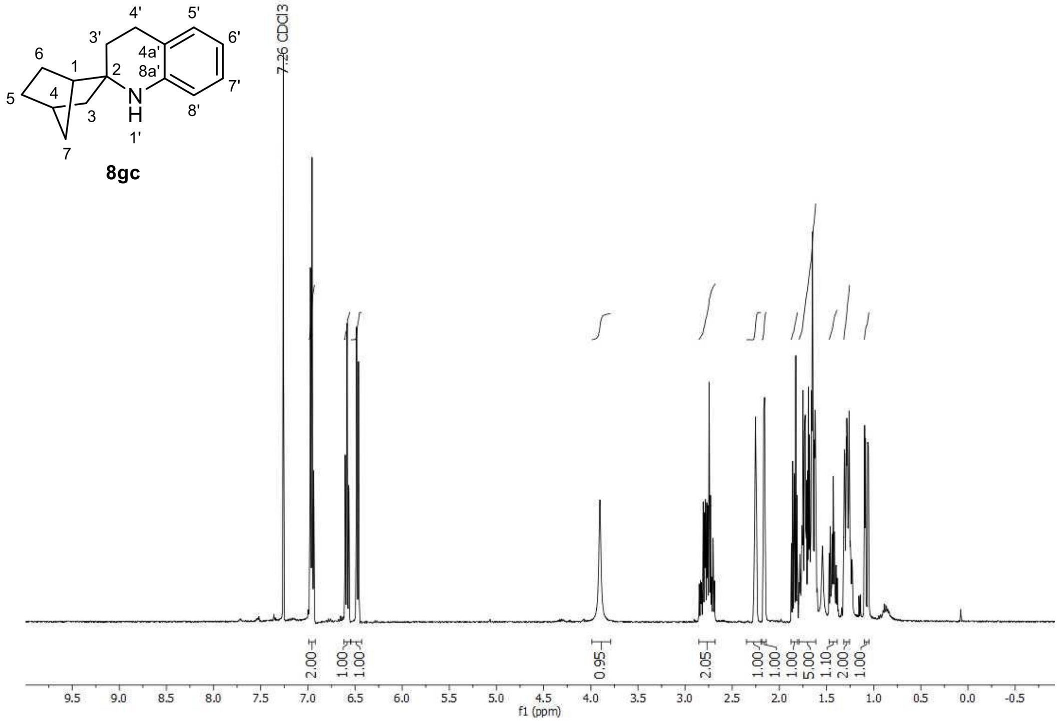

^{13}C{^{1}H} NMR (101 MHz, CDCl$_3$)

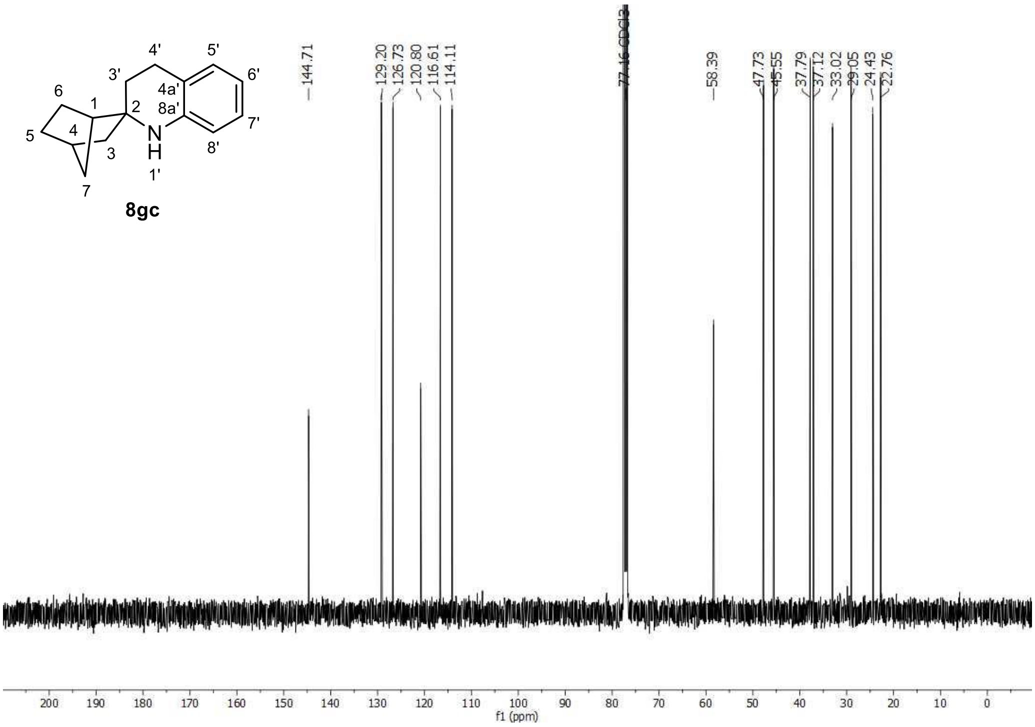

^{1}H NMR (400 MHz, CDCl3)

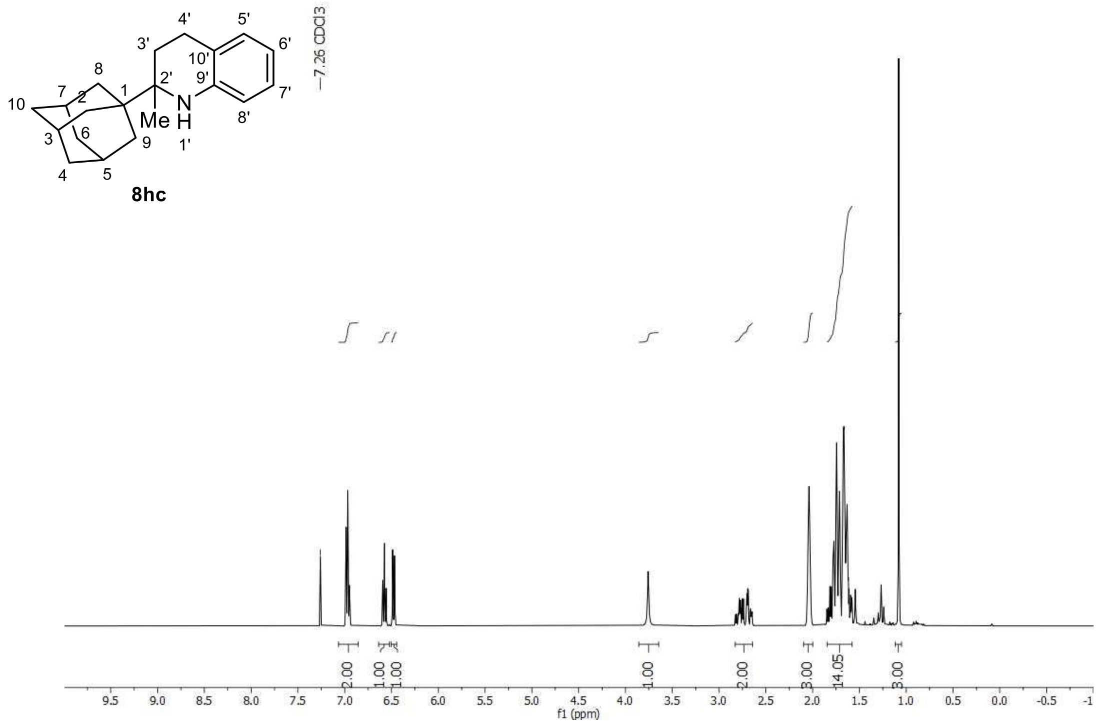

^{13}C{^{1}H} NMR (101 MHz, CDCl3)

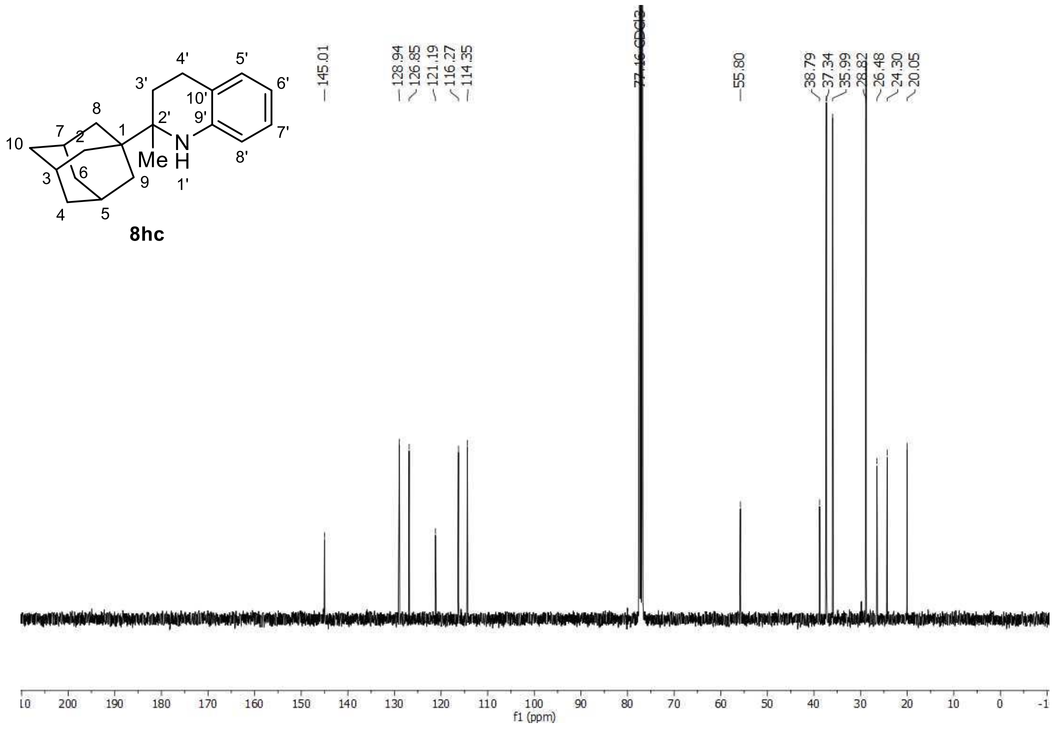

^{1}H NMR (400 MHz, CDCl3)

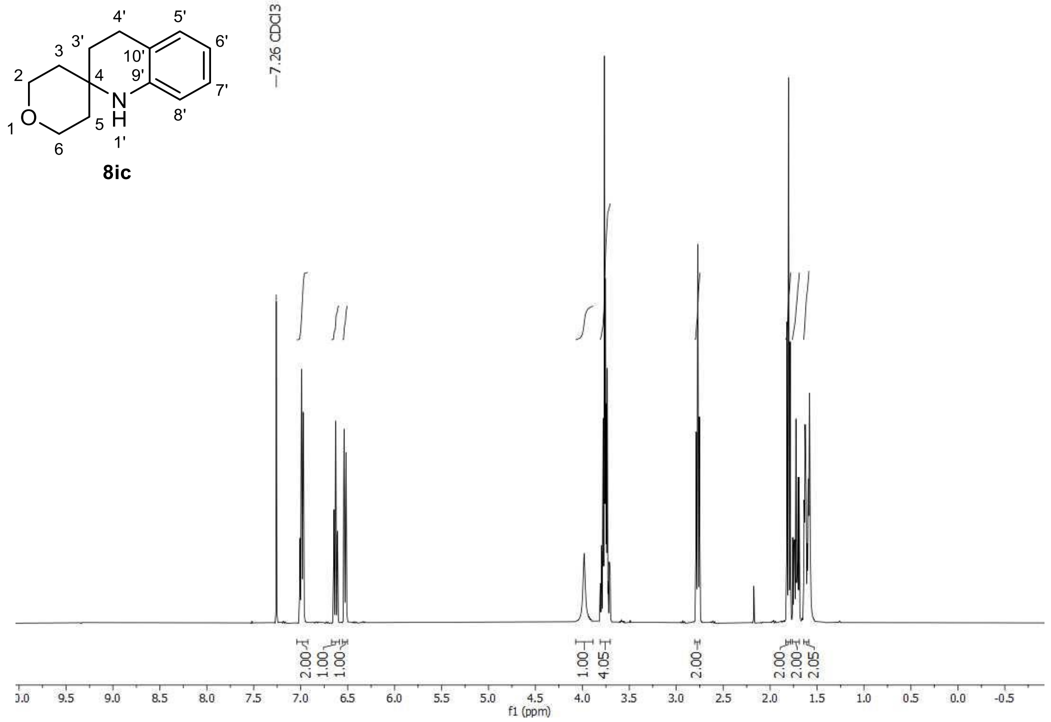

^{13}C{^{1}H} NMR (101 MHz, CDCl3)

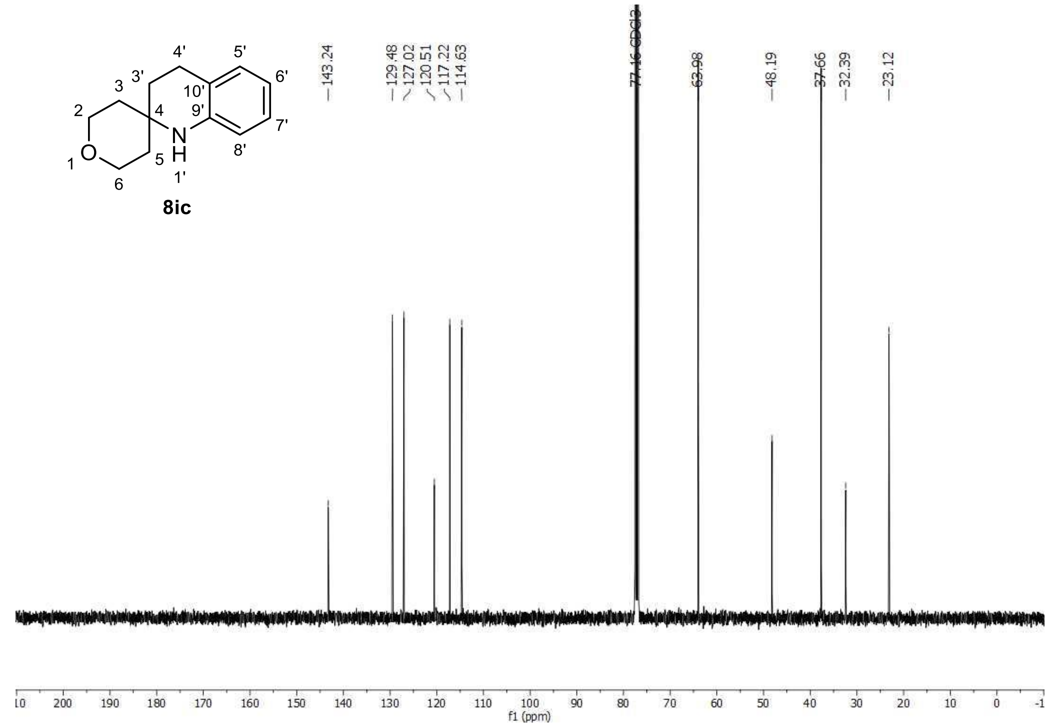

^{1}H NMR (400 MHz, $CDCl_3$)

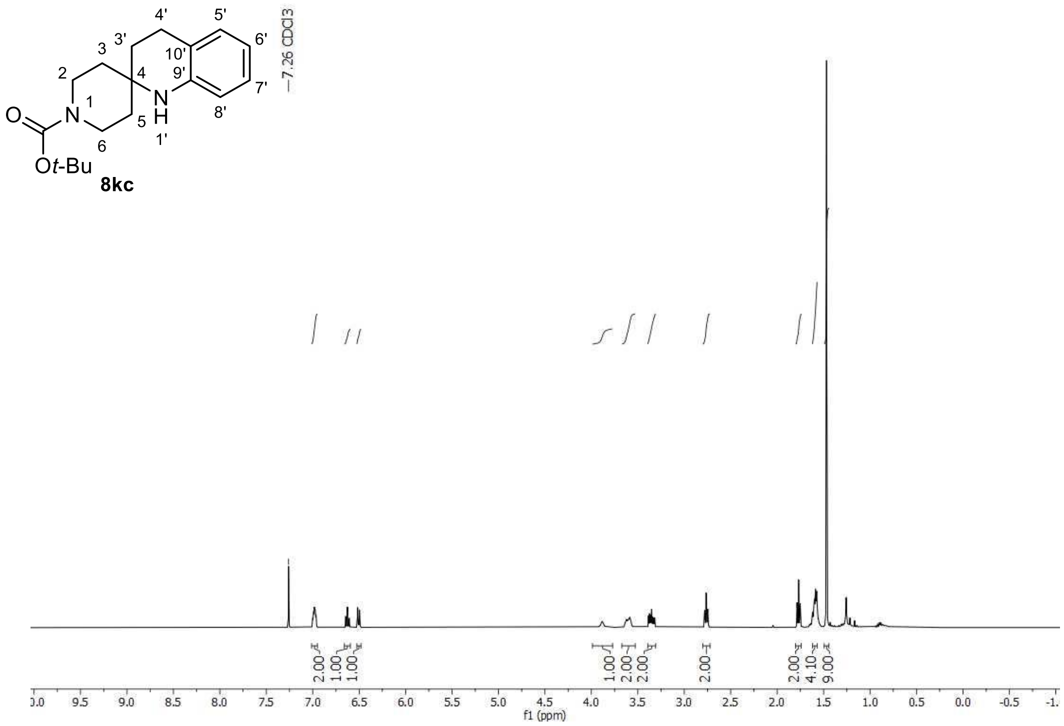

^{13}C{^{1}H} NMR (101 MHz, $CDCl_3$)

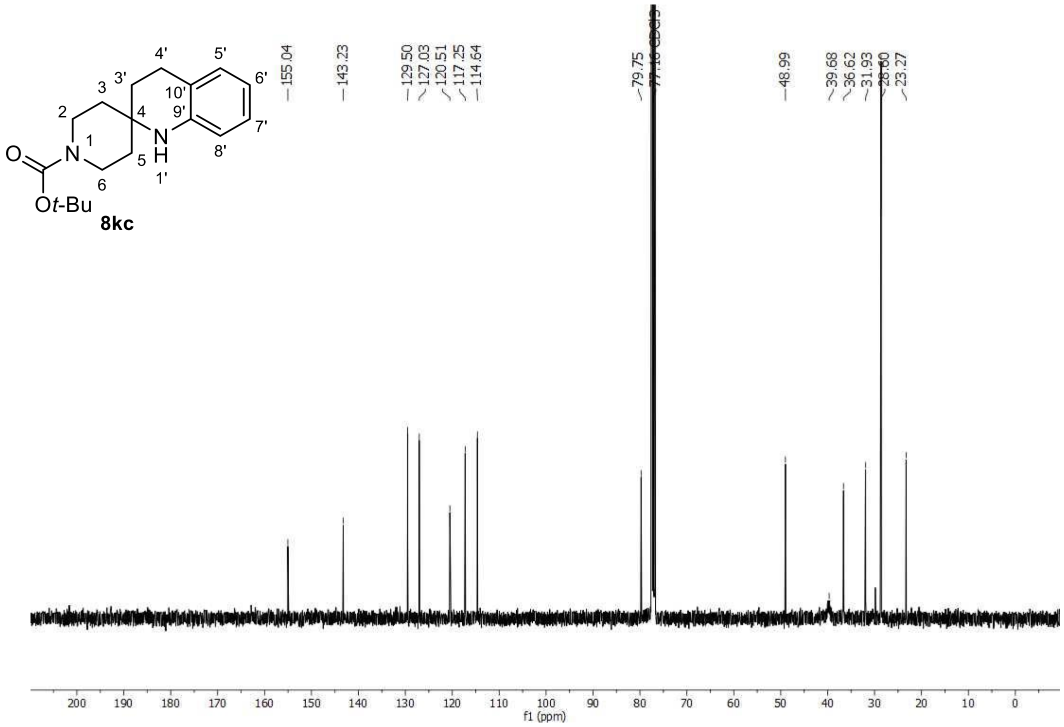

^{1}H NMR (400 MHz, CDCl$_3$)

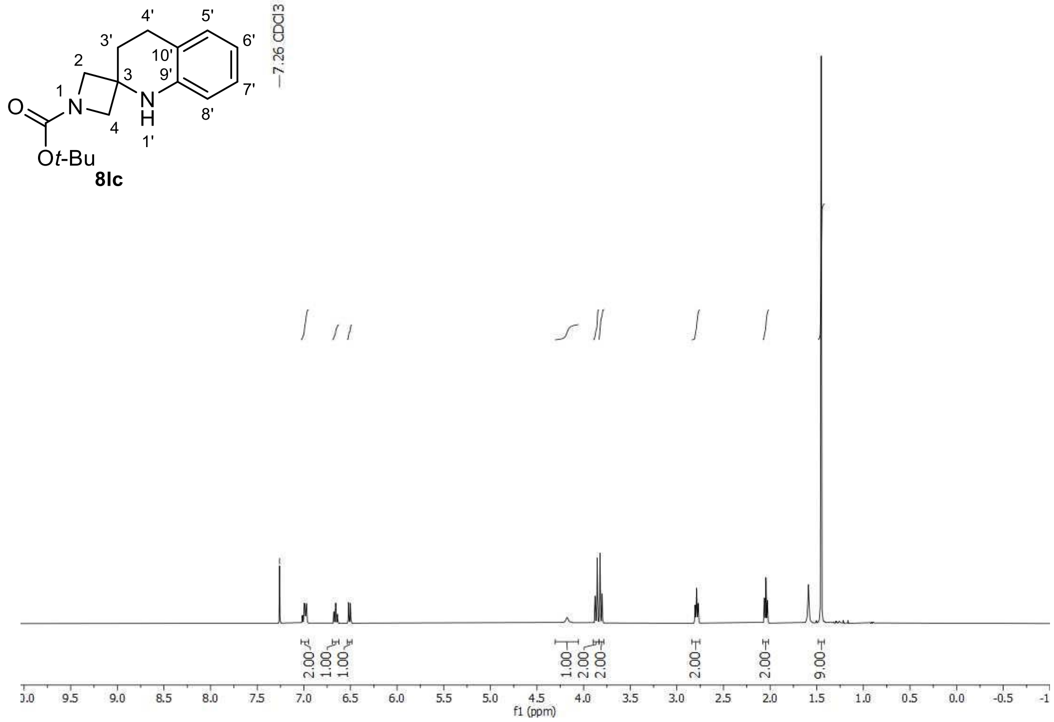

^{13}C{^{1}H} NMR (101 MHz, CDCl$_3$)

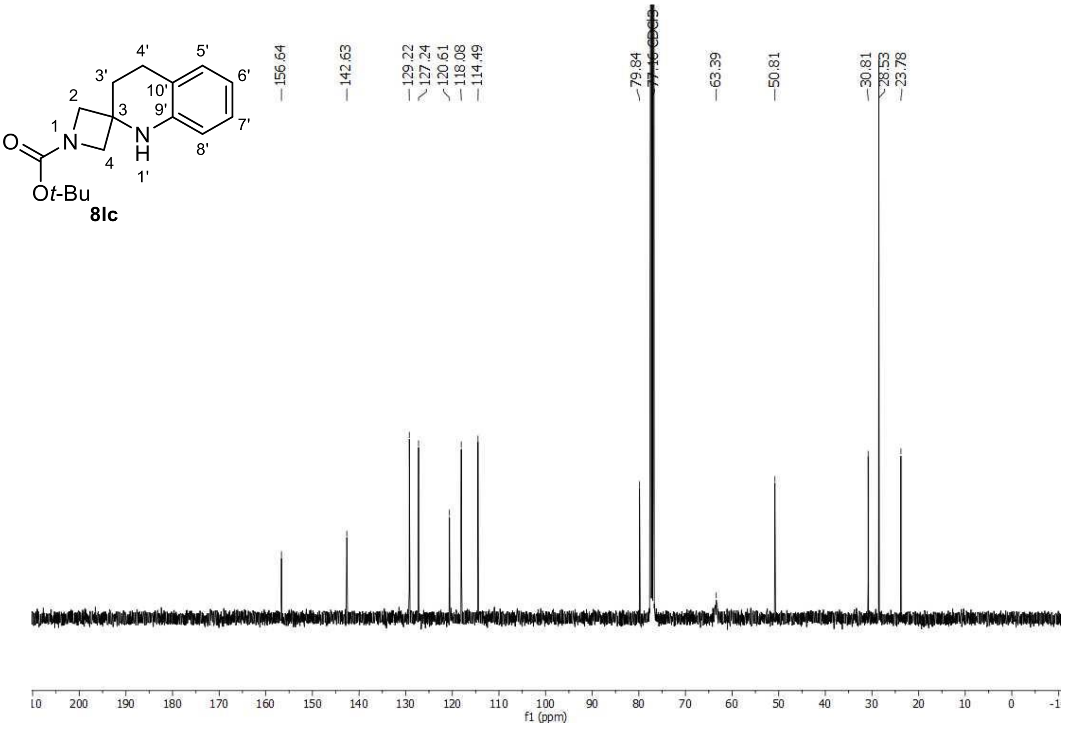

^{1}H NMR (400 MHz, $CDCl_3$)

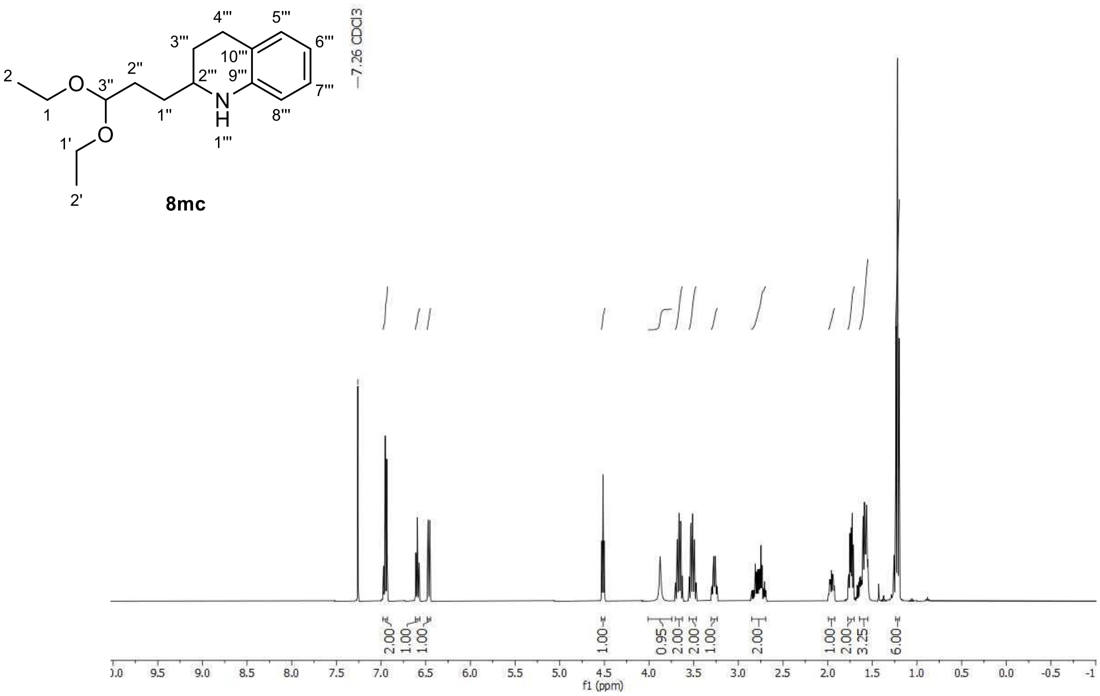

$^{13}C\{^{1}H\}$ NMR (101 MHz, $CDCl_3$)

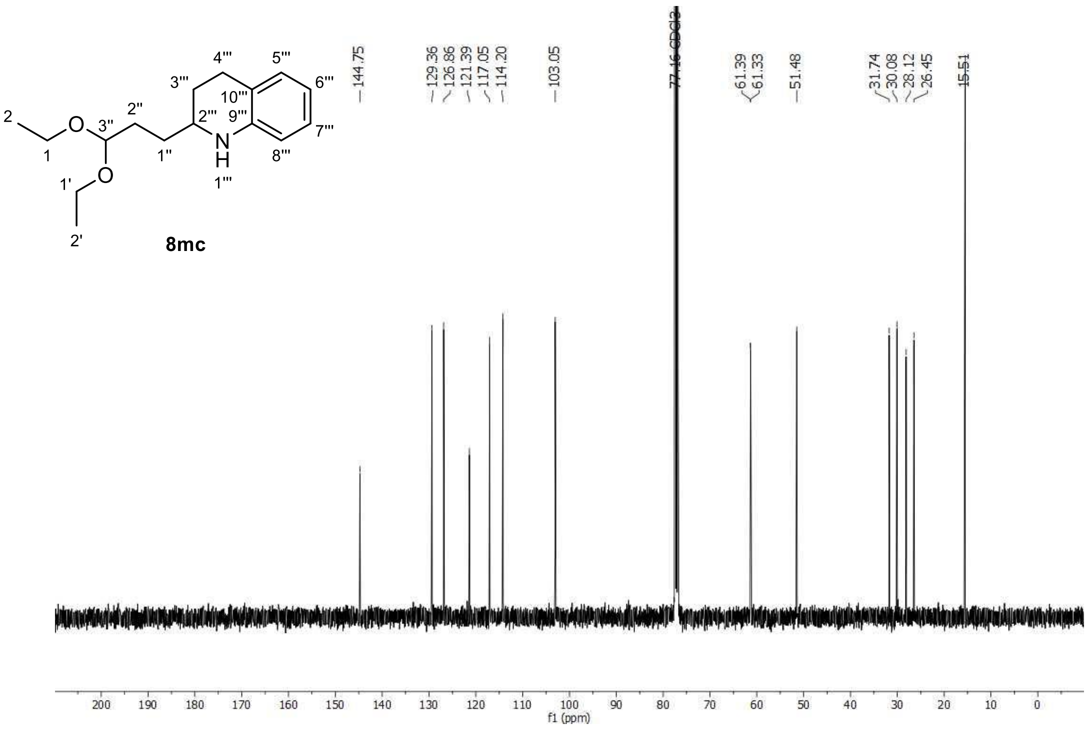

^{1}H NMR (400 MHz, CDCl3)

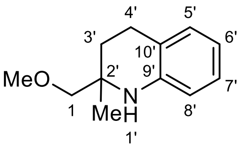

8qc

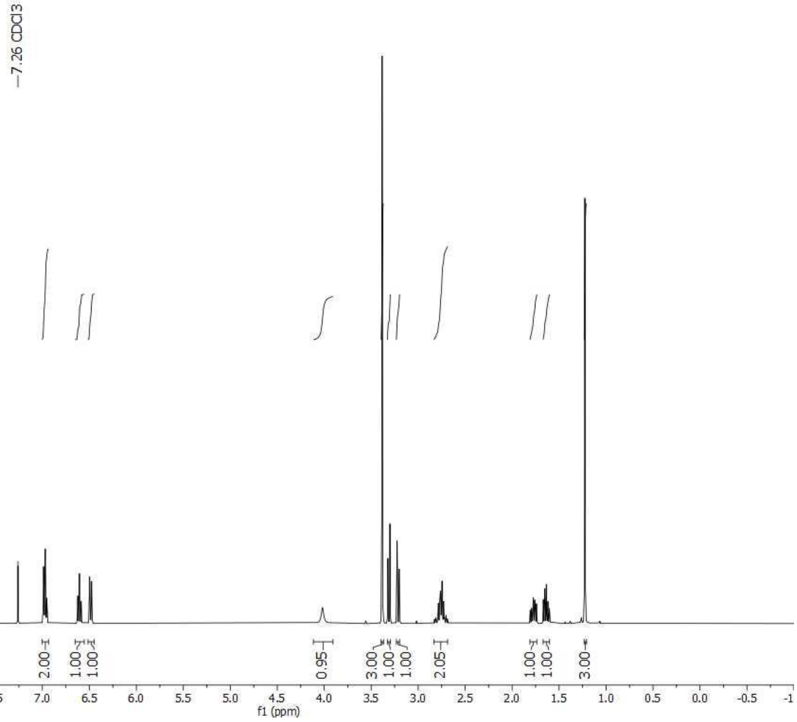

^{13}C{^{1}H} NMR (101 MHz, CDCl3)

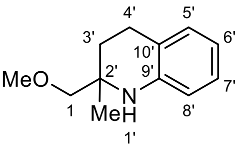

8qc

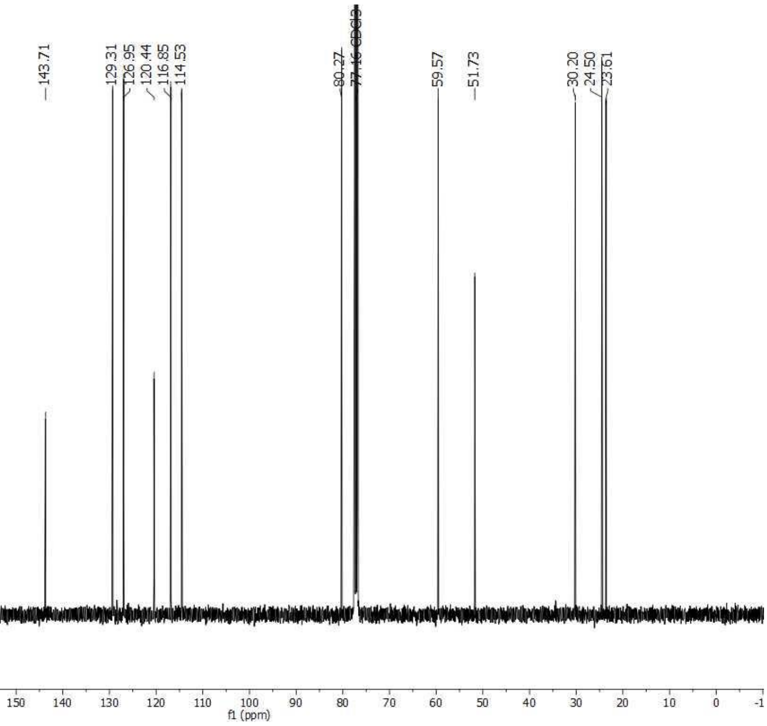

^{1}H NMR (400 MHz, $CDCl_3$)

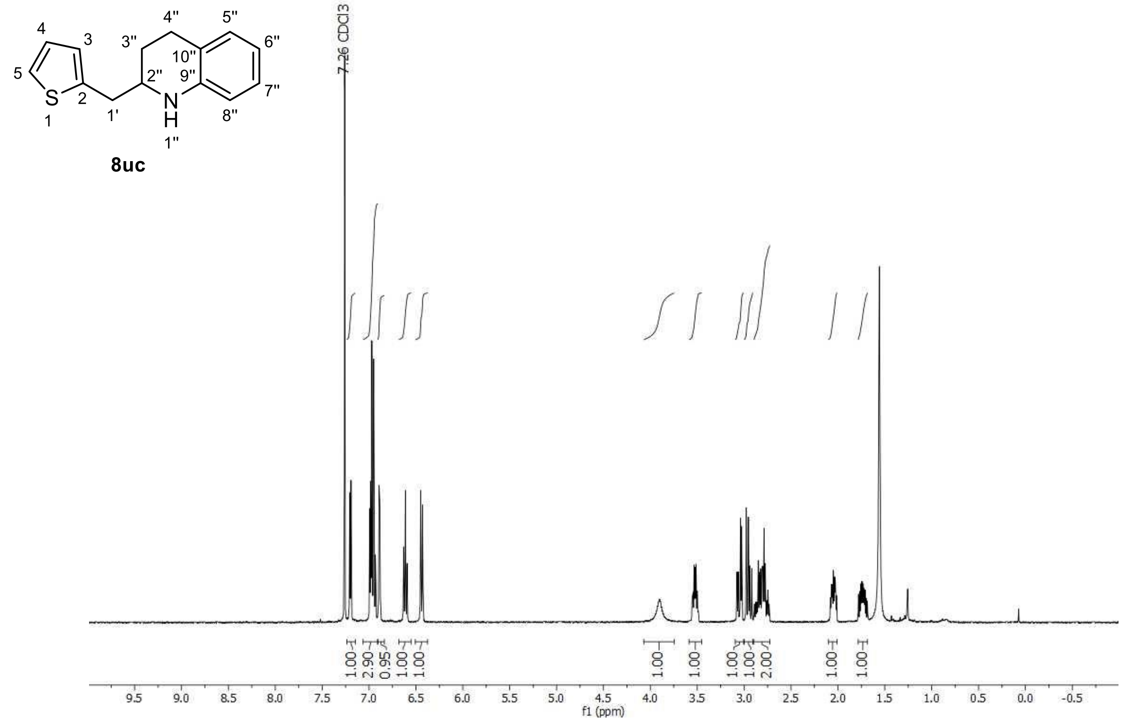

^{13}C{^{1}H} NMR (101 MHz, $CDCl_3$)

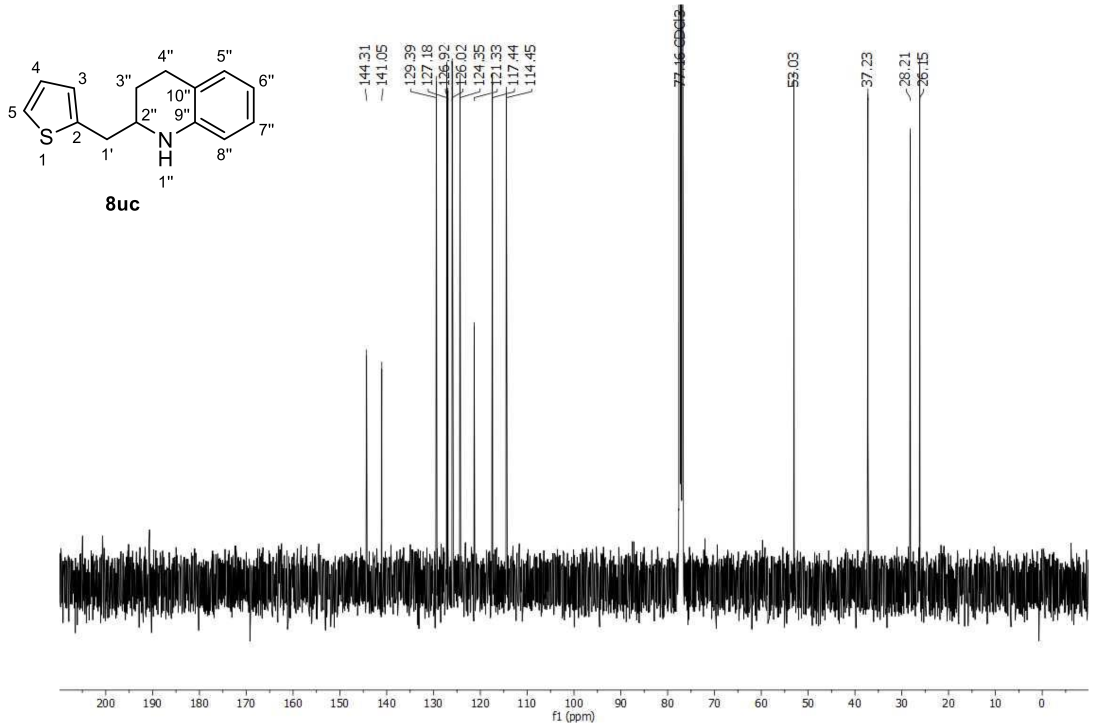

^{1}H NMR (400 MHz, CDCl₃)

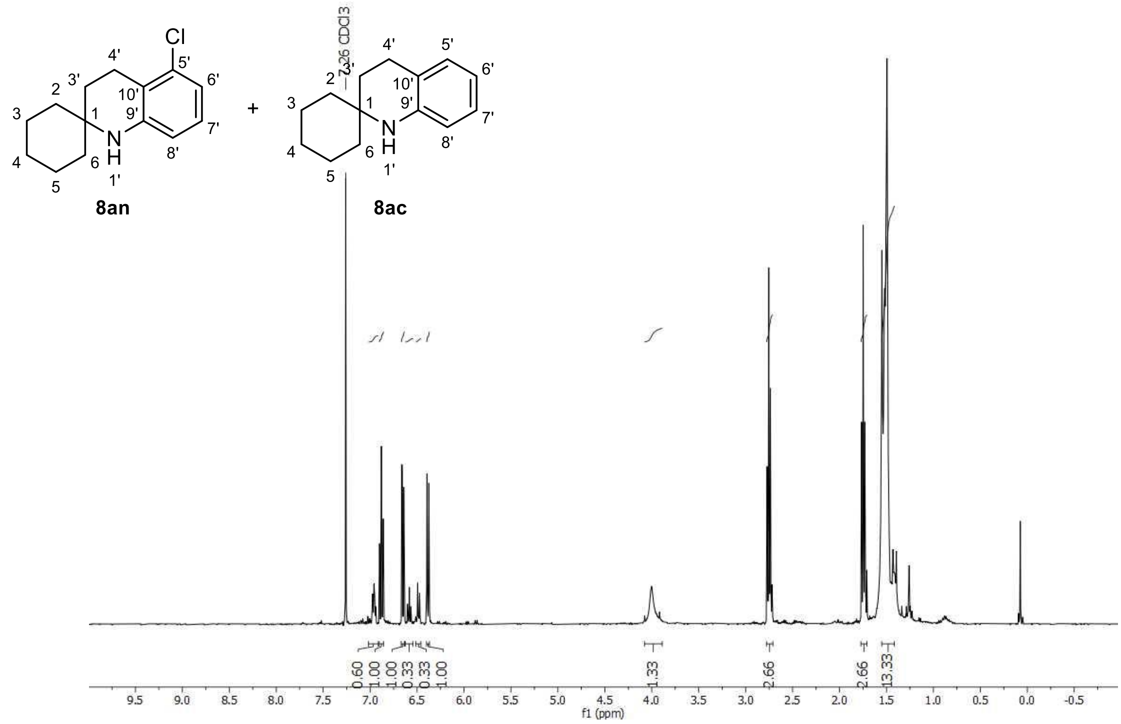

^{13}C{^{1}H} NMR (101 MHz, CDCl₃)

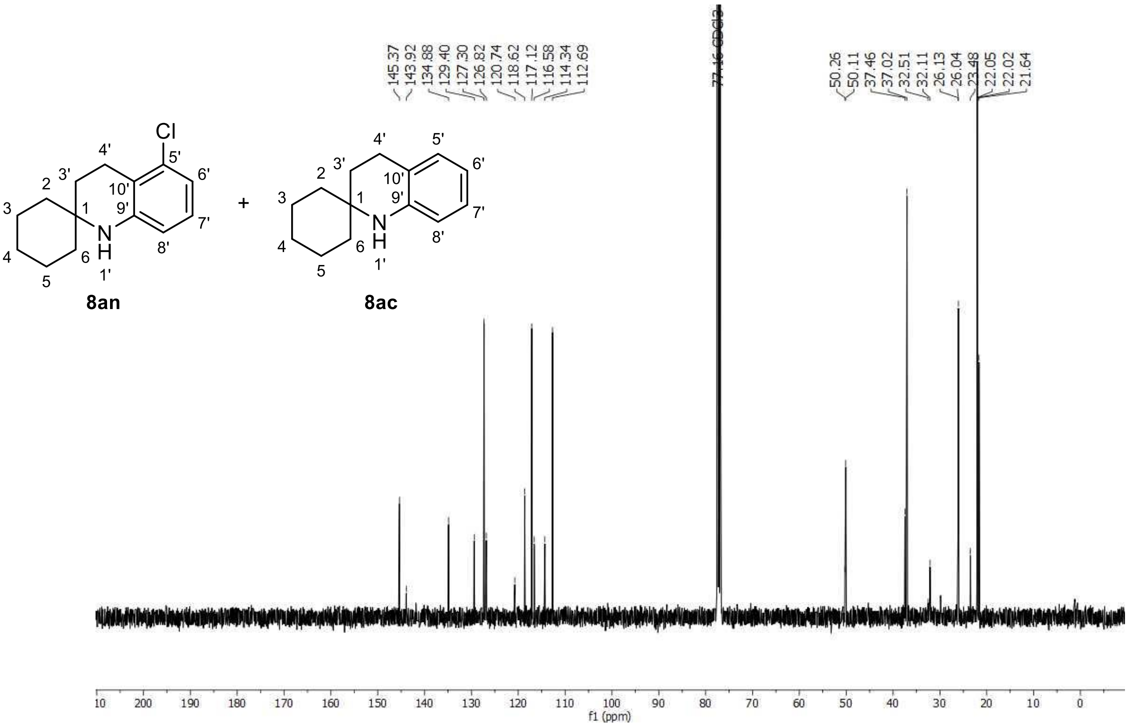

^{1}H NMR (400 MHz, CDCl$_3$)

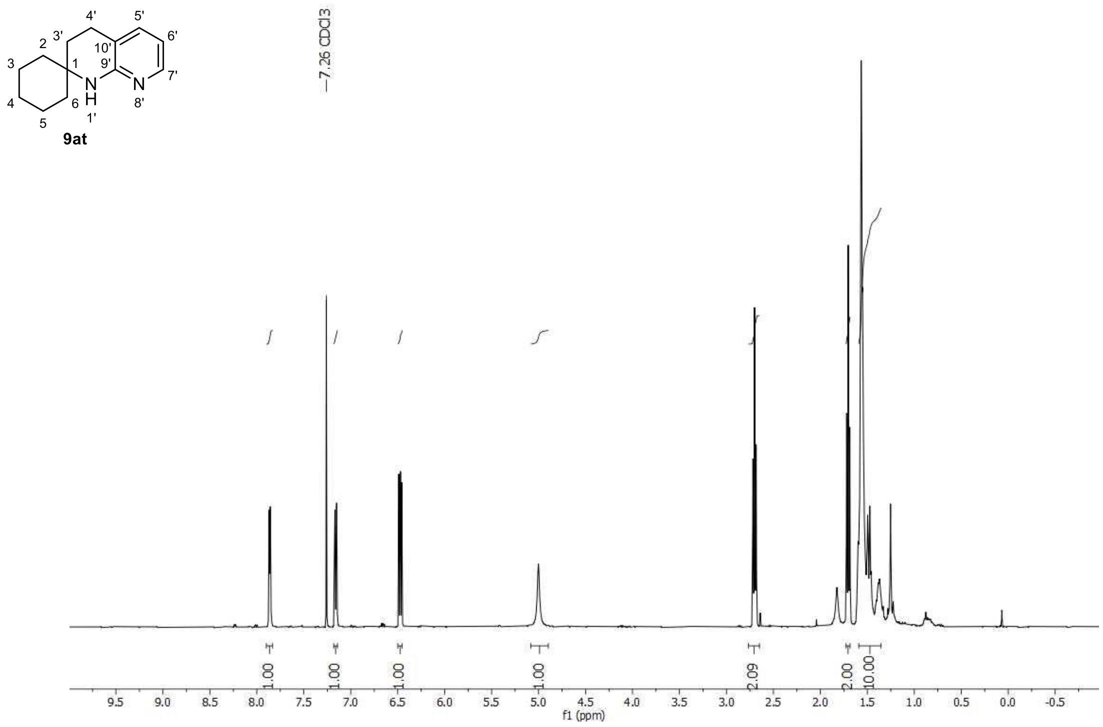

^{13}C{^{1}H} NMR (101 MHz, CDCl$_3$)

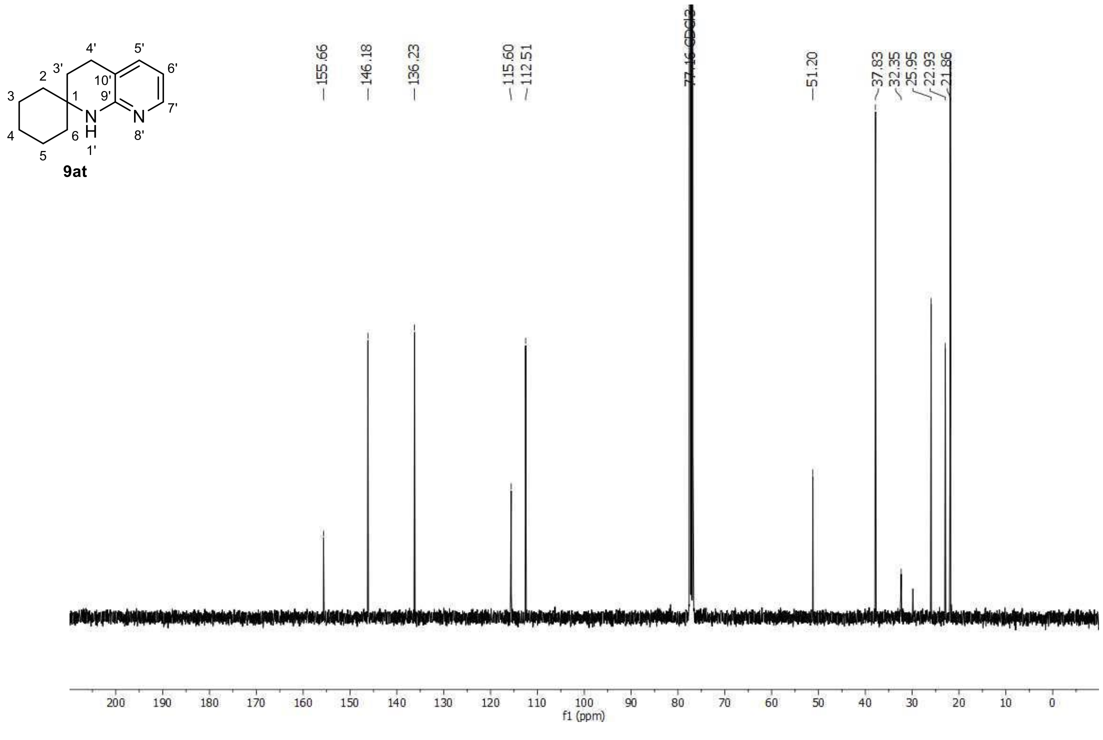

I. References and Notes

[1] Garreau, M.; Le Vaillant, F.; Waser, J. *C*-Terminal Bioconjugation of Peptides through Photoredox Catalyzed Decarboxylative Alkynylation. *Angew. Chem. Int. Ed.* **2019**, *58*, 8182–8186.

[2] Aratikatla, E. K.; Valkute, T. R.; Puri, S. K.; Srivastava, K.; Bhattacharya, A. K. Norepinephrine Alkaloids as Antiplasmodial Agents: Synthesis of Syncarpamide and Insight into the Structure-Activity Relationships of Its Analogues as Antiplasmodial Agents. *Eur. J. Med. Chem.* **2017**, *138*, 1089–1105.

[3] Campbell, K. B.; Erck, R.; Swita, M.; Cosimbescu, L. Multifunctional Tunable Polymethacrylates for Enhanced Shear Stability and Wear Prevention. *ACS Appl. Polym. Mater*. **2020**, *2*, 2839–2848.

[4] Liu, Z.; Hu, B.-H.; Messersmith, P. B. Acetonide Protection of Dopamine for the Synthesis of Highly Pure *N*-Docosahexaenoyldopamine. *Tetrahedron Lett.* **2010**, *51*, 2403–2405.

[5] Gentry, E. C.; Rono, L. J.; Hale, M. E.; Matsuura, R.; Knowles, R. R. Enantioselective Synthesis of Pyrroloindolines *via* Noncovalent Stabilization of Indole Radical Cations and Applications to the Synthesis of Alkaloid Natural Products. *J. Am. Chem. Soc.* **2018**, *140*, 3394–3402.

[6] Liu, K.; Jiang, H.-J.; Li, N.; Li, H.; Wang, J.; Zhang, Z.-Z.; Yu, J. Enantioselective Bromocyclization of Tryptamines Induced by Chiral Co(III)-Complex-Templated Brønsted Acids under an Air Atmosphere. *J. Org. Chem.* **2018**, *83*, 6815–6823.

[7] Jacquemard, U.; Bénéteau, V.; Lefoix, M.; Routier, S.; Mérour, J.-Y.; Coudert, G. Mild and Selective Deprotection of Carbamates with Bu_4NF. *Tetrahedron* **2004**, *60*, 10039–10047.

[8] Speckmeier, E.; Fischer, T. G.; Zeitler, K. A Toolbox Approach To Construct Broadly Applicable Metal-Free Catalysts for Photoredox Chemistry: Deliberate Tuning of Redox Potentials and Importance of Halogens in Donor–Acceptor Cyanoarenes. *J. Am. Chem. Soc.* **2018**, *140*, 15353–15365.

[9] Liu, Y.; Ge, H. Site-Selective C–H Arylation of Primary Aliphatic Amines Enabled by a Catalytic Transient Directing Group. *Nat. Chem.* **2017**, *9*, 26–32.

[10] Ryder, A. S. H.; Cunningham, W. B.; Ballantyne, G.; Mules, T.; Kinsella, A. G.; Turner-Dore, J.; Alder, C. M.; Edwards, L. J.; McKay, B. S. J.; Grayson, M. N.; Cresswell, A. J. Photocatalytic α-Tertiary Amine Synthesis *via* C−H Alkylation of Unmasked Primary Amines. *Angew. Chem. Int. Ed.* **2020**, *59*, 14986–14991.

[11] Calleja, J.; Pla, D.; Gorman, T. W.; Domingo, V.; Haffemayer, B.; Gaunt, M. J. A Steric Tethering Approach Enables Palladium-Catalysed C–H Activation of Primary Amino Alcohols. *Nat. Chem.* **2015**, *7*, 1009–1016.

[12] Kim, J.-W.; Kim, Y.-W.; Inagaki, Y.; Hwang, Y.-A.; Mitsutake, S.; Ryu, Y.-W.; Lee, W. K.; Ha, H.-J.; Park, C.-S.; Igarashi, Y. Synthesis and Evaluation of Sphingoid Analogs as Inhibitors of Sphingosine Kinases. *Bioorg. Med. Chem.* **2005**, *13*, 3475–3485.

[13] Wu, J.; Wang, C.; Tang, W.; Pettman, A.; Xiao, J. The Remarkable Effect of a Simple Ion: Iodide-Promoted Transfer Hydrogenation of Heteroaromatics. *Chem. Eur. J.* **2012**, *18*, 9525–9529.

[14] Pérez-Fuertes, Y.; Kelly, A. M.; Johnson, A. L.; Arimori, S.; Bull, S. D.; James, T. D. Simple protocol for NMR analysis of the enantiomeric purity of primary amines. *Org. Lett.* **2006**, *8*, 609–612.
